# Controlling π-Conjugated Polymer–Acceptor Interactions by Designing Polymers with a Mixture of π-Face Strapped and Nonstrapped Monomers

**DOI:** 10.1021/acs.macromol.3c00175

**Published:** 2023-04-25

**Authors:** Fatima Hameed, Manikandan Mohanan, Nafisa Ibrahim, Charles Ochonma, Joaquín Rodríguez-López, Nagarjuna Gavvalapalli

**Affiliations:** † Department of Chemistry, Georgetown University, Washington, D.C. 20057, United States; ‡ Institute for Soft Matter Synthesis and Metrology, Georgetown University, Washington, D.C. 20057, United States; § Department of Chemistry, University of Illinois Urbana-Champaign, Urbana, Illinois 61801, United States

## Abstract

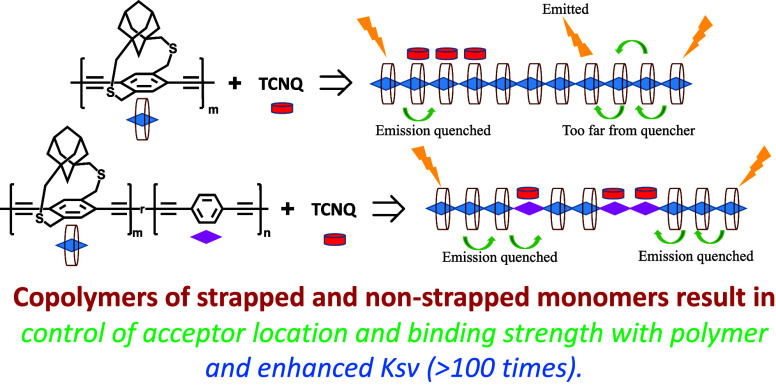

Controlling π-conjugated polymer–acceptor complex interaction, including the interaction strength and location along the polymer backbone, is central to organic electronics and energy applications. Straps in the strapped π-conjugated polymers mask the π-face of the polymer backbone and hence are useful to control the interactions of the π-face of the polymer backbone with other polymer chains and small molecules compared to the conventional pendant solubilizing chains. Herein, we have synthesized a series of strapped π-conjugated copolymers containing a mixture of strapped and nonstrapped comonomers to control the polymer–acceptor interactions. Simulations confirmed that the acceptor is directed toward the nonstrapped repeat unit. More importantly, strapped copolymers overcome a major drawback of homopolymers and display higher photoinduced photoluminescence (PL) quenching, which is a measure of electron transfer from the polymer to acceptor, compared to that of both the strapped homopolymer and the conventional polymer with pendant solubilizing chains. We have also shown that this strategy applies not only to strapped polymers, but also to the conventional polymers with pendant solubilizing chains. The increase in PL quenching is attributed to the absence of a steric sheath around the comonomers and their random location along the polymer backbone, which enhances the probability of non-neighbor acceptor binding events along the polymer backbone. Thus, by mixing insulated and noninsulated monomers along the polymer backbone, the location of the acceptor along the polymer backbone, polymer–acceptor interaction strength, and the efficiency of photoinduced charge transfer are controllable compared to the homopolymers.

## Introduction

Strapped π-conjugated polymers, wherein the repeat units contain straps, show enhanced chemical stability, photostability, fluorescence quantum yield, electroluminescence, and intrachain charge transport due to the insulated π-face of the polymer backbone.^1−17^ For example, Takeuchi and Sugiyasu have demonstrated that strapped π-conjugated polymers are thermo-formable like conventional plastics.^11^ Bronstein and co-workers have shown that strapped π-conjugated polymers hinder interchain interactions and enhance solid-state quantum yields.^8,10,18^ The Smith group has used aryl straps^9,19^ to reduce interchain interactions and enhance the sensory response toward nitroaromatic vapors in the solid state similar to pentiptycene polymers derived by the Swager^20^ group. So far, most of the studies in this research area have been focused on taking advantage of the π-face sheathing capability by the straps and developing conjugated polymers with enhanced chemical stability, photostability, fluorescence quantum yield, electroluminescence, and intrachain charge transport.^1−13,17−19,21^ However, their use in organic electronics and energy applications is limited due to insulation of the π-face, which hampers the interaction of the polymer backbone with acceptor molecules.

Our group has been focused on developing molecular design strategies that take advantage of the straps in the strapped polymers in organic electronics and energy applications, especially for controlling the π-conjugated polymer–acceptor interactions.^22,23^ Controlling the location of the dopant along the polymer backbone is considered one of the critical factors in determining charge generation, conductivity, and thermoelectric performance of π-conjugated polymers.^24−34^ Typically, varying the structural parameters of the pendant chains and their location along the polymer backbone has been the widely used strategy to control the polymer-dopant (acceptor molecule in the case of a *p*-type strapped polymer) complex formation.^24−33^ Recently, we have shown that straps can be used to control the location of the acceptor along a trimer backbone. The dopant molecule is directed toward the nonstrapped repeat units in trimers containing mixture of strapped and nonstrapped repeat units.^23^ Also, the dopant ionization is higher for the trimers containing a mixture of strapped and nonstrapped units than the analogous nonstrapped trimer.

**Scheme 1:**
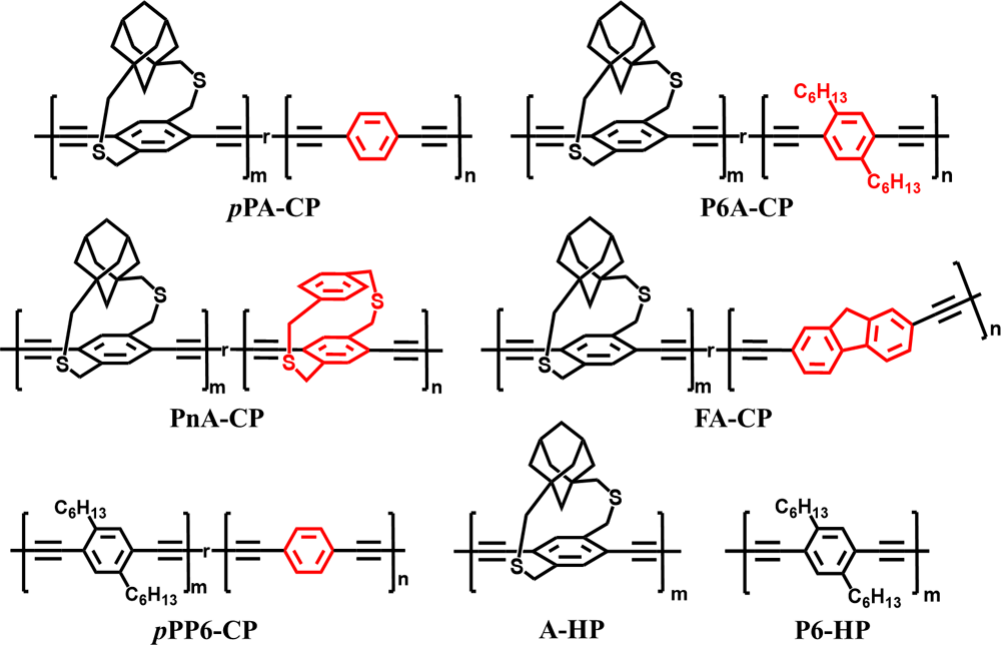
Homopolymers and Copolymers Synthesized and Studied in This Work. *n* = ∼20% in the Copolymers

π-Conjugated polymers rather than trimers are more desirable for electronics and energy applications. In this work, a series of strapped copolymers containing a mixture of strapped and nonstrapped repeat units are synthesized. We hypothesize that the location of the dopant along the π-conjugated polymer backbone and the interaction strength with the polymer can be controlled by varying the nonstrapped repeat unit structure in the strapped copolymers. Since the nonstrapped monomers have no insulating sheath around them compared to the strapped monomers, the acceptor units interact with the nonstrapped units along the polymer backbone. In addition to controlling the location of the acceptor along the polymer backbone, it is also desired to have an efficient electron transfer from the polymer to acceptor for electronics and energy applications.^1,3^ To achieve this, a series of strapped copolymers containing 20 mole % randomly incorporated nonstrapped repeat units are synthesized (Scheme 1). The impact of the structure of the nonstrapped monomers on the strapped copolymers interaction with the acceptor and photoinduced photoluminescence (PL) quenching, which is a measure of electron transfer from the polymer to the acceptor,^22,35−40^ is studied. Simulations are performed on the trimers corresponding to each of the polymers to determine the location of the acceptor along the trimer and their interaction strength. Structural and optical characterization studies of the copolymers including fluorescence quenching studies are done to determine the impact of the comonomer structure on the polymer PL quenching.

## Results and Discussion

**Scheme 2:**
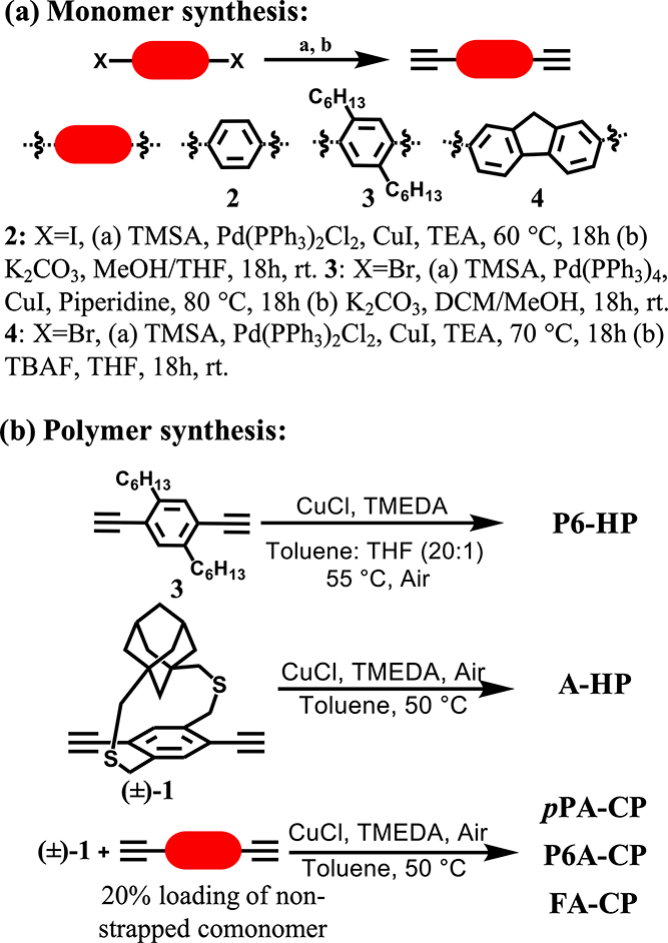
(a) Synthesis of Nonstrapped Aryl Comonomers; (b) Synthesis of Homo and Copolymers Using Glaser–Hay Polymerization, (±)-**1**: Comonomer Ratio in the Copolymers is 80:20

Strapped monomer, (±)-diethynyl adamantanocyclophane monomer ((±)-**1**), was synthesized from 1,3-adamantane dicarboxylic acid following our previously reported protocols.^17^ Structurally diverse nonstrapped aryldiacetylene comonomers (**2–4**) were synthesized following the reported synthetic protocols (Scheme 2).^41−44^ Random strapped copolymers of strapped ((±)-**1)** and nonstrapped aryl monomer (**2–4**) were synthesized following the Glaser–Hay polymerization protocol used for the adamantanocyclophane homopolymer synthesis.^17^ All the strapped copolymers were synthesized using a 20% nonstrapped aryl comonomer feed ratio. In a typical procedure, 80 mole % of strapped monomer ((±)-**1**) and 20 mole % of nonstrapped aryl comonomer (**2** to **4)** were reacted in the presence of copper(I) chloride and tetramethylethylenediamine in toluene in the presence of air at 50 °C (Scheme 2). The polymerization reaction mixture was added to methanol to stop the polymerization, and the resultant polymer precipitate was purified by soxhlation using methanol and chloroform. The chloroform solution was concentrated under vacuum and reprecipitated in ether, filtered, dried, and used for further characterization. The percent inclusion of nonstrapped aryl comonomer units into the copolymers was determined using proton nuclear magnetic resonance (^1^H NMR) analysis (Table 1 and Figures S56–59). The ratio of the integration of the aryl protons corresponding to strapped monomer ((±)-**1)** and aryl comonomers (**2–4**) was used to determine the percent incorporation of the nonstrapped aryl comonomers (**2–4**) into the copolymers. Based on the ^1^H NMR analysis, all the copolymers contained 16–20 mole % of the nonstrapped aryl comonomer. All the generated copolymers are atactic in nature, i.e., there is no control over the orientation of the cyclophane units along the polymer backbone since the racemic mixture of (±)-**1** was used to synthesize the copolymers. Previously, we have shown that the reactivity ratios of the diethynyl dithia[3.3] paracyclophane monomer and (±)-**1** are the same resulting in a random copolymer (PnA-CP).^22^ The reactivity of the nonstrapped aryl monomers **2–4** is expected to be similar to that of (±)-**1** as the ethynyls are far from the adamantyl strap, and the electronic nature of the phenyl core is not altered significantly. Hence, the polymers synthesized in this work are treated as random copolymers.

**Table 1:** Comprehensive Optical and Electrochemical Properties of Polymers

polymer	Mn/Đ (kDa)	% co-monomer incorporation^a^	λ_max_^abs^		λ_max_^em^		molar extinction coefficient (×10^3^) (M^–1^ cm^–1^)	quantum yield^c^	Ε_g_^opt^^d^ (eV)	HOMO^CV^^e^ (eV)	LUMO^UV^^f^ (eV)
			sol.^b^ (nm)	film (nm)	sol.^b^ (nm)	film (nm)					
*p*PACP	16/2.2	19	418	428	444	500	18.4	28	2.62		
P6A-CP	25/2.4	16	422	435	447	494	33.6	36	2.57	–5.37	–2.8
PnA-CP	11/1.8	20	421	420	338,478		28.7	8			
FA-CP	13/2.8	20	420	438	444	508	38.6	38	2.55	–5.06	–2.51
*p*PP6-CP	38/2.5	20	414	441	431	502	32.4	90			
A-HP	12/1.8		423	433	448	472,492	27.1	14	2.65	–5.38	–2.73
P6-HP	25/2.1		415	443	433	545	29.9	48	2.67	–5.35	–2.68

a Calculated from ^1^H NMR integration.

b In CHC1_3_.

c Relative quantum yield using perylene in ethanol as a reference.

d From the onset of thin-film UV–vis absorption spectra.

e From the oxidation peak onset in CV.

f LUMO^UV^ calculated from Ε_g_^opt^ – HOMO^CV^ values.

**Scheme 3:**
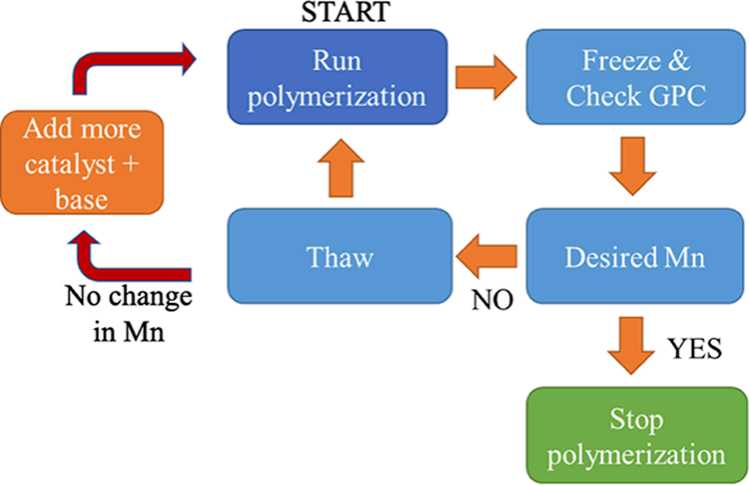
Freeze–Thaw–Run Approach Is Used to Synthesize Homo and Copolymers of Approximately Similar Molecular Weight

Generating copolymers of similar molecular weight eliminates the discrepancies that may arise due to molecular weight differences when comparing the copolymers’ properties. A freeze–thaw–run approach was utilized to generate copolymers of approximately similar molecular weights (Scheme 3). After taking an aliquot for molecular weight analysis, the polymer growth is temporarily seized by freezing the reaction mixture using liquid nitrogen. The polymerization mixture was thawed, quenched, and purified if the desired molecular weight was obtained. Otherwise, the polymerization mixture was thawed, bubbled with air for a couple of minutes, and allowed to polymerize further. In a few cases (see the Supporting Information, Table S1), wherein the molecular weight did not increase significantly after two freeze–thaw–run cycles, additional copper catalyst and/or ligand were added. This approach yielded copolymers with the number average molecular weight range from 13 to 15 kDa, except for polymers containing a dihexylphenyl monomer (Table 1). The copolymer molecular weights were determined using gel permeation chromatography (tetrahydrofuran as the eluent) against polystyrene standards. All the copolymers are soluble in chloroform and tetrahydrofuran.

**Figure 1:**
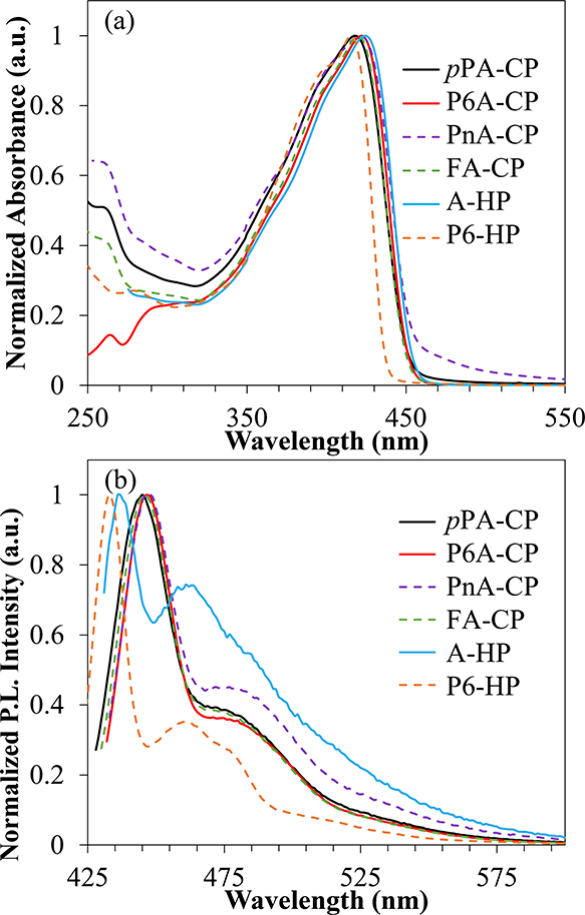
Normalized (a) UV–vis absorption spectra and (b) emission spectra of polymers in chloroform.

All the polymers contain a derivative of 1,4-phenyl as a nonstrapped comonomer except FA-CP, which has fluorene as a comonomer. To understand the impact of incorporating structurally diverse 1,4-phenyl aryl comonomer along the polymer backbone on optical properties, UV–vis absorption and emission spectra of the copolymers were recorded in chloroform and are shown in Figure 1. UV–vis absorption maximum of all the polymers is between 418 and 423 nm, indicating that the alkyl substituents used on the 1,4-phenyl aryl comonomers do not significantly alter the electronic nature of the copolymers (*p*PA-CP, P6A-CP, PnA-CP). The absorption maximum of FA-CP also differed by only a couple of nm compared to A-HP. Thus, the phenyl rings in fluorene behave like a conformationally locked biphenyl unit and do not behave like a fused acene. The spectral features of the copolymers and both the homopolymers (A-HP and P6-HP) are similar, indicating that the electronic nature of copolymers and homopolymers is optically the same. Emission spectra of all the copolymers are shown in Figure 1, and the emission maxima differ by only a few nm, indicating the length of the conjugated segment to which the exciton migrates to upon excitation is approximately the same in all the copolymers (Table 1). Interestingly, the emission intensities of the peaks are significantly different for a given concentration of the polymer solution, indicating that the quantum yields are different. The photoluminescence quantum yields (PLQYs) of the copolymers and homopolymers are determined and are shown in Table 1. The PLQY of all the copolymers is higher than the A-HP. UV–vis absorption and emission spectra of copolymers in thin films are shown in Figures S6 and S7.

**Figure 2:**
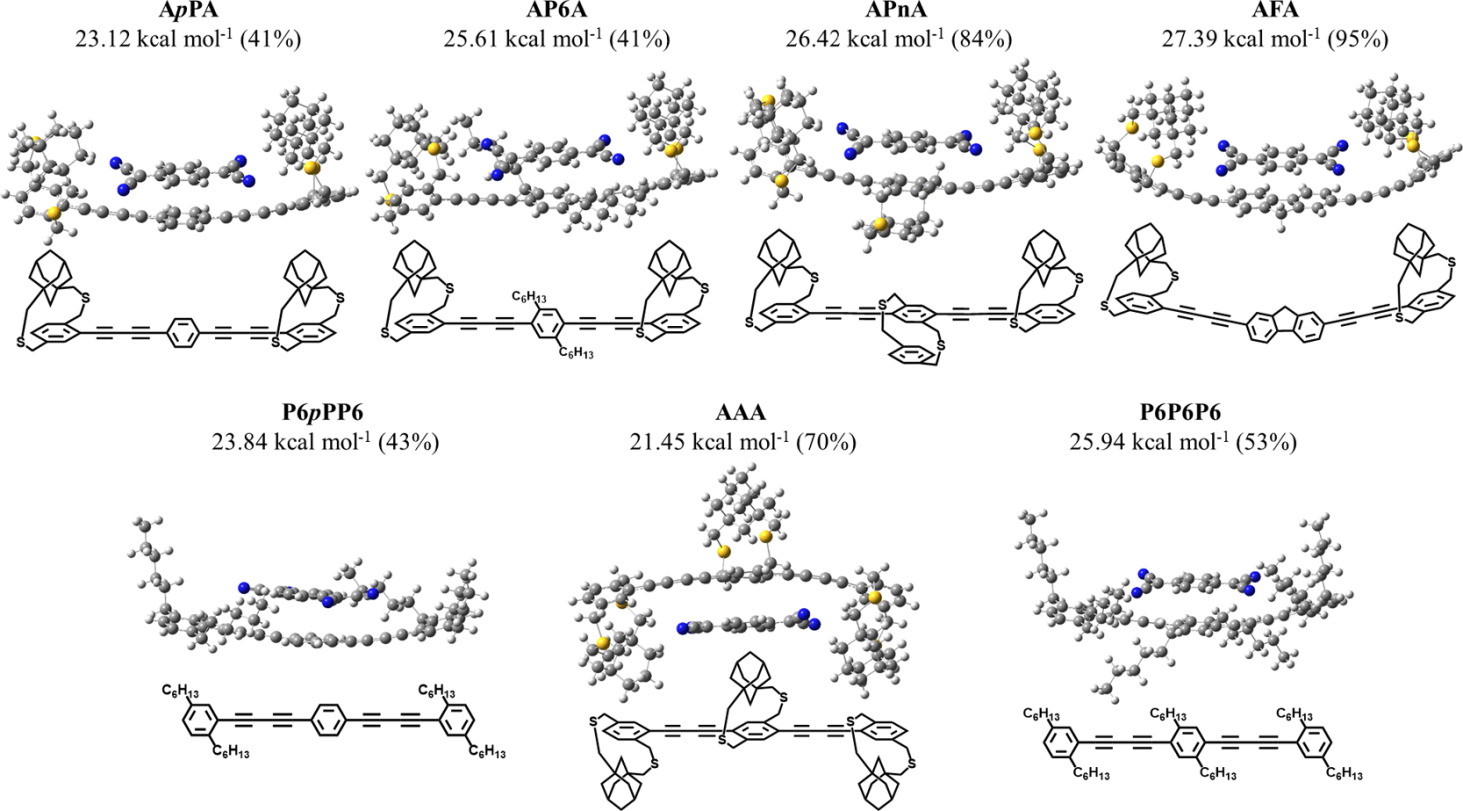
Configurations of most stable complexes of seven trimers with TCNQ determined using density functional theory (DFT)-B3LYP/6-311G** simulations and their binding energies and percent configurations (shown in the parenthesis). Line structures of the trimers are also shown for clarity.

The mixtures of strapped and nonstrapped aryl monomers are designed to direct the acceptor toward the nonstrapped unit and enhance the strength of acceptor interaction with the polymer. Structurally diverse nonstrapped aryl monomers are used to tune the polymer–acceptor interaction strength. Simulations (DFT-B3LYP/6-311G**) are performed on trimers corresponding to each copolymer to determine the location of the acceptor along the trimer as well as trimer-acceptor interaction strength. For simulations, trimers containing terminal adamantyl straps and a nonstrapped central repeat unit are used (Figure S48). Trimer-TCNQ complexes are optimized by placing the TCNQ on the strapped and nonstrapped units (see Supporting Information, Figures S49–S55). The presence of strapped repeat units on the trimer reduces the symmetry of the trimer and makes some of the TCNQ complexation locations on the trimer nondegenerate, increasing the number of possible donor–acceptor configurations. In addition, the orientation of the TCNQ on the trimer is also varied. Thus, overall ca. 69 trimer-TCNQ complex configurations are optimized using DFT-B3LYP/6-311G** calculations (see simulations supplementary information). The key optimized configurations and the corresponding binding energies for each trimer-TCNQ complex are determined and shown in the Supporting Information Figures S49–S55. The highest binding energy trimer-TCNQ configuration for each comonomer and homo-trimers is shown in Figure 2. The relative binding energy, i.e., the difference in the binding energy of a configuration compared to the most stable configuration, is also shown in Figures S49–S55. The Boltzmann factor is also calculated for each configuration and is used to determine the percentage of each configuration. Trimers containing a mixture of strapped and nonstrapped units showed higher binding energy than the completely strapped trimers, confirming that the nonstrapped aryl units enhance the interaction strength with acceptor. More importantly, in the trimers containing a mixture of strapped and nonstrapped repeat units, the TCNQ binds strongest to the nonstrapped aryl repeat unit, resulting in the highest percentage configuration (Table 2). Thus, TCNQ is directed toward the nonstrapped repeat units during the complex formation. Therefore, the complex simulations highlight that the nonstrapped comonomers direct the TCNQ toward the nonstrapped repeat unit and hence increase the interaction strength of the TCNQ with the polymer backbone. There is no clear trend in binding energy values with that of the frontier energy levels of the trimers and the band gap. The substituents on the comonomer play a role in stabilizing the complex since dihexylphenyl and dithia[3.3]paracyclophane units exhibit higher binding energy along with the fluorene, which contains a larger arene.

**Table 2:** Trimer-TCNQ Complex Binding Energies and Percentage Configuration Depending on the TCNQ Location on the Trimer^a^

trimer	HOMO (DFT studies) (eV)	LUMO (DFT studies) (eV)	band gap (DFT studies) (eV)	TCNQ in middle		TCNQ on terminal	
				BE^b^ (kcal mol^–1^)	% configuration	BE^b^ (kcal mol^–1^)	%configuration
A*p*PA	–5.83	–2.56	3.27	23.12 (41%)^c^	82	22.20 (17%)^c^	18
AP6A	–5.77	–2.46	3.31	25.61 (41%)^c^	99.50	19.27 (<0.1%)^c^	0.50
APnA	−5.83	–2.57	3.26	26.42 (84%)^c^	99	20.10 (0.1%)^c^	1
AFA	–5.67	–2.44	3.23	27.39 (95%)^c^	99	23.07 (0.3%)^c^	1
P6*p*PP6	–5.62	–2.34	3.28	23.84 (43%)^c^	54	23.45 (45%)^c^	46
AAA	–5.87	–2.62	3.25	21.45 (70%)^c^	98.5	17.54 (0.2%)^c^	1.5
P6P6P6	–5.56	–2.28	3.28	25.94 (53%)^c^	99	22.78 (0.3%)^c^	1

a DFT-B3LYP/6-311G**.

b BE (binding energy, BE = −(E_complex_ – E_donor_ – E_TCNQ_).

c % configuration of highest BE.

**Figure 3:**
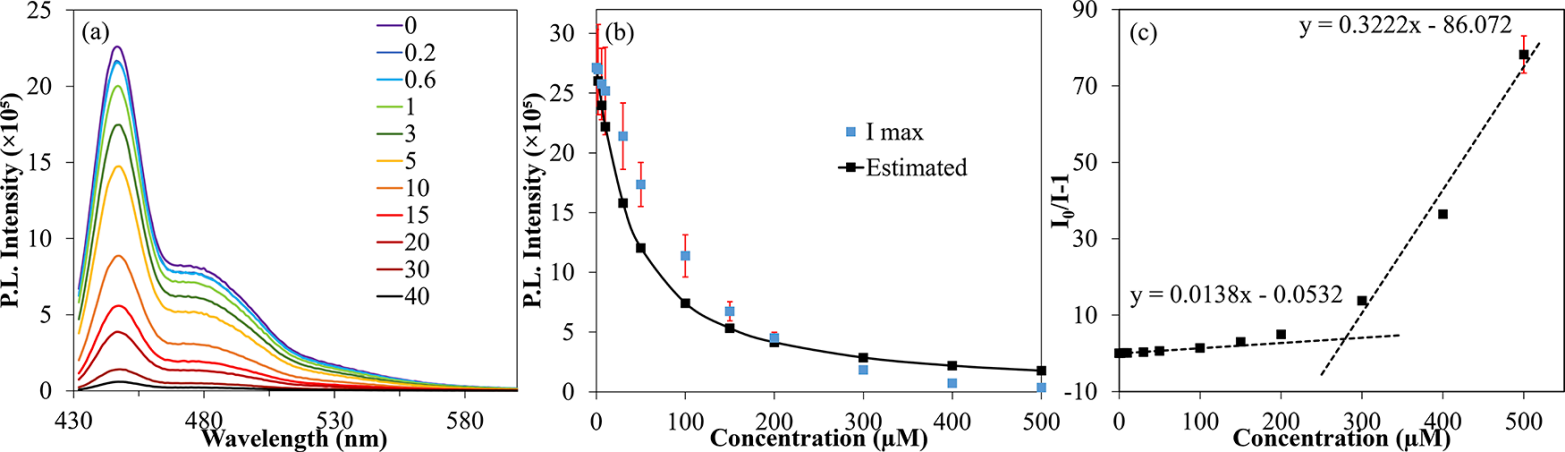
(a) TCNQ concentration-dependent fluorescence spectra of P6A-CP (legend: molar ratio of TCNQ to polymer repeat unit); (b) nonlinear fluorescence quenching data fit into a nonlinear Stern–Volmer equation to obtain *K*_SV_; and (c) fluorescence quenching data plotted using the linear Stern–Volmer equation and fit into two linear trend lines to obtain *K*_SV1_ (Stern–Volmer quenching constant in the low quencher concentration region) and *K*_SV2_ (Stern–Volmer quenching constant in the high quencher concentration region).

Strapped copolymers containing the mixture of strapped and nonstrapped aryl monomers are synthesized to enhance polymer interaction with the acceptor and facilitate electron transfer. PL quenching is a measure of electron transfer from the polymer to acceptor.^22,35−40^ Therefore, photoinduced PL quenching studies are conducted for the strapped copolymers in the presence of TCNQ to determine the Stern–Volmer (SV) quenching constant (*K*_SV_). The fluorescence intensity of all the copolymers reduces as the TCNQ concentration increases, and the reduction in intensity follows a concave-upward curvature of the SV plot (Figure 3b). In general, a nonlinear SV plot is due to simultaneous dynamic and static quenching.^45^ The fluorescence quenching data were fitted to a reported nonlinear SV equation^45^ to obtain the SV constant (*K*_SV_) (Figure 3, Table 3, and Figures S32–35). It is gratifying to see that the *K*_SV_ of all the copolymers is higher than that of the A-HP homopolymer. This indicates that incorporating nonstrapped comonomers along the insulated polymer backbone provides an easily accessible site for quenchers to interact and quench the fluorescence and increase the copolymers’ *K*_SV_.

**Table 3:** Nonlinear and Linear *K*_SV_ Values

polymer	nonlinear *K*_sv_ (×10^3^) (M^–1^)	linear *K*_sv_ (×10^3^)		*K*_SV2_/*K*_SV1_
		*K*_sv1_ (M^–1^)	*K*_sv2_ (M^–1^)	
*p*PA-CP	225 ± 20	28 ± 0.3	2092 ± 370	75
P6A-CP	30 ± 0.7	14 ± 1	335 ± 20	23
PnA-CP	44 ± 3	20 ± 0.1	246 ± 13	12
FA-CP	96 ± 0.5	19 ± 1.6	1360 ± 74	70
*p*PP6-CP	369 ± 7	242 ± 15	15716 ± 1600	65
A-HP	13 ± 1	8 ± 0.1	17 ± 13	2
P6-HP	110 ± 10	33	362	11

Previously, it has been shown that the strapped polymers’ fluorescence quenching efficiency follows the size of the steric shield, i.e., the smaller the diameter of the insulating sheath around the polymer backbone, the higher the *K*_SV_.^17^ The amount of insulating content around the nonstrapped *p*-phenylene-derivative comonomers reduces in the following order, A-HP > P6A-CP > PnA-CP > *p*PA-CP, while the *K*_SV_ increases (A-HP < P6A-CP < PnA-CP < *p*PA-CP) highlighting the importance of reducing the insulating content around the nonstrapped comonomers. The reduction in insulating content around the nonstrapped monomer allows the acceptor to approach the polymer backbone more closely and/or complex with the nonstrapped backbone.

**Scheme 4:**
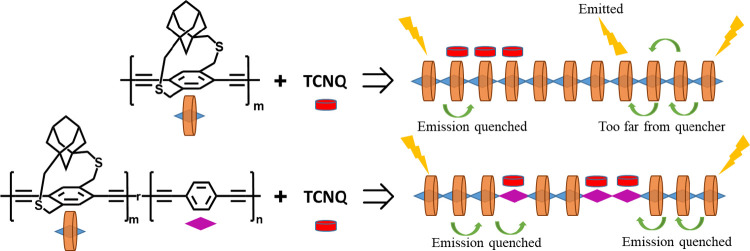
In Copolymers (Bottom) Containing a Mixture of Strapped and Nonstrapped Repeat Units, Acceptors Interact Strongly with the Nonstrapped Units Because the π-Face Is Not Blocked^*a*^ Since the nonstrapped units are randomly distributed along the polymer backbone in copolymers, it enhances the probability of random distribution of acceptors along the backbone, which increases non-neighbor acceptor binding events and results in higher PL quenching compared to homopolymers.

More importantly, the *K*_SV_ of one of the strapped copolymers (*p*PA-CP) is higher than that of the conventional pendant chain polymer (P6-HP) having no strapped repeat units, even though *p*PA-CP contains only 20% of nonstrapped units (Scheme 4). Comparing the *K*_SV_ of *p*PA-CP and P6-HP highlights the importance of randomly distributing the binding sites along the polymer backbone to realize higher PL quenching. For this, the fluorescence quenching data were analyzed in the low and high quencher concentration range to obtain two *K*_SV_ values (*K*_SV1_ and *K*_SV2_) (Table 3). *K*_SV1_ captures the copolymer quenching behavior in the low quencher concentration region; the increase in *K*_SV1_ matches with the change in steric sheath volume around the nonstrapped comonomer. *K*_SV2_ captures the copolymer quenching behavior in the higher quencher concentration region. The *K*_SV2_ of two copolymers (*p*PA-CP and FA-CP) is higher than that of P6-HP, the conventional polymer with pendant solubilizing chains. The increase in the polymer fluorescence quenching at higher acceptor concentration can be attributed to the change in polymer conformation, which favors the complex formation, and/or to the sphere of action model. According to the sphere of action model, there is always an acceptor molecule near the polymer at higher acceptor concentration leading to rapid quenching of the polymer fluorescence at the moment it is excited.^46,47^ The ratio of *K*_SV2_ to *K*_SV1_ highlighted how sensitive the PL quenching is to the change in acceptor concentration. The *p*PA-CPs *K*_SV_ increases 75 times in the higher concentration range, whereas the increase is only 11 times for P6-HP. Since the nonstrapped 1,4-phenyl binding sites in the *p*PA-CP copolymer are randomly distributed throughout the polymer chain, as the acceptor concentration increases, more binding sites will be occupied, and each acceptor binding event will lead to the quenching of excitons and reduction in the polymer PL. On the other hand, in the case of P6-HP, acceptors have a high probability of binding on neighboring units as it is energetically more favorable due to the neighbor effect, as shown in the literature.^48^ Since most acceptors localize at a location along the polymer backbone, the binding of new acceptors near the same location does not significantly quench the polymer PL. Thus, P6-HP is less sensitive to acceptors in the higher concentration range than the *p*PA-CP. To investigate if the polymer’s electronic energy levels are responsible for the observed trend in *K*_SV_ values, the redox potentials of the polymers were determined using cyclic voltammetry. All the polymers showed reversible oxidation peaks except the FA-CP (Figures S43–47). The HOMO energy levels of copolymers were calculated from the onset of the oxidation peak in reference to the ferrocene oxidation potential. The LUMO energy levels are calculated by combining the HOMO value and optical band gap (Table 1) following the previous literature^49−55^ (note: a few research groups^56−59^ have shown that combining optical absorption data and electrochemical redox potentials is not the best way to determine frontier energy levels because both of these are different physical processes). The free energy difference for the electron transfer from polymer to the TCNQ was determined using the Rehm–Weller equation.^60^ The Δ*G*_ET_ values are within 40 meV, and the Δ*G*_ET_ for A-HP is higher than the copolymers indicating that Δ*G*_ET_ does not capture the trend in the observed *K*_SV_ values. Also, there is no correlation between the binding energy of trimer-TCNQ and that of copolymer *K*_SV_ values. The PL quenching is a combination of steric sheath around the repeat units and the polymer interaction strength with the acceptor. A comparison of *p*PA-CP and P6-HP molecular weights highlights that the *K*_SV_ of *p*PA-CP is underestimated because the molecular weight of *p*PA-CP is lower than that of the control P6-HP, and the Swager group has shown that the *K*_SV_ of polymers increases with the increase in the polymer molecular weight.^61,62^ Thus, a mixture of strapped and nonstrapped random copolymers is an effective strategy to enhance the interaction strength of the acceptor with the polymer and control the location of the acceptor along the polymer backbone.

**Figure 4:**
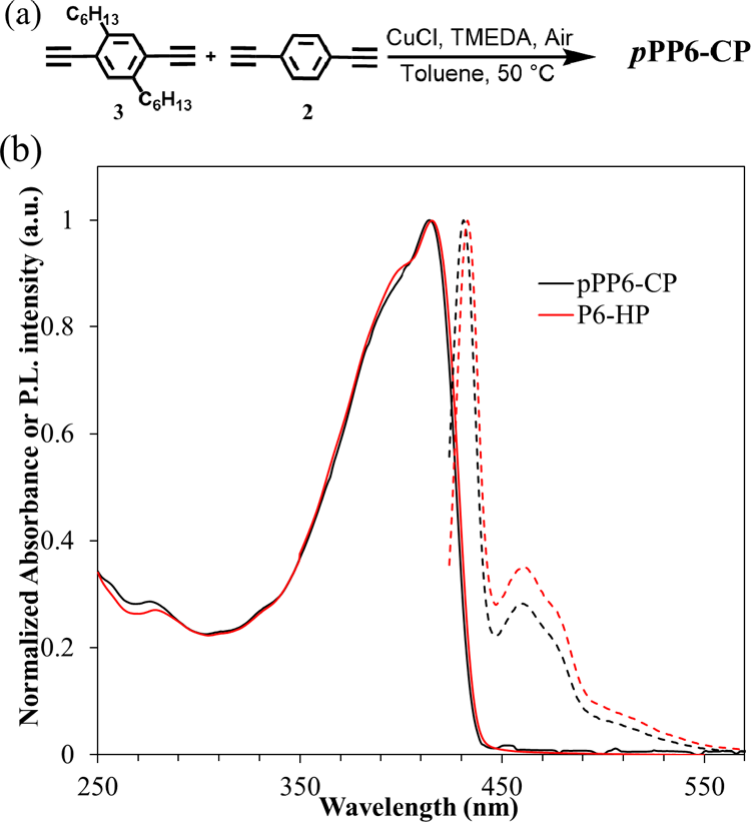
(a) Synthesis of the conventional pendant chain copolymer (*p*PP6-CP) containing an unsubstituted comonomer; (b) normalized UV–vis absorption spectra (solid lines) and fluorescence spectra (dashed lines) of copolymer *p*PP6-CP and pendant chain homopolymer P6-HP.

To determine if this strategy of mixing insulated and noninsulated monomers can be applied more broadly beyond strapped polymers to enhance the *K*_SV_ of conventional polymers with pendant solubilizing chains, a copolymer (*p*PP6-CP) containing dihexylphenyl and unsubstituted 1,4-phenyl is synthesized. *p*PP6-CP is synthesized following similar protocols discussed above, and the feed ratio of unsubstituted 1,4-phenyl is kept at 20 mole % (Figure 4). The percentage of incorporation of 1,4-phenyl in the copolymer is determined to be 20 mole % based on ^1^H NMR analysis. The UV–vis absorption and emission spectra features and maxima of copolymer *p*PP6-CP are similar to those of conventional pendant chain homopolymer (P6-HP), indicating that the 1,4-phenyl aryl comonomer does not significantly alter the electronic nature of the *p*PP6-CP compared to that of P6-HP (Figure 4b). For DFT simulations, a P6*p*PP6 trimer containing terminal dihexylphenyl and an unsubstituted 1,4-phenyl non-strapped central repeat unit was used for binding energy calculations. Simulations of various complex configurations of P6*p*PP6-TCNQ were simulated as discussed above (Figure S53). The binding energy of TCNQ with unsubstituted 1,4-phenyl of the P6*p*PP6 trimer is less favorable than that of binding to the dihexylphenyl unit. Nonetheless, the nonlinear *K*_SV_ of copolymer *p*PP6-CP is 3 times higher than that of homopolymer P6-HP. A comparison of *K*_SV1_ and *K*_SV2_ highlights the efficacy of incorporating unsubstituted 1,4-phenyls randomly along the conventional polymer backbone to realize higher PL quenching. The *K*_SV1_ of *p*PP6-CP is 7 times that of P6-HP due to a lower insulating content around the unsubstituted 1,4-phenyls. The *K*_SV2_ of *p*PP6-CP is 43 times higher than that of P6-HP. In addition, the *p*PP6-CP *K*_SV_ increases 65 times in the higher concentration range, whereas the increase is only 11 times for P6-HP. Both of these highlight the higher sensitivity of *p*PP6-CP to the PL quenching in the higher acceptor concentration region. Thus, in *p*PP6-CP, the binding sites are randomly spread along the polymer backbone, which helps to spread the acceptor location along the polymer backbone leading to higher exciton (PL) quenching. Thus, based on the simulations and PL quenching studies, copolymers containing a mixture of insulating pendant chains and unsubstituted monomers do not control the location of acceptor along the polymer backbone but enhance the polymers *K*_SV_.

## Conclusions

To summarize, we have shown that including a mixture of strapped and nonstrapped units along the polymer backbone is an effective strategy to enhance the polymers’ PL quenching efficiency, which is a measure of charge transfer from the polymer to acceptor. This strategy is applicable to not only strapped polymers but also to the conventional pendant chain polymers and enhances the polymers’ PL quenching efficiency. The strapped copolymers also control the acceptor location along the polymer backbone and tune polymer–acceptor interaction strength, which is an additional advantage over conventional pendant chain polymers. The nonstrapped comonomers also act as acceptor binding sites along the polymer backbone, and the acceptors can be directed toward them along the polymer backbone. The increase in PL quenching and higher *K*_SV_ is attributed to the absence of steric sheath around the comonomers along with their random location along the polymer backbone, which enhances the probability of non-neighbor acceptor binding events along the polymer backbone compared to homopolymers. Controlling the density, location, and strength of the charge transfer complexes is key for organic electronics, including solar cells and thermoelectrics. This work shows that designing conjugated polymers with a combination of insulated and noninsulated monomers will help us to develop materials that can help us systematically study the impact of these parameters on charge generation, charge transport, and device performance.

## Supporting Information Available

General information; general reaction scheme for the synthesis of non-strapped monomers and polymers; gel permeation chromatography (GPC) traces of polymers; normalized thin film UV-vis and emission spectra; molar extinction coefficient determination plots; quantum yield calculation; fluorescence quenching; non-linear Stern–Volmer plots of copolymers; linear Stern–Volmer plots; cyclic voltammograms; computational methods; ^1^H NMR spectra (PDF)
TCNQ coordinates; A*p*PA-TCNQ coordinates; AP6A-TCNQ coordinates; APnA-TCNQ coordinates; AFA-TCNQ coordinates; P6*p*PP6-TCNQ coordinates; AAA-TCNQ coordinates; P6P6P6-TCNQ coordinates (PDF)
