# Supplementary material for: Controlling π-Conjugated Polymer–Acceptor Interactions by Designing Polymers with a Mixture of π-Face Strapped and Nonstrapped Monomers
Source: Macromolecules. 2023 Apr 25;56(9):3421–9. doi: 10.1021/acs.macromol.3c00175 (PMC10950295; doi:10.1021/acs.macromol.3c00175)
Supplement: Supplementary file 1 — ma3c00175_si_001.pdf [file ma3c00175_si_001.pdf]

Supporting Information

Controlling π-Conjugated Polymer–Acceptor Interactions by Designing Polymers with a Mixture of π-Face Strapped and Non-Strapped Monomers

Fatima Hameed,[a,b] Manikandan Mohanan,[a,b] Nafisa Ibrahim[c], Charles Ochonma[a,b], Joaquín Rodríguez-López[c] and Nagarjuna Gavvalapalli[a,b]*

[a]Department of Chemistry, [b]Institute for Soft Matter Synthesis and Metrology, Georgetown University, Washington, D.C., 20057, USA, [c]Department of Chemistry, University of Illinois Urbana-Champaign, Urbana, IL 61801, USA

* Corresponding author: N.G. (email: ng554@georgetown.edu)

1.0 General information: S2
2.0 General reaction scheme for the synthesis of non-strapped monomers and Polymers ..S4
3.0 Gel Permeation Chromatography (GPC) Traces of Polymers.......................................S6
4.0 Normalized Thin film UV-Vis and emission spectra...S9
5.0 Molar Extinction Coefficient Determination Plots S10
6.0 Quantum yield calculation..S14
7.0 Fluorescence quenching..S22
8.0 Non-linear Stern-Volmer Plots of Copolymers S24
9.0 Linear Stern-Volmer plots..S26
10.0 Cyclic Voltammograms:...S29
11.0 Computational Methods: S32
12.0 ^{1}H NMR Spectra..S41

1.0 **General information:**

Reactions were performed in oven-dried glassware fitted with rubber septa and were stirred with Teflon-coated magnetic stirring bars. 1H-NMR spectra were recorded on Varian 400 NMR. Chemical shifts are reported in δ (ppm) relative to the residual solvent peak CD_2Cl_2: 5.3, $CDCl_3$: 7.27. Coupling constants (J) are expressed in Hertz (Hz). Splitting patterns are designated as s(singlet), br(broad signal), d(doublet), t(triplet), dd(doublet of doublets), dt(doublet of triplets), dq(doublet of quartets), m(multiplet), and q(quartet). NMR impurities include hexanes and diethyl ether. UV-vis absorption spectra were recorded on Agilent Technologies Cary Series 5000 UV-vis-NIR Spectrophotometer. Fluorescence absorption spectra were recorded on Horiba Scientific Fluoromax-4 Spectrophotometer. Cyclic voltammetry experiments were run using PGZ402 and data was analyzed by Voltamaster 4.

Cyclic voltammetry experiments were conducted as follows: A three-electrode cell was used, using a platinum disk (3 mm) working electrode and a platinum wire counter electrode. Silver/silver ion (0.01 M $AgNO_3$, 0.1M $LiBF_4$ solution in Acetonitrile) was used as a reference electrode. Thin film cyclic voltammetry was performed using 0.1 M $LiBF_4$ solution in acetonitrile as supporting electrolyte using a scan rate of 100 mVs^{-1}. Polymer solutions were prepared using 10 mg/mL concentration and 50 µL volume was used to make the film. Films were prepared by drop-coating the electrode and dried under vacuum for 1h. All solutions and cells were prepared under a nitrogen atmosphere in a glove box. The working electrode was polished with 0.05 µm alumina polish prior to each scan. All oxidation potentials are reported with respect to $E_{1/2}$ of the Fc/Fc^+ redox couple. The energy level of the Fc/Fc^+ was presumed at -4.8 eV at vacuum. HOMO values were calculated using the formula:

$$E_{HOMO} = -[4.8 - E_{\frac{1}{2},FC,FC^+} + E_{Ox,\,onset}]$$

Where $E_{Ox,\,onset}$ is the onset of oxidation and $E_{\frac{1}{2},FC,FC^+}$ is the half-wave potential of the ferrocene reference. HOMO values are determined from the onset of the second CV trace (for all polymers except FA-CP) that is obtained when the potential was swept towards more positive potentials from the open circuit potential. For FA-CP the onset of the first CV trace was used as the oxidation peak was irreversible.

Bad gap (E_g^{opt}) was calculated using the onset of thin-film UV-Vis absorption spectra (λ_{onset}) using the formula: (E_g^{opt}) = $1240/\lambda_{onset}$. $LUMO^{UV}$ was calculated from E_g^{opt} - $HOMO^{CV}$.[1-7]

Quantum yield *(QY_x)* of the polymers were determined as follows: Dilute polymer solutions were prepared such that their absorbance falls below 0.1 a.u. Emission spectra were measured by exciting at its corresponding absorbance maxima. The emission spectra were integrated and plotted against their corresponding absorbances to obtain the slope *($Slope_X$)* of emission integration v/s absorption for the sample. Perylene in ethanol was used as the standard. Emission spectra of perylene was obtained by exciting at the absorbance maxima of the polymers under study. Thus, the slope of emission integration v/s absorption for the standard *($Slope_{ST}$)* was also obtained. The obtained values were plugged into the following equation to obtain QY_x where *RI* is the refractive index of solvents used and QY_{ST} is the reported QY of perylene in ethanol.[8]

$$QY_X = QY_{ST}(Slope_X/\,Slope_{ST})(RI^2{}_X/RI^2{}_{ST})$$

Stern Volmer Quenching constant (K_{sv}) of the polymers were determined as follows: Polymer stock solutions of known concentrations (using repeat unit molecular weight) were prepared in chloroform. Similarly, TCNQ solution in chloroform was also prepared. Samples of different molar equivalents of quencher but constant polymer concentration (5 uM for *p*PP6-CP and 10 uM for all others) were prepared by mixing required amount of TCNQ solution, polymer solution and chloroform. Polymers were excited at their absorption maxima and 1 nm slit width was used for both excitation and emission. Linear and non-linear Ksv values were determined using literature reported formulas.[9]

Linear K_{SV} formula:

$$\frac{I_0}{I} = 1 + K[Q]$$

where I_0 is defined as the fluorescence intensity in the absence of quencher, [Q] is the quencher concentration at any given time, and I is the fluorescence intensity at [Q]

Non-linear K_{SV} formula:

$$I = I_0 - \frac{c}{2}\left(\frac{1}{K} + [F]_0 + [Q]_0 \quad - \sqrt{\left(\frac{1}{K} + [F]_0 + [Q]_0\right)^2 - 4[F]_0[Q]_0}\right)$$

where K is the association constant, I_0 is defined as the fluorescence intensity in the absence of quencher, $[Q]_0$ is the initial quencher concentration, I is the fluorescence intensity at [Q], [Q] is the quencher concentration at any given time, $[F]_0$ is initial fluorophore concentration, c is proportionality constant, where c is equal to I_0 divided by $[F]_0$ as measured experimentally.

2.0 **General reaction scheme for the synthesis of non-strapped monomers and Polymers**

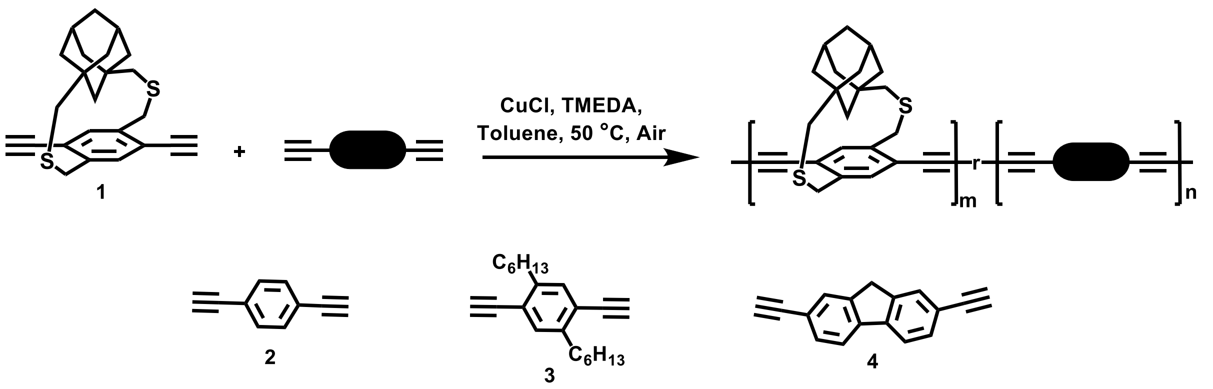

Synthesis of compound 1 was reported by us previously.[10] Compounds 2-4 were prepared by the reported procedures.[11-13]

General Procedure for Glaser-Hay Polymerization

Cyclophane diacetylene (±1) (0.8 eq., 300 mg) and subsequent diacetylene monomer (2-4) (0.2 eq.) were dissolved in toluene (1 mL toluene per 10 mg of diacetylene) and copper (I) chloride (0.8 eq.) was added. The reaction flask was bubbled with air for 10 min. N,N,N',N'-tetramethylethylenediamine (0.8 eq.) was added and the reaction mixture was stirred at 50 ˚C. Freeze-thaw method was used to seize the polymerization by immersing the reaction flask in liquid nitrogen and placing it in the freezer. An aliquot was taken to check the GPC /Mn at that particular time. If there was little to no change in Mn after restarting and running the reaction, extra equivalents of ligand and catalyst were added, as well purging with air. When the desired Mn was achieved, the reaction mixture was precipitated in methanol and filtered. The solid was washed with methanol and diethyl ether. The crude polymer was purified by Soxhlet with methanol for 18 hours and then with chloroform for 36 to 48 h. Polymerization details including the number and subsequent times of the freeze/thaw cycles as well as Mn/Mw and PDI details are shown in the Table S1:

Glaser Hay- polymerization of *p*PP6-CP:

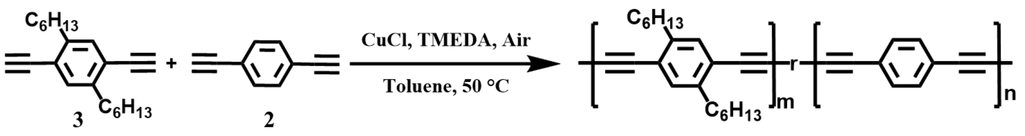

3 (0.8 eq.) and **2** (0.2 eq.) was polymerized using the same polymerization method as mentioned above. Polymerization details including the number and subsequent times of the freeze/thaw cycles as well as Mn/Mw and PDI details are shown in the Table S1:

Table S1:

Polymers	Time	Mn (kDa)	Mw (kDa)	PDI
*p*PA-CP	40 min	23	77	3.3
	After Soxhlet	15	35	2.2
P6A-CP	32 min	13	29	2.3
	45 min	13	29	2.3
	60 min (Air, TMEDA 0.5 eq)	13	33	2.3
	80 min (CuCl 1 eq., Air, TMEDA 1 eq.)	24	60	2.5
	After Soxhlet	25	61	2.4
FA-CP	35 min	17	39	2.2
	47 min	18	43	2.4
	After Soxhlet	13	38	2.8
*p*PP6-CP	2h	8	15	1.8
	3h (TMEDA 0.5 eq, Air)	27	57	1.9
	After Soxhlet	38	97	2.5
P6-HP	18h	17	35	2.0
	After Soxhlet	25	52	2.1

***p*PA-CP:** ^{1}H NMR (400 MHz, Methylene Chloride-d_2) δ 7.56 (t, J = 59.0 Hz, 3H), 4.05 (s, 2H), 3.43 (s, 2H), 2.70 (s, 2H), 2.21 – 1.89 (m, 4H), 1.69 (s, 1H), 1.45 (d, J = 21.9 Hz, 4H), 1.21 (s, 3H), 1.05 – 0.84 (m, 2H), -0.09, %) (d, J = 46.9 Hz, 2H). Yield: 40 mg (13%)

P6A-CP: ^{1}H NMR (400 MHz, Methylene Chloride-d_2) δ 7.70 (s, 1H), 7.41 (d, J = 11.8 Hz, 1H), 4.15 (d, J = 50.8 Hz, 2H), 3.59 (d, J = 18.2 Hz, 2H), 2.76 (d, J = 25.5 Hz, 2H), 2.19 – 2.01 (m, 1H), 1.93 (s, 2H), 1.58 (d, J = 82.2 Hz, 6H), 1.37 (d, J = 2.5 Hz, 4H), 0.90 (d, J = 5.7 Hz, 7H), 0.03 – -0.28 (m, 2H). Yield: 80 mg (30%)

FA-CP: ^{1}H NMR (400 MHz, Methylene Chloride-d_2) δ 7.87 – 7.29 (m, 3H), 4.14 (d, J = 63.3 Hz, 2H), 3.98 (s, 1H), 3.49 (s, 2H), 2.71 (s, 2H), 2.26 – 1.98 (m, 1H), 1.90 (d, J = 23.9 Hz, 2H), 1.48 (s, 2H), 1.24 (d, J = 29.3 Hz, 5H), 1.05 – 0.81 (m, 3H), -0.09 (d, J = 46.0 Hz, 2H). Yield: 20 mg (6%)

***p*PP6-CP:** ^{1}H NMR (400 MHz, Chloroform-d) δ 7.51 (s, 1H), 7.37 (s, 2H), 2.76 (d, J = 11.8 Hz, 4H), 1.73 – 1.60 (m, 4H), 1.34 (d, J = 8.5 Hz, 12H), 0.90 (d, J = 6.7 Hz, 7H). Yield: 200 mg (66%)

3.0 **Gel Permeation Chromatography (GPC) Traces of Polymers**

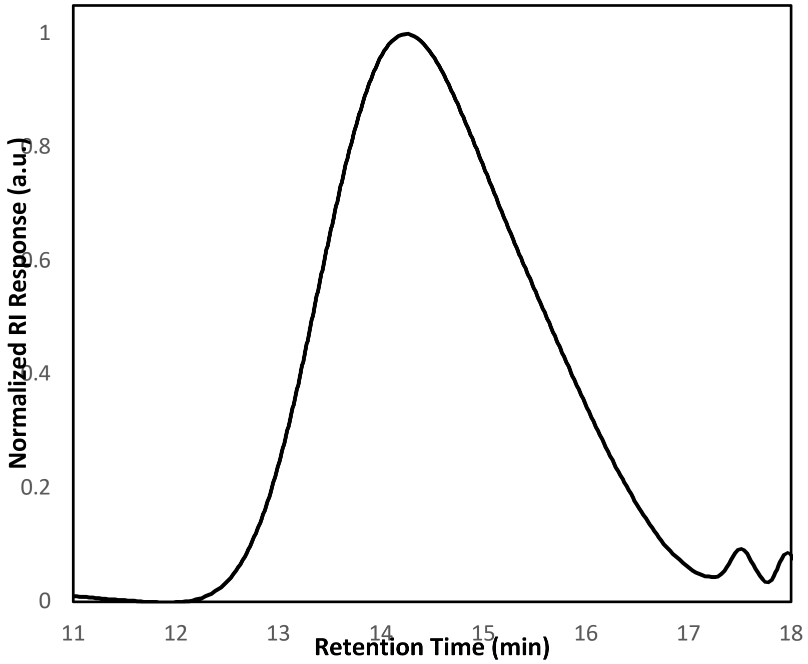

Figure S1: *p*PA-CP

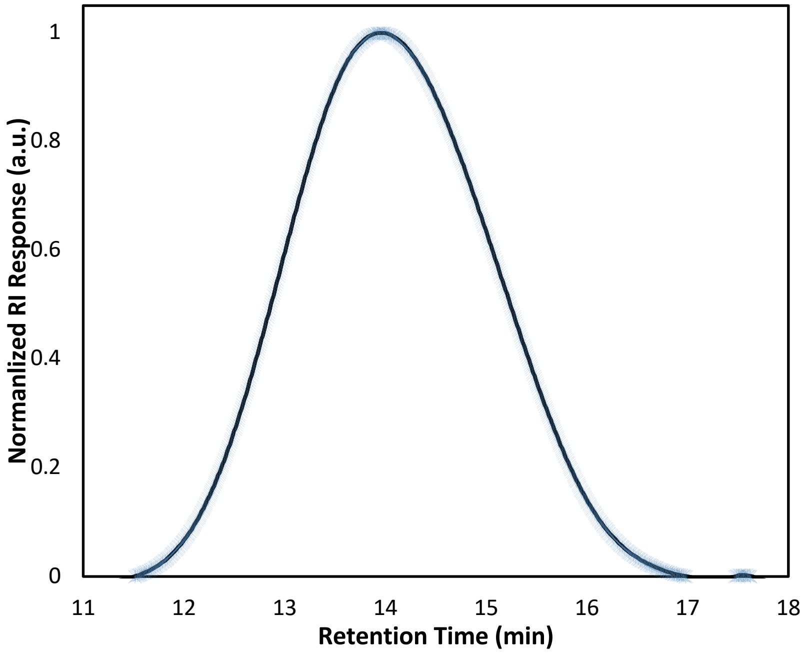

Figure S2: P6A-CP

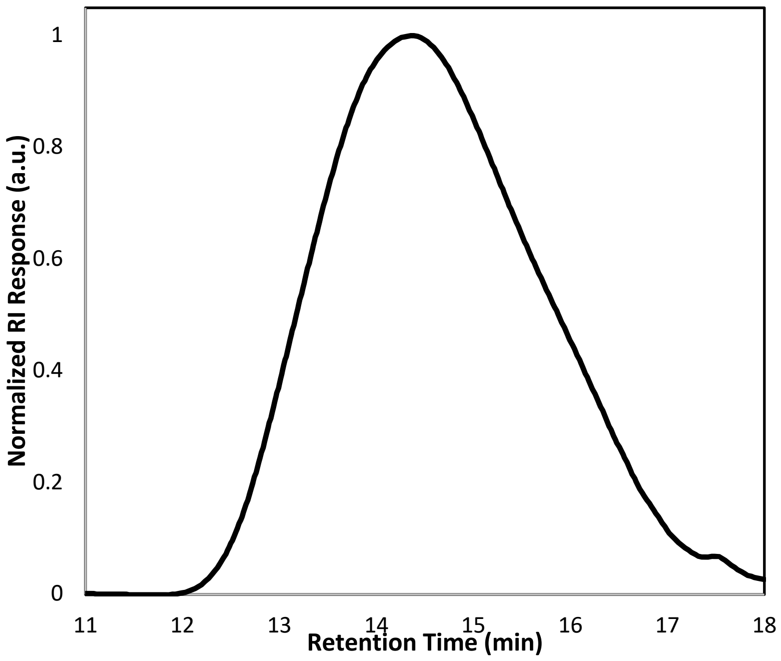

Figure S3: FA-CP

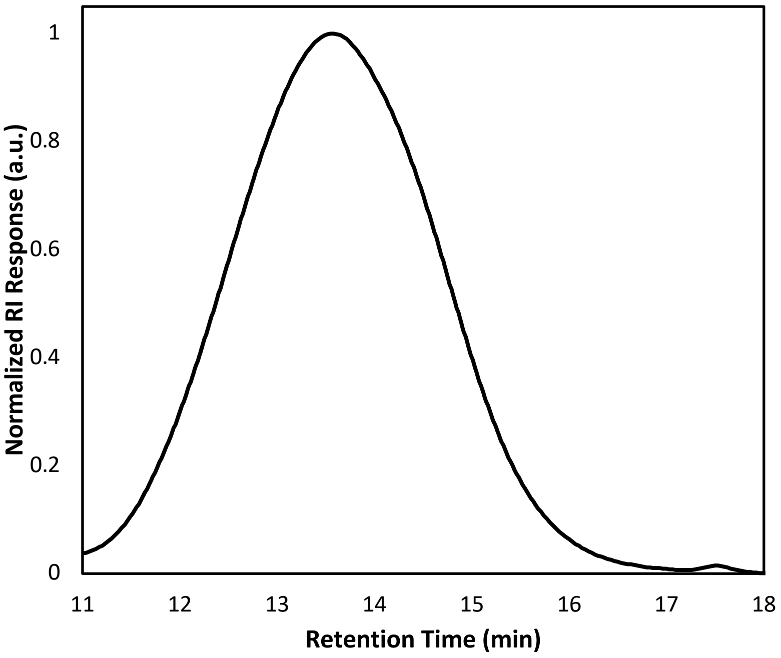

Figure S4: *p*PP6-CP

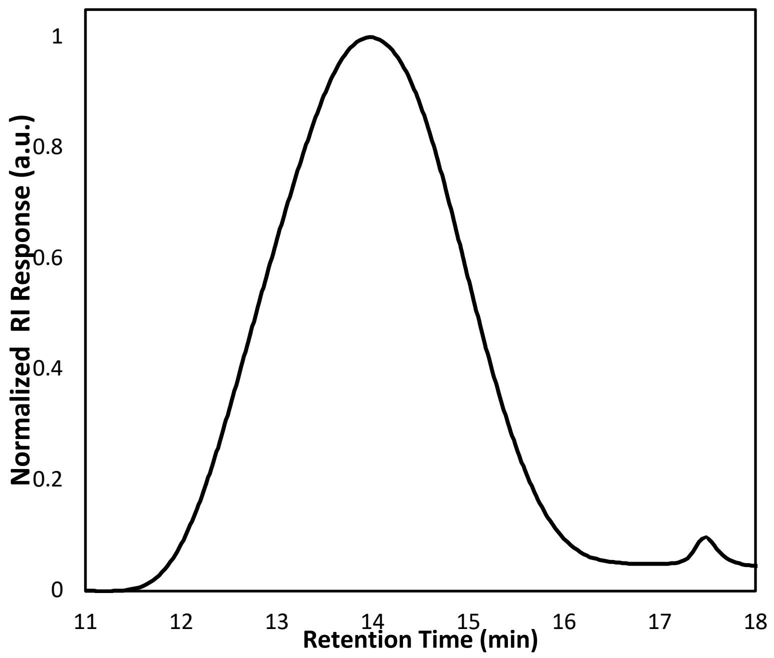

Figure S5: P6-HP

4.0 **Normalized Thin film UV-Vis and emission spectra**

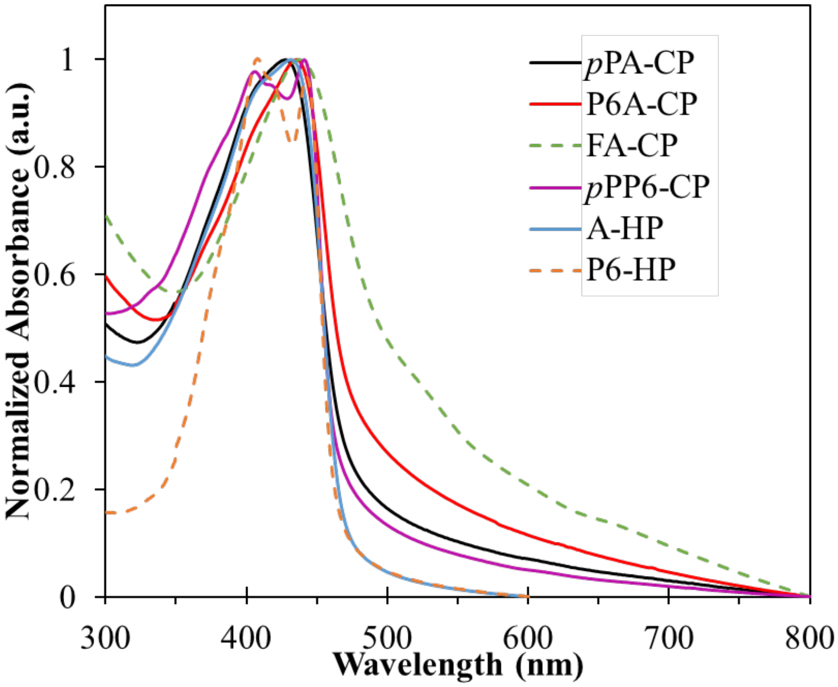

Figure S6: Normalized thin film UV-Vis absorption spectra

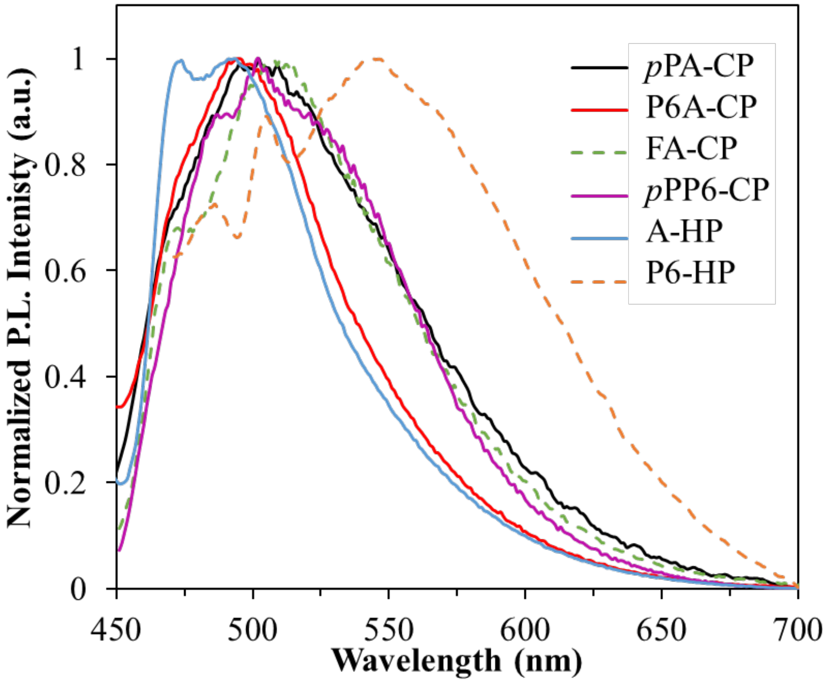

Figure S7: Normalized thin film emission spectra

5.0 **Molar Extinction Coefficient Determination Plots**

Concentration dependent UV-Vis absorption spectra of *p*PA-CP

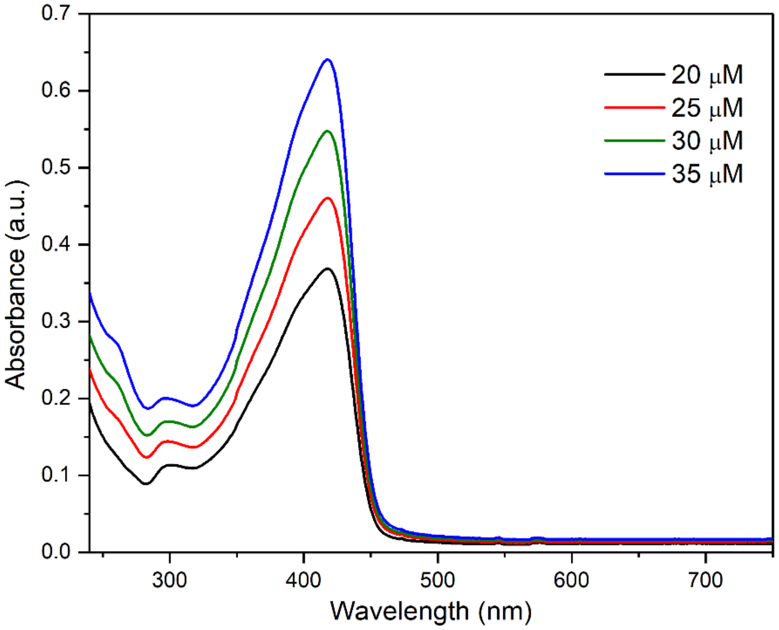

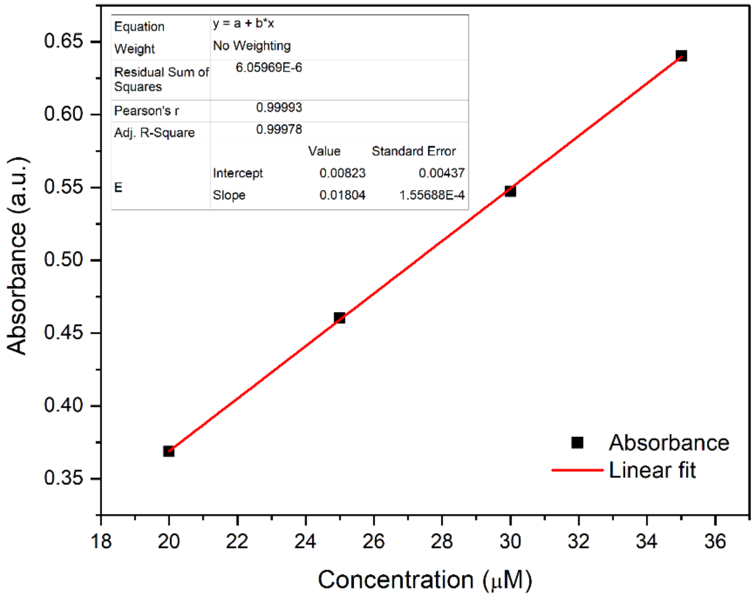

Figure S8: *p*PA-CP

Concentration dependent UV-Vis absorption spectra of P6A-CP

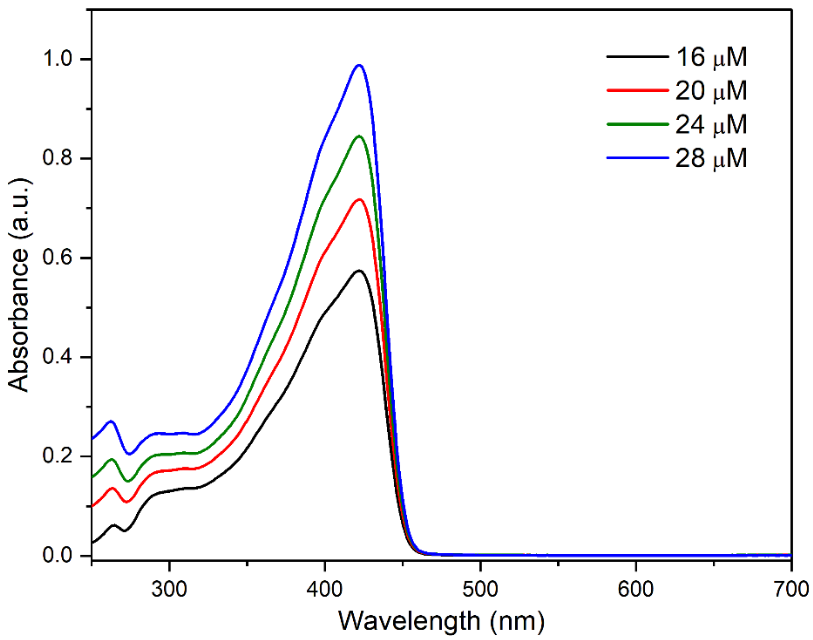

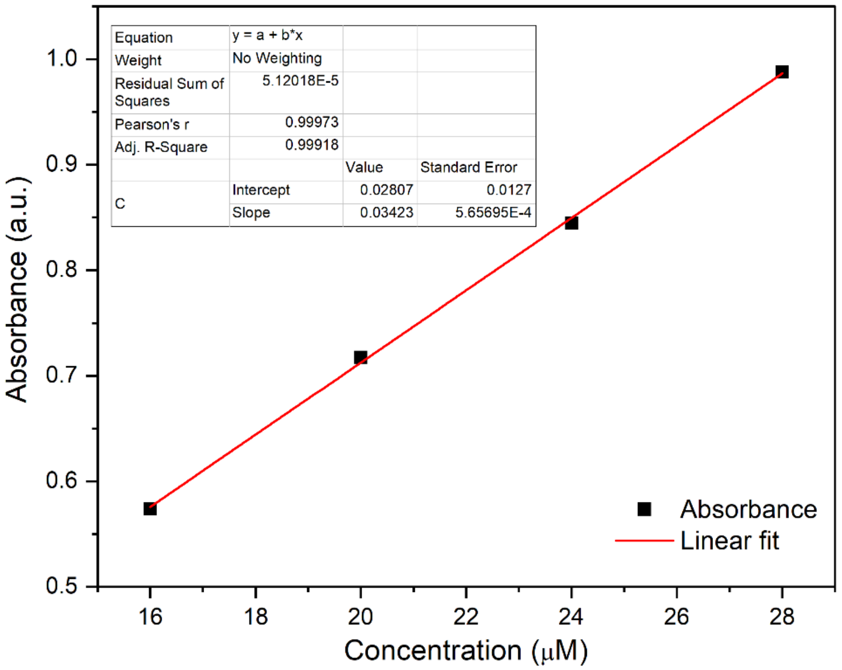

Figure S9: P6A-CP

Concentration dependent UV-Vis absorption spectra of FA-CP

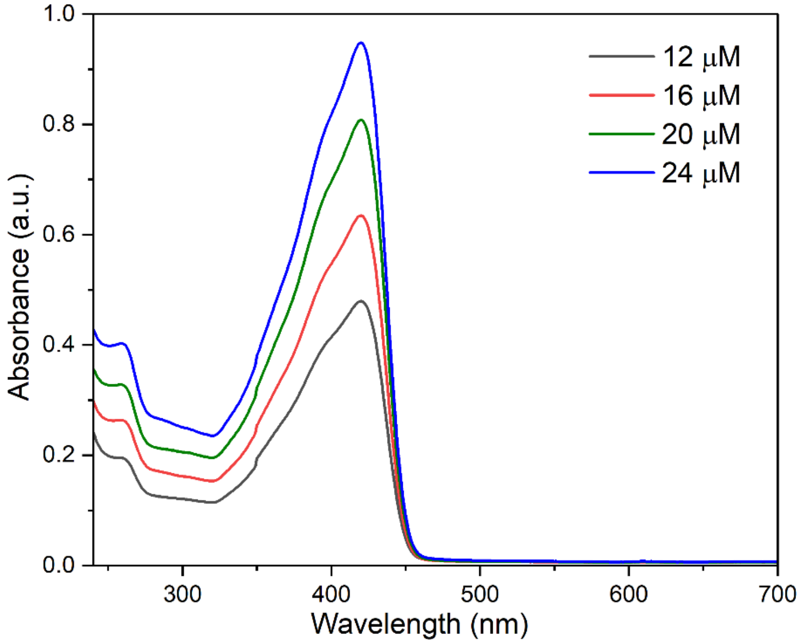

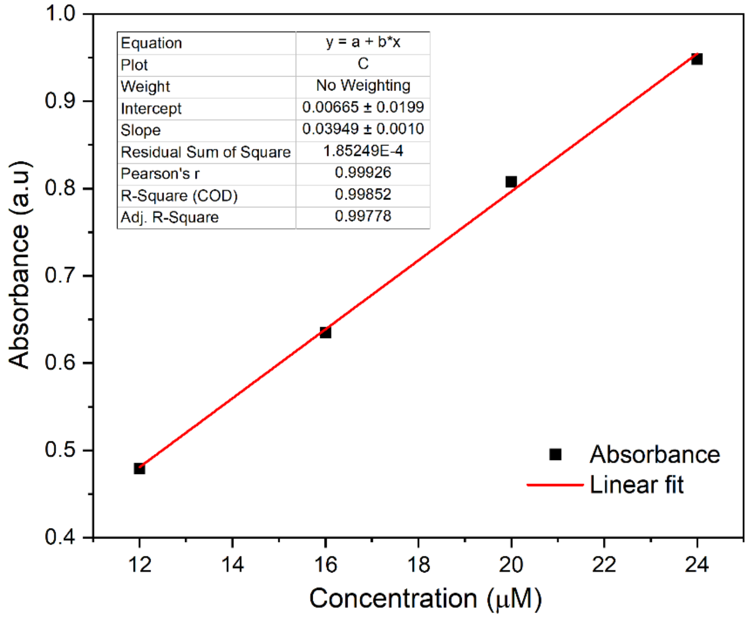

Figure S10: FA-CP

Concentration dependent UV-Vis absorption spectra of *p*PP6-CP

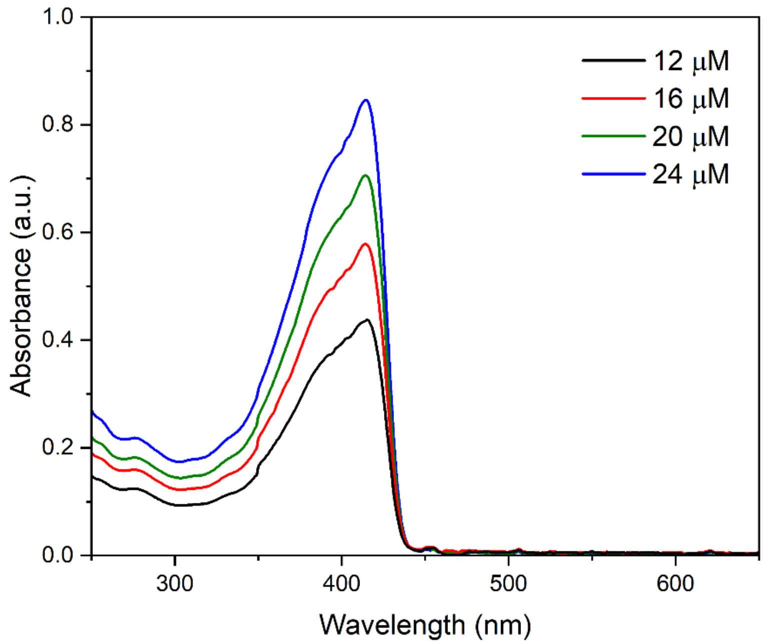

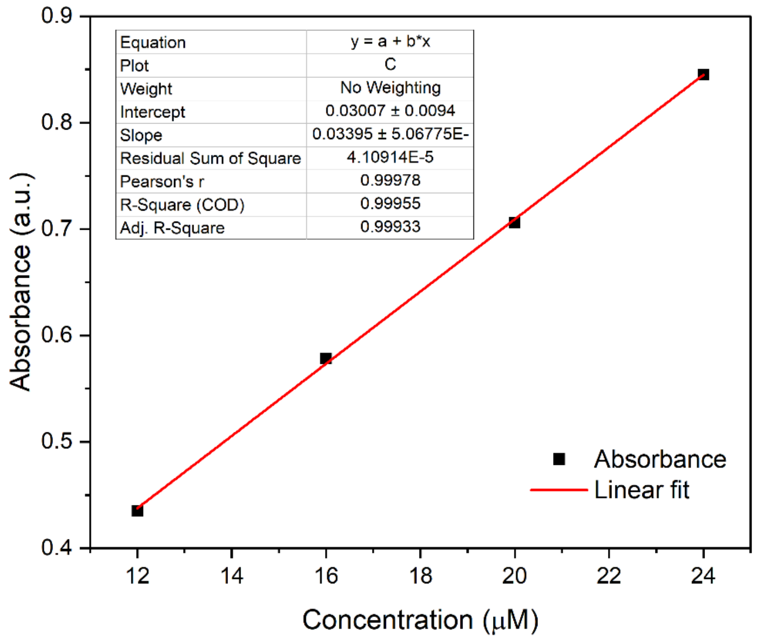

Figure S11: *p*PP6-CP

6.0 **Quantum yield calculation**

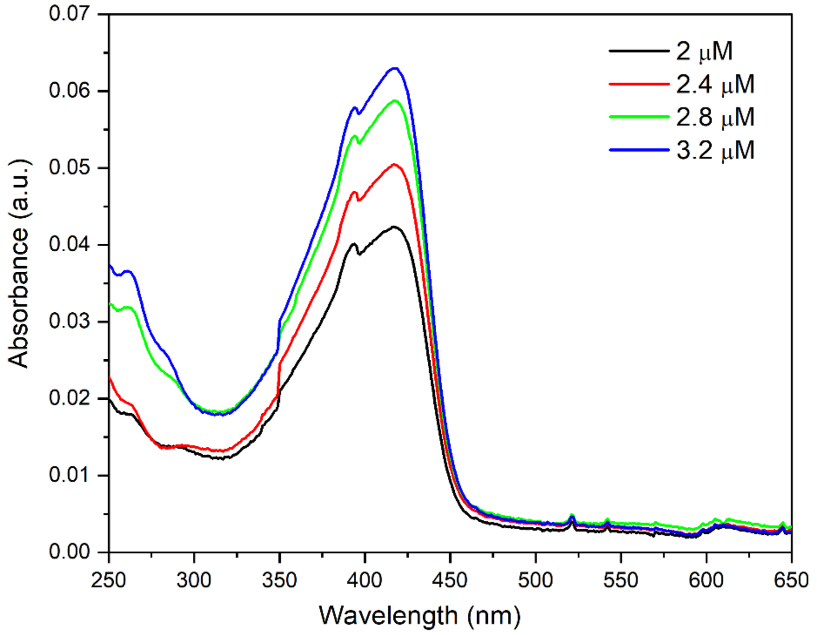

Figure S12: Concentration dependent UV-Vis absorption spectra of *p*PA-CP

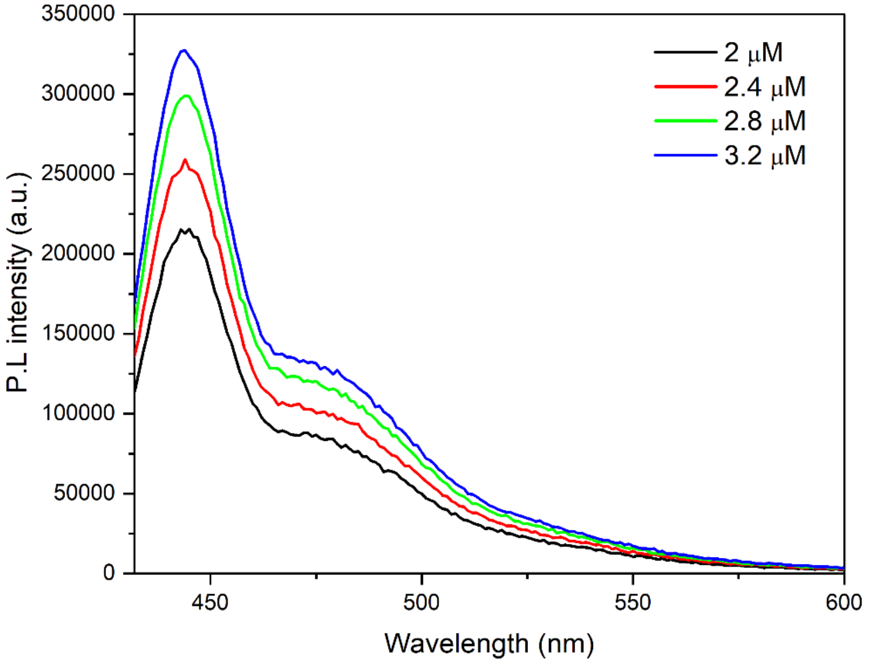

Figure S13*:* Concentration dependent emission spectra of *p*PA-*CP*

Plots used for Quantum Yield calculations

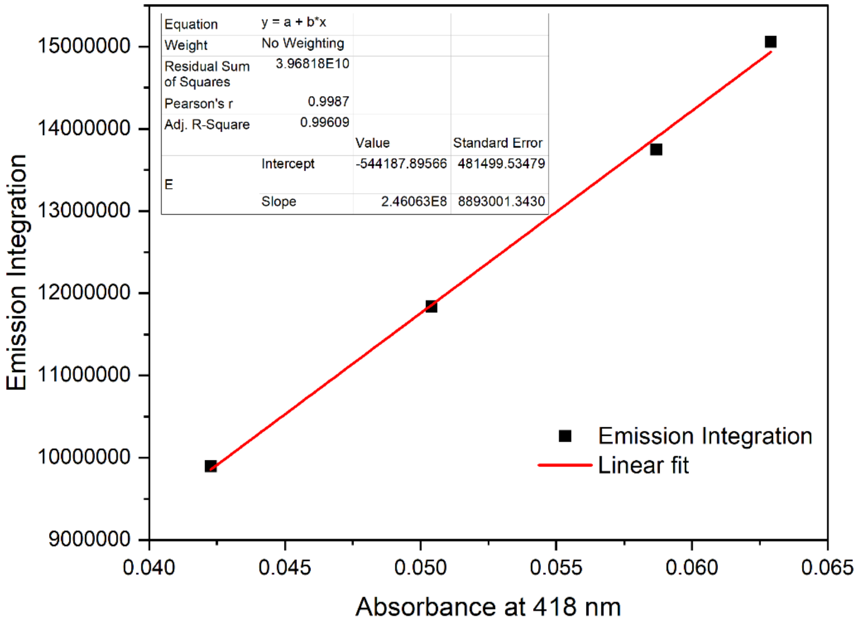

Figure S14: *p*PA-CP

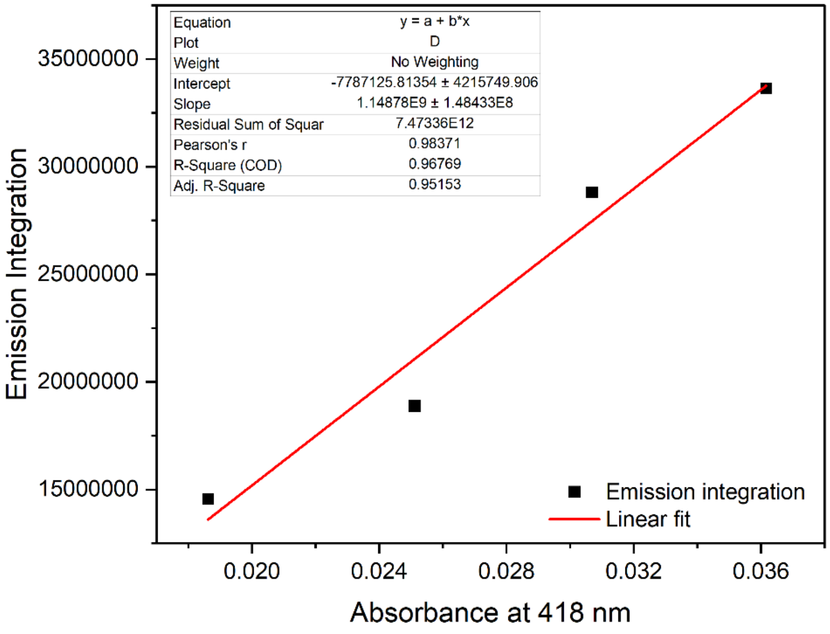

Figure S15: Perylene (reference)

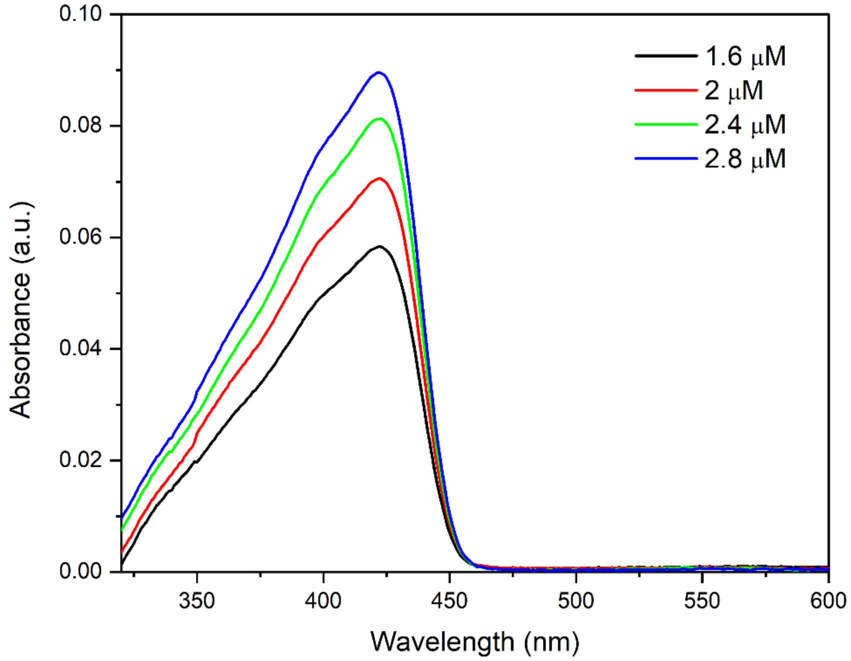

Figure S16: Concentration dependent UV-Vis absorption spectra of P6A-CP

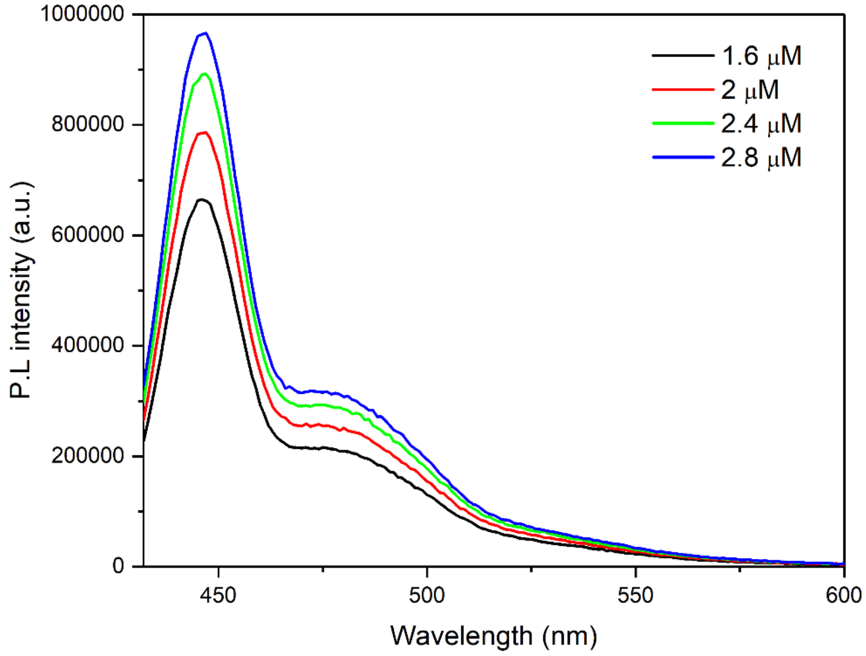

Figure S17: Concentration dependent emission spectra of P6A-CP

Plots used for Quantum Yield calculations

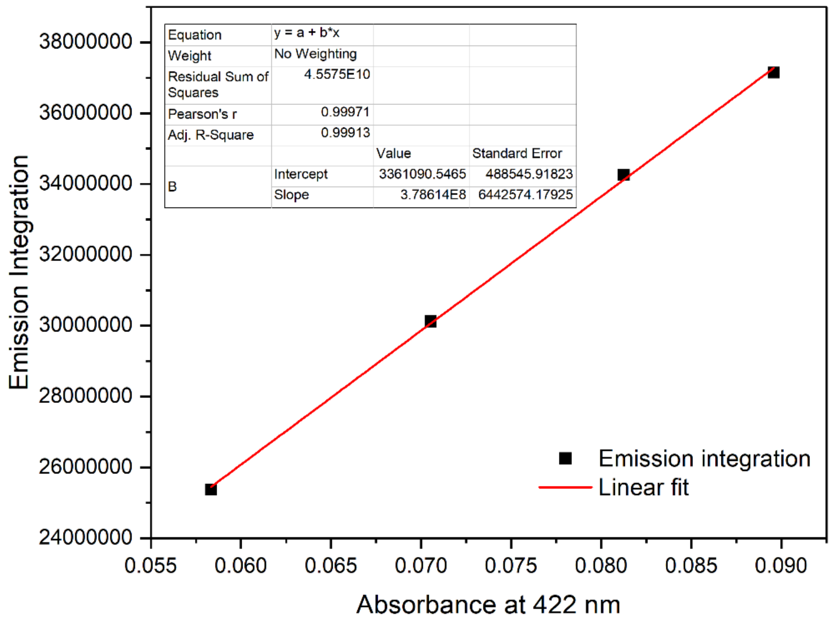

Figure S18: P6A-CP

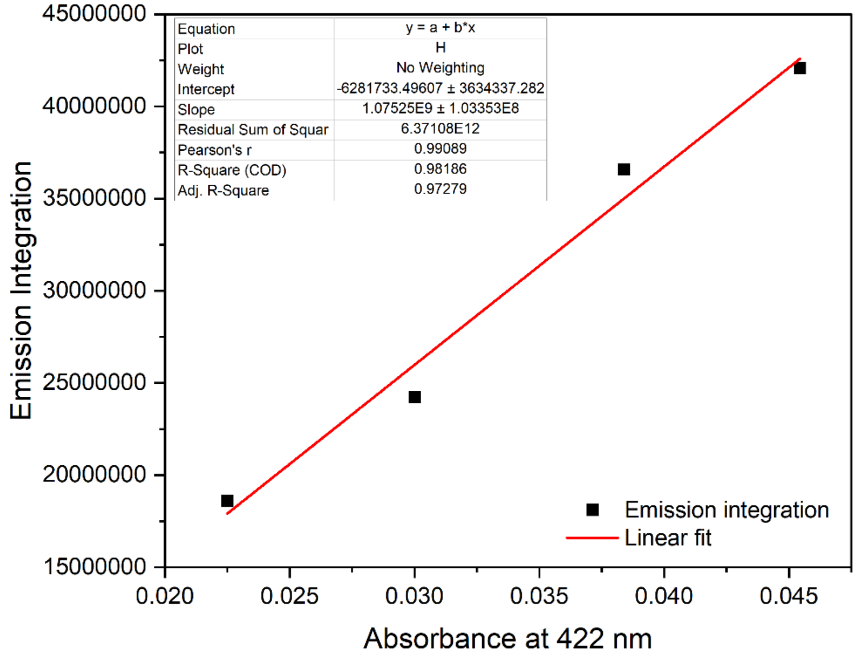

Figure S19: Perylene (reference)

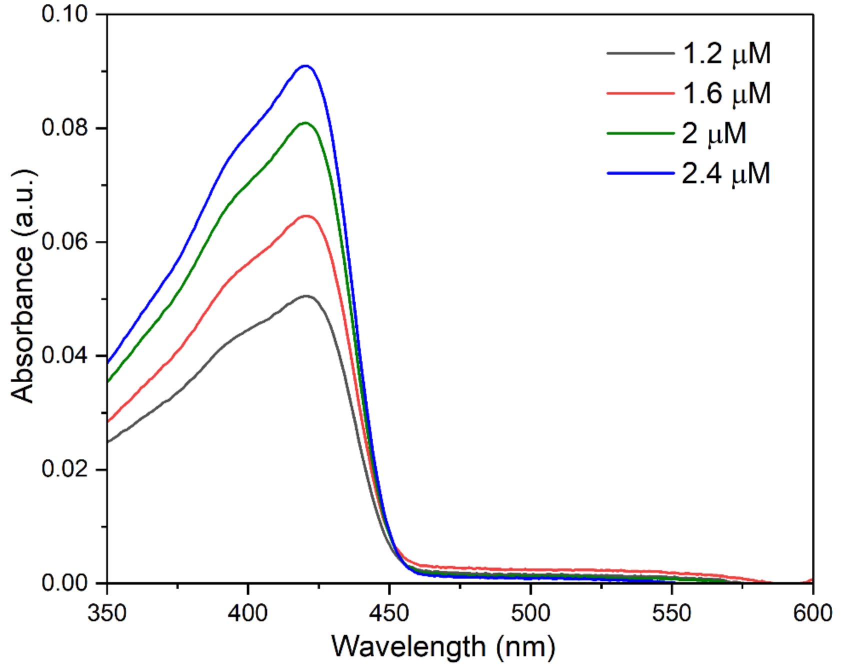

Figure S20: Concentration dependent UV-Vis absorption spectra of FA-CP

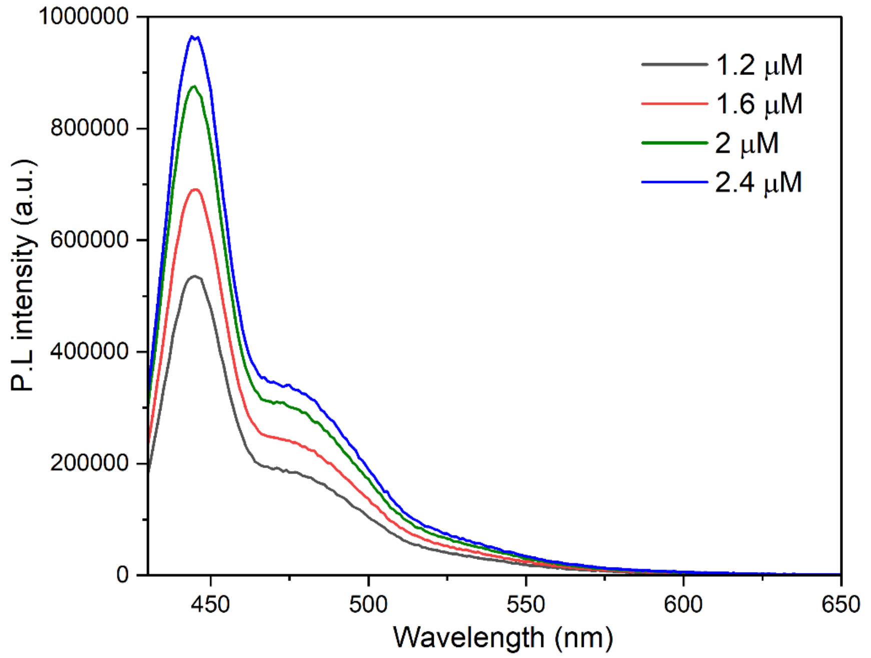

Figure S21: Concentration dependent emission spectra of FA-CP

Plots used for Quantum Yield calculations

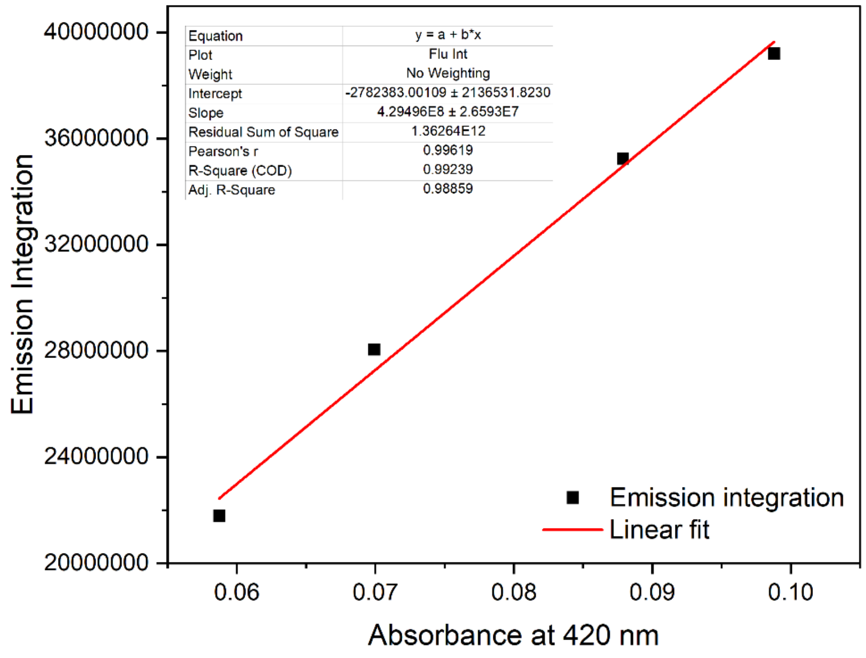

Figure S22: FA-CP

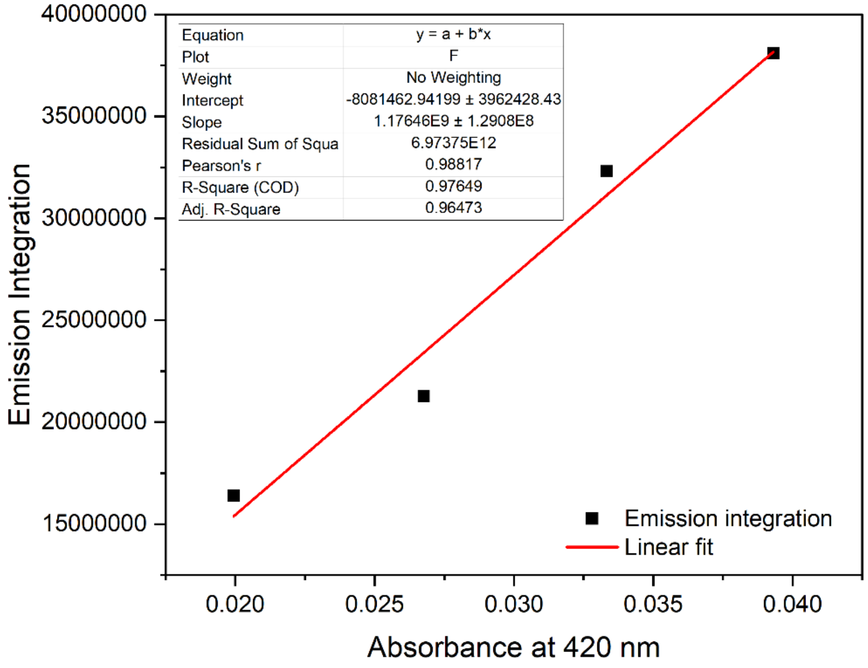

Figure S23: Perylene (reference)

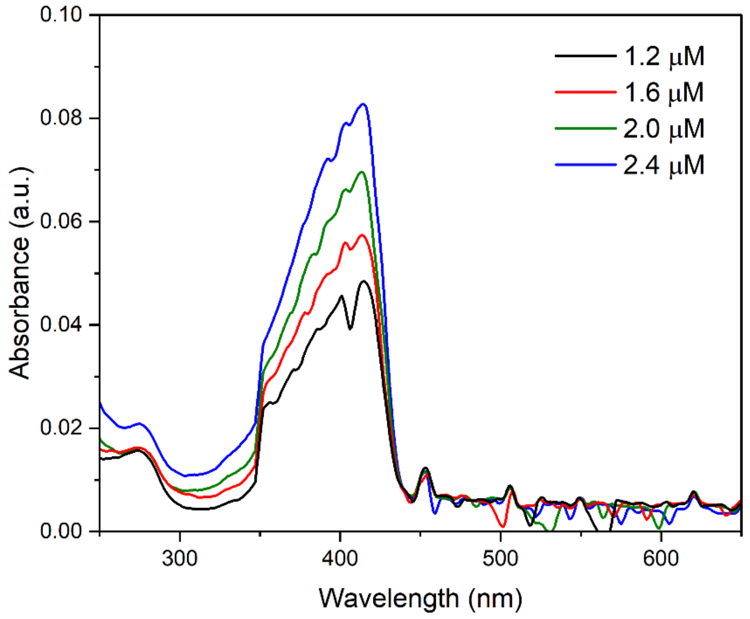

Figure S24: Concentration dependent UV-Vis absorption spectra of *p*PP6-CP

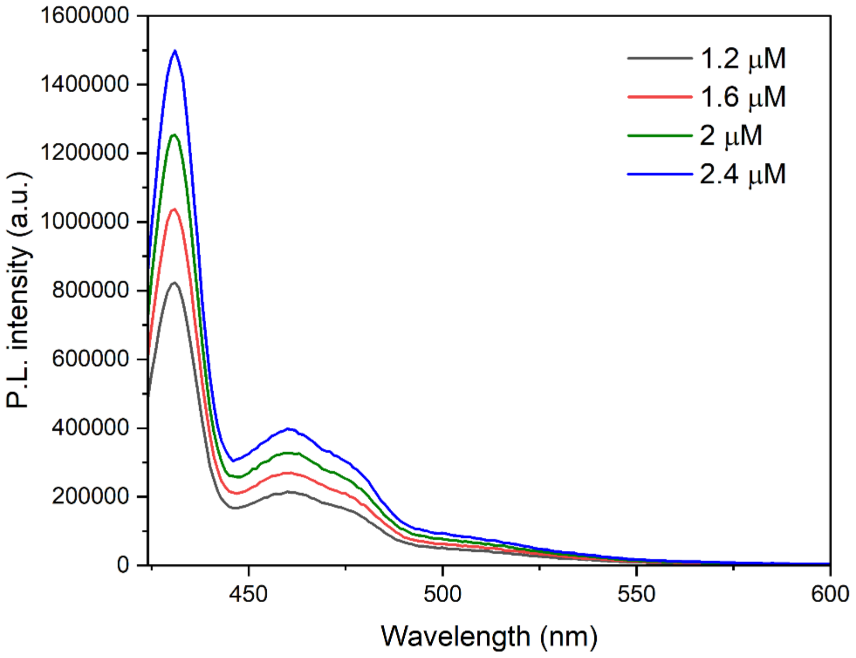

Figure S25: Concentration dependent emission spectra of *p*PP6-CP

Plots used for Quantum Yield calculations

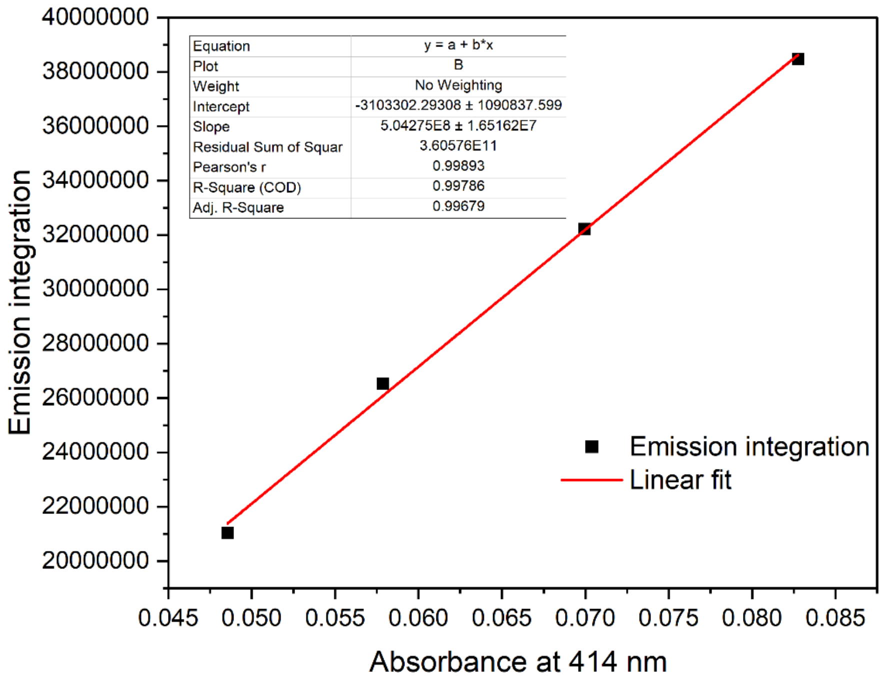

Figure S26: *p*PP6-CP

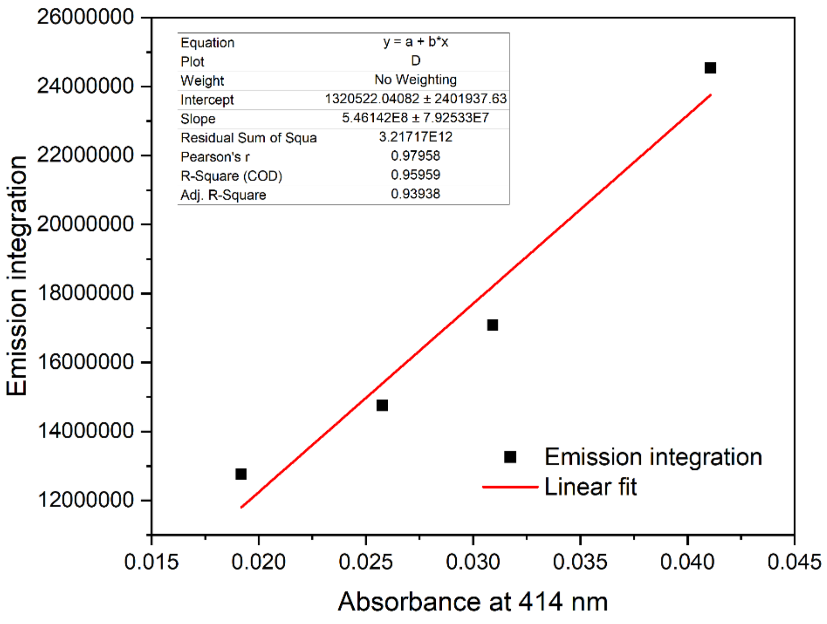

Figure S27: Perylene (reference)

7.0 **Fluorescence quenching**

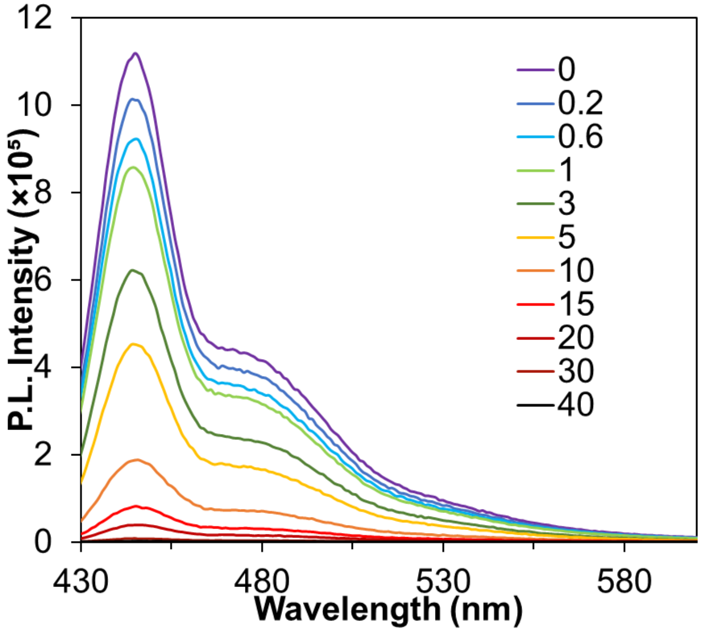

Figure S28: Quencher concentration dependent emission spectra of *p*PA-CP (legend: equivalents of TCNQ with respect to polymer repeat unit concentration)

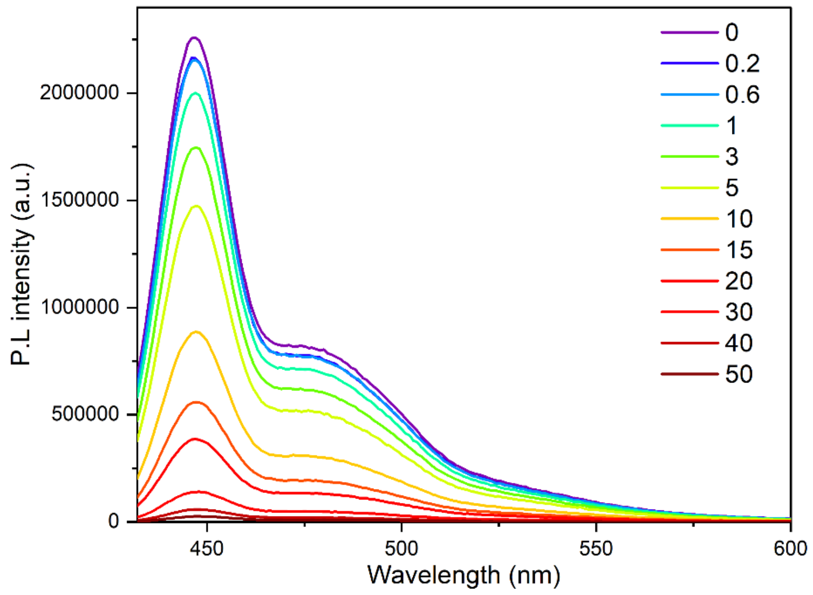

Figure S29: Quencher concentration dependent emission spectra of P6A-CP (legend: equivalents of TCNQ with respect to polymer repeat unit concentration)

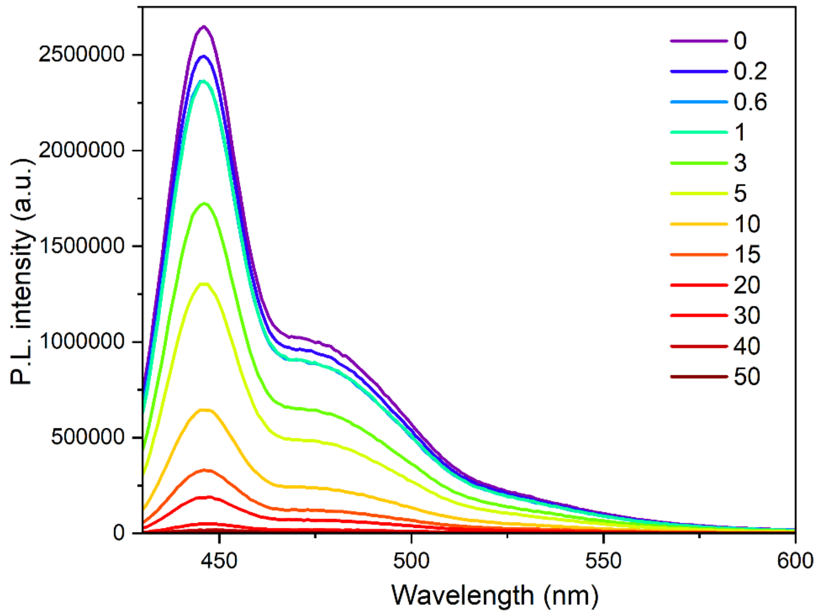

Figure S10: Quencher concentration dependent emission spectra of FA-CP (legend: equivalents of TCNQ with respect to polymer repeat unit concentration)

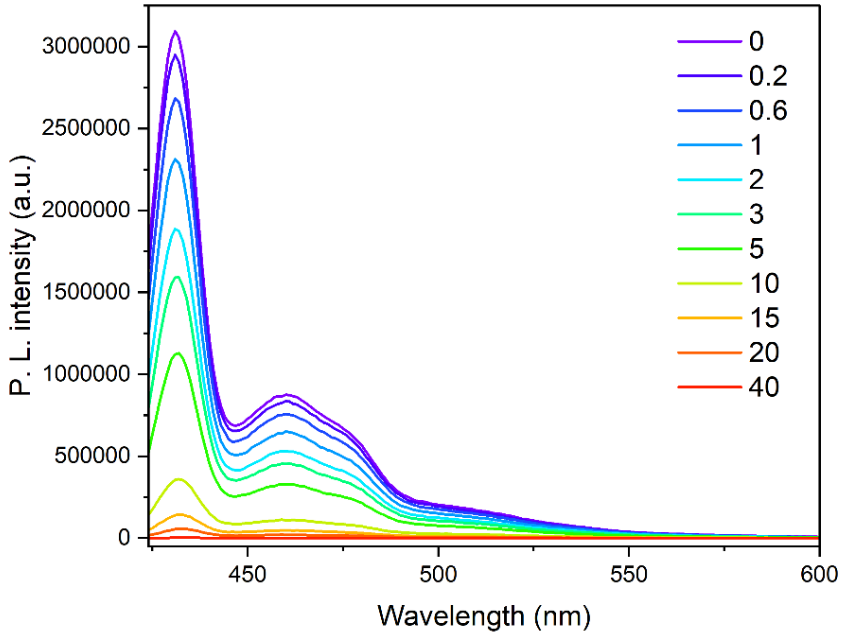

Figure S31: Quencher concentration dependent emission spectra of *p*PP6-CP (legend: equivalents of TCNQ with respect to polymer repeat unit concentration)

8.0 **Non-linear Stern-Volmer Plots of Copolymers**

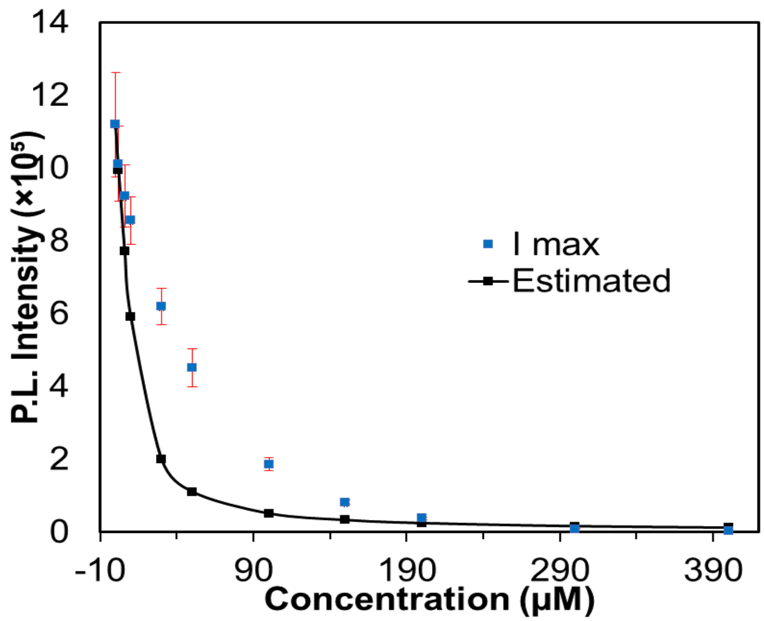

Figure S32*:* *p*PA-CP

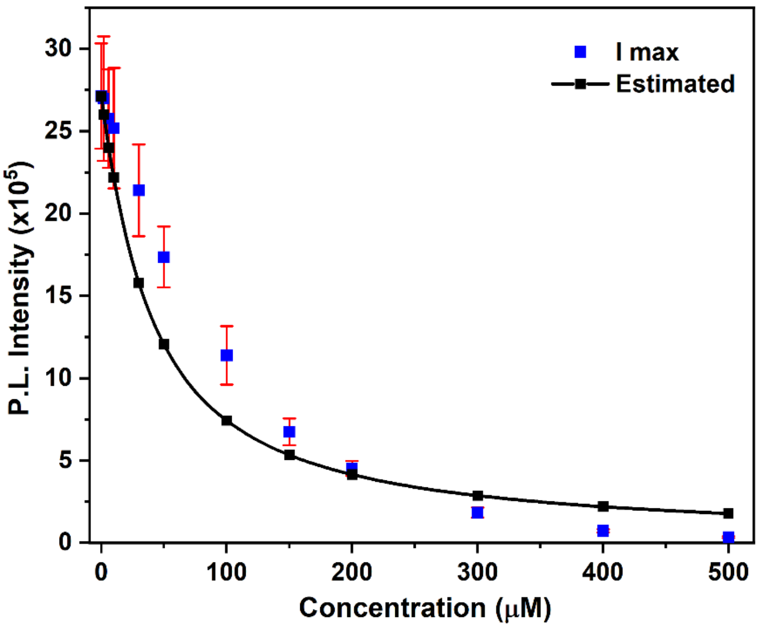

Figure S33: P6A-CP

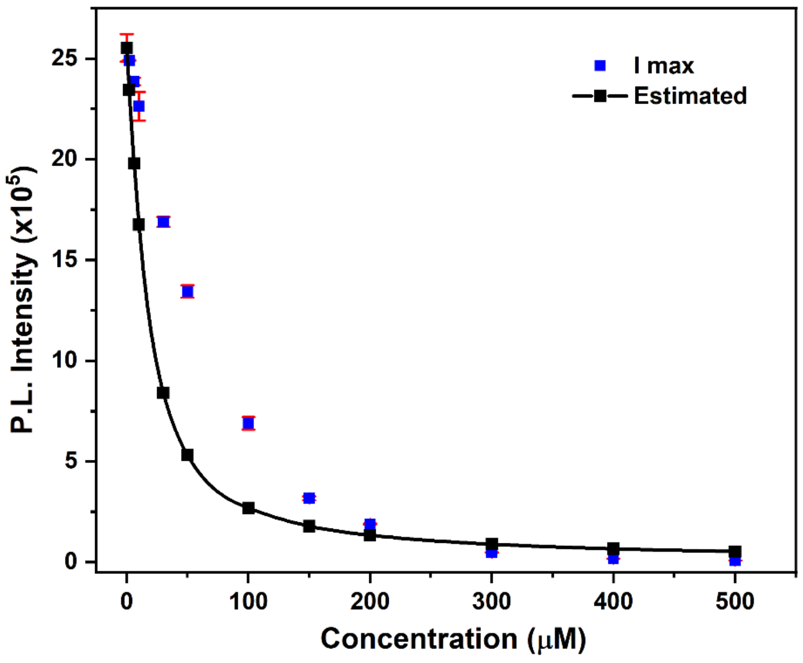

Figure S34: FA-CP

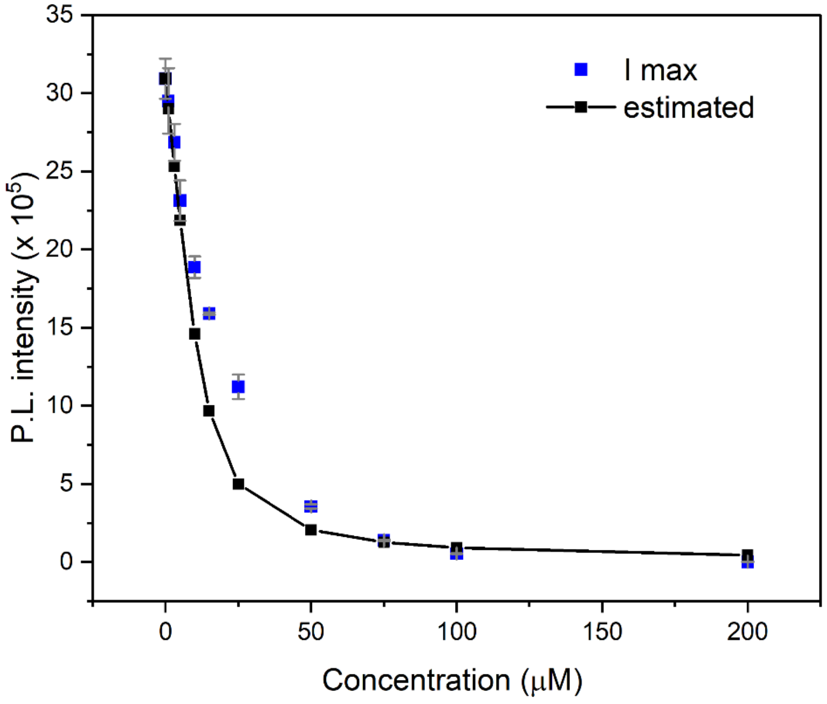

Figure S35: *p*PP6-CP

9.0 **Linear Stern-Volmer plots**

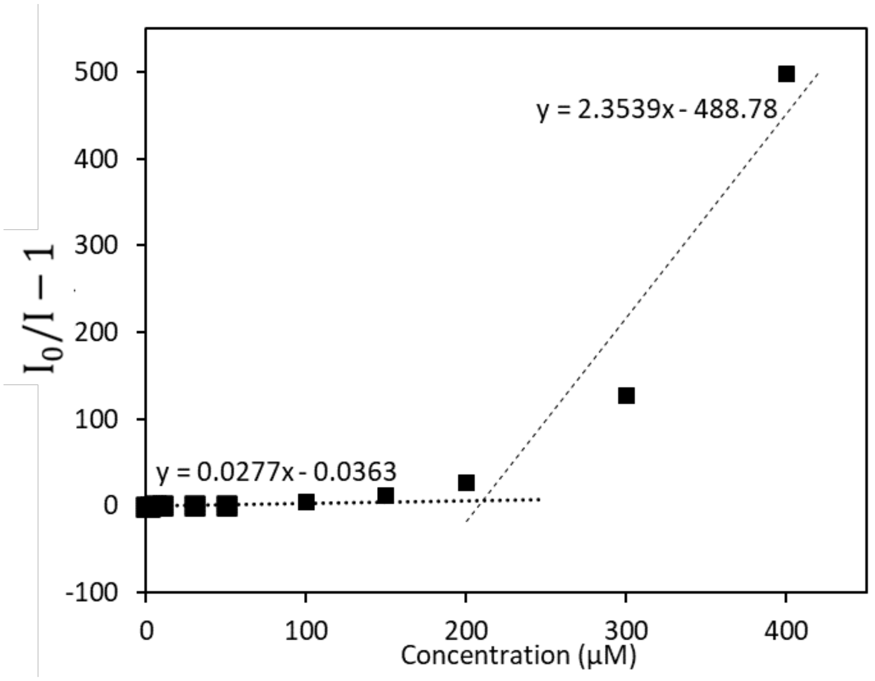

Figure S36: *p*PA-CP

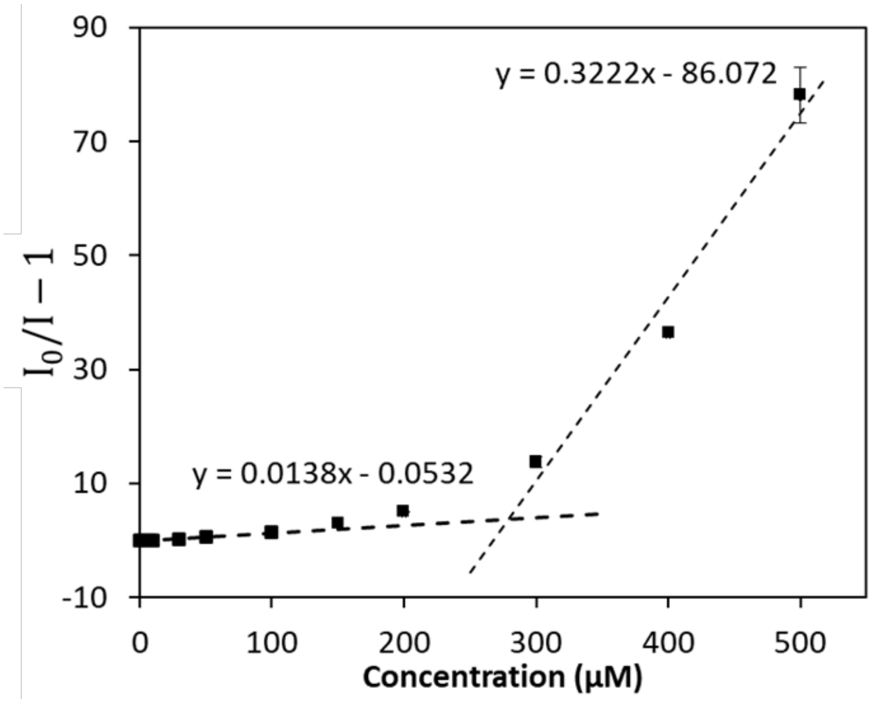

Figure S37: P6A-CP

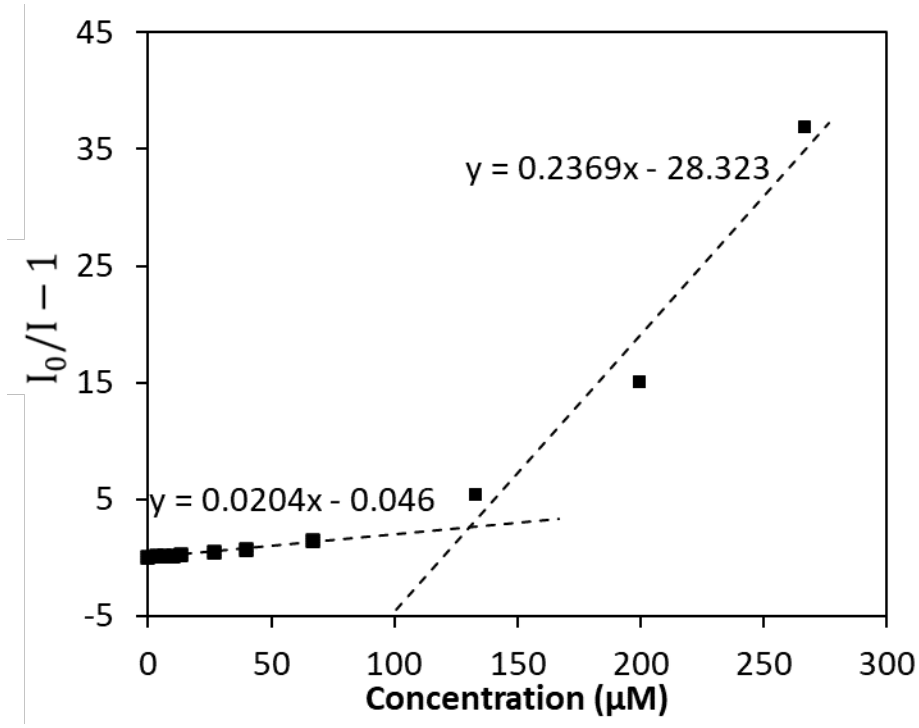

Figure S38: PnA-CP

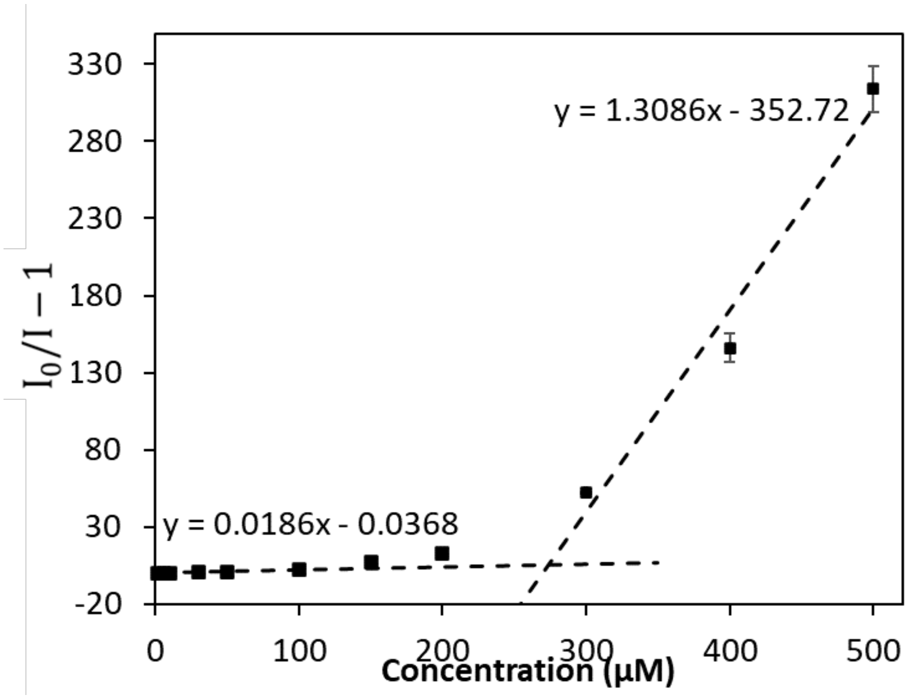

Figure S39: FA-CP

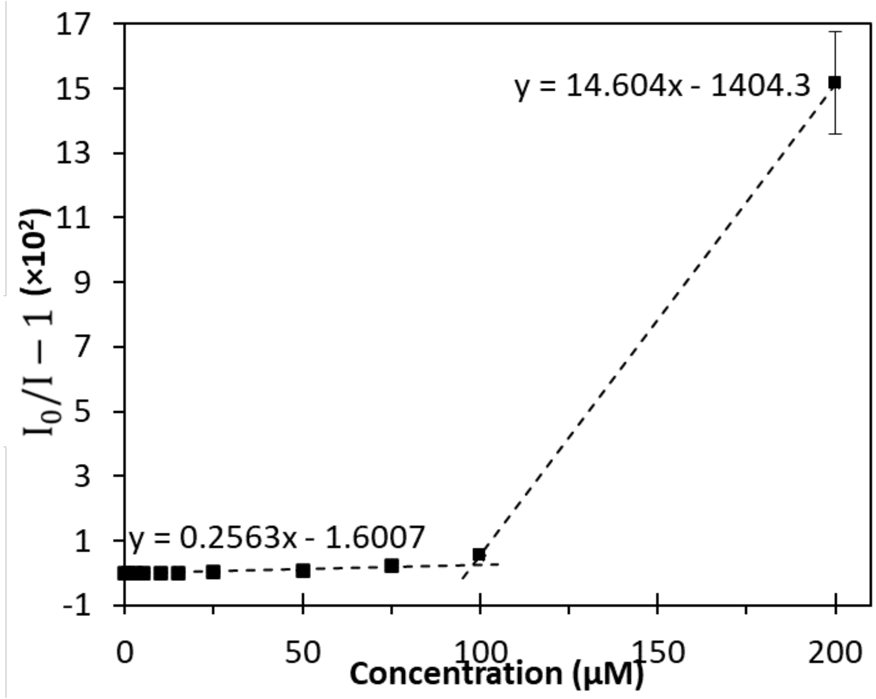

Figure S40: *p*PP6-CP

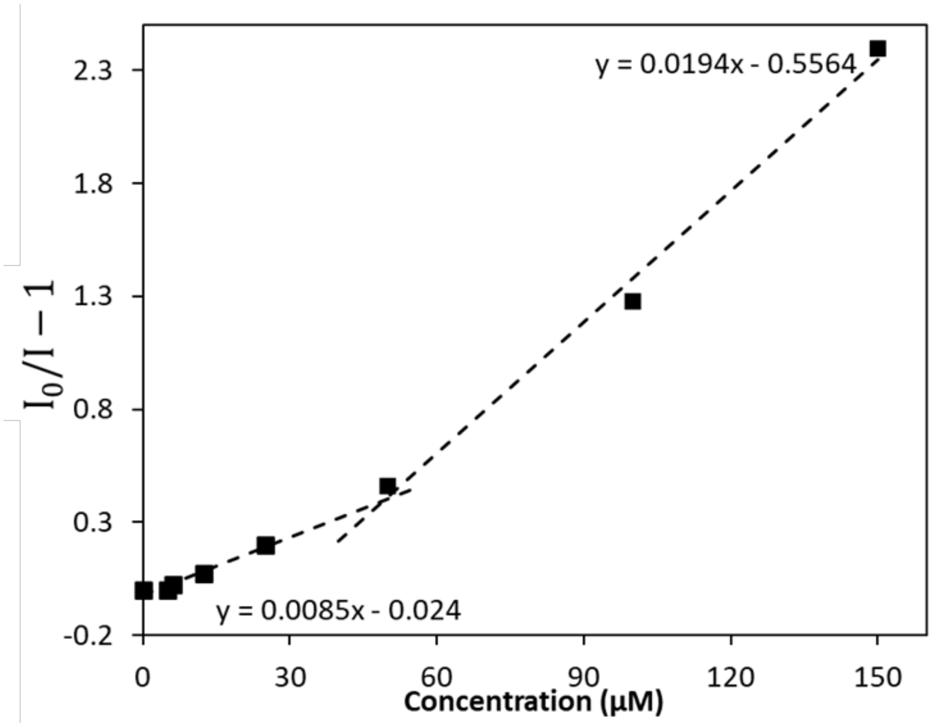

Figure S41: A-HP

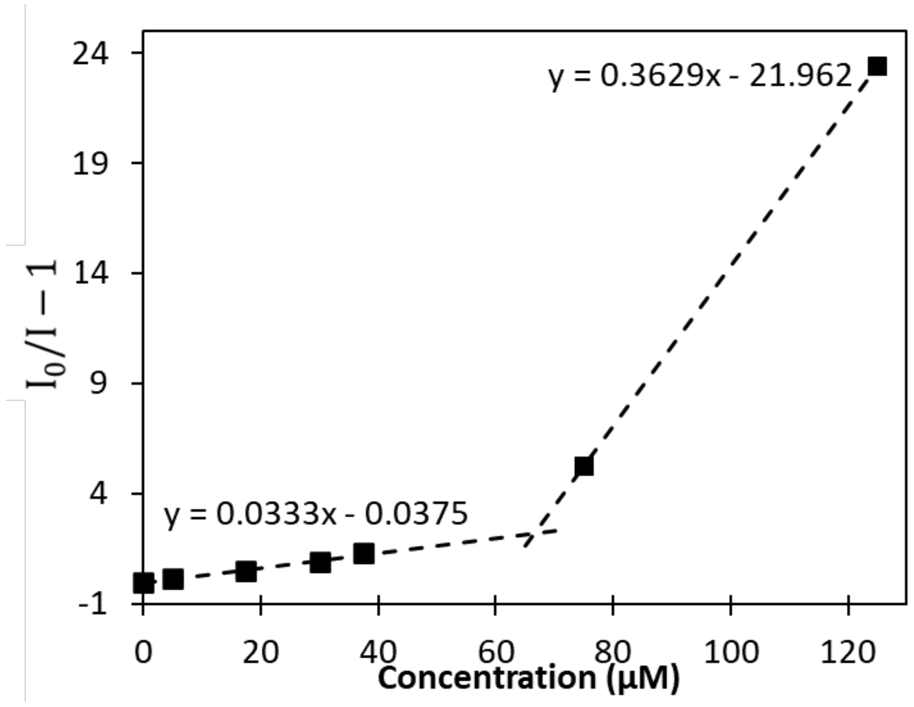

Figure S42: P6-HP

10.0 **Cyclic Voltammograms:**

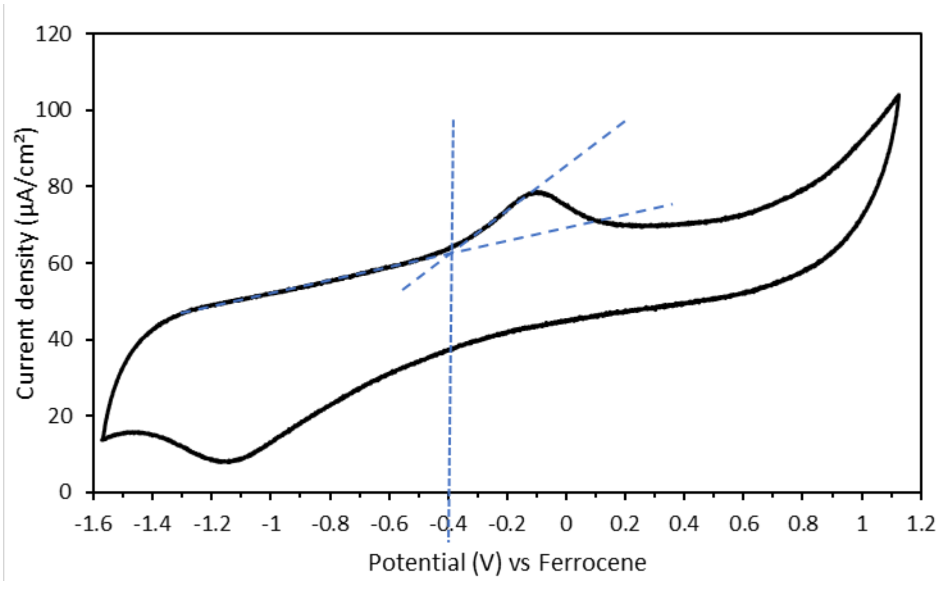

Figure S43: *p*PA-CP

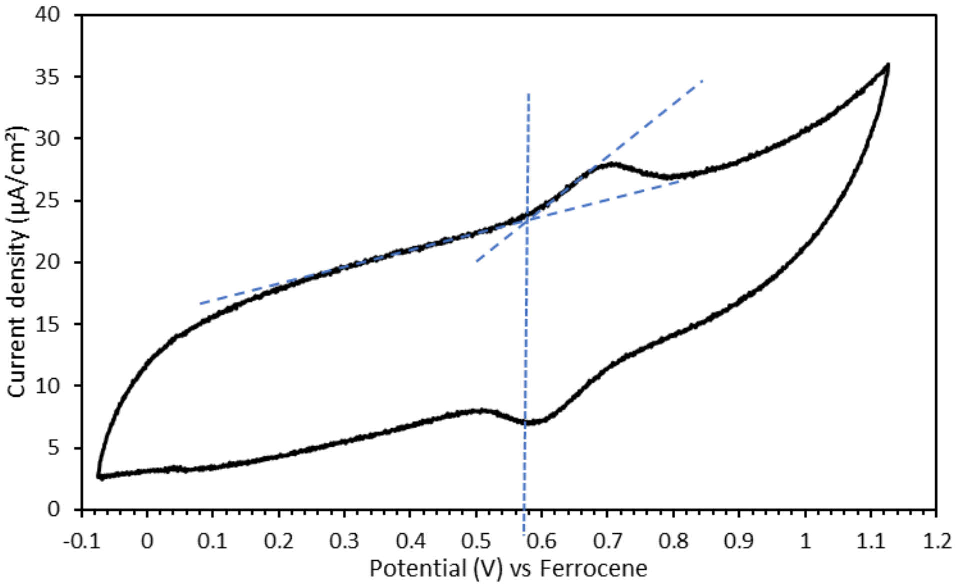

Figure S44: P6A-CP

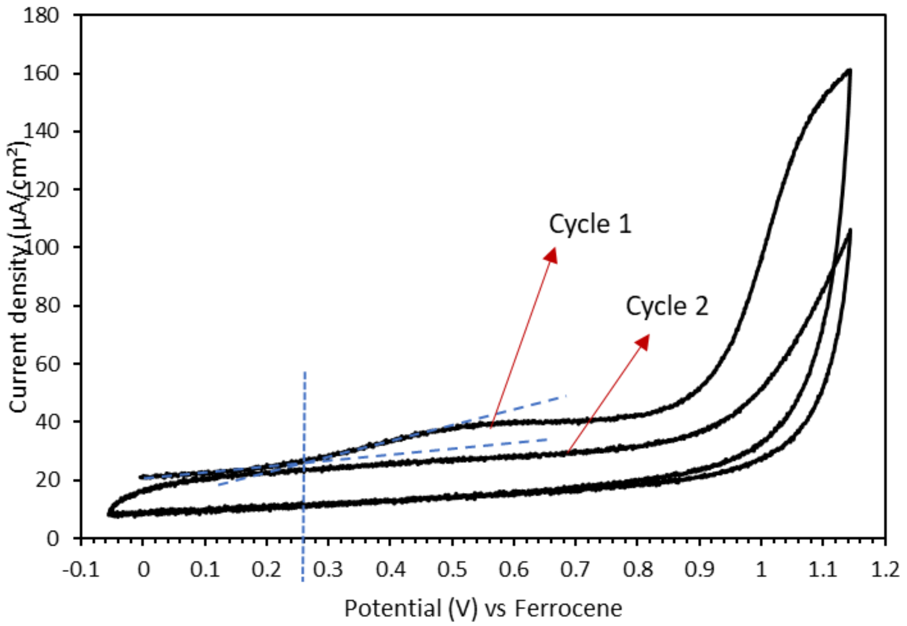

Figure S45: FA-CP

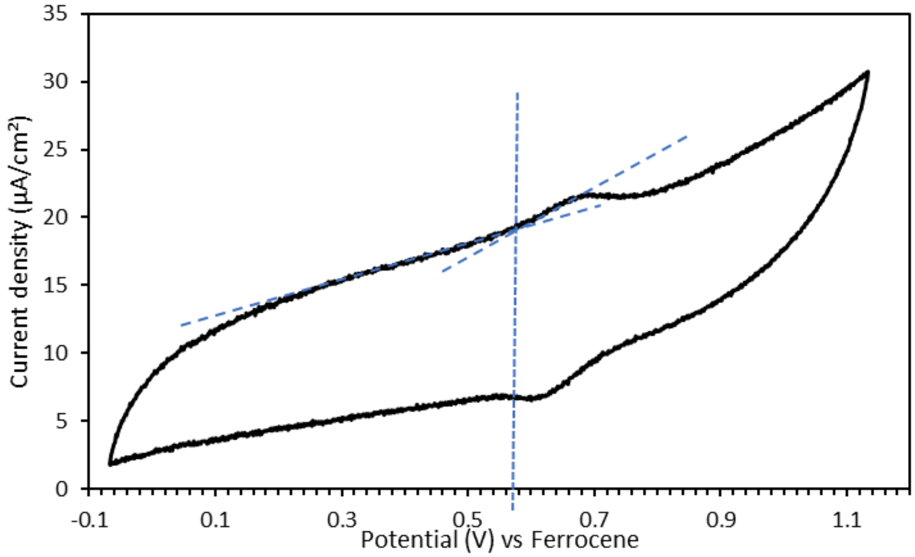

Figure S46: A-HP

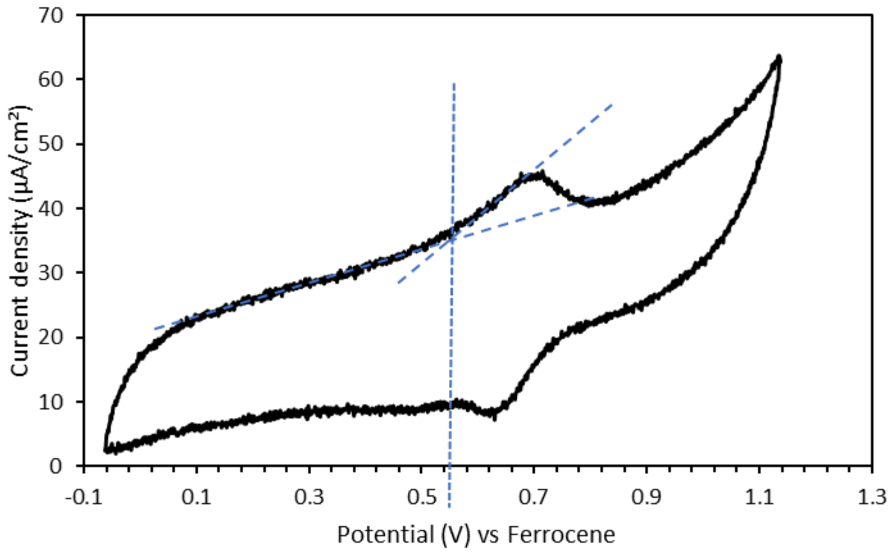

Figure S47: P6-HP

Table S2: ΔG_{ET} values from E^{ox} onset potentials using Rehm-Weller equation (below)[14]

**Polymers**	**$E_{(0,0)}$** (nm)	**$E_{(0,0)}$** (eV)	**E^{ox}** (eV)	**ΔG_{ET}** (eV)
*p*PA-CP	435	2.85	-	-
P6A-CP	438	2.83	-5.37	-3.66
FA-CP	436	2.84	-5.06	-3.36
A-HP	433	2.86	-5.38	-3.7
P6-HP	427	2.90	-5.35	-3.71

$$\Delta G_{\mathrm{ET}} = E_{1/2}{}^{\mathrm{ox}} - E_{1/2}{}^{\mathrm{red}} - E_{(0,0)} + C$$

$E_{1/2}{}^{red}$ of TCNQ= -4.54 eV

11.0 **Computational Methods:**

Charge Transfer complexes between the designed electron donors with TCNQ as an acceptor were computationally modeled using Density Functional Theory (DFT). Geometries were optimized using B3LYP/6-311G** with added dispersion correction (GD3BJ) for all systems. All investigated geometries for ApPA-TCNQ, AP6A-TCNQ, APnA-TCNQ, AFA-TCNQ, P6pPP6-TCNQ, AAA-TCNQ and P6P6P6-TCNQ complexes can be seen in Figures S53-S59.

[a] BE (binding energy, BE = $-(E_{complex} - E_{donor} - E_{TCNQ})$. [b] ΔBE_r : relative binding energy (Δ BE_r = BE (most stable configuration) − BE(complex)). [c] BF: Boltzmann factor (BF = g × $e^{-\Delta BEr/RT}$ where g describes the degeneracy of each energy level; ΔBE_r: relative binding energy of the complex).

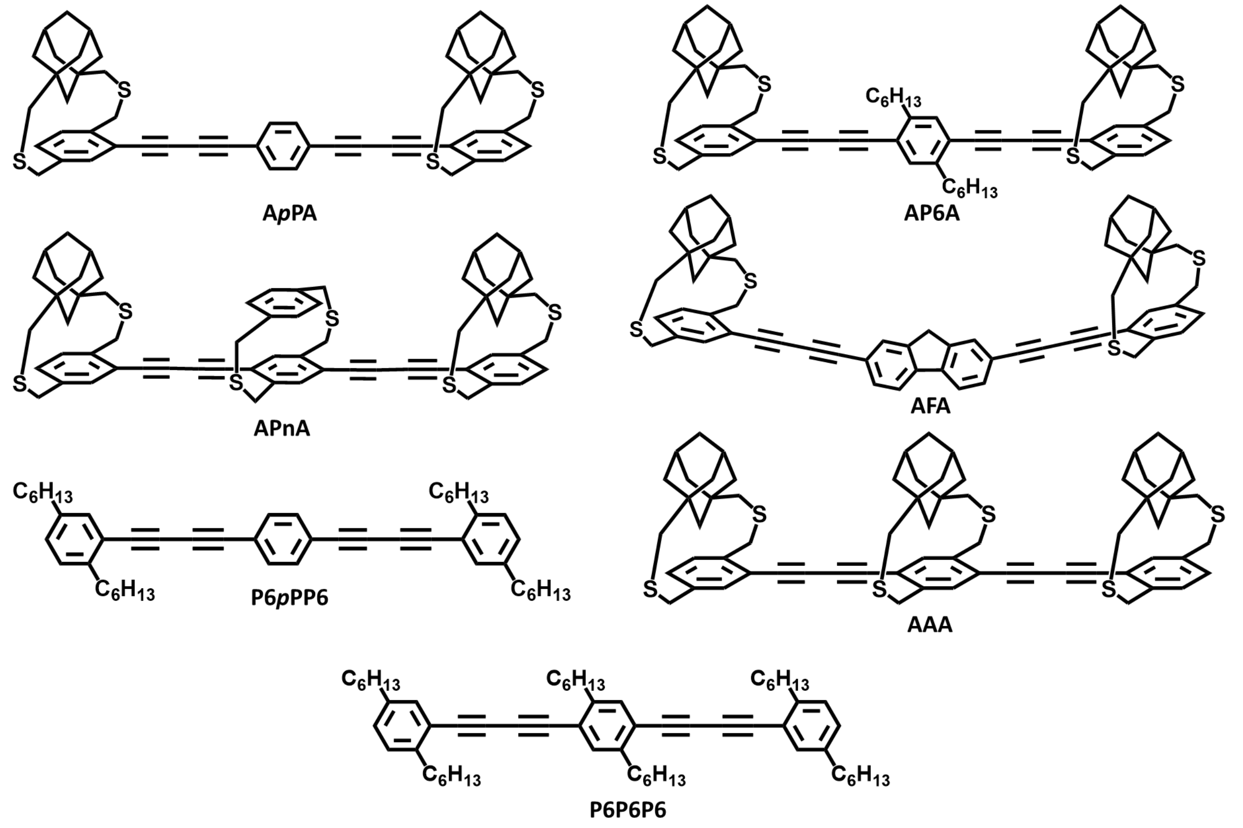

Figure S48: Trimer structures

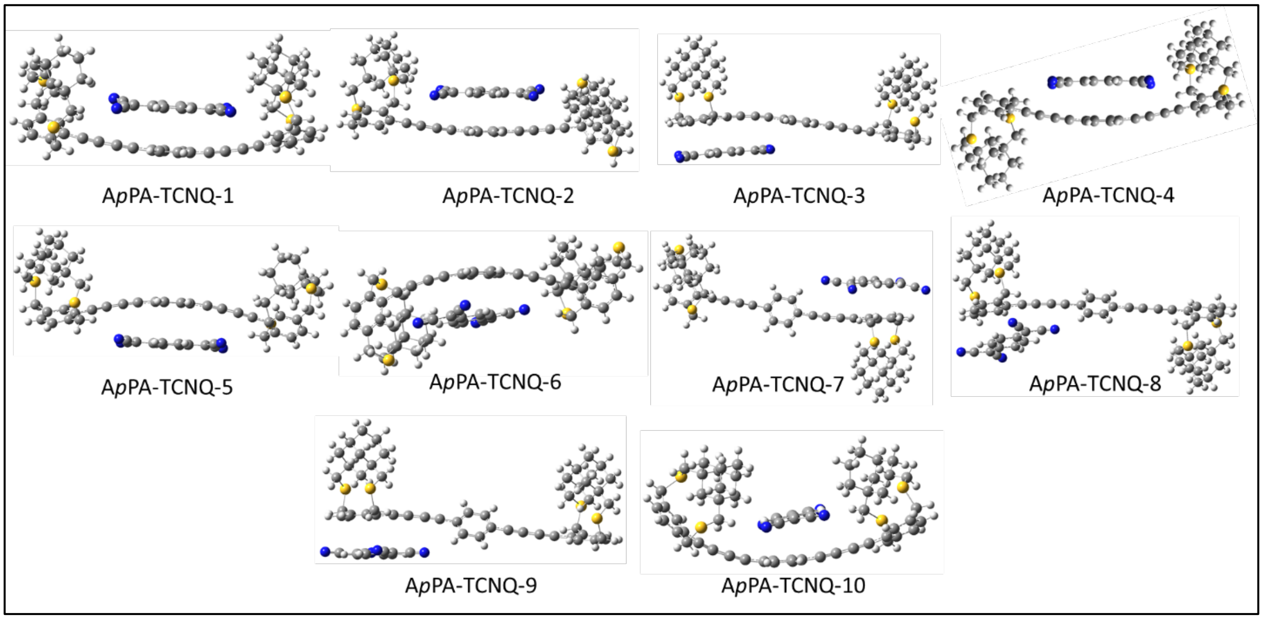

Complex	BE[a] (kcal mol^{-1})	ΔBE[b] (kcal mol^{-1})	BF[c]	% configuration
A*p*PA-TCNQ-1	23.12	0	1	41%
A*p*PA-TCNQ-2	23.06	0.06	0.904	37%
A*p*PA-TCNQ-3	22.2	0.92	0.424	17%
A*p*PA-TCNQ-4	21.21	1.91	0.0798	3.2%
A*p*PA-TCNQ-5	20.24	2.88	0.00777	0.3%
A*p*PA-TCNQ-6	19.8	3.32	0.0074	0.3%
A*p*PA-TCNQ-7	18.38	4.74	0.000674	<0.1%
A*p*PA-TCNQ-8	18.24	4.88	0.000532	<0.1%
A*p*PA-TCNQ-9	18.15	4.97	0.000458	<0.1%
A*p*PA-TCNQ-10	17.7	5.42	0.000107	<0.1%

Figure S49: Different geometries for A*p*PA-TCNQ donor/acceptor complex

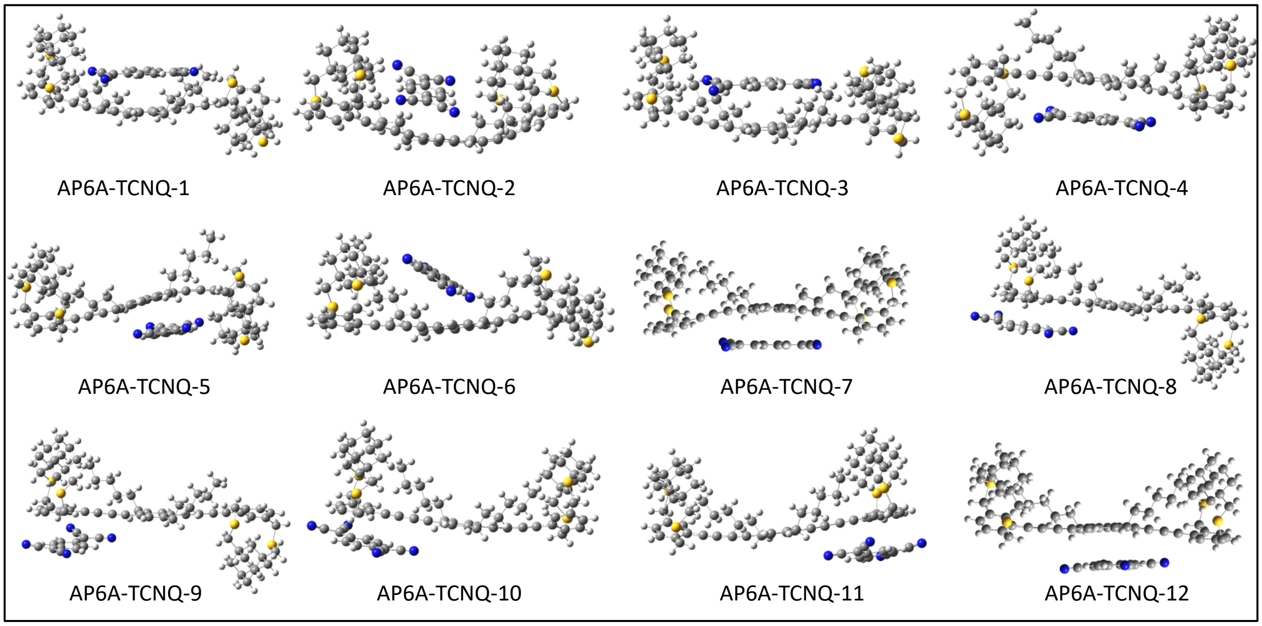

Complex	BE[a] (kcal mol^{-1})	ΔBE[b] (kcal mol^{-1})	BF[c]	% configuration
AP6A-TCNQ-1	25.61	0	1	41%
AP6A-TCNQ-2	25.46	0.15	0.77	32%
AP6A-TCNQ-3	25.31	0.3	0.6	25%
AP6A-TCNQ-4	23.56	2.05	0.03	1%
AP6A-TCNQ-5	22.15	3.46	0.003	0.10%
AP6A-TCNQ-6	21.6	4.01	0.001	<0.1%
AP6A-TCNQ-7	21.29	4.32	0.0007	<0.1%
AP6A-TCNQ-8	19.27	6.34	0.00004	<0.1%
AP6A-TCNQ-9	19.09	6.52	0.00003	<0.1%
AP6A-TCNQ-10	18.49	7.12	0.00001	<0.1%
AP6A-TCNQ-11	18.3	7.31	0.000008	<0.1%
AP6A-TCNQ-12	18.07	7.54	0.000003	<0.1%

Figure S50: Different geometries for AP6A-TCNQ donor/acceptor complex

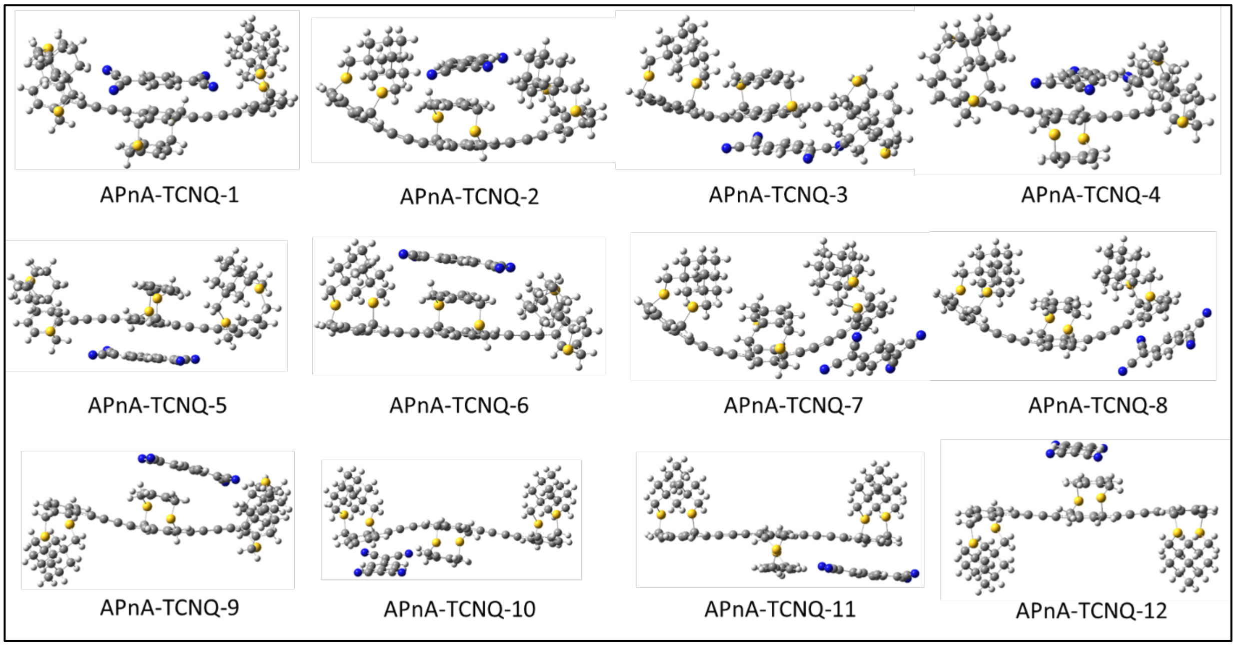

Complex	BE[a] (kcal mol^{-1})	ΔBE[b] (kcal mol^{-1})	BF[c]	% configuration
APnA-TCNQ-1	26.43	0.00	1.00	84.18
APnA-TCNQ-2	25.25	1.18	0.14	11.60
APnA-TCNQ-3	24.22	2.21	0.02	2.02
APnA-TCNQ-4	24.19	2.24	0.02	1.93
APnA-TCNQ-5	22.74	3.69	0.00	0.17
APnA-TCNQ-6	22.41	4.02	0.00	0.10
APnA-TCNQ-7	20.10	6.33	0.00	0.00
APnA-TCNQ-8	20.03	6.40	0.00	0.00
APnA-TCNQ-9	19.81	6.62	0.00	0.00
APnA-TCNQ-10	19.10	7.33	0.00	0.00
APnA-TCNQ-11	18.64	7.79	0.00	0.00
APnA-TCNQ-12	14.89	11.54	0.00	0.00

Figure S51: Different geometries for APnA-TCNQ donor/acceptor complex

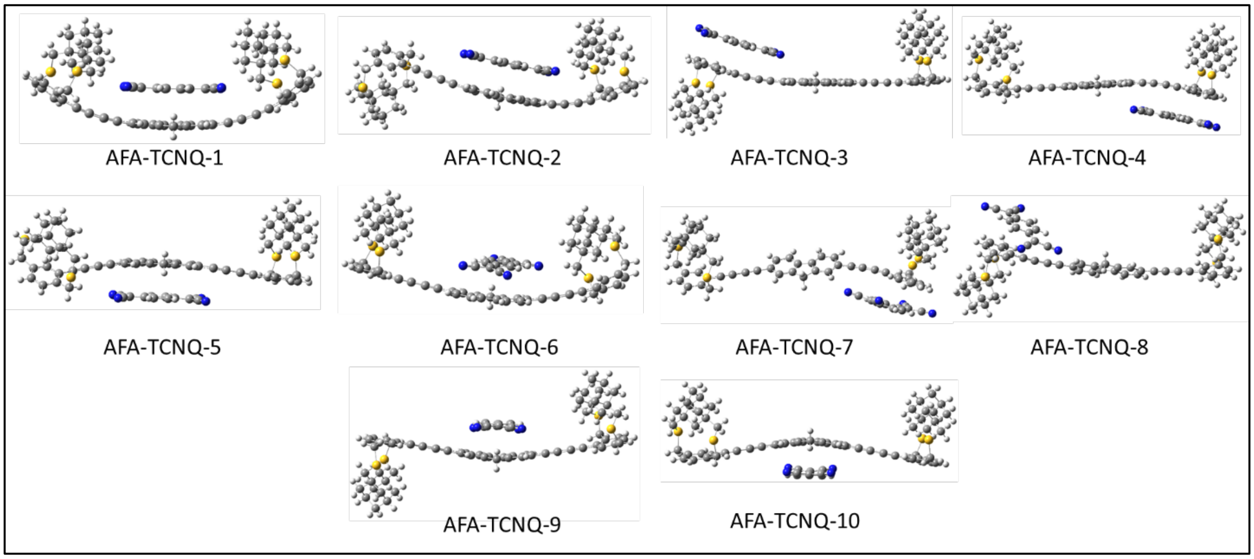

Complex	BE[a] (kcal mol^{-1})	ΔBE[b] (kcal mol^{-1})	BF[c]	% configuration
AFA-TCNQ-1	27.39	0	1	95%
AFA-TCNQ-2	25.11	2.28	0.042	4%
AFA-TCNQ-3	23.07	4.32	0.00274	0.26%
AFA-TCNQ-4	23.05	4.34	0.00133	0.1%
AFA-TCNQ-5	22.85	4.54	0.00047	< 0.1%
AFA-TCNQ-6	18.84	8.55	0.000000547	< 0.1%
AFA-TCNQ-7	18.39	9	0.000000256	< 0.1%
AFA-TCNQ-8	18.38	9.01	0.0000005	< 0.1%
AFA-TCNQ-9	17.45	9.94	0.0000001	< 0.1%
AFA-TCNQ-10	17.41	9.98	0.0000000491	< 0.1%

Figure S52: Different geometries for AFA-TCNQ donor/acceptor complex

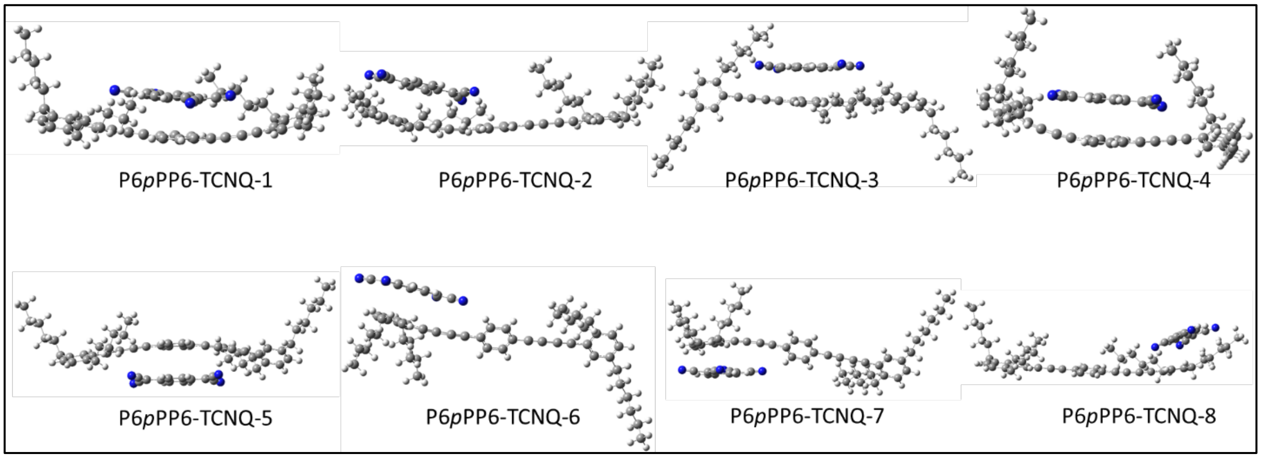

Complex	BE[a] (kcal mol^{-1})	ΔBE[b] (kcal mol^{-1})	BF[c]	% configuration
P6*p*PP6-TCNQ-1	23.84	0	1	43%
P6*p*PP6-TCNQ-2	23.45	0.39	1.036	45%
P6*p*PP6-TCNQ-3	22.78	1.06	0.167	7%
P6*p*PP6-TCNQ-4	22.46	1.38	0.0975	4%
P6*p*PP6-TCNQ-5	20.33	3.51	0.0027	0.1%
P6*p*PP6-TCNQ-6	19.43	4.41	0.00118	<0.1%
P6*p*PP6-TCNQ-7	19.39	4.45	0.00110	<0.1%
P6*p*PP6-TCNQ-8	15.78	8.06	0.0000025	<0.1%

Figure S53: Different geometries for P6*p*PP6-TCNQ donor/acceptor complex

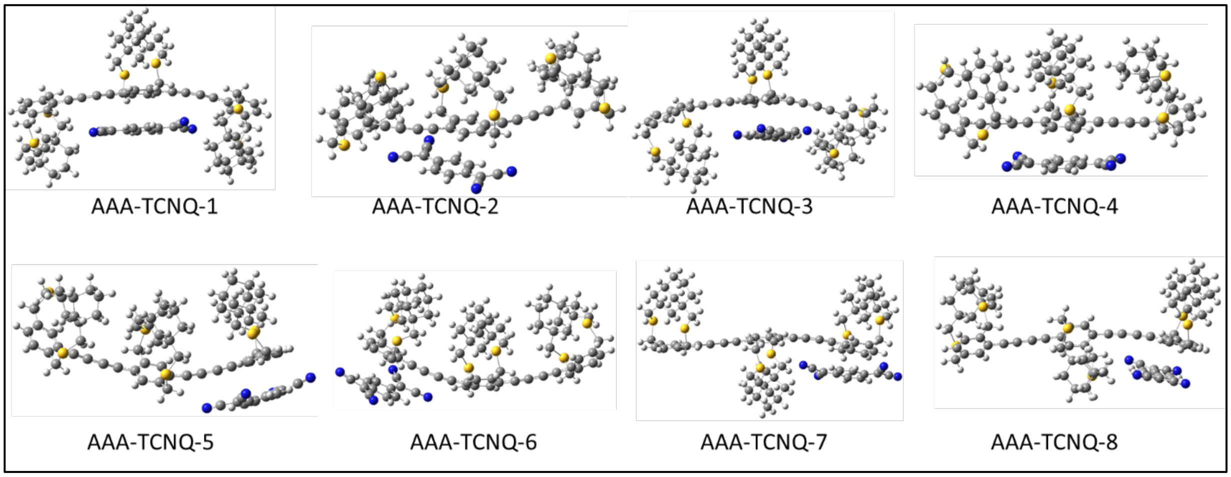

	BE[a] (kcal mol^{-1})	ΔBE[b] (kcal mol^{-1})	BF[c]	% configuration
AAA-TCNQ-1	21.46	0.00	1.00	70.40
AAA-TCNQ-2	20.87	0.59	0.37	26.15
AAA-TCNQ-3	19.44	2.01	0.03	2.35
AAA-TCNQ-4	18.60	2.86	0.01	0.57
AAA-TCNQ-5	17.54	3.91	0.00	0.19
AAA-TCNQ-6	17.54	3.91	0.00	0.19
AAA-TCNQ-7	17.34	4.11	0.00	0.14
AAA-TCNQ-8	17.20	4.25	0.00	0.11

Figure S54: Different geometries for AAA-TCNQ donor/acceptor complex

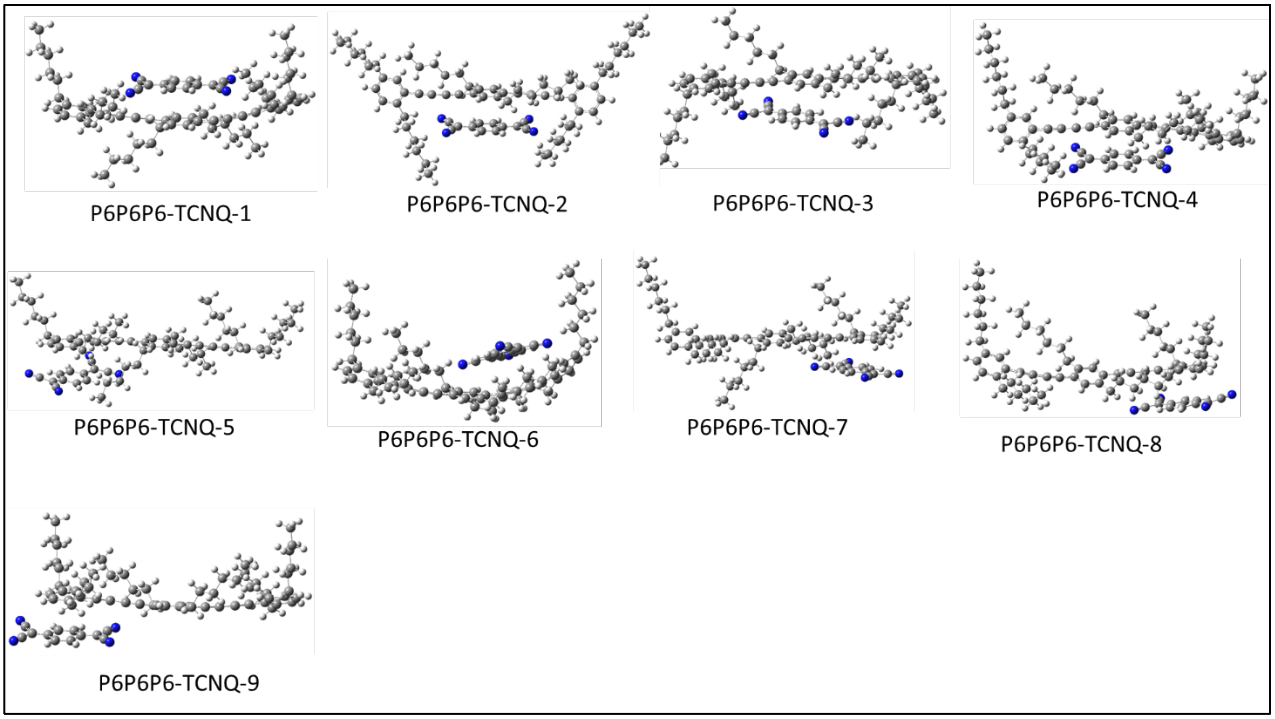

	BE[a] (kcal mol^{-1})	ΔBE[b] (kcal mol^{-1})	BF[c]	% configuration
P6P6P6-TCNQ-1	25.94	0.00	1.00	53.26
P6P6P6-TCNQ-2	25.69	0.25	0.66	35.10
P6P6P6-TCNQ-3	24.98	0.96	0.20	10.51
P6P6P6-TCNQ-4	23.37	2.57	0.01	0.70
P6P6P6-TCNQ-5	22.79	3.15	0.00	0.26
P6P6P6-TCNQ-6	22.12	3.82	0.00	0.09
P6P6P6-TCNQ-7	21.74	4.21	0.00	0.04
P6P6P6-TCNQ-8	21.69	4.26	0.00	0.04
P6P6P6-TCNQ-9	19.14	6.80	0.00	0.00

Figure S55: Different geometries for P6P6P6-TCNQ donor/acceptor complex

12.0 ^{1}H NMR Spectra

Percentage of non-strapped comonomer content:

Following equation is used to calculate the percentage of non-strapped comonomer content in the copolymers:

$$\%\, non-strapped\ comonomer\ content = \frac{n_B}{n_A + n_B} \times 100$$

n_A is the moles of strapped comonomer and is set as 1, and n_B is equal to moles of non-strapped comonomer. n_B is determined by taking the ratio of non-strapped comonomer aryl protons integration (I_{ns}) with the actual number of aryl protons in non-strapped unit. In order to determine I_{ns}, the integration of the highly shielded methylene protons of adamantyl (between 0 and -0.3 ppm) was set as 2 and rest of the peaks in the spectra were integrated. I_{ns} was determined by subtracting the total integration of aryl protons with 2 (arising from the phenylene protons of the adamantyl strapped unit).

$$n_B = \frac{I_{ns}\ (integration\ of\ comonomer\ in\ NMR)}{number\ of\ actual\ protons\ of\ comonomer\ in\ 1\ repeat\ unit}$$

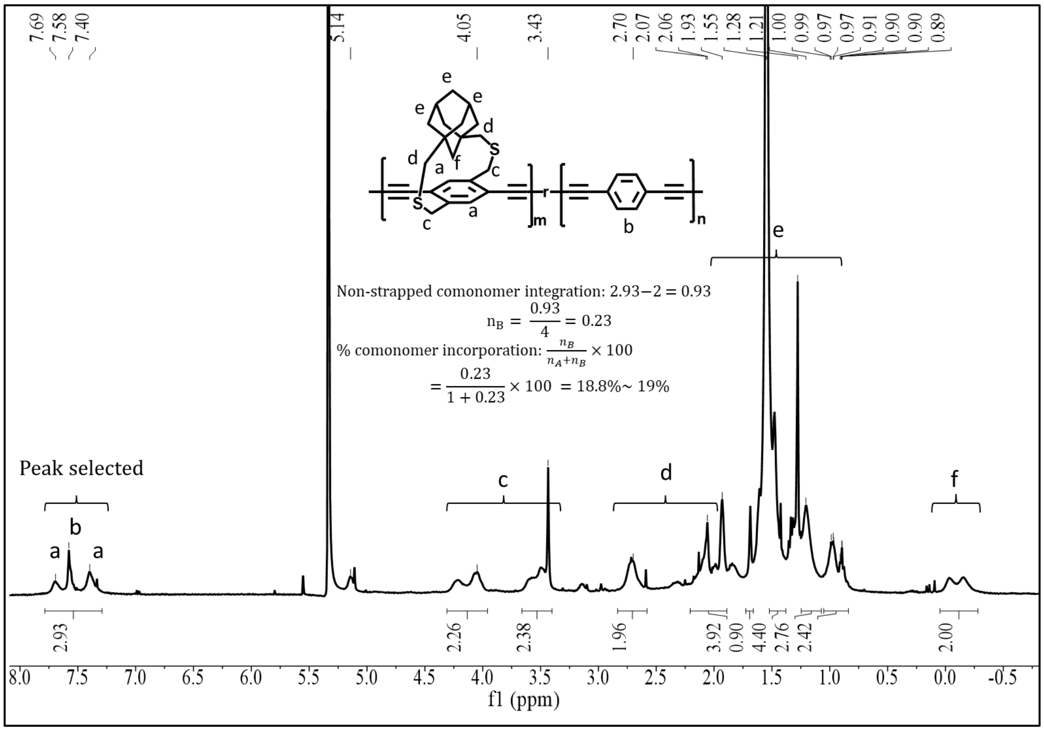

Figure S56: ^{1}H-NMR of *p*PA-CP in CD_2Cl_2

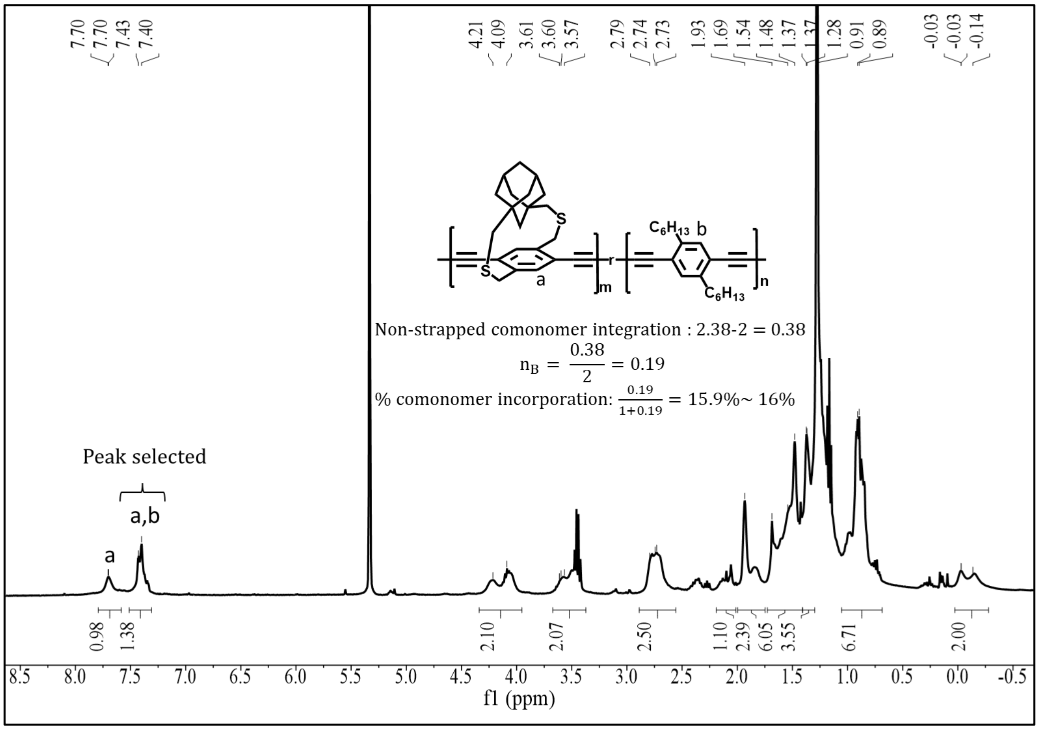

Figure S57: ^{1}H-NMR of P6A-CP in CD_2Cl_2

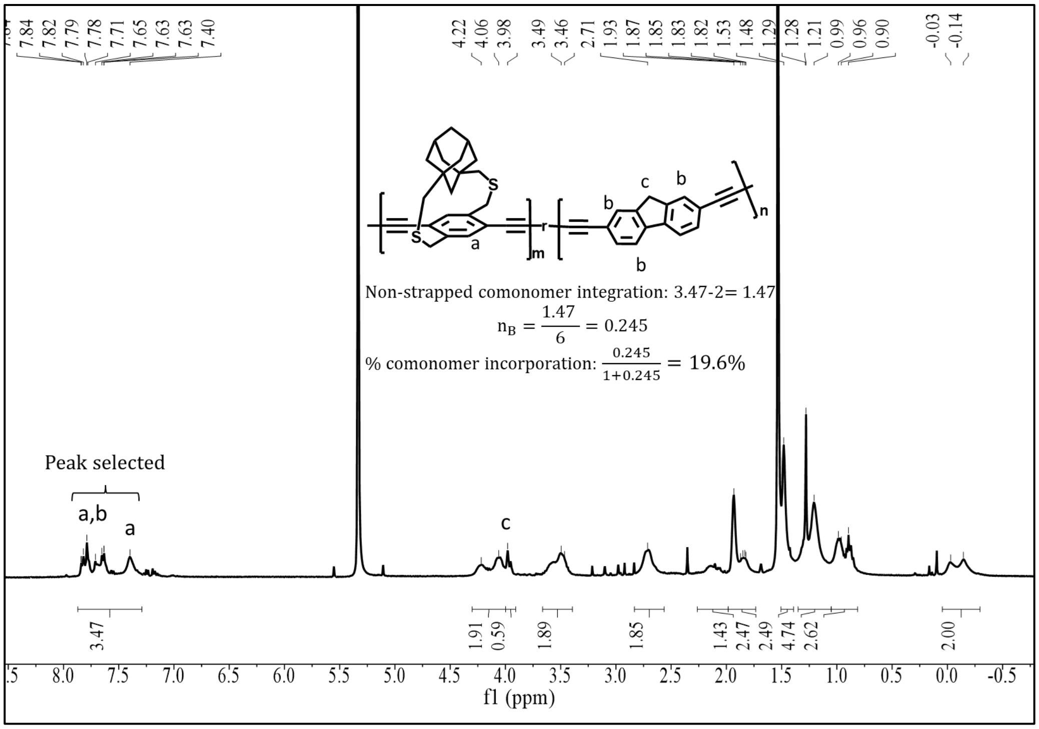

Figure S58: ^{1}H-NMR of FA-CP in CD_2Cl_2

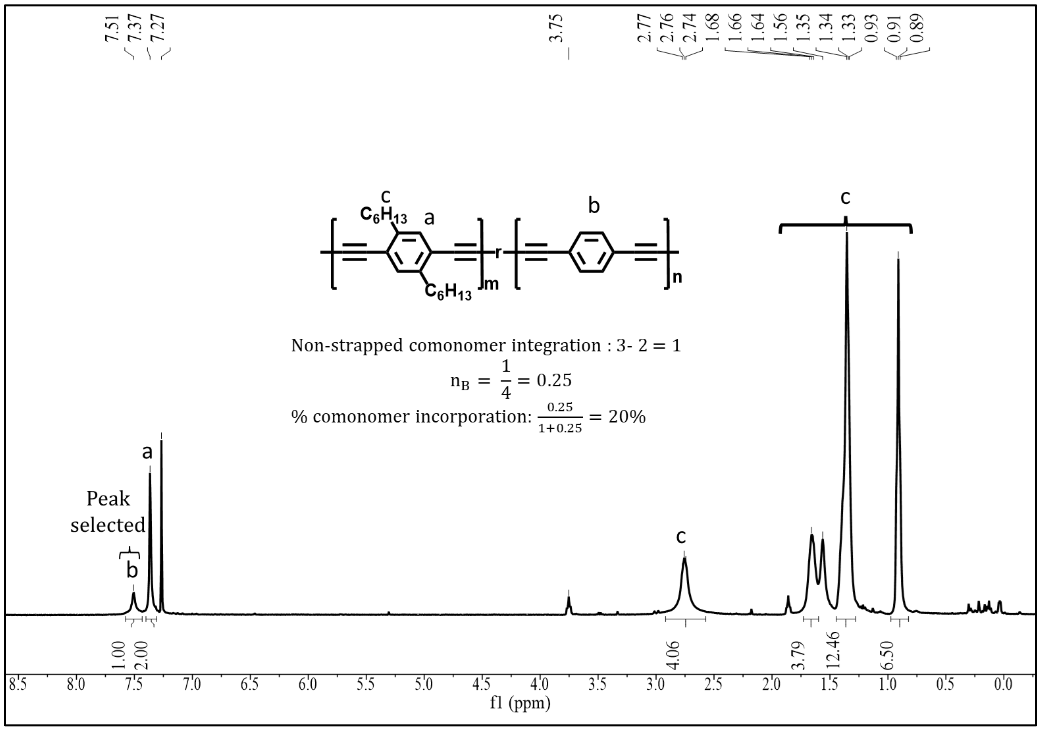

Figure S59: ^{1}H-NMR of *p*PP6-CP in $CDCl_3$

References:

1. Huang, F.; Chen, K.-S.; Yip, H.-L.; Hau, S. K.; Acton, O.; Zhang, Y.; Luo, J.; Jen, A. K.-Y., Development of new conjugated polymers with donor− π-bridge− acceptor side chains for high performance solar cells. *J. Am. Chem. Soc.* **2009,** *131* (39), 13886-13887.
2. Zheng, F.; Tan, S.-E.; Yanamoto, Y.; Shida, N.; Nishiyama, H.; Inagi, S.; Tomita, I., Preparation of a germole-containing π-conjugated polymer by the Te–Li exchange reaction of a tellurophene-containing polymer. *NPG Asia Mater.* **2020,** *12* (1), 41.
3. Nagarjuna, G.; Yurt, S.; Jadhav, K. G.; Venkataraman, D., Impact of pendant 1, 2, 3-triazole on the synthesis and properties of thiophene-based polymers. *Macromol.* **2010,** *43* (19), 8045-8050.
4. Tan, C.-H.; Gorman, J.; Wadsworth, A.; Holliday, S.; Subramaniyan, S.; Jenekhe, S. A.; Baran, D.; McCulloch, I.; Durrant, J. R., Barbiturate end-capped non-fullerene acceptors for organic solar cells: tuning acceptor energetics to suppress geminate recombination losses. *Chem comm* **2018,** *54* (24), 2966-2969.
5. Murugesan, V.; de Bettignies, R.; Mercier, R.; Guillerez, S.; Perrin, L., Synthesis and characterizations of benzotriazole based donor–acceptor copolymers for organic photovoltaic applications. *Synth. Met.* **2012,** *162* (11-12), 1037-1045.
6. Yu, C.-Y.; Ko, B.-T.; Ting, C.; Chen, C.-P., Two-dimensional regioregular polythiophenes with conjugated side chains for use in organic solar cells. *Sol. Energy Mater Sol. Cells* **2009,** *93* (5), 613-620.
7. Kumar, G. A.; Priya, P. G.; Alagar, M., Functional phenylethynylene side arm poly (arylene ethynylene) conjugated polymers: optical and electrochemical behavior for enrichment of electronic applications. *New J Chem* **2018,** *42* (8), 5767-5773.
8. Brouwer, A. M., Standards for photoluminescence quantum yield measurements in solution (IUPAC Technical Report). *Pure Appl. Chem.* **2011,** *83* (12), 2213-2228.

9. Campbell, K.; Zappas, A.; Bunz, U.; Thio, Y. S.; Bucknall, D. G., Fluorescence quenching of a poly (para-phenylene ethynylenes) by C60 fullerenes. *J. Photochem. Photobiol.* **2012,** *249*, 41-46.
10. Chaudhuri, S.; Mohanan, M.; Willems, A. V.; Bertke, J. A.; Gavvalapalli, N., β-Strand inspired bifacial π-conjugated polymers. *Chem. Sci.* **2019,** *10* (23), 5976-5982.
11. Lu, L.; Guo, K.; Zhu, J.; Wang, F.; Zhu, Y.; Qi, H., Silicon-containing fluorenylacetylene resins with low curing temperature and high thermal stability. *J. Appl. Polym. Sci.* **2019,** *136* (48), 48262.
12. Lahn, B.; Rehahn, M. In *Coordination polymers from kinetically labile copper (I) and silver (I) complexes: true macromolecules or solution aggregates?*, Macromol. Symp., Wiley Online Library: 2001; pp 157-176.
13. Gantenbein, M.; Li, X.; Sangtarash, S.; Bai, J.; Olsen, G.; Alqorashi, A.; Hong, W.; Lambert, C. J.; Bryce, M. R., Exploring antiaromaticity in single-molecule junctions formed from biphenylene derivatives. *Nanoscale* **2019,** *11* (43), 20659-20666.
14. Chaudhary, A.; Rath, S. P., Encapsulation of TCNQ and the Acridinium Ion within a Bisporphyrin Cavity: Synthesis, Structure, and Photophysical and HOMO–LUMO-Gap-Mediated Electron-Transfer Properties. *Eur. J. Chem.* **2012,** *18* (24), 7404-7417.
