# Supplementary material for: Controlling π-Conjugated Polymer–Acceptor Interactions by Designing Polymers with a Mixture of π-Face Strapped and Nonstrapped Monomers
Source: Macromolecules. 2023 Apr 25;56(9):3421–9. doi: 10.1021/acs.macromol.3c00175 (PMC10950295; doi:10.1021/acs.macromol.3c00175)
Supplement: Supplementary file 2 — ma3c00175_si_002.pdf [file ma3c00175_si_002.pdf]

Supporting Information: DFT

Controlling π-Conjugated Polymer–Acceptor Interactions by Designing Polymers with a Mixture of π-Face Strapped and Non-Strapped Monomers

Fatima Hameed,[a,b] Manikandan Mohanan,[a,b] Nafisa Ibrahim[c], Charles Ochonma[a,b], Joaquín Rodríguez-López[c] and Nagarjuna Gavvalapalli[a,b]*

[a]Department of Chemistry, [b]Institute for Soft Matter Synthesis and Metrology, Georgetown University, Washington, D.C., 20057, USA, [c]Department of Chemistry, University of Illinois Urbana-Champaign, Urbana, IL 61801, USA

* Corresponding author: N.G. (email: ng554@georgetown.edu)

Contents

1.0 TCNQ coordinates S2

2.0 A*p*PA-TCNQ coordinates S3

3.0 AP6A-TCNQ coordinates S38

4.0 APnA-TCNQ coordinates S95

5.0 AFA-TCNQ coordinates S147

6.0 P6*p*PP6-TCNQ coordinates S186

7.0 AAA-TCNQ coordinates S217

8.0 P6P6P6-TCNQ coordinates S256

The default integration grid in Gaussian 16, Ultrafine, was used.

Optimized Geometry Coordinates

1.0 TCNQ coordinates

Electronic Energy (EE): -678.791139 Hartree

EE + Thermal Free Energy Correction: -678.704922 Hartree

E (Thermal): 88.785 kcal/mol

Entropy (S): 118.313 cal/mol-kelvin

Imaginary frequencies: 0

0	1		
C	1.42017900	0.00000100	0.00017300
C	0.67560600	-1.23756500	0.00006100
H	1.22146200	-2.17252400	0.00005300
C	-0.67561400	-1.23756700	0.00025300
H	-1.22147600	-2.17252100	0.00022500
C	0.67560900	1.23757000	0.00006700
H	1.22147100	2.17252500	-0.00003800
C	-0.67561300	1.23756900	0.00022700
H	-1.22147500	2.17252500	0.00017200
C	-1.42018600	0.00000200	0.00043300
C	-2.80397600	0.00000100	0.00021000
C	2.80397400	0.00000100	0.00005700
C	-3.55028700	1.21030200	-0.00013800
C	-3.55028600	-1.21030000	-0.00009600
C	3.55029100	1.21029700	-0.00012200
C	3.55028400	-1.21030000	-0.00007500
N	-4.14387300	2.20213100	-0.00026400
N	-4.14386500	-2.20213300	-0.00031600
N	4.14386900	-2.20213000	-0.00019900
N	4.14388700	2.20212100	-0.00017900

2.0 A*p*PA-TCNQ coordinates

A*p*PA

Electronic Energy (EE): -3645.656408 Hartree

EE + Thermal Free Energy Correction: -3644.811767 Hartree

E (Thermal): 616.770 kcal/mol

Entropy (S): 292.947 cal/mol-kelvin

Imaginary frequencies: 0

0	1		
S	-10.3869	0.77324	3.100204
S	-8.32666	0.388621	-3.07339
C	-10.2701	2.547578	2.578551
H	-11.2733	2.970085	2.512678
H	-9.75858	3.018922	3.419964
C	-9.51456	2.71432	1.294733
C	-10.1842	2.998525	0.1009
C	-9.53611	2.867002	-1.12025
H	-10.0917	3.005543	-2.0409
C	-8.21053	2.445177	-1.19295
C	-7.48829	2.295836	0.014241
C	-8.15309	2.43735	1.242011
H	-7.60828	2.232345	2.155526
C	-7.62919	2.009592	-2.50191
H	-6.54384	1.940057	-2.46545
H	-7.90414	2.691589	-3.30832
C	-11.3699	-0.00577	1.740885
H	-12.2294	-0.46845	2.231121
H	-11.7479	0.798646	1.106199
C	-10.6487	-1.05897	0.880382
C	-11.6982	-1.7267	-0.03623
H	-12.1807	-0.96421	-0.65884
H	-12.4822	-2.19081	0.573204
C	-11.0313	-2.78744	-0.92944
H	-11.7904	-3.24732	-1.57017
C	-9.95898	-2.12203	-1.80695
H	-9.48779	-2.86984	-2.45595
H	-10.4181	-1.37176	-2.45747
C	-8.88967	-1.45051	-0.92389
C	-9.57288	-0.41984	-0.01173
H	-8.82303	0.05505	0.620545
H	-10.0257	0.357732	-0.62725

C	-9.99121	-2.15039	1.747007
H	-10.7472	-2.6266	2.382837
H	-9.25161	-1.6924	2.410091
C	-9.31747	-3.20321	0.851526
H	-8.8425	-3.96241	1.480934
C	-10.3758	-3.8625	-0.04754
H	-11.1329	-4.36089	0.567701
H	-9.91289	-4.63134	-0.67573
C	-8.24609	-2.52958	-0.02458
H	-7.74513	-3.28115	-0.64543
H	-7.47875	-2.06939	0.608236
C	-7.77846	-0.80814	-1.77258
H	-7.23746	-1.5911	-2.30878
H	-7.05872	-0.29627	-1.13278
H	-11.2351	3.265034	0.126723
C	-6.12315	1.915461	0.008339
C	-4.96029	1.561002	0.015328
C	-3.66644	1.16341	0.011972
C	-2.50586	0.80161	0.005193
C	-1.15582	0.38213	-0.0056
C	1.534949	-0.45119	-0.02887
C	-0.46455	0.223472	-1.22252
C	-0.47704	0.115493	1.199357
C	0.843516	-0.29329	1.187957
C	0.85578	-0.1856	-1.23393
H	-0.98054	0.426453	-2.15225
H	-1.00268	0.23517	2.138087
H	1.359629	-0.49588	2.117703
H	1.381411	-0.30525	-2.17268
C	2.886346	-0.8662	-0.0418
C	4.049248	-1.22025	-0.05666
C	5.346859	-1.60469	-0.07913
C	6.514654	-1.94104	-0.11193
S	10.81221	-1.05641	3.0499
S	8.705476	-0.06647	-2.98178
C	10.66384	-2.78606	2.398056
H	11.65797	-3.22278	2.299006
H	10.13838	-3.3113	3.197541
C	9.911206	-2.82537	1.103634
C	10.59009	-2.97321	-0.1103
C	9.946324	-2.72031	-1.31373
H	10.50692	-2.75568	-2.24101
C	8.614091	-2.31435	-1.34873
C	7.885592	-2.29902	-0.1367
C	8.548187	-2.55529	1.073625

H	7.997785	-2.45268	2.00091
C	8.036141	-1.7543	-2.6108
H	6.949014	-1.7123	-2.58466
H	8.337723	-2.33823	-3.48192
C	11.67159	-0.15932	1.681601
H	12.61664	0.195534	2.096926
H	11.90431	-0.87782	0.893473
C	10.87129	1.024578	1.114919
C	11.81922	1.972345	0.357911
H	12.4284	1.378533	-0.33643
H	12.51583	2.434662	1.066073
C	11.09964	3.057158	-0.46349
H	11.86729	3.586515	-1.03879
C	10.12671	2.38785	-1.4406
H	9.662862	3.140412	-2.08955
H	10.66511	1.689727	-2.0882
C	9.039893	1.635362	-0.65761
C	9.707505	0.557863	0.22271
H	8.951192	0.077941	0.845255
H	10.09144	-0.209	-0.45411
C	9.080065	3.584989	1.068215
H	8.465398	4.433796	1.384294
C	10.34704	4.111571	0.371327
H	11.01952	4.557237	1.111934
H	10.0602	4.920758	-0.31092
C	8.234156	2.670863	0.166258
H	7.687172	3.30026	-0.54618
H	7.47771	2.154299	0.766879
C	8.021088	0.977026	-1.61735
H	7.450226	1.760648	-2.11873
H	7.311303	0.36762	-1.05739
H	11.64283	-3.23367	-0.10645
H	10.46903	1.561895	1.974445
H	9.34545	3.060436	1.986131

A*p*PA-TCNQ-1

Electronic Energy (EE): -4324.484405 Hartree

EE + Thermal Free Energy Correction: -4323.527065 Hartree

E (Thermal): 707.346 kcal/mol

Entropy (S): 359.545 cal/mol-kelvin

Imaginary frequencies: 0

0	1		
S	8.67685200	2.00330900	2.84069400
S	8.58626200	-1.60960400	-2.57743000
C	9.49454600	0.42465100	3.35782300
H	10.55281600	0.60891900	3.54648300
H	9.01442400	0.20083600	4.31234200
C	9.29749900	-0.67659300	2.35959700
C	10.34513500	-1.08334900	1.52665400
C	10.10143600	-1.88566300	0.41988700
H	10.90886100	-2.11446800	-0.26636200
C	8.81210600	-2.30957000	0.10622400
C	7.77716000	-2.02623200	1.02699200
C	8.03279500	-1.20974900	2.14140700
H	7.20190700	-0.91596800	2.77108700
C	8.51423100	-2.89178900	-1.24032700
H	7.54465000	-3.38588400	-1.26896700
H	9.27679300	-3.61112100	-1.54338600
C	9.57081600	2.46208800	1.28847300
H	9.95442700	3.47042700	1.45992900
H	10.43133900	1.79620600	1.19230700
C	8.74547800	2.44664400	-0.01095100
C	9.60514500	3.07078900	-1.13271600
H	10.53517200	2.50087700	-1.24371500
H	9.88395200	4.09545600	-0.86075800
C	8.83067300	3.07333700	-2.46250100
H	9.45633500	3.51880600	-3.24258100
C	8.47628700	1.63016500	-2.85571100
H	7.93384300	1.62227200	-3.80853200
H	9.38966100	1.04468200	-2.99794600
C	7.61253200	0.97692000	-1.76017900
C	8.37995900	1.01409300	-0.42934800
H	7.76704100	0.56445400	0.35118700
H	9.28880800	0.41888400	-0.52282800
C	7.44824200	3.26677500	0.12857100
H	7.68806900	4.29679100	0.41923600
H	6.83095100	2.83820000	0.92314800
C	6.67443100	3.26077200	-1.20031400
H	5.74960500	3.83479900	-1.08183300
C	7.53957800	3.89225800	-2.30239200

H	7.77902000	4.92971200	-2.04411300
H	6.98989800	3.91343200	-3.24976600
C	6.32483400	1.81306000	-1.58654200
H	5.74116800	1.79722500	-2.51307400
H	5.70675800	1.36356400	-0.80355200
C	7.20617500	-0.45681000	-2.14005700
H	6.55181200	-0.43502800	-3.01226500
H	6.63857800	-0.91701900	-1.33156700
H	11.34841200	-0.71580400	1.71267400
C	6.45037200	-2.46364400	0.80584900
C	5.28483600	-2.77413300	0.65309700
C	3.96702200	-3.02334200	0.50483400
C	2.76520000	-3.17672900	0.38768400
C	1.36045500	-3.23016300	0.30289000
C	-1.44824500	-3.14975900	0.13634500
C	0.58419900	-2.86704100	1.42246700
C	0.70340300	-3.58837100	-0.89305800
C	-0.67463500	-3.55034800	-0.97324500
C	-0.79202600	-2.82650600	1.34108800
H	1.08559800	-2.60171500	2.34410400
H	1.29513000	-3.87721900	-1.75231500
H	-1.17586400	-3.81121800	-1.89655000
H	-1.38257900	-2.52988800	2.19842200
C	-2.85015600	-3.03591400	0.04380700
C	-4.05410400	-2.88220000	-0.03705500
C	-5.38369700	-2.67688100	-0.15186900
C	-6.57432200	-2.46002800	-0.26647900
S	-9.61227600	0.89543800	-3.27991600
S	-8.39022200	-0.59422100	2.89045300
C	-10.21164600	-0.85475100	-3.17057400
H	-11.29689000	-0.87511600	-3.27505500
H	-9.77502600	-1.32557500	-4.05302900
C	-9.76748300	-1.51387300	-1.90086300
C	-10.65800600	-1.67440800	-0.83336700
C	-10.19496000	-2.00941200	0.43131500
H	-10.88555200	-2.04122200	1.26627400
C	-8.83690700	-2.20353100	0.67376200
C	-7.95370100	-2.19013300	-0.43035400
C	-8.43060100	-1.83656500	-1.70288800
H	-7.72122400	-1.73539300	-2.51509700
C	-8.32453800	-2.25766400	2.07859800
H	-7.30979300	-2.64731500	2.13391400
H	-8.96414400	-2.87173500	2.71465600
C	-10.33244700	1.68431100	-1.77201700
H	-10.99347500	2.47640600	-2.12887400

H	-10.94960100	0.94438800	-1.25894900
C	-9.28329000	2.26955300	-0.81346000
C	-9.96204900	3.27114400	0.13888800
H	-10.85392000	2.79577900	0.56855300
H	-10.31557500	4.13674400	-0.43247600
C	-9.07268600	3.73414700	1.30715500
H	-9.70172300	4.34000800	1.96886500
C	-8.57979700	2.50783800	2.08374000
H	-8.00560600	2.82106600	2.96377800
H	-9.43213900	1.92445200	2.44386900
C	-7.70136400	1.63559600	1.17382100
C	-8.53439200	1.17171100	-0.03883100
H	-7.89372700	0.62142000	-0.72958300
H	-9.27580600	0.46447800	0.34019300
C	-6.76102500	3.84097600	0.15200700
H	-5.84411300	4.43927700	0.16138300
C	-7.86780300	4.60379300	0.89998300
H	-8.20280000	5.45638800	0.29957800
H	-7.44410800	5.02201500	1.82089500
C	-6.45691700	2.46442500	0.76646400
H	-5.85407300	2.61249000	1.66902500
H	-5.84572600	1.88270900	0.07061700
C	-7.15817000	0.40512900	1.93828600
H	-6.42603000	0.73178300	2.67650900
H	-6.64380000	-0.26871200	1.25189100
H	-11.71385700	-1.47865400	-0.98502400
H	-8.57165700	2.81644600	-1.43285000
H	-7.02397800	3.73013700	-0.90005500
C	-0.44049800	0.56827700	1.38790500
C	0.89939900	0.47770000	1.22891600
C	1.47368100	0.08556200	-0.03499500
C	-1.34148300	0.29877300	0.29319800
C	-0.76633300	-0.07812000	-0.97221100
C	0.57234200	-0.18825700	-1.12520800
C	2.84497200	-0.03161500	-0.20099000
C	-2.71519600	0.40466100	0.44867700
C	3.39911900	-0.42010900	-1.45053800
C	3.76004300	0.22925000	0.85333300
C	-3.61111200	0.26922400	-0.64538300
C	-3.29537000	0.68398600	1.71442200
N	-4.34477300	0.19688200	-1.53612400
N	-3.75425300	0.90028200	2.75345200
N	3.82116500	-0.73246800	-2.48024500
N	4.50778700	0.44045900	1.70965600
H	-1.43037200	-0.29239100	-1.79956200

H	0.98763300	-0.49277100	-2.07671800
H	1.56411600	0.69443100	2.05581700
H	-0.85781800	0.85725800	2.34423600

A*p*PA-TCNQ-2

Electronic Energy (EE): -4324.484311 Hartree

EE + Thermal Free Energy Correction: -4323.527024 Hartree

E (Thermal): 707.386 kcal/mol

Entropy (S): 359.791 cal/mol-kelvin

Imaginary frequencies: 0

S	10.87684900	2.29181500	-1.34514500
S	7.43246900	-1.86729500	2.29559100
C	10.94560400	3.04231800	0.34723900
H	11.96230400	2.96678700	0.73433800
H	10.73286800	4.09639300	0.15855800
C	9.95132100	2.42593800	1.28465900
C	10.36281400	1.54433100	2.28878500
C	9.43811200	0.75726200	2.96162600
H	9.78123600	0.01607100	3.67450400
C	8.07956800	0.81419700	2.65593300
C	7.64529700	1.80478100	1.74509500
C	8.58830100	2.59885600	1.07427800
H	8.24264600	3.28877600	0.31406700
C	7.14984000	-0.24857800	3.15330600
H	6.10134200	0.02286700	3.05493800
H	7.34444800	-0.48954800	4.19980300
C	11.36862500	0.53231300	-1.05274100
H	12.22499200	0.35785300	-1.70793600
H	11.72280800	0.45695500	-0.02228800
C	10.29676100	-0.53949000	-1.32082500
C	10.97453300	-1.92556100	-1.23590900
H	11.42792100	-2.05341900	-0.24587000
H	11.78322900	-1.98947000	-1.97320100
C	9.94474900	-3.04084700	-1.48872200
H	10.44451400	-4.01277500	-1.42428200
C	8.83436300	-2.96724200	-0.42868900
H	8.10238100	-3.76617900	-0.59598400
H	9.25651200	-3.11719800	0.56963500

C	8.13108200	-1.59763500	-0.48912200
C	9.17523600	-0.49195100	-0.27109700
H	8.68531700	0.48006000	-0.32020200
H	9.60530900	-0.59542600	0.72554500
C	9.67421500	-0.38501600	-2.72195300
H	10.46068200	-0.42931300	-3.48523000
H	9.19865700	0.59671000	-2.80275100
C	8.63801200	-1.49424300	-2.96557200
H	8.19309800	-1.36220700	-3.95691900
C	9.32939100	-2.86488400	-2.88639600
H	10.10728300	-2.93876600	-3.65453300
H	8.60616000	-3.66427500	-3.08030700
C	7.53007800	-1.41448500	-1.90062900
H	6.76925200	-2.17768100	-2.08831900
H	7.02394300	-0.44413700	-1.95905000
C	6.98704500	-1.51135800	0.53618500
H	6.22019600	-2.24240700	0.27478100
H	6.52055000	-0.52623800	0.50498100
H	11.42050900	1.43111400	2.50030400
C	6.27375800	1.95244200	1.41930900
C	5.10759800	2.07957500	1.10192900
C	3.80401700	2.19630400	0.76191100
C	2.62773000	2.28382900	0.46727800
C	1.25740000	2.34709600	0.13193400
C	-1.48294600	2.36981600	-0.51367000
C	0.30676900	2.82762100	1.05564400
C	0.81426900	1.90578600	-1.13016500
C	-0.52943600	1.91736200	-1.44696100
C	-1.03802500	2.83768400	0.74047100
H	0.64425300	3.17709100	2.02301100
H	1.54163200	1.54550500	-1.84631200
H	-0.86638900	1.56613300	-2.41366100
H	-1.76704800	3.19244800	1.45778800
C	-2.85734900	2.30932600	-0.82131100
C	-4.04008500	2.18461500	-1.07893200
C	-5.35101200	2.00713600	-1.35027900
C	-6.52622800	1.81393500	-1.59506100
S	-11.18170700	2.31897000	1.16075800
S	-7.71736100	-2.08549000	-2.03630400
C	-11.19779500	2.98053200	-0.57059200
H	-12.20781100	2.91246700	-0.97578100
H	-10.95371400	4.03613600	-0.43929500
C	-10.20473100	2.27475300	-1.44218100
C	-10.62282900	1.30160700	-2.35676200
C	-9.70438300	0.44852400	-2.95172800

H	-10.05272200	-0.35777400	-3.58702800
C	-8.34318400	0.53680600	-2.66534300
C	-7.90216900	1.61206500	-1.85932600
C	-8.84151700	2.46082400	-1.25152400
H	-8.49013800	3.22072400	-0.56423600
C	-7.42129500	-0.57346700	-3.06276300
H	-6.36976500	-0.30348900	-2.99886200
H	-7.62533600	-0.91844300	-4.07770400
C	-11.57830800	0.52873800	0.93048600
H	-12.51426200	0.35341700	1.46417500
H	-11.76417300	0.35333000	-0.13081100
C	-10.49141100	-0.42438800	1.45228900
C	-11.08912600	-1.83184500	1.63245800
H	-11.62645500	-2.10385100	0.71416900
H	-11.83470900	-1.81222500	2.43514400
C	-10.04591300	-2.93061200	1.90449700
H	-10.57853000	-3.88812200	1.89359600
C	-9.00877000	-2.93199700	0.77554800
H	-8.30092900	-3.75847600	0.91196500
H	-9.50328300	-3.08506000	-0.18800600
C	-8.25082200	-1.59534700	0.76550700
C	-9.25724300	-0.44829500	0.53424700
H	-8.73903000	0.50913300	0.60742100
H	-9.60844600	-0.54036000	-0.49615600
C	-8.34325300	-1.64462000	3.36675000
H	-7.67928000	-1.80447300	4.22207500
C	-9.32831400	-2.82174600	3.26358100
H	-10.06220200	-2.76561800	4.07467500
H	-8.77427700	-3.75630300	3.41324600
C	-7.48397900	-1.46516700	2.10460500
H	-6.70359600	-2.23597800	2.10852500
H	-6.96034800	-0.50645000	2.14476700
C	-7.17518000	-1.57327600	-0.34629200
H	-6.37363600	-2.26540400	-0.08142100
H	-6.73252200	-0.57989400	-0.42384300
H	-11.68127900	1.17737900	-2.55783100
H	-10.19876500	-0.05022500	2.43393100
H	-8.88073200	-0.72182400	3.58607000
C	0.54403400	-1.50530000	-0.97603800
C	-0.79987900	-1.40367200	-1.07743500
C	-1.59454500	-0.91136100	0.02208900
C	1.23003500	-1.13234300	0.23775200
C	0.43437200	-0.65341400	1.33971500
C	-0.90933400	-0.54856500	1.23675500
C	-2.96927900	-0.77798300	-0.08847900

C	2.60805600	-1.23565800	0.34333900
C	-3.75473000	-0.31000100	0.99905400
C	-3.65092300	-1.07677800	-1.29747000
C	3.29023500	-0.95599300	1.55726900
C	3.40308200	-1.65151600	-0.75776600
N	3.83289100	-0.74838000	2.55618400
N	4.03109200	-1.98810300	-1.66832300
N	-4.36895600	0.07035100	1.90120600
N	-4.18410100	-1.30442500	-2.29749100
H	0.93673000	-0.36240400	2.25303600
H	-1.49140300	-0.17204600	2.06746800
H	-1.30191400	-1.68999400	-1.99299100
H	1.12759500	-1.87442700	-1.80987300

A*p*PA-TCNQ-3

Electronic Energy (EE): -4324.482928 Hartree

EE + Thermal Free Energy Correction: -4323.526861 Hartree

E (Thermal): 707.446 kcal/mol

Entropy (S): 362.561 cal/mol-kelvin

Imaginary frequencies: 0

S	11.74081900	-0.59596500	2.91455600
S	9.70094100	0.35994800	-3.20145300
C	11.84750900	-2.28561700	2.16138400
H	12.89546500	-2.57154200	2.06571300
H	11.39021500	-2.92368500	2.91998500
C	11.12950300	-2.36873400	0.84820800
C	11.83896500	-2.40647700	-0.35596600
C	11.19042300	-2.19180900	-1.56506000
H	11.76716800	-2.13613300	-2.48139500
C	9.82323000	-1.92969700	-1.61490400
C	9.07830300	-2.03482000	-0.41687200
C	9.74478300	-2.25577100	0.79832400
H	9.17038500	-2.24502500	1.71662700
C	9.20287100	-1.39523700	-2.86812200
H	8.11694400	-1.46298300	-2.84924100
H	9.56581000	-1.92396000	-3.75111500
C	12.63542000	0.46874300	1.69518400
H	13.42747300	0.96111200	2.26376200
H	13.11468800	-0.19301300	0.97051300

C	11.79991300	1.53344000	0.96187600
C	12.76920600	2.43382400	0.16342600
H	13.34339400	1.82124800	-0.54150400
H	13.48809500	2.90242700	0.84534900
C	11.98735800	3.51711500	-0.59993200
H	12.69098000	4.14274300	-1.15827400
C	11.00871800	2.85161200	-1.58053900
H	10.45656900	3.61765700	-2.13796400
H	11.55900400	2.25231500	-2.31187600
C	10.02041400	1.94925100	-0.81655000
C	10.81467200	0.89521000	-0.02958000
H	10.12216300	0.25688900	0.51885200
H	11.36159900	0.26345100	-0.72961100
C	11.01084200	2.41711200	1.94716300
H	11.69993900	2.89122500	2.65634800
H	10.32626200	1.79254700	2.52835300
C	10.22312000	3.49202600	1.18005600
H	9.65631400	4.10121200	1.89119100
C	11.20223500	4.38296900	0.39846300
H	11.89015700	4.88113900	1.09035600
H	10.65599400	5.16832600	-0.13521600
C	9.24626500	2.81854100	0.19956600
H	8.66451400	3.58132400	-0.33050800
H	8.53414900	2.19303300	0.74975800
C	9.00114100	1.29650500	-1.76671600
H	8.37102400	2.07254400	-2.20703800
H	8.34609800	0.62024200	-1.21598600
H	12.91452000	-2.54434500	-0.33938600
C	7.67548000	-1.83644200	-0.40696500
C	6.47473600	-1.64788000	-0.37982300
C	5.13806600	-1.43943700	-0.35727900
C	3.93727000	-1.25037800	-0.33748500
C	2.54131700	-1.03372700	-0.31079400
C	-0.23261900	-0.60164700	-0.25569600
C	1.86965900	-0.85728600	0.91547000
C	1.79954300	-0.99792700	-1.50850000
C	0.43481800	-0.78737100	-1.48262700
C	0.50535500	-0.64471800	0.94410800
H	2.43454800	-0.89704100	1.83766100
H	2.31058200	-1.14617500	-2.45082800
H	-0.13595800	-0.78232000	-2.40140800
H	-0.01095000	-0.53091900	1.88770500
C	-1.62891400	-0.41545000	-0.22401200
C	-2.84119400	-0.30717200	-0.18757500
C	-4.18696200	-0.28368200	-0.12453900

C	-5.39970800	-0.34579000	-0.03866300
S	-9.75758400	1.17578500	-2.88275600
S	-7.07641500	1.82232800	2.97005800
C	-9.80701900	-0.55866000	-2.23419800
H	-10.83832700	-0.85645900	-2.04875600
H	-9.42980300	-1.16016400	-3.06223100
C	-8.96402600	-0.69607800	-1.00537700
C	-9.55741600	-0.77714800	0.25993400
C	-8.79891800	-0.62090200	1.41271000
H	-9.28766200	-0.61626600	2.37945800
C	-7.42792100	-0.39544300	1.34601700
C	-6.79993400	-0.45954800	0.07716900
C	-7.58083400	-0.60461900	-1.08183300
H	-7.09326000	-0.56501200	-2.04791500
C	-6.68206400	0.05920500	2.55949400
H	-5.60525800	-0.05752500	2.45318500
H	-7.00396200	-0.48160000	3.45089600
C	-10.38096900	2.17878700	-1.45951100
H	-11.29199700	2.66697900	-1.81004800
H	-10.66150700	1.49669200	-0.65474200
C	-9.38367800	3.23422800	-0.95443200
C	-10.13040300	4.29727000	-0.12833100
H	-10.76258800	3.78743100	0.61062200
H	-10.80692200	4.85872200	-0.78193000
C	-9.20671900	5.26388600	0.63485800
H	-9.84749100	5.88801100	1.26725400
C	-8.26399500	4.45717900	1.53541000
H	-7.65232100	5.13232500	2.14570500
H	-8.84305500	3.83412600	2.22328800
C	-7.35534500	3.56946600	0.67121600
C	-8.23255600	2.60291900	-0.15179900
H	-7.60040900	2.02825500	-0.82997700
H	-8.66798600	1.89280000	0.55516300
C	-7.25792600	5.52254400	-1.04945900
H	-6.55691900	6.28065600	-1.41246900
C	-8.38082000	6.21257400	-0.25539300
H	-9.03942900	6.75587900	-0.94102500
H	-7.93190900	6.96605900	0.40267000
C	-6.48034500	4.49034000	-0.21612900
H	-5.79603100	5.03021700	0.44945600
H	-5.85342100	3.87892600	-0.87433700
C	-6.36462500	2.76838300	1.54914500
H	-5.65170100	3.46050400	2.00052500
H	-5.79271100	2.07052900	0.93660100
H	-10.62970700	-0.91587400	0.33825700

H	-8.97488100	3.71875000	-1.84163300
H	-7.66409000	5.05040100	-1.94409400
C	-6.32225800	-3.51396300	1.48598500
C	-4.97442000	-3.40938300	1.44146200
C	-4.26284600	-3.46538900	0.18818800
C	-7.09833100	-3.67299000	0.28036900
C	-6.38968500	-3.68387300	-0.97595200
C	-5.04157500	-3.58891800	-1.01944500
C	-2.87887800	-3.40949100	0.14483200
C	-8.47578100	-3.81530700	0.32637400
C	-2.16540500	-3.47967300	-1.08214600
C	-2.10042100	-3.26295600	1.32445900
C	-9.24778000	-3.93453600	-0.86012200
C	-9.18133900	-3.83258300	1.55900800
N	-9.85890600	-4.01553500	-1.83856100
N	-9.73932300	-3.84041400	2.57170000
N	-1.59084000	-3.53186600	-2.08404400
N	-1.47410100	-3.13027400	2.28718400
H	-6.95962300	-3.78056600	-1.89078500
H	-4.52373800	-3.60691600	-1.96988500
H	-4.40614000	-3.29103700	2.35523800
H	-6.84130400	-3.48263600	2.43508800

A*p*PA-TCNQ-4

Electronic Energy (EE): -4324.481344 Hartree

EE + Thermal Free Energy Correction: -4323.525279 Hartree

E (Thermal): 707.272 kcal/mol

Entropy (S): 361.980 cal/mol-kelvin

Imaginary frequencies: 0

0	1		
S	-10.71301400	0.89793000	2.88027200
S	-7.75593000	0.52473700	-2.89178500
C	-10.71700700	2.65021800	2.27883700
H	-11.73954500	2.94727300	2.04330900
H	-10.39457300	3.21414100	3.15618700
C	-9.79946700	2.85541700	1.11120200
C	-10.30656300	3.00460300	-0.18389500
C	-9.47096500	2.90267000	-1.28788600
H	-9.89460400	2.92453700	-2.28549200

C	-8.10749300	2.65301800	-1.14381700
C	-7.56733900	2.66110200	0.16405200
C	-8.42319900	2.75550200	1.27357100
H	-8.00029100	2.66414400	2.26660800
C	-7.29062200	2.22567200	-2.32459600
H	-6.21988700	2.25356000	-2.13400900
H	-7.49906800	2.84270000	-3.20028400
C	-11.41308900	-0.03952000	1.44811200
H	-12.27652400	-0.57798800	1.84527100
H	-11.78611100	0.69260700	0.72849600
C	-10.47303600	-1.03463600	0.74543900
C	-11.30583500	-1.82683000	-0.28725300
H	-11.76100200	-1.13203100	-1.00289100
H	-12.12380800	-2.35213100	0.21928000
C	-10.41525000	-2.83705400	-1.03161200
H	-11.02347200	-3.38598700	-1.75771700
C	-9.29325300	-2.08869400	-1.76945700
H	-8.66084400	-2.80122900	-2.31252500
H	-9.72137900	-1.40595100	-2.50932400
C	-8.43697200	-1.29194500	-0.76630300
C	-9.34418600	-0.31246400	-0.00544400
H	-8.74509100	0.25279600	0.70901200
H	-9.77435100	0.39772100	-0.71153200
C	-9.84919000	-2.03168100	1.74101800
H	-10.64203700	-2.56420400	2.28023900
H	-9.26067900	-1.48535600	2.48377500
C	-8.95368200	-3.03433500	0.99389800
H	-8.50589800	-3.72499700	1.71529100
C	-9.80006500	-3.81868200	-0.02119400
H	-10.59047100	-4.37407700	0.49586000
H	-9.17811300	-4.55340600	-0.54414900
C	-7.83382700	-2.27833300	0.25795100
H	-7.17901800	-2.99169800	-0.25617500
H	-7.21092800	-1.73648000	0.97468700
C	-7.28331700	-0.55168100	-1.46535800
H	-6.57557100	-1.27937000	-1.86939000
H	-6.74212700	0.06510400	-0.74722800
H	-11.37322400	3.13746300	-0.32808600
C	-6.17742800	2.51217700	0.38276300
C	-4.98634000	2.38731300	0.59206100
C	-3.66167800	2.21289200	0.78435100
C	-2.47021500	2.01166600	0.92880500
C	-1.09757400	1.70360300	1.00307200
C	1.60335200	0.91428700	1.03650700
C	-0.27255000	1.92151200	-0.11911700

C	-0.53410900	1.12571000	2.16001400
C	0.79134500	0.73861400	2.17642900
C	1.05126000	1.53511300	-0.10244900
H	-0.69967200	2.37670500	-1.00345700
H	-1.16145000	0.96827600	3.02802600
H	1.21510600	0.27405900	3.05757400
H	1.67608000	1.68348800	-0.97376400
C	2.92809900	0.43652400	0.99468300
C	4.04319100	-0.03435600	0.87119900
C	5.25852300	-0.59369400	0.69125900
C	6.32730500	-1.13136000	0.47662900
S	11.18637800	-1.06016600	2.83008600
S	8.28759500	0.30201800	-2.78239800
C	10.61964600	-2.73257000	2.26447900
H	11.48656300	-3.34242500	2.00891600
H	10.15568600	-3.15595700	3.15700400
C	9.65817900	-2.63090800	1.12044700
C	10.07375200	-2.90167000	-0.18779300
C	9.28725800	-2.53483200	-1.27107400
H	9.66004700	-2.67748900	-2.27890900
C	8.06776100	-1.88493800	-1.09191500
C	7.58183600	-1.73566600	0.22821900
C	8.38413900	-2.11309900	1.31634100
H	8.02946700	-1.91364400	2.32010200
C	7.38957200	-1.23050400	-2.25445500
H	6.34597000	-0.99466000	-2.06098500
H	7.42890600	-1.86046600	-3.14427300
C	11.94639000	-0.33324600	1.31030700
H	12.99687500	-0.15802800	1.54962100
H	11.90754600	-1.08110300	0.51607100
C	11.28823200	0.97783800	0.85077600
C	12.24731800	1.73455900	-0.08602100
H	12.60981800	1.03802400	-0.85361500
H	13.12761300	2.06119000	0.47868700
C	11.60607700	2.93249800	-0.80939100
H	12.34628800	3.31010600	-1.52348800
C	10.37419500	2.45252700	-1.58507500
H	9.94952100	3.27664200	-2.17082900
H	10.65660100	1.66604400	-2.29094900
C	9.32009300	1.91444300	-0.60509600
C	9.92287700	0.73304900	0.18417500
H	9.21163100	0.40166800	0.94195300
H	10.03882000	-0.09209100	-0.52247100
C	10.01114500	3.82527400	1.02357500
H	9.62463000	4.77300700	1.41170000

C	11.21239300	4.10893500	0.10472000
H	12.07194100	4.42491500	0.70528600
H	10.96301700	4.95566200	-0.54596200
C	8.87059500	3.08140200	0.30921900
H	8.33139300	3.79981300	-0.32019100
H	8.14893400	2.71349500	1.04671200
C	8.04907400	1.45458800	-1.35762900
H	7.55265100	2.33034900	-1.77973400
H	7.34696500	0.98780400	-0.66637100
H	11.04465500	-3.35448700	-0.35674500
H	11.14441800	1.58019000	1.74848600
H	10.33221000	3.26173400	1.89969300
C	0.31724000	-1.24505400	-1.83079400
C	-0.97895700	-0.85862700	-1.84414200
C	-1.84191400	-1.10606700	-0.71533200
C	0.88233600	-1.91843600	-0.68731700
C	0.01030800	-2.19474200	0.42606800
C	-1.28533800	-1.81014700	0.41192700
C	-3.15833500	-0.67067000	-0.70028200
C	2.21928300	-2.28203700	-0.64778300
C	-4.01527000	-0.95214700	0.39719900
C	-3.70294800	0.10205500	-1.75932100
C	2.76464800	-2.98874000	0.45807000
C	3.11439300	-1.96596600	-1.70414800
N	3.18875700	-3.57407700	1.36008500
N	3.84330500	-1.69599800	-2.55998300
N	-4.69670600	-1.19787600	1.29792300
N	-4.12625300	0.75639400	-2.61346700
H	0.41831100	-2.70411300	1.28947600
H	-1.92187900	-2.00901200	1.26433000
H	-1.38772700	-0.34820000	-2.70710500
H	0.95471100	-1.04673700	-2.68327100

A*p*PA-TCNQ-5

Electronic Energy (EE): -4324.477474 Hartree

EE + Thermal Free Energy Correction: -4323.524240 Hartree

E (Thermal): 707.255 kcal/mol

Entropy (S): 367.881 cal/mol-kelvin

Imaginary frequencies: 0

0	1		
S	-10.71879900	1.65740600	-2.83285300
S	-8.30368200	-0.88616000	2.65832000
C	-10.57208500	-0.14680500	-3.22903700
H	-11.56953400	-0.57755000	-3.32204100
H	-10.10980700	-0.14591300	-4.21797500
C	-9.74333200	-0.88378100	-2.22082500
C	-10.34168300	-1.72120000	-1.27411200
C	-9.62581000	-2.17138600	-0.17258100
H	-10.12752900	-2.75018400	0.59453200
C	-8.29889500	-1.79795000	0.02896700
C	-7.65055900	-1.07144200	-0.99816000
C	-8.38130400	-0.62959700	-2.11196500
H	-7.88700300	-0.00412400	-2.84521000
C	-7.64209000	-2.02717100	1.35416600
H	-6.55940200	-1.94198900	1.30793700
H	-7.88122900	-3.01496600	1.75093500
C	-11.62162200	1.66670500	-1.21833400
H	-12.50869000	2.28212800	-1.38394200
H	-11.96127600	0.64678400	-1.02538300
C	-10.85150700	2.20325800	0.00175000
C	-11.84406700	2.32178800	1.17999700
H	-12.28714600	1.34074900	1.38754700
H	-12.66415600	2.99653700	0.90862900
C	-11.12521000	2.84790900	2.43469700
H	-11.84472300	2.92453100	3.25607900
C	-10.00169300	1.87636300	2.83038700
H	-9.49232700	2.23818200	3.73152300
H	-10.42017100	0.89382400	3.06743000
C	-8.98735500	1.73798000	1.67893300
C	-9.72364800	1.24804500	0.42255600
H	-9.01329700	1.15443500	-0.39860800
H	-10.13821600	0.25834900	0.61477900
C	-10.24778400	3.59440000	-0.27164700
H	-11.04082700	4.29407000	-0.56180000
H	-9.54935400	3.52983200	-1.11101600
C	-9.52181500	4.11193300	0.98105200
H	-9.08574700	5.09277700	0.76669500
C	-10.52469800	4.23192400	2.13982800
H	-11.31768300	4.94087900	1.87760800
H	-10.02562900	4.62187500	3.03362100
C	-8.39944000	3.13334900	1.37189500
H	-7.86266700	3.51230400	2.24916800

H	-7.67112900	3.05326100	0.55678600
C	-7.82896800	0.79840800	2.05868400
H	-7.25570500	1.24629300	2.87368100
H	-7.14874300	0.67253700	1.21562100
H	-11.39206200	-1.97303700	-1.37177200
C	-6.28805100	-0.71066500	-0.87929000
C	-5.12106600	-0.39209400	-0.76016400
C	-3.80961600	-0.11564100	-0.60015700
C	-2.61855600	0.07523900	-0.44042400
C	-1.22997800	0.17643000	-0.22580100
C	1.55100400	0.15205500	0.19923000
C	-0.34014600	0.40611600	-1.29570700
C	-0.70362900	-0.01418300	1.06822200
C	0.66005900	-0.02541800	1.27695400
C	1.02438900	0.39350300	-1.08688300
H	-0.74129100	0.56722900	-2.28815000
H	-1.38524800	-0.17728100	1.89310700
H	1.06149500	-0.19777100	2.26734500
H	1.70622400	0.54514100	-1.91387000
C	2.94023200	0.02996000	0.40117400
C	4.12771300	-0.17046900	0.57411200
C	5.43955800	-0.44721200	0.73329900
C	6.61070600	-0.75374500	0.84120700
S	10.98023400	1.51670500	3.05151400
S	8.69357800	-0.52275100	-2.64005100
C	10.83555500	-0.31228600	3.31995700
H	11.83179000	-0.74594600	3.41069900
H	10.34161000	-0.38577500	4.29038300
C	10.04402800	-0.96766600	2.23029100
C	10.68483100	-1.68892800	1.21715400
C	10.00300200	-2.04585900	0.06205700
H	10.53476800	-2.53239500	-0.74762100
C	8.66766400	-1.69536900	-0.12673000
C	7.98070100	-1.08969500	0.95131200
C	8.67886900	-0.73656600	2.11641600
H	8.15447600	-0.19794300	2.89617700
C	8.04528300	-1.81653700	-1.48246000
H	6.95899400	-1.77506800	-1.45791100
H	8.33247000	-2.74992000	-1.96899700
C	11.79081000	1.63955400	1.39518900
H	12.74266200	2.14791900	1.55959600
H	12.01071100	0.62924800	1.04522100
C	10.96231900	2.40483600	0.35042600
C	11.88009300	2.86427300	-0.79698500
H	12.47658400	2.00610600	-1.13389500

H	12.59058500	3.60868900	-0.42077400
C	11.12857900	3.41870600	-2.02047900
H	11.87544400	3.59896900	-2.80158800
C	10.13558300	2.36475300	-2.52310900
H	9.64697100	2.71137700	-3.44166000
H	10.66214500	1.43751600	-2.76705600
C	9.07768300	2.09042100	-1.44304500
C	9.78054200	1.56911600	-0.17171100
H	9.04707100	1.45181300	0.62713200
H	10.15497600	0.56994500	-0.40627700
C	9.14748700	4.63268400	-0.87894600
H	8.53330200	5.53114800	-0.99634500
C	10.38909700	4.74912700	-1.78062900
H	11.07719300	5.49522500	-1.36930600
H	10.07515400	5.12670200	-2.76114000
C	8.28604000	3.39952300	-1.19869600
H	7.71133400	3.60759400	-2.10944900
H	7.55328000	3.24206400	-0.39975400
C	8.03881200	1.05539000	-1.93583900
H	7.44916000	1.50211200	-2.73855300
H	7.34781200	0.79945200	-1.13232200
H	11.73920000	-1.92387000	1.31389000
H	10.57733800	3.29244600	0.85373300
H	9.44397500	4.61839100	0.16992300
C	1.06438000	-3.06509900	-1.25300400
C	-0.26359300	-3.04210000	-1.50489900
C	-1.22727800	-3.19616800	-0.44417400
C	1.56586900	-3.23693000	0.08700900
C	0.60241100	-3.41033400	1.14636900
C	-0.72578400	-3.39440000	0.89337200
C	-2.58773200	-3.14792500	-0.70492600
C	2.92731600	-3.22840400	0.34703300
C	-3.55295500	-3.27055800	0.32991600
C	-3.08548900	-2.97198400	-2.02424900
C	3.43324400	-3.33748800	1.66989900
C	3.88643900	-3.11163000	-0.69477300
N	3.82549500	-3.41907100	2.75432000
N	4.66305100	-3.02759500	-1.54682800
N	-4.33358700	-3.36562100	1.17728900
N	-3.47096100	-2.83428900	-3.10528200
H	0.96363900	-3.55208400	2.15704300
H	-1.43645800	-3.52362800	1.69990000
H	-0.62434300	-2.89431500	-2.51459500
H	1.77458800	-2.93620900	-2.05968600

A*p*PA-TCNQ-6

Electronic Energy (EE): -4324.479109 Hartree

EE + Thermal Free Energy Correction: -4323.524133 Hartree

E (Thermal): 707.316 kcal/mol

Entropy (S): 364.419 cal/mol-kelvin

Imaginary frequencies: 0

S	8.15262600	-0.14254200	2.75897400
S	6.94319300	2.28609700	-3.15908900
C	9.53293600	-0.83992000	1.73604000
H	10.47247800	-0.37183700	2.03139300
H	9.56142700	-1.88682000	2.04365400
C	9.27153000	-0.69203200	0.26711800
C	9.96803000	0.24232200	-0.50504500
C	9.51987300	0.58376700	-1.77524900
H	10.02498500	1.36638500	-2.33005300
C	8.35509200	0.02968700	-2.30301000
C	7.72302600	-1.00393500	-1.57674200
C	8.20427600	-1.36951900	-0.31030600
H	7.65509600	-2.10890500	0.25869600
C	7.71169400	0.63598900	-3.51220800
H	6.96022900	-0.02043200	-3.94821200
H	8.45031000	0.86880400	-4.28119100
C	8.19706900	1.66244600	2.35944400
H	8.33086500	2.17114200	3.31665700
H	9.09311700	1.84559900	1.76248200
C	6.96496500	2.24737100	1.64381800
C	7.10785100	3.78624800	1.63347000
H	8.03903400	4.06452400	1.12642900
H	7.17593500	4.15889400	2.66189500
C	5.90830200	4.43283500	0.91948400
H	6.02936900	5.52055700	0.92445800
C	5.84479900	3.93352400	-0.53239300
H	5.00044300	4.39990300	-1.05344700
H	6.75473500	4.21734400	-1.06993400
C	5.69056400	2.40150300	-0.56002100
C	6.87340000	1.76370700	0.18729900
H	6.76423700	0.67940900	0.18054200

H	7.80001100	2.00221000	-0.33598100
C	5.65398700	1.88376200	2.36869600
H	5.69382200	2.22882000	3.40853200
H	5.54433500	0.79500000	2.39436100
C	4.45657500	2.52410900	1.64776600
H	3.53064200	2.25684600	2.16775400
C	4.60988700	4.05338900	1.65019700
H	4.63327300	4.42374300	2.68061400
H	3.74740900	4.51657300	1.16063800
C	4.39732800	2.02293200	0.19435000
H	3.52497500	2.45108200	-0.31237200
H	4.28731800	0.93279700	0.16787900
C	5.56458700	1.87439700	-1.99889500
H	4.66556700	2.29618300	-2.45465400
H	5.45046500	0.79074500	-1.99703600
H	10.83174900	0.74738600	-0.08631400
C	6.48779400	-1.56377400	-1.98999600
C	5.36022000	-1.97353800	-2.18333600
C	4.05444800	-2.31251600	-2.27460200
C	2.86075100	-2.54339500	-2.26921800
C	1.45819600	-2.64512800	-2.12705800
C	-1.29916000	-2.75021600	-1.57151700
C	0.69463800	-1.46272500	-2.06369500
C	0.80735500	-3.88311200	-1.96764800
C	-0.55023200	-3.93376400	-1.69704900
C	-0.65752000	-1.51417100	-1.79195000
H	1.18937700	-0.50991900	-2.20232400
H	1.38273400	-4.79795800	-2.02807700
H	-1.03781500	-4.88832800	-1.54525800
H	-1.23702900	-0.60293800	-1.71687500
C	-2.64817000	-2.75461200	-1.14808000
C	-3.76844600	-2.63444800	-0.69282800
C	-4.99530300	-2.44342100	-0.15580100
C	-6.07090200	-2.20626900	0.35646600
S	-10.91064700	-0.76031200	-1.51315500
S	-6.16330900	1.39836600	2.28298800
C	-10.96186700	-1.96266000	-0.10220300
H	-11.87761500	-1.81202300	0.47014700
H	-11.02622300	-2.93171000	-0.60025300
C	-9.74385400	-1.84853400	0.76202200
C	-9.80313300	-1.19398100	1.99676700
C	-8.63963800	-0.82451200	2.65712200
H	-8.70164700	-0.24161000	3.56906200
C	-7.38390200	-1.08782300	2.11285200
C	-7.31915200	-1.87311800	0.93865600

C	-8.50154200	-2.24331000	0.28061900
H	-8.43018500	-2.76554200	-0.66570700
C	-6.16952600	-0.41108000	2.67203800
H	-5.23886100	-0.84334100	2.31110400
H	-6.16683900	-0.43668000	3.76312100
C	-10.77953500	0.87849100	-0.66880300
H	-11.67660200	1.43532300	-0.94580300
H	-10.81222400	0.71177100	0.40950700
C	-9.52573200	1.68133400	-1.05315700
C	-9.74522200	3.16678100	-0.71310900
H	-10.11583300	3.23908200	0.31801400
H	-10.53228800	3.57604900	-1.35625200
C	-8.47516600	4.03041500	-0.81288800
H	-8.73453900	5.02734900	-0.43950700
C	-7.38868900	3.43377700	0.08837400
H	-6.50218900	4.07929100	0.09296000
H	-7.74794300	3.37051000	1.11979800
C	-7.00732000	2.03248100	-0.41334800
C	-8.25760700	1.12752800	-0.37879500
H	-8.01630300	0.15735300	-0.81525600
H	-8.48863800	0.95078200	0.67426400
C	-7.28822600	2.93903000	-2.83828500
H	-6.67741200	3.21645100	-3.70335600
C	-7.92030900	4.20787200	-2.23959700
H	-8.70476300	4.58136300	-2.90637700
H	-7.15496100	4.99195400	-2.19439300
C	-6.40827700	2.17533600	-1.83472400
H	-5.45543600	2.70942900	-1.73452500
H	-6.16690300	1.18359400	-2.23244400
C	-5.88486300	1.42328100	0.45744500
H	-4.97004800	2.00115800	0.31683000
H	-5.66547900	0.40253100	0.14581200
H	-10.76750900	-0.92301200	2.41250400
H	-9.42329500	1.59224700	-2.13534300
H	-8.06406400	2.28071700	-3.22932500
C	0.94528500	-3.09018100	1.20823600
C	0.08254400	-2.05708000	1.32191400
C	0.55885000	-0.69554900	1.31073900
C	2.36565200	-2.86643400	1.08719300
C	2.84667000	-1.50673900	1.13952300
C	1.98364700	-0.47353700	1.24204700
C	-0.32049900	0.37310300	1.33594100
C	3.23934300	-3.92092400	0.89656000
C	0.14955500	1.71448900	1.29266400
C	-1.72802500	0.17654900	1.36006700

C	4.64594800	-3.73058200	0.83706700
C	2.76537900	-5.24922100	0.72301000
N	5.79016400	-3.57252900	0.81516700
N	2.35390900	-6.31650100	0.55520400
N	0.56304700	2.79319800	1.24481300
N	-2.86883600	-0.00953700	1.36547500
H	3.91121000	-1.32497000	1.06415800
H	2.35240000	0.54241000	1.25270600
H	-0.98138200	-2.23870200	1.39807500
H	0.57731400	-4.10691100	1.19544500

A*p*PA-TCNQ-7

Electronic Energy (EE): -4324.476838 Hartree

EE + Thermal Free Energy Correction: -4323.523280 Hartree

E (Thermal): 707.462 kcal/mol

Entropy (S): 367.894 cal/mol-kelvin

Imaginary frequencies: 0

0	1		
S	-11.95710800	-2.62418100	1.61875300
S	-10.02171100	3.14870600	-0.70937000
C	-12.05934900	-1.51163200	3.09722500
H	-13.10598400	-1.36690500	3.36698000
H	-11.58543200	-2.10219800	3.88362900
C	-11.35978300	-0.20470700	2.87508200
C	-12.08647900	0.96878100	2.65301100
C	-11.45760800	2.10669700	2.16496100
H	-12.04789100	2.98097100	1.91459800
C	-10.09440600	2.11216300	1.87831100
C	-9.33075900	0.97658900	2.23644300
C	-9.97723800	-0.16537900	2.73385800
H	-9.39018600	-1.05669000	2.91905500
C	-9.49916900	3.22185500	1.06867200
H	-8.41235300	3.23153700	1.12311500
H	-9.86931800	4.19612600	1.39238100

C	-12.87846700	-1.68696000	0.31735000
H	-13.66586700	-2.36189900	-0.02552300
H	-13.36307300	-0.83816900	0.80475500
C	-12.06279900	-1.20126600	-0.89449000
C	-13.05138300	-0.64104600	-1.94174300
H	-13.63285800	0.17573800	-1.49832200
H	-13.76220700	-1.42160400	-2.23654900
C	-12.28985300	-0.13098600	-3.17765600
H	-13.00706600	0.26086100	-3.90590600
C	-11.32342000	0.98920700	-2.76107900
H	-10.78582200	1.36710600	-3.63892200
H	-11.88170900	1.82904300	-2.33689100
C	-10.31631500	0.46351600	-1.71992100
C	-11.08954300	-0.07700800	-0.50694900
H	-10.38314000	-0.45761200	0.23048800
H	-11.64321300	0.73918500	-0.04243900
C	-11.26356600	-2.34908400	-1.54112300
H	-11.94410200	-3.15692400	-1.83593200
H	-10.56535000	-2.76440500	-0.80868100
C	-10.49596500	-1.83305500	-2.76951800
H	-9.92137700	-2.65493600	-3.20821400
C	-11.49404700	-1.28766100	-3.80370000
H	-12.17375000	-2.08422400	-4.12567500
H	-10.96213600	-0.93725200	-4.69491100
C	-9.53129500	-0.71114700	-2.34537900
H	-8.96398100	-0.35881500	-3.21442200
H	-8.80549900	-1.09479700	-1.61940100
C	-9.30754200	1.55096800	-1.31091600
H	-8.69359700	1.81508600	-2.17492100
H	-8.63568000	1.17571000	-0.53806100
H	-13.16013300	0.97072600	2.80591200
C	-7.93098400	0.93610000	2.01877900
C	-6.73431700	0.87662100	1.81317900
C	-5.40221000	0.81776400	1.58038700
C	-4.20655400	0.76480600	1.36772200
C	-2.81532000	0.70295300	1.12253900
C	-0.04942000	0.58128000	0.63988200
C	-2.09964700	1.86274200	0.76691600
C	-2.12273700	-0.51906300	1.22884900
C	-0.76202500	-0.58184700	0.99194800
C	-0.73903500	1.80291700	0.52899700
H	-2.62802700	2.80365300	0.68224000
H	-2.67001400	-1.41357300	1.49743800
H	-0.23030400	-1.52203700	1.06358000
H	-0.19282100	2.69686900	0.25609500

C	1.34194700	0.50529600	0.40086700
C	2.53431600	0.38522600	0.20069000
C	3.85022000	0.18014500	-0.02990700
C	5.02356900	-0.05009900	-0.25240400
S	9.70330000	0.27576500	2.45997900
S	6.90521500	2.33654900	-2.97830300
C	9.44234700	-1.30334200	1.52785400
H	10.40552900	-1.71186000	1.22314500
H	9.01089500	-1.96765100	2.27889400
C	8.53140300	-1.10131700	0.35625800
C	9.04974400	-1.03150200	-0.94404100
C	8.26882000	-0.56105100	-1.99043200
H	8.70964700	-0.42817200	-2.97184300
C	6.95054500	-0.15782600	-1.78524000
C	6.38045000	-0.36164700	-0.50429700
C	7.18468200	-0.82357300	0.54921900
H	6.75663700	-0.88755800	1.54191300
C	6.22830400	0.61872800	-2.84126400
H	5.15553400	0.65896800	-2.66859100
H	6.39354700	0.19884700	-3.83413700
C	10.41864700	1.40747400	1.18508600
H	11.41232800	1.67920800	1.54549500
H	10.54608300	0.84486200	0.25837000
C	9.58402900	2.67497800	0.94141200
C	10.45410300	3.74023800	0.24983000
H	10.95905900	3.27763200	-0.60848200
H	11.24317900	4.06855700	0.93516500
C	9.66504400	4.95468800	-0.27189600
H	10.36851600	5.57506200	-0.83792800
C	8.56271600	4.47117300	-1.22100400
H	8.04122700	5.32770300	-1.66457200
H	9.00028000	3.89734000	-2.04314200
C	7.56264900	3.59477300	-0.45136100
C	8.30834700	2.37709700	0.13408200
H	7.62379000	1.79504400	0.75267900
H	8.58883800	1.74375600	-0.71081800
C	7.86456200	5.20764400	1.57038500
H	7.31469400	5.98785400	2.10597400
C	9.04736500	5.84694200	0.82194400
H	9.81669200	6.15389600	1.53818800
H	8.69764900	6.76412800	0.33326400
C	6.89093000	4.46924800	0.63676100
H	6.27486000	5.21565800	0.12088100
H	6.20293800	3.85592800	1.22882900
C	6.41724500	3.11787600	-1.37510700

H	5.80457900	3.97726700	-1.65296900
H	5.76967900	2.41700300	-0.84695800
H	10.08485800	-1.30145300	-1.11991900
H	9.30381300	3.05141600	1.92594700
H	8.22925900	4.52820400	2.34074300
C	7.48783700	-3.84898100	-1.12164300
C	6.29172600	-3.42733700	-1.58847100
C	5.12422200	-3.42379800	-0.74399000
C	7.63762300	-4.28976300	0.24177100
C	6.45440400	-4.35491700	1.06658000
C	5.25687900	-3.94092100	0.59720400
C	3.91899100	-2.91440200	-1.19453100
C	8.88417500	-4.60446300	0.75870400
C	2.74370000	-2.93909800	-0.39666000
C	3.81043200	-2.31854100	-2.48016900
C	9.05232500	-5.02934200	2.10339000
C	10.06143700	-4.43222700	-0.01621300
N	9.17188500	-5.36206800	3.20436600
N	11.00421900	-4.24243500	-0.65853400
N	1.78781000	-2.97164000	0.25185300
N	3.76209600	-1.82295000	-3.52322900
H	6.54811400	-4.71644900	2.08278300
H	4.38110200	-3.96702400	1.23315900
H	6.19754100	-3.07597200	-2.60676700
H	8.35780600	-3.83750800	-1.76320100

A*p*PA-TCNQ-8

Electronic Energy (EE): -4324.476623 Hartree

EE + Thermal Free Energy Correction: -4323.523651 Hartree

E (Thermal): 707.469 kcal/mol

Entropy (S): 369.154 cal/mol-kelvin

Imaginary frequencies: 0

S	11.90291200	1.75069500	-2.83438900
S	9.86918000	-0.74324900	2.83410100
C	12.02350100	3.18703700	-1.66955700
H	13.07380100	3.43112400	-1.50696000
H	11.56991800	4.00268300	-2.23592100
C	11.30827200	2.93269100	-0.37705200

C	12.02007100	2.65364800	0.79342200
C	11.37163800	2.13707600	1.90755800
H	11.94904700	1.84384400	2.77709100
C	10.00310600	1.87743900	1.89132000
C	9.25668800	2.29240900	0.76365900
C	9.92287300	2.81846700	-0.35387100
H	9.34745000	3.04807700	-1.24243100
C	9.38162400	1.04116200	2.96628100
H	8.29610000	1.11747500	2.96881300
H	9.74920600	1.32237500	3.95461100
C	12.79213300	0.40002900	-1.93679300
H	13.57904800	0.06664100	-2.61703200
H	13.27807200	0.84798200	-1.06730300
C	11.95027600	-0.81281800	-1.50091300
C	12.91467100	-1.89568200	-0.96675100
H	13.49500400	-1.49035400	-0.12961500
H	13.62860600	-2.17644600	-1.74964900
C	12.12659400	-3.13449200	-0.50650200
H	12.82692600	-3.88807000	-0.13241100
C	11.15576700	-2.73886800	0.61747000
H	10.59900200	-3.61930700	0.95994900
H	11.71246200	-2.35329500	1.47670900
C	10.17250900	-1.66275100	0.11762700
C	10.97229400	-0.44614300	-0.37378300
H	10.28294300	0.31626900	-0.73611400
H	11.52543700	-0.01950800	0.46309300
C	11.15199100	-1.40591100	-2.67784100
H	11.83562700	-1.68506500	-3.48860500
H	10.47077100	-0.64761400	-3.07463800
C	10.35802500	-2.63737000	-2.21134300
H	9.78453000	-3.03788900	-3.05324800
C	11.33238900	-3.70687400	-1.69157000
H	12.01469000	-4.01360600	-2.49192000
H	10.78151000	-4.59994500	-1.37698700
C	9.38914000	-2.23416900	-1.08528100
H	8.80303500	-3.10437700	-0.76821700
H	8.68018300	-1.48289700	-1.45136400
C	9.16015100	-1.27289500	1.20882400
H	8.52700200	-2.13361300	1.43579800
H	8.50726700	-0.47414300	0.85486500
H	13.09646300	2.78491900	0.80973900
C	7.85310000	2.10571900	0.70466900
C	6.65275700	1.92858100	0.62970000
C	5.31618000	1.72847500	0.55418700
C	4.11622200	1.54613700	0.48707600

C	2.71976600	1.33782500	0.40960000
C	-0.05717500	0.93103000	0.25885200
C	2.05612100	0.55926300	1.37796600
C	1.96938600	1.90693800	-0.63774700
C	0.60334700	1.70705400	-0.71176600
C	0.69018800	0.35698300	1.30603600
H	2.62965200	0.11810100	2.18315600
H	2.47522900	2.50531800	-1.38458000
H	0.03015700	2.14816700	-1.51734900
H	0.17907700	-0.24693100	2.04462600
C	-1.45427300	0.72134400	0.19670200
C	-2.65104900	0.51231900	0.18672400
C	-3.97444500	0.23450800	0.21628900
C	-5.15452100	-0.05148500	0.27412800
S	-9.38353700	-0.21109400	-3.11455000
S	-7.62093400	2.27617000	2.57034100
C	-9.13074200	-1.73436500	-2.09144900
H	-10.08254400	-2.23812800	-1.92729500
H	-8.52155800	-2.36680800	-2.74010200
C	-8.43796700	-1.41262500	-0.80381100
C	-9.15089300	-1.34891300	0.39937200
C	-8.58149100	-0.77134800	1.52601000
H	-9.17459400	-0.64531800	2.42467500
C	-7.28616000	-0.25570100	1.49920000
C	-6.51239000	-0.45022600	0.33052000
C	-7.10422400	-1.01882900	-0.80589500
H	-6.52629100	-1.07888400	-1.72004000
C	-6.80044700	0.61769600	2.61327000
H	-5.72034600	0.75007800	2.59240400
H	-7.07699700	0.22141500	3.59058800
C	-10.37925800	0.88696300	-2.01067700
H	-11.32872300	1.05131600	-2.52330000
H	-10.59123700	0.34547700	-1.08690900
C	-9.70571000	2.23467800	-1.70716800
C	-10.75827200	3.23450600	-1.19515400
H	-11.34045500	2.75362400	-0.39794700
H	-11.46447100	3.46585600	-2.00009900
C	-10.16419800	4.53322400	-0.62052400
H	-10.99482000	5.10277500	-0.18948100
C	-9.17507100	4.18441700	0.49747200
H	-8.80156500	5.09921300	0.97286700
H	-9.67666400	3.59823200	1.27289700
C	-7.99968300	3.38144100	-0.08094200
C	-8.54098400	2.08228500	-0.71338200
H	-7.72427500	1.54856100	-1.20140600

H	-8.88513000	1.45140500	0.10956600
C	-8.14111900	4.89563000	-2.19411800
H	-7.58920900	5.70710100	-2.67878100
C	-9.47295200	5.44438700	-1.65308200
H	-10.15228500	5.65530800	-2.48563300
H	-9.28127500	6.40519000	-1.16070000
C	-7.25436800	4.28081100	-1.09849300
H	-6.78895500	5.09699800	-0.53285100
H	-6.43547200	3.71641100	-1.55776300
C	-6.96656900	3.03980500	1.01856500
H	-6.47328500	3.95851800	1.34059200
H	-6.19381500	2.37968800	0.62223000
H	-10.17543800	-1.70229800	0.43120900
H	-9.31842100	2.60540300	-2.65690500
H	-8.32677900	4.16182000	-2.97854100
C	-7.04314900	-3.94781200	0.91461900
C	-6.38961100	-3.37551400	1.94786500
C	-5.01155500	-2.96722500	1.82289500
C	-6.38761000	-4.15435800	-0.35301100
C	-4.99726700	-3.77964400	-0.46821800
C	-4.34071900	-3.21489700	0.56817900
C	-4.36237500	-2.33488200	2.86740000
C	-7.07993200	-4.67611300	-1.43184600
C	-2.99981100	-1.93952900	2.78740800
C	-5.04255400	-2.02825600	4.07721700
C	-6.46389400	-4.86617100	-2.69783800
C	-8.46260400	-4.98918400	-1.33509000
N	-5.95386400	-5.00359600	-3.72631000
N	-9.59372700	-5.20812100	-1.23862600
N	-1.88783900	-1.63019200	2.73335900
N	-5.62499600	-1.77062700	5.04201000
H	-4.49221700	-3.94005300	-1.41220100
H	-3.30669500	-2.91359700	0.46317600
H	-6.89832500	-3.20599700	2.88709800
H	-8.07976600	-4.23739000	1.01777300

A*p*PA-TCNQ-9

Electronic Energy (EE): -4324.476472 Hartree

EE + Thermal Free Energy Correction: -4323.521449 Hartree

E (Thermal): 707.585 kcal/mol

Entropy (S): 365.223 cal/mol-kelvin

Imaginary frequencies: 0

0	1		
S	11.85093	-0.22263	2.893487
S	9.782643	-0.17917	-3.28715
C	12.11529	-1.97255	2.34338
H	13.18545	-2.17132	2.277129
H	11.71347	-2.55501	3.174708
C	11.41583	-2.27589	1.052692
C	12.13405	-2.39462	-0.14083
C	11.47705	-2.38282	-1.36417
H	12.05263	-2.38898	-2.28293
C	10.09287	-2.24458	-1.43963
C	9.351637	-2.26713	-0.23528
C	10.02667	-2.28953	0.994793
H	9.447688	-2.21633	1.907365
C	9.438427	-1.91927	-2.74628
H	8.362465	-2.07966	-2.71686
H	9.853651	-2.51558	-3.56051
C	12.65239	0.772635	1.555941
H	13.39085	1.398763	2.061626
H	13.1965	0.07908	0.911102
C	11.72678	1.663226	0.707219
C	12.61239	2.556415	-0.19034
H	13.2497	1.926164	-0.8216
H	13.27609	3.167075	0.432565
C	11.73689	3.464936	-1.07129
H	12.38214	4.088275	-1.69845
C	10.83621	2.600239	-1.96764
H	10.21812	3.24066	-2.60817
H	11.4482	1.977127	-2.62654
C	9.932364	1.699937	-1.10386
C	10.81689	0.823741	-0.20314
H	10.18425	0.188556	0.416264
H	11.42867	0.171714	-0.82695
C	10.84676	2.572574	1.586548
H	11.48021	3.188622	2.236047
H	10.21994	1.95474	2.235984
C	9.966382	3.471998	0.703241

H	9.337003	4.100446	1.341206
C	10.86232	4.358521	-0.17683
H	11.49246	4.996757	0.452246
H	10.24779	5.021112	-0.79597
C	9.068502	2.59979	-0.19218
H	8.420699	3.237259	-0.80483
H	8.415219	1.974524	0.426867
C	8.987309	0.851485	-1.97213
H	8.295664	1.511832	-2.50022
H	8.389124	0.188036	-1.34688
H	13.21741	-2.43527	-0.11201
C	7.93778	-2.16286	-0.24277
C	6.729885	-2.02713	-0.23182
C	5.386863	-1.8559	-0.22776
C	4.18353	-1.6846	-0.22312
C	2.785662	-1.4686	-0.2138
C	0.012888	-1.01082	-0.17928
C	1.972706	-2.0697	0.766775
C	2.185417	-0.64064	-1.18222
C	0.821511	-0.41464	-1.16479
C	0.608053	-1.84727	0.78544
H	2.429603	-2.71071	1.50987
H	2.806079	-0.17844	-1.9391
H	0.365055	0.226611	-1.90811
H	-0.02015	-2.3155	1.5321
C	-1.38157	-0.77914	-0.14286
C	-2.58197	-0.60807	-0.06634
C	-3.9216	-0.46845	0.056453
C	-5.12653	-0.38683	0.194922
S	-9.38809	1.24141	-2.74015
S	-6.61138	2.196904	3.007689
C	-9.55845	-0.42504	-1.94923
H	-10.6081	-0.64206	-1.75456
H	-9.21308	-1.10397	-2.73121
C	-8.72672	-0.52284	-0.70832
C	-9.31369	-0.43236	0.559368
C	-8.52903	-0.2285	1.686327
H	-9.0007	-0.06905	2.649327
C	-7.14162	-0.125	1.5935
C	-6.53531	-0.36238	0.337139
C	-7.339	-0.54942	-0.79609
H	-6.86222	-0.64022	-1.76446
C	-6.34527	0.382971	2.754339
H	-5.28048	0.188124	2.640306
H	-6.6743	-0.05671	3.696202

C	-9.93015	2.405249	-1.41066
H	-10.8089	2.921918	-1.8006
H	-10.2479	1.817764	-0.54727
C	-8.85646	3.427716	-1.0057
C	-9.51798	4.604181	-0.26553
H	-10.1738	4.201317	0.517652
H	-10.1631	5.155582	-0.95831
C	-8.52122	5.564095	0.409197
H	-9.11044	6.279509	0.993271
C	-7.62346	4.770624	1.365247
H	-6.95855	5.449247	1.912742
H	-8.2343	4.248097	2.107092
C	-6.78968	3.753775	0.570797
C	-7.74179	2.78585	-0.16197
H	-7.16001	2.112312	-0.79278
H	-8.21631	2.170056	0.605643
C	-6.58086	5.551638	-1.30346
H	-5.83376	6.228034	-1.73041
C	-7.645	6.379062	-0.56131
H	-8.27385	6.908228	-1.28494
H	-7.13863	7.15138	0.029804
C	-5.86561	4.536617	-0.39546
H	-5.13638	5.078387	0.218868
H	-5.29166	3.832238	-1.00742
C	-5.84823	2.957429	1.504919
H	-5.07631	3.627515	1.887206
H	-5.34312	2.165276	0.950471
H	-10.3937	-0.46439	0.650167
H	-8.42745	3.807217	-1.93382
H	-7.02879	5.039511	-2.15515
C	-8.07182	-3.5927	0.52827
C	-7.23346	-3.40012	1.568487
C	-5.80284	-3.39692	1.381311
C	-7.56274	-3.78673	-0.80692
C	-6.13027	-3.8228	-0.98955
C	-5.29109	-3.63788	0.052452
C	-4.95074	-3.14461	2.440763
C	-8.42496	-3.91228	-1.88217
C	-3.53685	-3.14763	2.298125
C	-5.45644	-2.84377	3.734702
C	-7.94877	-4.0781	-3.21036
C	-9.83238	-3.8189	-1.70974
N	-7.54778	-4.19954	-4.28797
N	-10.9719	-3.7107	-1.54674
N	-2.38622	-3.16132	2.193121

N	-5.89356	-2.58019	4.771955
H	-5.73706	-3.9797	-1.98587
H	-4.2197	-3.64023	-0.09962
H	-7.6277	-3.23352	2.561722
H	-9.14225	-3.57844	0.680248

A*p*PA-TCNQ-10

Electronic Energy (EE): -4324.475807 Hartree

EE + Thermal Free Energy Correction: -4323.521841 Hartree

E (Thermal): 707.448 kcal/mol

Entropy (S): 366.988 cal/mol-kelvin

Imaginary frequencies: 0

S	-7.79338200	2.24691500	-2.78819700
S	-7.33372800	-0.82921100	2.93190300
C	-9.29833900	1.16483600	-2.77516500
H	-10.18892000	1.78524800	-2.67057300
H	-9.30113800	0.73477400	-3.77865900
C	-9.22717800	0.11255900	-1.70935200
C	-10.01528000	0.19693000	-0.55762100
C	-9.73770300	-0.59543300	0.54913200
H	-10.30675700	-0.46028900	1.46198100
C	-8.65770800	-1.47580300	0.55504900
C	-7.93940500	-1.65578900	-0.64969100
C	-8.24891100	-0.87239400	-1.77156900
H	-7.62732500	-0.95990400	-2.65423200
C	-8.17702900	-2.07055600	1.84320100
H	-7.50954500	-2.91495800	1.68134600
H	-9.01269700	-2.40783600	2.45876300
C	-7.86043600	3.09305000	-1.14448400
H	-7.86388800	4.16199900	-1.36862600
H	-8.82297200	2.85013400	-0.68902600
C	-6.72159100	2.78693800	-0.15354700
C	-6.84303700	3.77799500	1.02608500
H	-7.82672800	3.66941200	1.49747900
H	-6.77445900	4.80681200	0.65423300
C	-5.73548500	3.51865000	2.06168100
H	-5.83906100	4.23112000	2.88611300
C	-5.86475300	2.08701900	2.60412700
H	-5.08740700	1.89406000	3.35233800
H	-6.83135000	1.95834200	3.10060300

C	-5.73999600	1.06810900	1.45542100
C	-6.82463000	1.35838000	0.40515700
H	-6.72868200	0.65056700	-0.41752300
H	-7.80799900	1.21082300	0.85346100
C	-5.33372300	2.96441600	-0.80003300
H	-5.23391700	3.98169900	-1.19702600
H	-5.23523800	2.27602300	-1.64449500
C	-4.22907800	2.69392100	0.23544000
H	-3.25027600	2.80891300	-0.24235200
C	-4.36005800	3.69272200	1.39694900
H	-4.24551500	4.71683000	1.02532400
H	-3.56550300	3.52484800	2.13227600
C	-4.36388800	1.26069400	0.77918800
H	-3.56894300	1.06089600	1.50538100
H	-4.25582000	0.53647300	-0.03759300
C	-5.81173400	-0.37419000	1.98415800
H	-4.98110500	-0.54738900	2.67064000
H	-5.71247000	-1.08404200	1.16451400
H	-10.81272900	0.93025200	-0.50734500
C	-6.77755500	-2.46466700	-0.69221500
C	-5.68914000	-3.00556300	-0.68674200
C	-4.41313100	-3.44327100	-0.59261900
C	-3.23714000	-3.72147400	-0.46006200
C	-1.84647300	-3.84207200	-0.24473500
C	0.92792800	-3.66479100	0.18850700
C	-0.94839700	-3.94608800	-1.32347700
C	-1.33271300	-3.72421700	1.06189800
C	0.02987600	-3.63366400	1.27335700
C	0.41549400	-3.85680100	-1.10918300
H	-1.33614600	-4.04870400	-2.32908100
H	-2.01978500	-3.65708200	1.89589200
H	0.41884200	-3.50732700	2.27575700
H	1.10162300	-3.90277600	-1.94534100
C	2.30521500	-3.43289700	0.39719000
C	3.46954500	-3.13521400	0.57999000
C	4.75314400	-2.75238200	0.76741700
C	5.89648600	-2.36398400	0.90820700
S	7.96796500	2.13520600	2.99822500
S	7.53605800	-1.11369800	-2.56982200
C	8.94563400	0.60343700	3.36707500
H	9.99891300	0.86215300	3.47836300
H	8.56538600	0.29308100	4.34186800
C	8.74191200	-0.44660400	2.31832900
C	9.71973300	-0.68210900	1.34612700
C	9.42539300	-1.42803500	0.21315300

H	10.16782000	-1.53178200	-0.56994200
C	8.15307300	-1.95721500	0.00767300
C	7.20394300	-1.83770500	1.04900200
C	7.51467400	-1.08645000	2.19316900
H	6.74584100	-0.93081000	2.94025400
C	7.76910900	-2.48046100	-1.34111400
H	6.86850300	-3.09071200	-1.30784200
H	8.57124800	-3.07610300	-1.77975400
C	8.58267800	2.64796100	1.33208300
H	9.02842000	3.63565200	1.46328000
H	9.37729100	1.96241700	1.03160200
C	7.48261200	2.70643200	0.25953900
C	7.94389700	3.59050200	-0.91315600
H	8.94638400	3.26725400	-1.22319500
H	8.04253500	4.62718400	-0.57255200
C	7.02904700	3.52241800	-2.14980600
H	7.51970500	4.09507300	-2.94444600
C	6.89767500	2.06426300	-2.60307900
H	6.31010100	2.00321500	-3.52648000
H	7.88521900	1.64635900	-2.81947000
C	6.21911500	1.23695500	-1.50089900
C	7.07457000	1.30264200	-0.21961900
H	6.55653100	0.78791000	0.59058800
H	7.98772000	0.73702100	-0.41972600
C	4.70175900	3.30730000	-1.03558600
H	3.66670700	3.63136700	-1.18574400
C	5.62456700	4.12627200	-1.95537700
H	5.70190600	5.15243600	-1.58109200
H	5.16094800	4.19302700	-2.94664400
C	4.78859800	1.79289200	-1.29078900
H	4.21287900	1.55924900	-2.19362000
H	4.31397000	1.25615100	-0.46055000
C	6.03490600	-0.23096200	-1.95117900
H	5.32754100	-0.26426400	-2.78019100
H	5.61609900	-0.82581300	-1.13914100
H	10.69992100	-0.23071500	1.45377500
H	6.62350100	3.18690900	0.72924300
H	4.92558000	3.52239700	0.00922100
C	1.37068200	-0.08274700	0.58550600
C	1.58256600	-0.20562900	-0.74198700
C	0.50504700	-0.55837200	-1.63499600
C	0.05705400	-0.29072800	1.14730400
C	-1.03388600	-0.57369800	0.24457700
C	-0.81975600	-0.70583600	-1.08087600
C	0.74206600	-0.77826200	-2.97990900

C	-0.14792600	-0.25117400	2.51464700
C	-0.28739400	-1.23419900	-3.84691100
C	2.03354700	-0.60605500	-3.54763700
C	-1.42714000	-0.51604600	3.07453800
C	0.91241000	0.00384500	3.42590600
N	-2.47418400	-0.74894200	3.50588100
N	1.78437900	0.20547800	4.15753300
N	-1.13312600	-1.63442500	-4.52599400
N	3.08789100	-0.45137300	-3.99553900
H	-2.02920100	-0.70048500	0.64752900
H	-1.64204000	-0.94203600	-1.74303900
H	2.57643000	-0.07580700	-1.14791600
H	2.19467200	0.14257100	1.25027700

3.0 AP6A-TCNQ coordinates

AP6A

Electronic Energy (EE): -4155.760135 Hartree

EE + Thermal Free Energy Correction: -4154.587474 Hartree

E (Thermal): 844.511 kcal/mol

Entropy (S): 366.419 cal/mol-kelvin

Imaginary frequencies: 0

0	1		
C	-1.68474	3.894469	-0.62483
H	-2.44252	4.157328	-1.36988
H	-1.16439	4.819943	-0.36413
C	-2.40089	3.327822	0.618622
H	-1.72098	3.360561	1.47697
H	-2.63626	2.274132	0.450953
C	-3.69782	4.07461	0.934903
H	-3.47758	5.125189	1.162929
H	-4.32441	4.079706	0.034602
C	-4.49225	3.454276	2.085026
H	-4.71074	2.406256	1.843763
H	-3.87654	3.435577	2.992985
C	-5.8038	4.186798	2.379395

H	-6.40937	4.217947	1.465182
H	-5.58564	5.231636	2.629445
C	-6.6074	3.543178	3.510493
H	-7.54063	4.081591	3.698751
H	-6.03671	3.538917	4.443909
H	-6.86048	2.504964	3.277825
C	2.054258	0.095663	-3.06565
H	2.892391	0.639162	-3.51189
H	1.559691	-0.44822	-3.8755
C	2.606207	-0.91099	-2.03818
H	1.796804	-1.58073	-1.72586
H	2.928443	-0.37375	-1.1412
C	3.779583	-1.72358	-2.58814
H	3.504139	-2.15432	-3.55927
H	4.619825	-1.04531	-2.7818
C	4.233671	-2.84971	-1.65724
H	4.455659	-2.43978	-0.66393
H	3.403486	-3.55364	-1.51669
C	5.458492	-3.60825	-2.17651
H	6.324421	-2.93642	-2.18395
H	5.287508	-3.89481	-3.22109
C	5.781124	-4.8591	-1.35743
H	6.693017	-5.34543	-1.71321
H	4.966207	-5.58696	-1.41849
H	5.926322	-4.61458	-0.30231
C	-0.71286	2.929534	-1.25379
C	-1.1898	1.810646	-1.98425
C	0.65814	3.095157	-1.13949
H	1.04878	3.942917	-0.58915
C	1.566441	2.194008	-1.72262
C	-0.28206	0.916567	-2.57587
H	-0.67201	0.073858	-3.1346
C	1.08999	1.08255	-2.46121
C	-2.58566	1.605449	-2.08298
C	2.96025	2.376929	-1.57442
C	4.171628	2.423422	-1.48174
S	-8.38244	-1.80499	-3.09702
S	-9.15314	0.734609	2.857557
C	-8.24461	0.018994	-3.40218
H	-8.90936	0.175848	-4.25337
C	-8.67169	0.8244	-2.21492

C	-7.71861	1.415432	-1.3549
C	-8.12363	1.912726	-0.10618
H	-7.36785	2.278361	0.577514
C	-9.44833	1.82767	0.306287
C	-10.4029	1.371456	-0.60805
C	-10.0148	0.881271	-1.84818
H	-10.7565	0.450973	-2.51161
C	-9.8071	2.062466	1.74249
H	-10.8855	2.147376	1.879948
H	-9.3411	2.966156	2.138651
C	-7.17772	-2.1112	-1.72615
H	-6.50068	-2.88162	-2.1021
H	-6.59108	-1.20195	-1.58996
C	-7.77379	-2.57191	-0.38457
C	-6.60786	-2.93538	0.561856
H	-5.95817	-2.06286	0.695065
H	-5.99745	-3.72754	0.113308
C	-7.15145	-3.40088	1.924587
H	-6.31176	-3.65245	2.580163
C	-7.97625	-2.273	2.566342
H	-8.35568	-2.59464	3.5437
H	-7.34493	-1.39601	2.736246
C	-9.15297	-1.88808	1.648588
C	-8.59518	-1.45798	0.282936
H	-9.41883	-1.1779	-0.37402
H	-7.96709	-0.57759	0.417238
C	-8.66762	-3.81455	-0.56097
H	-8.0907	-4.62336	-1.02513
H	-9.49355	-3.57629	-1.23767
C	-9.21502	-4.27252	0.800179
H	-9.8587	-5.14631	0.657091
C	-8.04351	-4.63708	1.726409
H	-7.46253	-5.4572	1.290673
H	-8.4203	-4.98671	2.693871
C	-10.0371	-3.13698	1.434381
H	-10.4528	-3.46707	2.393432
H	-10.8831	-2.88141	0.785657
C	-10.0278	-0.78638	2.273731
H	-10.5368	-1.18571	3.153838
H	-10.7963	-0.47312	1.56386
H	-7.22808	0.255058	-3.71162

H	-11.4487	1.344637	-0.32189
S	9.70855	-2.09556	-1.63629
S	7.947294	2.539637	2.590977
C	10.55564	-0.58285	-2.29195
H	10.34482	-0.62594	-3.36228
C	10.03442	0.675196	-1.66451
C	10.79119	1.377794	-0.72196
C	10.203	2.359762	0.065109
H	10.78106	2.837319	0.848266
C	8.84764	2.662059	-0.04889
C	8.1114	2.049641	-1.08872
C	8.722016	1.072878	-1.8902
H	8.118557	0.550048	-2.62223
C	8.163673	3.476571	1.005046
H	7.194786	3.844816	0.672788
H	8.772143	4.330885	1.306513
C	10.17908	-2.10134	0.153096
H	10.65914	-3.06729	0.324495
H	10.93645	-1.32748	0.295694
C	9.037543	-1.91417	1.168727
C	9.601603	-2.18765	2.580694
H	10.43083	-1.50045	2.785579
H	10.00514	-3.20565	2.628683
C	8.499489	-2.00957	3.639674
H	8.91796	-2.20783	4.63156
C	7.965929	-0.56925	3.589826
H	7.189122	-0.42987	4.351097
H	8.770036	0.138039	3.814349
C	7.388101	-0.26271	2.194899
C	8.485678	-0.48019	1.14134
H	8.079155	-0.2772	0.151303
H	9.297151	0.227033	1.315923
C	7.878829	-2.89342	0.90526
H	8.248788	-3.92537	0.92594
H	7.476535	-2.7152	-0.09476
C	6.775771	-2.7065	1.958914
H	5.953553	-3.39833	1.750248
C	7.350522	-2.99089	3.355849
H	7.71237	-4.02354	3.409125
H	6.569205	-2.88277	4.115966
C	6.246753	-1.26275	1.903462

H	5.440007	-1.1318	2.633663
H	5.823327	-1.05603	0.913848
C	6.805287	1.158952	2.129573
H	5.965227	1.233678	2.823904
H	6.420141	1.367259	1.13166
H	11.83065	1.111999	-0.56363
H	11.63253	-0.68553	-2.15479
C	-3.79773	1.520996	-2.03649
C	-5.1401	1.462109	-1.87916
C	-6.33873	1.439201	-1.67741
C	5.5192	2.390126	-1.3711
C	6.725252	2.290687	-1.25584

AP6A-TCNQ-1

Electronic Energy (EE): -4834.592092 Hartree

EE + Thermal Free Energy Correction: -4833.305468 Hartree

E (Thermal): 935.635 kcal/mol

Entropy (S): 432.193 cal/mol-kelvin

Imaginary frequencies: 0

C	-1.68737	3.285304	-1.92656
H	-2.18246	3.144516	-2.89264
H	-1.11806	4.215728	-1.99983
C	-2.77287	3.430067	-0.84677
H	-2.33362	3.875061	0.052608
H	-3.13269	2.439754	-0.56744
C	-3.97256	4.255133	-1.31461
H	-3.63798	5.23572	-1.67548
H	-4.42389	3.746342	-2.17512
C	-5.03025	4.432465	-0.22301
H	-5.26003	3.456575	0.215204
H	-4.6194	5.047043	0.588521
C	-6.33345	5.059632	-0.72306
H	-6.73182	4.445733	-1.54003
H	-6.12828	6.045314	-1.15718
C	-7.3837	5.187111	0.382225
H	-8.31845	5.60985	0.003279
H	-7.03038	5.838545	1.187133

H	-7.60554	4.212317	0.826581
C	2.199319	-1.09291	-1.82657
H	3.109691	-0.65888	-2.25231
H	1.836914	-1.83236	-2.54668
C	2.571356	-1.80693	-0.5186
H	1.675947	-2.27871	-0.09781
H	2.923626	-1.0719	0.208695
C	3.653893	-2.86863	-0.71561
H	3.288221	-3.64198	-1.40285
H	4.520388	-2.40833	-1.20607
C	4.103107	-3.51356	0.597282
H	4.524632	-2.74363	1.253585
H	3.22881	-3.91392	1.126456
C	5.1236	-4.63855	0.401829
H	5.950089	-4.27525	-0.22058
H	4.658508	-5.45335	-0.16563
C	5.681373	-5.17188	1.722273
H	6.404125	-5.97516	1.556056
H	4.880872	-5.56933	2.353922
H	6.181366	-4.3765	2.281477
C	-0.72844	2.136253	-1.72962
C	-1.11276	0.808856	-2.05908
C	0.564238	2.335006	-1.27786
H	0.880212	3.328068	-0.98034
C	1.508655	1.289898	-1.22588
C	-0.15398	-0.22114	-2.0661
H	-0.46167	-1.20859	-2.39012
C	1.164124	-0.00231	-1.69662
C	-2.46234	0.546211	-2.3691
C	2.801747	1.551583	-0.72454
C	3.90746	1.743502	-0.25492
S	-8.09719	-3.27427	-2.31212
S	-9.58625	1.876405	1.394364
C	-7.89628	-1.85175	-3.48373
H	-8.43854	-2.18416	-4.37051
C	-8.46938	-0.58378	-2.93174
C	-7.63393	0.417337	-2.38648
C	-8.18767	1.447282	-1.60867
H	-7.51967	2.136792	-1.10863
C	-9.55544	1.503713	-1.36936
C	-10.3865	0.596287	-2.03467

C	-9.84771	-0.42897	-2.80115
H	-10.5018	-1.17755	-3.23361
C	-10.0986	2.40743	-0.30369
H	-11.1862	2.473075	-0.34925
H	-9.69368	3.41753	-0.37976
C	-7.07971	-2.78272	-0.84591
H	-6.35699	-3.58817	-0.70606
H	-6.51352	-1.89258	-1.12098
C	-7.85068	-2.54353	0.463966
C	-6.82282	-2.33417	1.597922
H	-6.18514	-1.48192	1.352711
H	-6.17566	-3.21392	1.682138
C	-7.54256	-2.07286	2.932405
H	-6.79661	-1.92161	3.719603
C	-8.41518	-0.81142	2.812291
H	-8.91812	-0.61124	3.766177
H	-7.79059	0.056553	2.582274
C	-9.46167	-0.99758	1.696594
C	-8.72536	-1.28219	0.378373
H	-9.45175	-1.40567	-0.42507
H	-8.09839	-0.42554	0.132161
C	-8.73874	-3.74753	0.831332
H	-8.12318	-4.6517	0.907863
H	-9.46966	-3.91938	0.035641
C	-9.46177	-3.48686	2.162899
H	-10.0991	-4.34387	2.403221
C	-8.42648	-3.28064	3.280503
H	-7.81093	-4.17998	3.391879
H	-8.93209	-3.11403	4.238117
C	-10.3347	-2.22644	2.036564
H	-10.8748	-2.05055	2.974093
H	-11.0868	-2.37055	1.251981
C	-10.3928	0.222241	1.576847
H	-11.0072	0.294582	2.477168
H	-11.0689	0.091471	0.729052
H	-6.84436	-1.74669	-3.74369
H	-11.4608	0.657058	-1.89831
S	11.02223	2.34165	-1.64918
S	7.563041	-1.54658	2.264755
C	10.90898	3.310618	-0.07318
H	10.6493	4.312902	-0.41949

C	9.881338	2.755074	0.866615
C	10.26234	2.04929	2.011864
C	9.336149	1.292341	2.7169
H	9.66378	0.684524	3.55267
C	8.009431	1.199495	2.300335
C	7.591237	2.019803	1.226866
C	8.532753	2.795022	0.532302
H	8.209578	3.347098	-0.34182
C	7.112174	0.145608	2.870955
H	6.060462	0.321516	2.660618
H	7.234763	0.060744	3.952029
C	11.60205	0.676196	-1.09003
H	12.51678	0.482114	-1.65457
H	11.8781	0.761024	-0.03666
C	10.62555	-0.49821	-1.28365
C	11.37907	-1.80724	-0.95739
H	11.75773	-1.76708	0.070605
H	12.24726	-1.91108	-1.61842
C	10.44474	-3.0188	-1.12417
H	10.99714	-3.93411	-0.88899
C	9.250118	-2.8838	-0.16662
H	8.585603	-3.74997	-0.26962
H	9.598523	-2.86552	0.870202
C	8.470443	-1.58964	-0.46842
C	9.421635	-0.3907	-0.33579
H	8.877509	0.526608	-0.55838
H	9.772578	-0.32399	0.694461
C	10.10856	-0.57954	-2.73317
H	10.9539	-0.66895	-3.426
H	9.580405	0.344995	-2.98368
C	9.167499	-1.78538	-2.89346
H	8.7976	-1.82055	-3.9231
C	9.934693	-3.07844	-2.57315
H	10.7749	-3.19751	-3.26599
H	9.2819	-3.94829	-2.7048
C	7.974765	-1.64492	-1.93055
H	7.289523	-2.49074	-2.05967
H	7.410842	-0.73367	-2.16089
C	7.245255	-1.44515	0.448315
H	6.541715	-2.24773	0.231162
H	6.730296	-0.5049	0.253443

H	11.30326	2.046008	2.316466
H	11.89429	3.358447	0.391579
C	-3.66568	0.430225	-2.51068
C	-5.01225	0.367925	-2.56861
C	-6.22699	0.364613	-2.52313
C	5.142143	1.897578	0.272543
C	6.258099	1.977888	0.747518
C	0.447419	1.039753	2.085485
C	0.273543	-0.32288	1.653973
H	1.129155	-0.98183	1.665516
C	-0.92017	-0.76744	1.202743
H	-1.0223	-1.78263	0.841835
C	-0.71453	1.893185	2.098386
H	-0.60809	2.912331	2.447603
C	-1.92246	1.429921	1.70575
H	-2.79105	2.07216	1.748113
C	-2.07722	0.089712	1.194937
C	-3.29534	-0.36039	0.710291
C	1.6913	1.50424	2.484413
C	-4.46432	0.44358	0.740436
C	-3.44241	-1.66338	0.162841
C	1.904885	2.867822	2.81766
C	2.807688	0.633097	2.591583
N	-5.42679	1.083464	0.770168
N	-3.55177	-2.72601	-0.27883
N	3.698322	-0.09593	2.698225
N	2.058287	3.985015	3.073052

AP6A-TCNQ-2

Electronic Energy (EE): -4834.591855 Hartree

EE + Thermal Free Energy Correction: -4833.305732 Hartree

E (Thermal): 935.641 kcal/mol

Entropy (S): 433.269 cal/mol-kelvin

Imaginary frequencies: 0

C	-1.59354	0.017548	-3.74943

H	-2.43995	-0.56771	-4.11948
H	-1.21828	0.603894	-4.59313
C	-2.09129	0.973179	-2.65374
H	-1.26806	1.634599	-2.35908
H	-2.35865	0.391142	-1.76868
C	-3.29562	1.805728	-3.0953
H	-3.07943	2.274179	-4.0636
H	-4.14988	1.138219	-3.26198
C	-3.67652	2.895085	-2.09223
H	-3.86002	2.446494	-1.10825
H	-2.82469	3.572017	-1.95794
C	-4.90456	3.709536	-2.50712
H	-5.79094	3.065168	-2.48886
H	-4.78497	4.033152	-3.54805
C	-5.13166	4.934826	-1.61996
H	-6.04776	5.462701	-1.89716
H	-4.29767	5.637476	-1.70446
H	-5.21157	4.653406	-0.56794
C	2.642506	-3.62924	-1.97434
H	3.42079	-3.65914	-2.74259
H	2.251801	-4.64424	-1.8695
C	3.27975	-3.19519	-0.6391
H	2.594727	-3.44923	0.174993
H	3.412836	-2.10843	-0.62995
C	4.630029	-3.86224	-0.37662
H	4.544644	-4.94144	-0.55248
H	5.360536	-3.48611	-1.10316
C	5.141802	-3.62736	1.045821
H	5.135995	-2.55237	1.263842
H	4.441061	-4.08636	1.754568
C	6.547431	-4.18387	1.287686
H	7.26454	-3.62354	0.677768
H	6.586287	-5.22269	0.938485
C	6.974133	-4.12537	2.75499
H	7.992009	-4.49907	2.88942
H	6.307565	-4.72751	3.380374
H	6.949672	-3.10039	3.132442
C	-0.50993	-0.91773	-3.27869
C	-0.82524	-2.12586	-2.60855
C	0.828477	-0.61165	-3.47132
H	1.097266	0.31309	-3.96849

C	1.857467	-1.46669	-3.04037
C	0.202996	-3.0096	-2.23263
H	-0.06162	-3.95123	-1.76915
C	1.541797	-2.70627	-2.43051
C	-2.17359	-2.39986	-2.28402
C	3.210446	-1.08683	-3.18585
C	4.390183	-0.79356	-3.18936
S	-6.91361	-2.52091	2.594162
S	-8.92006	1.946398	-1.68961
C	-7.12663	-3.51467	1.044129
H	-7.6891	-4.38606	1.383706
C	-7.87682	-2.75013	-0.00268
C	-7.21238	-2.15685	-1.09953
C	-7.88394	-1.21121	-1.89208
H	-7.33139	-0.7015	-2.67202
C	-9.18944	-0.83111	-1.60852
C	-9.8809	-1.52325	-0.60933
C	-9.23157	-2.47	0.171668
H	-9.75778	-2.93568	0.997235
C	-9.76995	0.396263	-2.24503
H	-10.8394	0.483462	-2.05075
H	-9.62096	0.40691	-3.32654
C	-5.8147	-1.14313	2.045721
H	-4.94221	-1.18647	2.70278
H	-5.48098	-1.38039	1.034754
C	-6.40895	0.27448	2.09148
C	-5.29587	1.275617	1.713728
H	-4.92462	1.038093	0.708923
H	-4.45708	1.176106	2.413005
C	-5.82991	2.717539	1.742978
H	-5.02285	3.405492	1.474742
C	-6.98224	2.854715	0.735486
H	-7.36087	3.883262	0.73623
H	-6.62386	2.645319	-0.27533
C	-8.11795	1.875418	1.086121
C	-7.55555	0.445462	1.083233
H	-8.35012	-0.26221	1.321139
H	-7.19397	0.209644	0.082837
C	-6.92306	0.624274	3.500679
H	-6.11016	0.517859	4.229073
H	-7.70796	-0.08234	3.786489

C	-7.46752	2.061473	3.525079
H	-7.84194	2.290152	4.527805
C	-6.34245	3.04203	3.155093
H	-5.5251	2.96762	3.880598
H	-6.71052	4.072836	3.19013
C	-8.61881	2.193592	2.512585
H	-9.02884	3.209457	2.544058
H	-9.4314	1.507822	2.77931
C	-9.30641	2.016976	0.118327
H	-9.78472	2.98623	0.275054
H	-10.0513	1.245487	0.32519
H	-6.14594	-3.8467	0.710118
H	-10.9164	-1.27479	-0.40393
S	9.334054	-1.34437	1.636063
S	6.536503	3.679206	-1.42275
C	10.52302	-0.97583	0.263256
H	10.7584	-1.96661	-0.13072
C	9.921871	-0.08084	-0.77872
C	10.31667	1.255052	-0.89702
C	9.562226	2.151672	-1.64306
H	9.836797	3.200381	-1.65895
C	8.382759	1.756951	-2.27122
C	8.04776	0.383276	-2.25611
C	8.836411	-0.51952	-1.52716
H	8.511179	-1.54999	-1.45185
C	7.422309	2.785714	-2.78459
H	6.688899	2.357495	-3.46552
H	7.945981	3.58888	-3.30586
C	9.031961	0.31306	2.40274
H	9.305348	0.197924	3.453851
H	9.737507	1.016182	1.954724
C	7.600476	0.874684	2.309398
C	7.51018	2.10612	3.238217
H	8.25179	2.853326	2.932558
H	7.751214	1.812743	4.266579
C	6.099091	2.716647	3.182087
H	6.055901	3.584844	3.847402
C	5.78655	3.158038	1.744229
H	4.78842	3.606147	1.692457
H	6.502567	3.920103	1.420754
C	5.859113	1.951667	0.788671

C	7.258226	1.321239	0.878874
H	7.305435	0.454778	0.220828
H	8.001051	2.040512	0.530976
C	6.551383	-0.15731	2.763216
H	6.769341	-0.48959	3.785381
H	6.611153	-1.03603	2.116971
C	5.141275	0.450967	2.696675
H	4.409825	-0.30214	3.008993
C	5.066426	1.669288	3.631471
H	5.262772	1.361971	4.664591
H	4.060888	2.103373	3.607874
C	4.833395	0.892794	1.255168
H	3.823651	1.313136	1.199609
H	4.868281	0.029437	0.579891
C	5.492381	2.364301	-0.64608
H	4.477711	2.763334	-0.65111
H	5.511939	1.49992	-1.30662
H	11.18615	1.606506	-0.35229
H	11.43719	-0.55192	0.679833
C	-3.34921	-2.47791	-1.98107
C	-4.66164	-2.47043	-1.65585
C	-5.84043	-2.38691	-1.37029
C	5.687507	-0.43896	-3.05267
C	6.815344	-0.07451	-2.78318
C	-0.52896	0.979147	0.626812
C	0.526571	0.005341	0.483576
H	1.535046	0.345644	0.285243
C	0.268251	-1.31528	0.609554
H	1.069004	-2.0338	0.514926
C	-1.87145	0.498528	0.850575
H	-2.67359	1.217859	0.933569
C	-2.13338	-0.82162	0.947505
H	-3.14703	-1.16578	1.100539
C	-1.07206	-1.7935	0.854515
C	-1.33924	-3.14289	0.991416
C	-0.27467	2.338016	0.566923
C	-2.66181	-3.62937	1.173831
C	-0.31089	-4.12152	0.935838
C	-1.30995	3.294422	0.755592
C	1.032511	2.846435	0.3394
N	-3.73944	-4.02288	1.310755

N	0.529722	-4.9133	0.88174
N	2.099696	3.25177	0.158492
N	-2.16567	4.053846	0.920529

AP6A-TCNQ-3

Electronic Energy (EE): -4834.591605 Hartree

EE + Thermal Free Energy Correction: -4833.304600 Hartree

E (Thermal): 935.592 kcal/mol

Entropy (S): 431.248 cal/mol-kelvin

Imaginary frequencies: 0

C	-1.91113	-3.42533	1.90255
H	-2.5667	-3.43716	2.778712
H	-1.35263	-4.36533	1.917013
C	-2.7913	-3.37568	0.643743
H	-2.18423	-3.62663	-0.23327
H	-3.15403	-2.35793	0.500374
C	-4.00143	-4.30701	0.731221
H	-3.67341	-5.32557	0.97393
H	-4.6296	-3.97747	1.56781
C	-4.83475	-4.32199	-0.55214
H	-5.0607	-3.2929	-0.84739
H	-4.23983	-4.75381	-1.36747
C	-6.14898	-5.09506	-0.42261
H	-6.74258	-4.65407	0.387576
H	-5.94225	-6.12791	-0.11808
C	-6.96241	-5.08972	-1.71838
H	-7.91319	-5.61709	-1.60061
H	-6.41302	-5.57867	-2.52844
H	-7.17801	-4.06748	-2.04253
C	1.959411	0.752873	3.237006
H	2.801824	0.19266	3.655242
H	1.521284	1.32318	4.061028
C	2.503052	1.722968	2.177748
H	1.700545	2.400792	1.86347
H	2.809029	1.156069	1.298101

C	3.70322	2.533332	2.669937
H	3.445451	3.05225	3.60187
H	4.515725	1.839594	2.918421
C	4.200399	3.547226	1.636372
H	4.384316	3.0338	0.687162
H	3.407675	4.281989	1.4429
C	5.472838	4.281648	2.065287
H	6.277315	3.550151	2.204838
H	5.312748	4.751147	3.043359
C	5.914428	5.341778	1.054978
H	6.834468	5.840388	1.370985
H	5.144989	6.11	0.931067
H	6.097123	4.895385	0.073697
C	-0.94104	-2.28026	2.064122
C	-1.36845	-1.03054	2.588768
C	0.400526	-2.41951	1.758826
H	0.755853	-3.34147	1.31363
C	1.342657	-1.41127	2.046826
C	-0.41857	-0.05443	2.945501
H	-0.76773	0.855847	3.418973
C	0.938939	-0.23757	2.731822
C	-2.75132	-0.79916	2.731852
C	2.685637	-1.61345	1.6653
C	3.820821	-1.81873	1.278044
S	-8.58754	2.809147	2.350112
S	-9.06423	-1.6956	-2.33697
C	-8.46189	1.219303	3.295045
H	-9.16034	1.366484	4.120526
C	-8.84054	0.038184	2.457155
C	-7.85404	-0.8099	1.905428
C	-8.19592	-1.71622	0.888268
H	-7.40555	-2.27387	0.402384
C	-9.50012	-1.7999	0.416123
C	-10.4941	-1.06437	1.070235
C	-10.1647	-0.16114	2.072557
H	-10.9354	0.467472	2.504107
C	-9.79608	-2.5337	-0.85724
H	-10.8689	-2.66002	-1.00725
H	-9.33195	-3.52073	-0.87673
C	-7.318	2.626724	1.015441
H	-6.64542	3.478079	1.130852

H	-6.73597	1.731195	1.234027
C	-7.85315	2.579302	-0.42633
C	-6.64728	2.625406	-1.39089
H	-5.98911	1.777891	-1.18543
H	-6.06718	3.538675	-1.22006
C	-7.12693	2.564418	-2.85146
H	-6.25773	2.592825	-3.5168
C	-7.90321	1.257407	-3.08781
H	-8.23371	1.201113	-4.13209
H	-7.25444	0.396498	-2.90221
C	-9.1225	1.18997	-2.14765
C	-8.62613	1.278324	-0.69622
H	-9.47656	1.221524	-0.0166
H	-7.97868	0.425886	-0.49204
C	-8.76694	3.780608	-0.7349
H	-8.22172	4.715416	-0.55835
H	-9.62417	3.772159	-0.05527
C	-9.25035	3.718868	-2.19291
H	-9.90941	4.569865	-2.39273
C	-8.0402	3.766413	-3.13968
H	-7.48916	4.702445	-2.9972
H	-8.37493	3.743632	-4.18269
C	-10.0278	2.411663	-2.42298
H	-10.3989	2.37427	-3.4538
H	-10.9024	2.37659	-1.76277
C	-9.96019	-0.07883	-2.38703
H	-10.4147	-0.02707	-3.37903
H	-10.7718	-0.13113	-1.65788
H	-7.45846	1.124481	3.706666
H	-11.5254	-1.1549	0.746913
S	11.01829	-0.50815	2.076533
S	7.251362	-0.91801	-3.23893
C	10.96671	-2.3224	1.706601
H	10.78792	-2.76872	2.686844
C	9.891739	-2.67605	0.723037
C	10.20807	-3.02161	-0.59426
C	9.22068	-3.07118	-1.56906
H	9.494382	-3.255	-2.60176
C	7.892461	-2.77322	-1.27042
C	7.547488	-2.56106	0.08382
C	8.553151	-2.51941	1.06225

H	8.27998	-2.26764	2.079734
C	6.911413	-2.52218	-2.37316
H	5.880127	-2.53634	-2.02495
H	7.008953	-3.26061	-3.17051
C	11.46884	0.248291	0.449512
H	12.36369	0.843274	0.645382
H	11.7555	-0.56395	-0.22221
C	10.40498	1.135765	-0.22137
C	11.0607	1.835559	-1.43306
H	11.44843	1.081295	-2.12786
H	11.91419	2.43638	-1.09806
C	10.0371	2.73247	-2.15149
H	10.52106	3.217867	-3.00509
C	8.861888	1.87633	-2.64957
H	8.13334	2.506463	-3.17326
H	9.216721	1.128138	-3.36515
C	8.179337	1.169274	-1.46363
C	9.218245	0.305124	-0.73397
H	8.742432	-0.19093	0.111126
H	9.580566	-0.46966	-1.41059
C	9.875646	2.217792	0.739913
H	10.70892	2.828966	1.107427
H	9.41635	1.739191	1.609747
C	8.846206	3.106243	0.022615
H	8.469702	3.858908	0.721158
C	9.515013	3.799486	-1.17517
H	10.33972	4.433041	-0.83036
H	8.796572	4.452397	-1.68263
C	7.673602	2.243721	-0.47385
H	6.921676	2.871978	-0.96212
H	7.177162	1.756816	0.372841
C	6.965026	0.347431	-1.92192
H	6.211377	1.022082	-2.32628
H	6.508085	-0.15875	-1.07204
H	11.24329	-3.1933	-0.86826
H	11.94868	-2.64798	1.361709
C	-3.96505	-0.71409	2.690624
C	-5.30491	-0.68702	2.533589
C	-6.49473	-0.717	2.285954
C	5.081357	-2.0611	0.855438
C	6.21059	-2.30092	0.47482

C	0.843245	-0.28752	-1.18244
C	0.487937	0.968646	-0.57243
H	1.273397	1.673185	-0.3417
C	-0.79365	1.256533	-0.25593
H	-1.03624	2.18839	0.238229
C	-0.22473	-1.19724	-1.51224
H	0.021217	-2.13626	-1.9919
C	-1.51454	-0.87916	-1.25658
H	-2.30741	-1.55711	-1.53954
C	-1.86117	0.345897	-0.57892
C	-3.17411	0.646579	-0.25013
C	2.169231	-0.5925	-1.45166
C	-4.26049	-0.18822	-0.61888
C	-3.50935	1.829476	0.462587
C	2.575064	-1.85561	-1.95669
C	3.19324	0.368123	-1.24468
N	-5.15984	-0.84735	-0.92449
N	-3.77401	2.795066	1.04037
N	3.993388	1.186967	-1.08644
N	2.915262	-2.88704	-2.35344

AP6A-TCNQ-4

Electronic Energy (EE): -4834.588834 Hartree

EE + Thermal Free Energy Correction: -4833.304491 Hartree

E (Thermal): 935.425 kcal/mol

Entropy (S): 436.291 cal/mol-kelvin

Imaginary frequencies: 0

C	1.70236	-2.18523	2.607315
H	2.090996	-2.85011	1.830039
H	1.098799	-2.80848	3.271479
C	2.90933	-1.59587	3.374697
H	2.626693	-1.39874	4.413768
H	3.178514	-0.62916	2.944416
C	4.133624	-2.51395	3.311957
H	3.943669	-3.43018	3.884504
H	4.276998	-2.83068	2.272802

C	5.42254	-1.85263	3.800477
H	5.60238	-0.94822	3.205536
H	5.306481	-1.51916	4.839384
C	6.641439	-2.77273	3.693081
H	6.708154	-3.15057	2.665545
H	6.485403	-3.65416	4.325845
C	7.952356	-2.08601	4.078916
H	8.801856	-2.77122	4.009144
H	7.914138	-1.71402	5.107164
H	8.165463	-1.23322	3.428313
C	-1.75715	1.658668	-0.16559
H	-2.66154	1.127421	-0.47276
H	-1.2634	1.99782	-1.08047
C	-2.19432	2.87411	0.670884
H	-1.33265	3.526581	0.848539
H	-2.53208	2.530186	1.651881
C	-3.32583	3.654235	-0.00183
H	-2.96409	4.102107	-0.93555
H	-4.11973	2.95488	-0.29138
C	-3.92707	4.739578	0.892371
H	-4.33506	4.267441	1.795753
H	-3.13613	5.417933	1.236851
C	-5.02962	5.545471	0.201252
H	-5.76848	4.856847	-0.22109
H	-4.59886	6.087106	-0.64909
C	-5.72563	6.533047	1.138735
H	-6.50794	7.093225	0.619921
H	-5.01669	7.256051	1.554207
H	-6.19188	6.011477	1.981027
C	0.815	-1.16455	1.940934
C	1.324897	-0.34349	0.897977
C	-0.52365	-1.03469	2.266179
H	-0.94703	-1.6583	3.044467
C	-1.37315	-0.13983	1.587349
C	0.484755	0.577424	0.247637
H	0.903347	1.189322	-0.54226
C	-0.85768	0.696432	0.562635
C	2.662094	-0.48828	0.480872
C	-2.74761	-0.08151	1.894906
C	-3.94385	0.038802	2.077225
S	7.787289	1.213784	-3.35642

S	10.98661	-0.91977	1.900374
C	7.200683	-0.53968	-3.20531
H	7.324663	-0.92447	-4.2188
C	8.018821	-1.318	-2.22265
C	7.544791	-1.57543	-0.91509
C	8.424372	-2.05334	0.068496
H	8.062664	-2.16179	1.082899
C	9.767495	-2.26764	-0.21571
C	10.19455	-2.14455	-1.5418
C	9.32946	-1.68173	-2.52514
H	9.69951	-1.51308	-3.53006
C	10.75108	-2.46067	0.898581
H	11.71851	-2.80154	0.528635
H	10.39601	-3.18229	1.636664
C	7.505303	1.926325	-1.67245
H	6.877209	2.806026	-1.8308
H	6.917268	1.207675	-1.10082
C	8.763552	2.337236	-0.88731
C	8.316921	3.054494	0.406282
H	7.683433	2.383391	0.997656
H	7.711284	3.932746	0.154908
C	9.544304	3.482654	1.231062
H	9.207452	3.987248	2.142168
C	10.37034	2.24337	1.612995
H	11.2396	2.541356	2.211483
H	9.770094	1.566799	2.228268
C	10.83686	1.504916	0.343534
C	9.602181	1.114671	-0.48375
H	9.919283	0.58587	-1.38278
H	8.985516	0.431824	0.10028
C	9.646877	3.30166	-1.70217
H	9.065084	4.186677	-1.9862
H	9.963434	2.811678	-2.62762
C	10.8752	3.721211	-0.87914
H	11.4993	4.393891	-1.47602
C	10.41362	4.439388	0.399187
H	9.844641	5.338633	0.139121
H	11.28036	4.76402	0.985307
C	11.69379	2.474306	-0.501
H	12.58582	2.769687	0.063338
H	12.0386	1.964906	-1.40843

C	11.70003	0.276527	0.684137
H	12.64497	0.608537	1.119859
H	11.9353	-0.27819	-0.22672
H	6.138377	-0.5506	-2.97167
H	11.22807	-2.35737	-1.79239
S	-10.8667	-2.54006	1.495306
S	-7.90929	2.902547	-0.5203
C	-10.9816	-1.42243	2.968001
H	-10.6877	-2.07202	3.794714
C	-10.0846	-0.22762	2.845386
C	-10.6099	1.038273	2.568046
C	-9.78031	2.07813	2.168886
H	-10.2128	3.027074	1.87263
C	-8.40737	1.891772	2.026906
C	-7.85614	0.656715	2.443431
C	-8.70347	-0.38479	2.851454
H	-8.26915	-1.35145	3.075283
C	-7.57898	2.907467	1.302973
H	-6.51331	2.768816	1.47659
H	-7.84398	3.923832	1.598686
C	-11.4916	-1.48487	0.110488
H	-12.3213	-2.04322	-0.32882
H	-11.9055	-0.57503	0.550946
C	-10.4824	-1.12256	-0.99411
C	-11.2586	-0.43797	-2.14162
H	-11.7704	0.453046	-1.75912
H	-12.0307	-1.11587	-2.52368
C	-10.2983	-0.04334	-3.27727
H	-10.8675	0.438927	-4.07847
C	-9.24216	0.936429	-2.74155
H	-8.55936	1.231248	-3.54689
H	-9.72631	1.847245	-2.37609
C	-8.4429	0.283317	-1.59765
C	-9.41523	-0.1406	-0.48603
H	-8.85503	-0.61335	0.32026
H	-9.90162	0.745728	-0.07753
C	-9.77778	-2.3725	-1.55627
H	-10.5239	-3.08239	-1.93356
H	-9.22823	-2.87321	-0.75413
C	-8.81276	-1.97213	-2.68424
H	-8.30864	-2.86742	-3.06191

C	-9.60168	-1.30169	-3.82017
H	-10.3429	-1.99899	-4.22626
H	-8.92777	-1.03176	-4.64045
C	-7.7585	-0.99108	-2.14183
H	-7.05446	-0.72252	-2.93575
H	-7.18469	-1.46862	-1.34112
C	-7.34842	1.226364	-1.06894
H	-6.61752	1.410451	-1.85722
H	-6.81901	0.764476	-0.23477
H	-11.6829	1.19149	2.605999
H	-12.0211	-1.13003	3.120512
C	3.810415	-0.70513	0.142249
C	5.080024	-0.98494	-0.21591
C	6.213197	-1.27074	-0.54967
C	-5.27072	0.217632	2.246436
C	-6.46372	0.421545	2.359346
C	-2.07572	-1.60789	-1.52824
C	-1.21794	-0.95048	-2.48491
H	-1.64903	-0.20788	-3.14399
C	0.096393	-1.25452	-2.56884
H	0.726798	-0.75764	-3.29556
C	-1.48	-2.58087	-0.64985
H	-2.10661	-3.06509	0.087739
C	-0.16404	-2.88396	-0.73317
H	0.268417	-3.61614	-0.06425
C	0.688951	-2.24421	-1.70171
C	2.034365	-2.56391	-1.79406
C	-3.43095	-1.31893	-1.46851
C	2.624144	-3.54599	-0.95278
C	2.899702	-1.93133	-2.7266
C	-4.31818	-2.00359	-0.59558
C	-4.01153	-0.32879	-2.30517
N	3.087801	-4.34418	-0.25688
N	3.609218	-1.41603	-3.47989
N	-4.47401	0.487967	-2.98076
N	-5.0577	-2.5707	0.088747

AP6A-TCNQ-5

Electronic Energy (EE): -4834.586581 Hartree

EE + Thermal Free Energy Correction: -4833.304683 Hartree

E (Thermal): 935.464 kcal/mol

Entropy (S): 441.568 cal/mol-kelvin

Imaginary frequencies: 0

C	-1.84142	-3.03821	-0.87084
H	-2.21028	-3.22846	0.143251
H	-1.27825	-3.92503	-1.17116
C	-3.06213	-2.84435	-1.79445
H	-2.75943	-2.99999	-2.83541
H	-3.41143	-1.81147	-1.72374
C	-4.21551	-3.779	-1.42572
H	-3.8997	-4.8232	-1.54198
H	-4.44407	-3.64323	-0.3614
C	-5.48813	-3.53217	-2.23652
H	-5.79324	-2.48513	-2.11443
H	-5.27975	-3.66681	-3.3052
C	-6.64735	-4.44494	-1.82801
H	-6.83943	-4.31914	-0.75512
H	-6.34568	-5.49091	-1.95707
C	-7.92928	-4.17633	-2.6174
H	-8.74124	-4.83854	-2.30356
H	-7.77135	-4.33519	-3.68829
H	-8.26914	-3.1454	-2.48575
C	1.796414	1.538843	-0.46258
H	2.772513	1.158168	-0.14446
H	1.446055	2.211059	0.325459
C	1.991433	2.332932	-1.76584
H	1.07071	2.878232	-2.00005
H	2.159117	1.63431	-2.59055
C	3.176885	3.293167	-1.67593
H	3.022097	3.998038	-0.84933
H	4.069926	2.713113	-1.41886
C	3.440978	4.067387	-2.96778
H	3.559694	3.355934	-3.79495
H	2.563621	4.678388	-3.21487
C	4.682095	4.960659	-2.89253
H	5.559184	4.344013	-2.66791
H	4.571888	5.660409	-2.05523
C	4.936449	5.741444	-4.18263

H	5.831548	6.363393	-4.10198
H	4.093226	6.396587	-4.42286
H	5.07957	5.062402	-5.02887
C	-0.91921	-1.84837	-0.81547
C	-1.31023	-0.66989	-0.1309
C	0.342236	-1.8772	-1.39034
H	0.6657	-2.76958	-1.91205
C	1.234348	-0.79521	-1.28375
C	-0.42631	0.419521	-0.042
H	-0.74838	1.30307	0.496017
C	0.844616	0.380463	-0.59703
C	-2.59188	-0.61323	0.462105
C	2.55185	-0.91256	-1.78127
C	3.725261	-0.97301	-2.09342
S	-8.07816	2.479649	2.872747
S	-10.5595	-1.6385	-1.52305
C	-7.53979	0.823793	3.510297
H	-7.8104	0.867888	4.56658
C	-8.23572	-0.30278	2.81162
C	-7.58332	-1.05323	1.80704
C	-8.33119	-1.89905	0.972494
H	-7.82984	-2.39587	0.151188
C	-9.71007	-2.00692	1.110066
C	-10.3226	-1.37187	2.195175
C	-9.59176	-0.53705	3.030388
H	-10.0981	0.01588	3.813536
C	-10.5286	-2.64805	0.030486
H	-11.5517	-2.83532	0.357764
H	-10.0987	-3.59461	-0.30179
C	-7.51826	2.471101	1.109202
H	-6.88588	3.355139	1.000915
H	-6.88106	1.597498	0.967298
C	-8.6267	2.506315	0.043007
C	-7.96002	2.651568	-1.34316
H	-7.2757	1.811981	-1.51054
H	-7.36036	3.56845	-1.37378
C	-9.02991	2.68769	-2.44926
H	-8.53849	2.789414	-3.42204
C	-9.84424	1.383672	-2.426
H	-10.6	1.398651	-3.22048
H	-9.18947	0.528708	-2.6177

C	-10.5279	1.20948	-1.05595
C	-9.44929	1.208523	0.038446
H	-9.92057	1.080131	1.013071
H	-8.78362	0.360217	-0.11981
C	-9.57787	3.699729	0.256848
H	-9.00599	4.635264	0.249872
H	-10.0514	3.616977	1.239645
C	-10.6492	3.729305	-0.84481
H	-11.3246	4.573105	-0.67147
C	-9.96996	3.881314	-2.21545
H	-9.4064	4.819931	-2.25229
H	-10.724	3.926111	-3.00891
C	-11.4571	2.420085	-0.81398
H	-12.2404	2.446123	-1.58019
H	-11.9564	2.312318	0.156051
C	-11.3855	-0.068	-1.00394
H	-12.2422	0.044675	-1.6721
H	-11.774	-0.21582	0.005979
H	-6.45662	0.746015	3.436614
H	-11.3901	-1.48562	2.348684
S	9.321599	-2.62195	1.511682
S	8.019371	2.615605	-2.15837
C	10.05029	-3.06696	-0.13374
H	9.715281	-4.09563	-0.27926
C	9.568395	-2.15862	-1.22475
C	10.40709	-1.19214	-1.78771
C	9.882659	-0.17069	-2.56987
H	10.53526	0.614229	-2.93519
C	8.510977	-0.06225	-2.79138
C	7.678406	-1.10426	-2.32658
C	8.222373	-2.15443	-1.57048
H	7.552049	-2.90139	-1.16334
C	7.93781	1.202064	-3.35497
H	6.906685	1.073208	-3.68113
H	8.522863	1.560899	-4.2035
C	9.998712	-0.93237	1.841262
H	10.51645	-1.00919	2.79987
H	10.75134	-0.72375	1.077736
C	8.983259	0.224766	1.906186
C	9.712491	1.469828	2.459676
H	10.56238	1.715436	1.812306

H	10.11672	1.250536	3.454516
C	8.748842	2.667013	2.537187
H	9.284565	3.535905	2.931955
C	8.216998	2.990419	1.132077
H	7.539015	3.850739	1.175979
H	9.043036	3.261167	0.466887
C	7.47323	1.77224	0.555402
C	8.431738	0.571395	0.513138
H	7.907136	-0.29635	0.115312
H	9.258514	0.792895	-0.16298
C	7.801209	-0.10252	2.840026
H	8.173373	-0.34283	3.842956
H	7.280891	-0.9904	2.467873
C	6.834848	1.091071	2.908617
H	5.995019	0.844595	3.56703
C	7.57087	2.320894	3.463533
H	7.934613	2.112835	4.475503
H	6.881784	3.168371	3.534725
C	6.307109	1.410661	1.500135
H	5.587221	2.234777	1.548798
H	5.785108	0.542588	1.081003
C	6.864391	2.0886	-0.81908
H	6.159883	2.91475	-0.70448
H	6.303006	1.232218	-1.19277
H	11.46839	-1.20722	-1.56452
H	11.13812	-3.06978	-0.05822
C	-3.72982	-0.67268	0.885409
C	-5.02352	-0.77789	1.264481
C	-6.19819	-0.90145	1.551627
C	5.054572	-1.02416	-2.33286
C	6.265787	-1.04587	-2.42946
C	2.679809	-2.68388	1.070872
C	3.661427	-1.67256	1.382879
H	4.67737	-1.80207	1.032208
C	3.31149	-0.56113	2.062835
H	4.045892	0.204491	2.267573
C	1.340575	-2.51486	1.58462
H	0.610817	-3.29065	1.397752
C	0.990065	-1.39924	2.260946
H	-0.02361	-1.26976	2.615719
C	1.949495	-0.34568	2.488093

C	1.579487	0.858898	3.057802
C	3.008167	-3.76138	0.269356
C	0.232213	1.132152	3.415367
C	2.524743	1.904693	3.246035
C	2.026899	-4.70785	-0.13128
C	4.325575	-3.94414	-0.23222
N	-0.87268	1.347281	3.679191
N	3.311831	2.741702	3.374968
N	5.404749	-4.08407	-0.62072
N	1.202431	-5.44626	-0.46554

AP6A-TCNQ-6

Electronic Energy (EE): -4834.585697 Hartree

EE + Thermal Free Energy Correction: -4833.302451 Hartree

E (Thermal): 935.713 kcal/mol

Entropy (S): 439.567 cal/mol-kelvin

Imaginary frequencies: 0

C	2.371658	2.097242	1.677297
H	3.258931	1.770361	2.227957
H	2.112248	3.093951	2.043871
C	2.719789	2.18657	0.181275
H	1.849182	2.562928	-0.36481
H	2.937066	1.18293	-0.1997
C	3.912094	3.102044	-0.09667
H	3.681096	4.114433	0.256277
H	4.7742	2.760448	0.489437
C	4.29483	3.150301	-1.57712
H	4.561498	2.14122	-1.91509
H	3.421542	3.446074	-2.17141
C	5.452169	4.107986	-1.87561
H	6.28092	3.899914	-1.18896
H	5.135307	5.13459	-1.65902
C	5.952325	4.008088	-3.31737
H	6.773931	4.704147	-3.50594
H	5.152054	4.236984	-4.02769
H	6.309793	2.997928	-3.53743
C	-2.08406	-1.58238	2.674554

H	-2.80456	-1.14456	3.369952
H	-1.71267	-2.50419	3.131221
C	-2.78808	-1.92186	1.350951
H	-2.06342	-2.42064	0.701858
H	-3.07322	-0.99417	0.845239
C	-4.01507	-2.81906	1.507517
H	-3.75229	-3.69057	2.119864
H	-4.79812	-2.28116	2.054848
C	-4.55788	-3.30303	0.160836
H	-4.85084	-2.43795	-0.44808
H	-3.75071	-3.80415	-0.38679
C	-5.74005	-4.2675	0.274846
H	-6.59164	-3.75254	0.732385
H	-5.47053	-5.08084	0.958822
C	-6.14678	-4.85783	-1.07665
H	-7.00597	-5.52615	-0.97832
H	-5.32342	-5.42735	-1.51747
H	-6.41979	-4.07057	-1.78431
C	1.231136	1.153592	1.963505
C	1.436309	-0.24617	2.015002
C	-0.06232	1.629247	2.136921
H	-0.24705	2.694493	2.07926
C	-1.14862	0.765168	2.358626
C	0.355839	-1.10657	2.284397
H	0.546239	-2.17246	2.335239
C	-0.93373	-0.63251	2.461802
C	2.712102	-0.79853	1.751735
C	-2.47073	1.254699	2.465469
C	-3.65921	1.50015	2.544056
S	7.197304	-0.00644	-2.52057
S	11.15326	-1.65187	2.404613
C	6.678443	-1.74498	-2.13001
H	6.671252	-2.22234	-3.11122
C	7.630797	-2.42735	-1.19797
C	7.34409	-2.54312	0.182844
C	8.355739	-2.93006	1.072996
H	8.143767	-2.92952	2.13528
C	9.646917	-3.18785	0.623736
C	9.88328	-3.20277	-0.75377
C	8.885814	-2.83175	-1.6464
H	9.108777	-2.76784	-2.70551
C	10.78256	-3.27908	1.597944

H	11.688	-3.66061	1.124997
H	10.54523	-3.92481	2.44558
C	7.180708	0.845255	-0.87973
H	6.524956	1.704229	-1.01104
H	6.70092	0.180008	-0.16245
C	8.538317	1.327135	-0.34227
C	8.2788	2.179833	0.920067
H	7.742985	1.579437	1.664212
H	7.636659	3.031703	0.669581
C	9.609729	2.683103	1.506949
H	9.405715	3.283148	2.399358
C	10.49227	1.484121	1.891856
H	11.43749	1.836682	2.32177
H	9.993572	0.880835	2.655935
C	10.77404	0.612448	0.652691
C	9.435668	0.147773	0.059582
H	9.622404	-0.47474	-0.81581
H	8.91583	-0.4652	0.795195
C	9.283391	2.194277	-1.37507
H	8.660085	3.048622	-1.66357
H	9.46503	1.606975	-2.27985
C	10.61569	2.687989	-0.78862
H	11.14078	3.289333	-1.53754
C	10.3395	3.540502	0.460404
H	9.730318	4.410702	0.192548
H	11.27952	3.919821	0.875752
C	11.491	1.481263	-0.40492
H	12.45344	1.827935	-0.01128
H	11.70368	0.876972	-1.29454
C	11.68369	-0.58232	0.991391
H	12.6774	-0.21619	1.258916
H	11.79501	-1.22573	0.115892
H	5.661727	-1.73827	-1.74289
H	10.87232	-3.44907	-1.12427
S	-9.41991	-2.33367	1.385027
S	-8.0247	3.811181	-0.26684
C	-10.0503	-1.34823	2.822453
H	-9.68877	-1.91092	3.685403
C	-9.53802	0.06107	2.812988
C	-10.3754	1.132649	2.487351
C	-9.84291	2.38204	2.197173
H	-10.4975	3.18058	1.86678

C	-8.46642	2.601556	2.203548
C	-7.6275	1.559585	2.656785
C	-8.17992	0.307125	2.969497
H	-7.51165	-0.50492	3.229022
C	-7.89694	3.838453	1.581793
H	-6.85832	3.999237	1.866399
H	-8.47026	4.724822	1.858359
C	-10.1517	-1.46736	-0.07688
H	-10.7136	-2.23316	-0.61595
H	-10.8704	-0.73696	0.301005
C	-9.16746	-0.78465	-1.04384
C	-9.95624	-0.34421	-2.29714
H	-10.7617	0.339051	-2.00388
H	-10.4263	-1.21672	-2.76537
C	-9.02015	0.350647	-3.30117
H	-9.59667	0.65374	-4.18081
C	-8.39521	1.592996	-2.64734
H	-7.73492	2.103146	-3.35773
H	-9.1784	2.303038	-2.36474
C	-7.59415	1.190146	-1.39451
C	-8.52652	0.461778	-0.41461
H	-7.95854	0.159916	0.464475
H	-9.30658	1.148355	-0.08362
C	-8.04612	-1.74419	-1.48303
H	-8.47912	-2.64173	-1.93952
H	-7.48468	-2.06842	-0.60368
C	-7.1081	-1.04519	-2.47948
H	-6.31095	-1.73852	-2.77017
C	-7.90614	-0.62181	-3.72321
H	-8.33669	-1.50363	-4.21005
H	-7.24539	-0.13966	-4.45179
C	-6.48651	0.199666	-1.82269
H	-5.80931	0.696173	-2.52536
H	-5.89647	-0.09104	-0.94485
C	-6.91776	2.411812	-0.75061
H	-6.20742	2.839624	-1.45802
H	-6.35783	2.113343	0.135812
H	-11.4446	0.970817	2.403658
H	-11.1402	-1.38077	2.830489
C	3.79154	-1.2705	1.452826
C	4.99498	-1.75974	1.073209
C	6.074685	-2.15934	0.684937

C	-5.00111	1.648333	2.624056
C	-6.21566	1.67957	2.669923
C	-1.65929	0.729038	-1.60261
C	-2.52287	-0.26527	-2.19573
H	-3.47078	0.047628	-2.61416
C	-2.17073	-1.56938	-2.20828
H	-2.83514	-2.31004	-2.63392
C	-0.40385	0.29324	-1.04465
H	0.25512	1.026151	-0.60412
C	-0.04603	-1.00863	-1.06585
H	0.901146	-1.31729	-0.64428
C	-0.92169	-2.00714	-1.62873
C	-0.58971	-3.3497	-1.57907
C	-2.01814	2.066044	-1.57796
C	0.606201	-3.79284	-0.95095
C	-1.45217	-4.35139	-2.1011
C	-1.19856	3.045384	-0.95486
C	-3.20615	2.534199	-2.1991
N	1.574526	-4.13435	-0.41958
N	-2.17311	-5.15246	-2.51968
N	-4.16294	2.905561	-2.73098
N	-0.52233	3.827937	-0.43871

AP6A-TCNQ-7

Electronic Energy (EE): -4834.585206 Hartree

EE + Thermal Free Energy Correction: -4833.301925 Hartree

E (Thermal): 935.322 kcal/mol

Entropy (S): 438.181 cal/mol-kelvin

Imaginary frequencies: 0

C	-1.8316	2.618908	2.441939
H	-2.39179	3.263756	1.756278
H	-1.25268	3.284407	3.086583
C	-2.8543	1.812461	3.277872
H	-2.45129	1.630548	4.27925
H	-2.99895	0.8288	2.826348

C	-4.21404	2.512532	3.362332
H	-4.12719	3.427395	3.961416
H	-4.50197	2.835511	2.355684
C	-5.32519	1.625633	3.925008
H	-5.39831	0.719463	3.310345
H	-5.06516	1.290323	4.936932
C	-6.6881	2.32242	3.952083
H	-6.9108	2.69987	2.946818
H	-6.6294	3.206584	4.597381
C	-7.82184	1.412507	4.428102
H	-8.77937	1.940782	4.448876
H	-7.63038	1.04065	5.439062
H	-7.93987	0.542125	3.776128
C	1.779964	-0.423	-1.03727
H	2.535283	0.274422	-1.40761
H	1.215697	-0.76789	-1.90821
C	2.513042	-1.61647	-0.39913
H	1.79877	-2.42793	-0.22111
H	2.904292	-1.32402	0.579076
C	3.66589	-2.10312	-1.27869
H	3.288233	-2.33156	-2.28345
H	4.383359	-1.28324	-1.40409
C	4.385267	-3.33211	-0.72262
H	4.747647	-3.11654	0.29081
H	3.667815	-4.15639	-0.62039
C	5.559892	-3.78783	-1.59209
H	6.316135	-2.99544	-1.62526
H	5.21467	-3.91805	-2.62453
C	6.193582	-5.09017	-1.10164
H	7.058277	-5.36851	-1.70905
H	5.47523	-5.91474	-1.14165
H	6.531726	-4.99794	-0.06587
C	-0.88889	1.783332	1.61303
C	-1.39766	0.954337	0.577508
C	0.485119	1.854388	1.764168
H	0.906363	2.498414	2.526891
C	1.364738	1.159509	0.911207
C	-0.52001	0.220592	-0.23986
H	-0.94115	-0.39903	-1.02246
C	0.853309	0.31062	-0.10589
C	-2.77994	0.913476	0.315544

C	2.7596	1.344365	0.980294
C	3.961517	1.450827	0.823378
S	-7.89058	-1.26123	-3.32274
S	-10.7571	-0.4379	2.465414
C	-7.6227	0.542347	-2.98584
H	-7.92787	1.013361	-3.92164
C	-8.45402	1.021828	-1.83631
C	-7.88947	1.220828	-0.55525
C	-8.7233	1.395939	0.559719
H	-8.27283	1.461201	1.541414
C	-10.1061	1.357563	0.43273
C	-10.6539	1.298479	-0.85279
C	-9.83667	1.139831	-1.96501
H	-10.2827	1.01279	-2.94486
C	-10.9669	1.213641	1.651403
H	-12.0213	1.371236	1.422664
H	-10.6814	1.9142	2.438269
C	-7.26825	-2.08943	-1.7904
H	-6.50986	-2.79827	-2.13066
H	-6.75939	-1.33578	-1.1888
C	-8.31504	-2.83572	-0.94484
C	-7.57673	-3.58718	0.185301
H	-7.01376	-2.87092	0.794725
H	-6.8507	-4.28634	-0.24524
C	-8.58244	-4.35132	1.064951
H	-8.04116	-4.87534	1.859056
C	-9.576	-3.36156	1.695626
H	-10.2875	-3.89912	2.333915
H	-9.0435	-2.64904	2.332416
C	-10.335	-2.59703	0.593745
C	-9.31472	-1.86705	-0.29375
H	-9.83969	-1.31591	-1.07424
H	-8.77196	-1.14272	0.313091
C	-9.09277	-3.86286	-1.78984
H	-8.39242	-4.56928	-2.25122
H	-9.61375	-3.34668	-2.60161
C	-10.1013	-4.61955	-0.91093
H	-10.6551	-5.33487	-1.52731
C	-9.35017	-5.36668	0.202944
H	-8.65635	-6.09261	-0.2348
H	-10.0559	-5.92863	0.824568

C	-11.0895	-3.62101	-0.28349
H	-11.8281	-4.1573	0.32344
H	-11.6394	-3.0944	-1.07226
C	-11.3701	-1.62276	1.184531
H	-12.1656	-2.19246	1.669997
H	-11.8279	-1.03587	0.385233
H	-6.5597	0.729162	-2.84592
H	-11.7312	1.318156	-0.97645
S	9.370187	-1.84101	-2.68375
S	9.358606	1.400789	2.964998
C	9.549157	-0.07504	-3.22012
H	8.822194	0.012751	-4.02956
C	9.265217	0.88431	-2.10424
C	10.30223	1.558169	-1.45091
C	10.07541	2.211995	-0.24629
H	10.90642	2.658976	0.287489
C	8.817057	2.205375	0.351145
C	7.740752	1.644645	-0.37233
C	7.97643	1.000992	-1.59757
H	7.146361	0.523406	-2.10373
C	8.646805	2.632236	1.775103
H	7.603202	2.814803	2.025219
H	9.215098	3.538213	1.991858
C	10.69129	-2.02983	-1.40218
H	11.31748	-2.85622	-1.74569
H	11.30151	-1.12439	-1.42316
C	10.21819	-2.31271	0.034773
C	11.45773	-2.63783	0.897498
H	12.15674	-1.79373	0.87103
H	11.98241	-3.50595	0.48217
C	11.03713	-2.92426	2.350029
H	11.9277	-3.15192	2.944379
C	10.33535	-1.68925	2.936745
H	10.04346	-1.8817	3.976092
H	11.02037	-0.83649	2.94557
C	9.088836	-1.33672	2.102177
C	9.516566	-1.09056	0.647334
H	8.638376	-0.84886	0.04871
H	10.18662	-0.23163	0.611723
C	9.256083	-3.5139	0.087154
H	9.740896	-4.40099	-0.33758

H	8.378566	-3.30066	-0.52704
C	8.8306	-3.78966	1.537799
H	8.137519	-4.63627	1.557119
C	10.07273	-4.12065	2.380261
H	10.56558	-5.01554	1.984865
H	9.782185	-4.34153	3.413113
C	8.128974	-2.54765	2.115994
H	7.797136	-2.74882	3.141007
H	7.23483	-2.31455	1.525934
C	8.345698	-0.12299	2.687407
H	7.944201	-0.3858	3.668744
H	7.500757	0.148488	2.053531
H	11.3056	1.520819	-1.86091
H	10.54494	0.074071	-3.63867
C	-3.97496	0.956226	0.092377
C	-5.30071	1.039792	-0.13711
C	-6.49284	1.143427	-0.34932
C	5.27397	1.547482	0.527425
C	6.424868	1.627718	0.145521
C	1.559977	3.24499	-1.76975
C	0.870319	2.415603	-2.72742
H	1.456532	1.81208	-3.40854
C	-0.4814	2.386371	-2.78193
H	-0.98481	1.760006	-3.50774
C	0.758661	4.046614	-0.88019
H	1.26067	4.669884	-0.15176
C	-0.59172	4.011488	-0.92967
H	-1.17612	4.608969	-0.24233
C	-1.28252	3.178768	-1.88048
C	-2.66827	3.143533	-1.91599
C	2.94476	3.27854	-1.69622
C	-3.44913	3.933903	-1.02915
C	-3.38544	2.316468	-2.82103
C	3.619211	4.171328	-0.81944
C	3.766265	2.431373	-2.48712
N	-4.06453	4.581151	-0.29495
N	-3.9794	1.642408	-3.54886
N	4.437519	1.723138	-3.10797
N	4.154609	4.918289	-0.11837

AP6A-TCNQ-8

Electronic Energy (EE): -4834.581995 Hartree

EE + Thermal Free Energy Correction: -4833.301228 Hartree

E (Thermal): 935.279 kcal/mol

Entropy (S): 443.327 cal/mol-kelvin

Imaginary frequencies: 0

C	3.495582	1.123846	1.450193
H	4.3541	0.444547	1.447221
H	3.117883	1.153286	2.476231
C	3.978957	2.526231	1.039355
H	3.153893	3.238856	1.149775
H	4.241723	2.518146	-0.02194
C	5.18771	2.993179	1.851678
H	4.910361	3.081388	2.909596
H	5.972169	2.227532	1.805884
C	5.764611	4.322403	1.360505
H	6.096657	4.202962	0.321009
H	4.973346	5.08252	1.338308
C	6.933339	4.826344	2.210496
H	7.678548	4.030024	2.303855
H	6.575982	5.029027	3.227035
C	7.590187	6.083445	1.638387
H	8.419445	6.422999	2.264587
H	6.87301	6.906385	1.559128
H	7.985244	5.89735	0.63422
C	-0.65986	-1.02293	-2.09111
H	-1.27763	-1.71107	-1.50746
H	-0.20846	-1.61135	-2.89391
C	-1.55605	0.08386	-2.68972
H	-1.07054	0.504498	-3.57718
H	-1.6459	0.905471	-1.97415
C	-2.96266	-0.40608	-3.0374
H	-2.90399	-1.25365	-3.73181
H	-3.43254	-0.78824	-2.12296
C	-3.84074	0.691169	-3.64129
H	-3.81718	1.571737	-2.98652
H	-3.41003	1.013364	-4.59766
C	-5.29736	0.273896	-3.85819

H	-5.75888	0.054805	-2.8883
H	-5.32794	-0.66006	-4.43233
C	-6.12064	1.342531	-4.57782
H	-7.16645	1.043788	-4.67411
H	-5.72605	1.53133	-5.58082
H	-6.09469	2.28952	-4.03028
C	2.427992	0.568643	0.542682
C	2.755918	0.075913	-0.74626
C	1.097704	0.534977	0.930357
H	0.81823	0.902897	1.910523
C	0.093173	0.028076	0.08847
C	1.751731	-0.44309	-1.58311
H	2.033493	-0.82364	-2.5576
C	0.422342	-0.48033	-1.19513
C	4.099509	0.124583	-1.18208
C	-1.25752	0.010492	0.495421
C	-2.45368	-0.05199	0.706832
S	9.504962	1.89319	1.561196
S	12.14623	-2.3144	-2.65243
C	9.029992	2.601494	-0.08487
H	9.299667	3.65404	0.01555
C	9.767216	1.951182	-1.21467
C	9.149263	0.967836	-2.02378
C	9.932829	0.178406	-2.87814
H	9.457763	-0.6282	-3.42314
C	11.31353	0.333484	-2.93412
C	11.89425	1.396332	-2.23606
C	11.12717	2.192053	-1.39465
H	11.6066	2.957315	-0.79463
C	12.16414	-0.71413	-3.58689
H	13.19465	-0.37838	-3.70705
H	11.78353	-0.9982	-4.56987
C	8.953323	0.130377	1.461942
H	8.294098	-0.01586	2.320881
H	8.34559	0.021919	0.563307
C	10.06603	-0.93172	1.49099
C	9.402123	-2.32497	1.561239
H	8.74558	-2.46248	0.694634
H	8.774172	-2.39363	2.456956
C	10.47629	-3.42722	1.588887
H	9.986935	-4.40508	1.637103

C	11.33206	-3.35017	0.313605
H	12.09123	-4.14147	0.322917
H	10.70658	-3.51139	-0.56912
C	12.01369	-1.97177	0.214663
C	10.92945	-0.88266	0.220468
H	11.39863	0.098525	0.143381
H	10.29191	-1.01257	-0.65363
C	10.97717	-0.75993	2.721698
H	10.37555	-0.80536	3.637402
H	11.44785	0.227288	2.691896
C	12.05262	-1.85764	2.743541
H	12.69959	-1.7143	3.614824
C	11.37624	-3.23592	2.820402
H	10.78302	-3.31157	3.738203
H	12.13278	-4.0273	2.858918
C	12.90198	-1.77275	1.463346
H	13.68833	-2.536	1.48536
H	13.3991	-0.79714	1.409251
C	12.91381	-1.86785	-1.02997
H	13.76586	-2.54156	-0.91533
H	13.3074	-0.85315	-1.12096
H	7.950539	2.535505	-0.2056
H	12.96368	1.56216	-2.30611
S	-9.18992	0.192243	-2.36177
S	-6.95393	2.254371	3.392992
C	-9.22833	-1.33497	-1.3177
H	-8.78124	-2.08749	-1.96999
C	-8.46098	-1.1631	-0.04196
C	-9.1277	-1.01189	1.180425
C	-8.44492	-0.57468	2.308422
H	-8.99198	-0.37841	3.223547
C	-7.08486	-0.27781	2.26147
C	-6.37996	-0.56363	1.065666
C	-7.08189	-1.0023	-0.06878
H	-6.54062	-1.13225	-0.99744
C	-6.43122	0.475322	3.377025
H	-5.3462	0.410919	3.338652
H	-6.75815	0.111793	4.352146
C	-10.0573	1.450286	-1.31798
H	-10.8783	1.821617	-1.93497
H	-10.4983	0.92673	-0.46702

C	-9.20793	2.637319	-0.82935
C	-10.1523	3.673855	-0.18126
H	-10.6973	3.208605	0.648379
H	-10.8981	4.005423	-0.91268
C	-9.34732	4.881284	0.331855
H	-10.0329	5.603378	0.786237
C	-8.32993	4.413206	1.384613
H	-7.76186	5.270344	1.765112
H	-8.85012	3.965458	2.23667
C	-7.36553	3.380317	0.770698
C	-8.18246	2.198217	0.227159
H	-7.50605	1.470442	-0.21961
H	-8.6984	1.708544	1.053594
C	-8.45984	3.315591	-1.9926
H	-9.1757	3.647121	-2.75419
H	-7.79528	2.590304	-2.46793
C	-7.64895	4.514205	-1.47445
H	-7.11161	4.974861	-2.30943
C	-8.60259	5.53978	-0.84092
H	-9.31718	5.899315	-1.58925
H	-8.04106	6.410775	-0.48612
C	-6.63297	4.036643	-0.42114
H	-6.03333	4.883836	-0.06947
H	-5.94061	3.314103	-0.86869
C	-6.30929	2.92025	1.790706
H	-5.67813	3.769087	2.063077
H	-5.65938	2.16227	1.352229
H	-10.1962	-1.18792	1.231426
H	-10.263	-1.62205	-1.12966
C	5.272829	0.253394	-1.47338
C	6.583376	0.464525	-1.73157
C	7.763833	0.700312	-1.90034
C	-3.78632	-0.18307	0.86463
C	-4.98788	-0.34695	0.969207
C	-8.11379	-4.38955	0.23812
C	-6.93541	-4.55333	-0.57846
H	-7.05136	-4.90981	-1.59415
C	-5.712	-4.22928	-0.10446
H	-4.83812	-4.32324	-0.73664
C	-7.93578	-3.96457	1.603063
H	-8.80642	-3.87933	2.23854

C	-6.71089	-3.6484	2.079804
H	-6.59419	-3.30844	3.099807
C	-5.54433	-3.72898	1.239134
C	-4.30465	-3.31137	1.693549
C	-9.37955	-4.59111	-0.29079
C	-4.14616	-2.76859	2.997629
C	-3.14698	-3.38128	0.873932
C	-10.5439	-4.30603	0.469453
C	-9.57048	-4.99924	-1.63735
N	-4.05753	-2.31953	4.058961
N	-2.21678	-3.44543	0.190767
N	-9.70528	-5.31945	-2.74039
N	-11.4725	-4.02473	1.099059

AP6A-TCNQ-9

Electronic Energy (EE): -4834.581708 Hartree

EE + Thermal Free Energy Correction: -4833.300807 Hartree

E (Thermal): 935.382 kcal/mol

Entropy (S): 443.390 cal/mol-kelvin

Imaginary frequencies: 0

C	3.28245	0.084942	1.617799
H	4.171858	-0.44489	1.261716
H	2.876268	-0.4991	2.448714
C	3.709724	1.473418	2.125939
H	2.846343	1.972211	2.580443
H	4.010295	2.089201	1.273884
C	4.863124	1.405632	3.127999
H	4.543215	0.857769	4.023254
H	5.686999	0.825287	2.694454
C	5.388631	2.784681	3.5318
H	5.766498	3.29647	2.637191
H	4.560178	3.400901	3.903758
C	6.495671	2.726501	4.587064
H	7.276984	2.038591	4.248865
H	6.089038	2.295687	5.509692
C	7.107596	4.095593	4.88733

H	7.892134	4.026651	5.645535
H	6.351739	4.798117	5.25204
H	7.551805	4.532697	3.986961
C	-0.67671	0.333482	-2.70346
H	-1.28424	-0.57378	-2.66319
H	-0.17803	0.328628	-3.676
C	-1.6032	1.565291	-2.59679
H	-1.11598	2.428765	-3.06312
H	-1.74498	1.826989	-1.54462
C	-2.97715	1.327232	-3.2257
H	-2.8602	0.980668	-4.25992
H	-3.46859	0.510391	-2.68396
C	-3.87074	2.567857	-3.20281
H	-3.89678	2.978786	-2.1852
H	-3.42139	3.346573	-3.83199
C	-5.3048	2.304516	-3.66986
H	-5.80117	1.635272	-2.95748
H	-5.28514	1.77083	-4.62746
C	-6.12556	3.585865	-3.81818
H	-7.16094	3.367823	-4.08859
H	-5.70144	4.23433	-4.59088
H	-6.13751	4.154152	-2.88318
C	2.266317	0.142213	0.505937
C	2.650254	0.502872	-0.81036
C	0.928163	-0.13742	0.737303
H	0.607013	-0.41494	1.734447
C	-0.03067	-0.07611	-0.28798
C	1.691794	0.553406	-1.83804
H	2.01507	0.821582	-2.83677
C	0.354822	0.269972	-1.60839
C	4.002689	0.820628	-1.07247
C	-1.39233	-0.34651	-0.02413
C	-2.59061	-0.52095	0.086799
S	9.175853	0.747073	2.47767
S	12.19038	-0.28218	-3.20136
C	8.762411	2.273483	1.51021
H	8.989564	3.076553	2.213207
C	9.580556	2.389283	0.261129
C	9.042544	2.045113	-1.00206
C	9.898361	1.888291	-2.10205
H	9.482744	1.538162	-3.03907

C	11.27345	2.048862	-1.97091
C	11.77703	2.521248	-0.75545
C	10.93799	2.691422	0.33845
H	11.35662	2.976034	1.297249
C	12.19565	1.5649	-3.04905
H	13.21757	1.910253	-2.88934
H	11.87756	1.894634	-4.03998
C	8.696201	-0.63857	1.350035
H	7.99705	-1.25106	1.924398
H	8.142944	-0.21113	0.513644
C	9.845161	-1.5253	0.839662
C	9.232413	-2.70827	0.057296
H	8.629414	-2.32526	-0.77376
H	8.56011	-3.27765	0.709398
C	10.34513	-3.62723	-0.47827
H	9.891985	-4.45717	-1.02956
C	11.26439	-2.83328	-1.42125
H	12.05206	-3.48664	-1.81534
H	10.69362	-2.46104	-2.27689
C	11.89667	-1.64558	-0.67
C	10.77294	-0.75679	-0.11454
H	11.20795	0.091527	0.414457
H	10.18877	-0.36269	-0.94564
C	10.6816	-2.08832	2.004687
H	10.03389	-2.65059	2.687987
H	11.11436	-1.26133	2.575425
C	11.79612	-2.99957	1.466818
H	12.38952	-3.3802	2.304221
C	11.17003	-4.17436	0.69789
H	10.53198	-4.76222	1.366713
H	11.95318	-4.84465	0.327055
C	12.70879	-2.19618	0.523798
H	13.52177	-2.83367	0.157647
H	13.16993	-1.36462	1.069437
C	12.85622	-0.84651	-1.57057
H	13.72536	-1.46387	-1.80831
H	13.2165	0.03713	-1.03927
H	7.693727	2.290246	1.305843
H	12.84173	2.698274	-0.64959
S	-9.2072	1.336814	-2.62986
S	-7.20828	0.304046	3.480621

C	-9.2868	-0.50508	-2.46989
H	-8.80259	-0.85839	-3.38236
C	-8.57832	-0.98479	-1.23939
C	-9.28663	-1.44768	-0.12649
C	-8.64738	-1.62247	1.095057
H	-9.22625	-1.90375	1.967182
C	-7.28809	-1.34806	1.247071
C	-6.53936	-1.01984	0.094173
C	-7.19878	-0.84034	-1.13145
H	-6.62869	-0.49671	-1.98528
C	-6.68574	-1.24818	2.611979
H	-5.59867	-1.30184	2.586987
H	-7.05537	-2.03261	3.27449
C	-10.1262	1.937721	-1.13979
H	-10.9247	2.574101	-1.52725
H	-10.5963	1.071471	-0.66915
C	-9.30585	2.724512	-0.10173
C	-10.2805	3.326029	0.934673
H	-10.8537	2.522951	1.41259
H	-10.9999	3.981887	0.431259
C	-9.50243	4.119844	1.999576
H	-10.2088	4.538394	2.723314
C	-8.52338	3.184164	2.727095
H	-7.97548	3.739488	3.497557
H	-9.07342	2.38511	3.233156
C	-7.53015	2.568456	1.722825
C	-8.31809	1.81285	0.642397
H	-7.61982	1.388942	-0.07802
H	-8.86116	0.987706	1.104237
C	-8.51742	3.872795	-0.75826
H	-9.20468	4.543177	-1.28809
H	-7.83147	3.460735	-1.50127
C	-7.73354	4.655878	0.306619
H	-7.16722	5.457392	-0.17818
C	-8.71674	5.255981	1.324442
H	-9.40391	5.944415	0.820697
H	-8.1745	5.835871	2.079139
C	-6.75583	3.712178	1.029822
H	-6.1752	4.272542	1.771359
H	-6.04273	3.290079	0.312162
C	-6.51009	1.654758	2.424794

H	-5.89154	2.254317	3.096337
H	-5.84304	1.195609	1.694155
H	-10.3556	-1.61372	-0.20111
H	-10.3284	-0.82737	-2.48629
C	5.178043	1.112349	-1.1789
C	6.487603	1.449889	-1.20125
C	7.664013	1.749318	-1.13953
C	-3.93104	-0.68373	0.14059
C	-5.13847	-0.82693	0.148746
C	-5.57886	-3.71696	-1.50505
C	-4.66282	-3.97206	-0.41823
H	-3.60727	-3.7921	-0.57422
C	-5.11703	-4.36784	0.790129
H	-4.42729	-4.51442	1.611302
C	-6.97805	-4.00342	-1.2962
H	-7.66578	-3.8669	-2.1192
C	-7.43182	-4.39891	-0.08814
H	-8.48607	-4.58223	0.066467
C	-6.52916	-4.54912	1.02842
C	-7.00647	-4.82794	2.296757
C	-5.13684	-3.15881	-2.6905
C	-8.40068	-4.91364	2.556799
C	-6.13506	-4.97401	3.409876
C	-6.05435	-2.78081	-3.70797
C	-3.76756	-2.85699	-2.91565
N	-9.54011	-4.94724	2.750097
N	-5.41555	-5.08233	4.308229
N	-2.65083	-2.61773	-3.0926
N	-6.8294	-2.44703	-4.49832

AP6A-TCNQ-10

Electronic Energy (EE): -4834.580742 Hartree

EE + Thermal Free Energy Correction: -4833.298533 Hartree

E (Thermal): 935.257 kcal/mol

Entropy (S): 440.220 cal/mol-kelvin

Imaginary frequencies: 0

C	3.170234	-1.40004	3.296966
H	3.82691	-2.27579	3.311543
H	2.624499	-1.39996	4.244213
C	4.048078	-0.13501	3.197642
H	3.465791	0.738299	3.511252
H	4.32164	0.034828	2.153556
C	5.328283	-0.25031	4.026744
H	5.075804	-0.3703	5.087879
H	5.852735	-1.16677	3.729697
C	6.275685	0.937737	3.855017
H	6.522191	1.047927	2.79135
H	5.765325	1.864101	4.146927
C	7.570525	0.799126	4.659751
H	8.07128	-0.13405	4.373443
H	7.32533	0.692895	5.722945
C	8.524598	1.978706	4.466177
H	9.443816	1.852496	5.04528
H	8.060117	2.915742	4.787406
H	8.803844	2.096095	3.415395
C	-0.57631	-2.02308	-1.17282
H	-1.43131	-2.60878	-0.82421
H	-0.10906	-2.59777	-1.97682
C	-1.07616	-0.66984	-1.72132
H	-0.31145	-0.24016	-2.37792
H	-1.19991	0.034598	-0.89455
C	-2.40774	-0.78269	-2.46534
H	-2.31887	-1.51111	-3.28122
H	-3.16001	-1.18345	-1.77445
C	-2.8904	0.556054	-3.02615
H	-2.93096	1.293597	-2.21439
H	-2.1502	0.934171	-3.7425
C	-4.25908	0.488851	-3.70805
H	-5.01666	0.197502	-2.97092
H	-4.24726	-0.30154	-4.46822
C	-4.6679	1.813626	-4.35255
H	-5.6573	1.74996	-4.80972
H	-3.95287	2.107895	-5.12697
H	-4.70163	2.615234	-3.60919
C	2.198445	-1.55416	2.155634
C	2.663119	-1.92925	0.868192
C	0.840079	-1.33492	2.316998

H	0.457002	-1.04839	3.28925
C	-0.06416	-1.47766	1.25004
C	1.756074	-2.0881	-0.19364
H	2.137303	-2.38722	-1.16272
C	0.396671	-1.8711	-0.03321
C	4.050978	-2.11321	0.671107
C	-1.4416	-1.22747	1.424612
C	-2.63973	-1.018	1.419911
S	9.796763	-2.42762	-2.88803
S	11.09725	2.090793	1.624045
C	9.586633	-3.35782	-1.29769
H	10.16015	-4.27015	-1.47004
C	10.11062	-2.59313	-0.12233
C	9.234567	-1.91437	0.754918
C	9.749757	-0.97864	1.66589
H	9.057434	-0.40207	2.266501
C	11.11035	-0.69854	1.715924
C	11.9795	-1.47675	0.944911
C	11.48175	-2.40932	0.044122
H	12.16384	-2.94271	-0.60837
C	11.60476	0.511346	2.449601
H	12.68903	0.498356	2.564014
H	11.15872	0.599434	3.441633
C	8.739279	-0.92607	-2.6602
H	8.047073	-0.92805	-3.50524
H	8.147549	-1.07039	-1.75554
C	9.474798	0.424385	-2.61105
C	8.416662	1.549985	-2.58558
H	7.765352	1.421118	-1.7136
H	7.781362	1.483927	-3.47626
C	9.10263	2.926956	-2.53212
H	8.337726	3.709701	-2.51354
C	9.963536	3.028108	-1.26204
H	10.44509	4.012091	-1.2121
H	9.333929	2.929906	-0.37296
C	11.03456	1.920182	-1.25733
C	10.3348	0.554983	-1.34444
H	11.08295	-0.23789	-1.33636
H	9.704773	0.422174	-0.46515
C	10.37261	0.623579	-3.84737
H	9.769618	0.54556	-4.75991

H	11.12227	-0.17229	-3.88568
C	11.06189	1.996017	-3.78761
H	11.70631	2.116806	-4.66418
C	9.996306	3.103739	-3.77039
H	9.39226	3.05676	-4.68303
H	10.47463	4.089111	-3.75025
C	11.91921	2.087579	-2.51307
H	12.43478	3.054215	-2.47816
H	12.69106	1.309167	-2.52561
C	11.94347	2.010239	-0.01811
H	12.54826	2.91775	-0.07894
H	12.62842	1.159914	0.004333
H	8.539335	-3.62812	-1.17575
H	13.04877	-1.30714	1.008167
S	-8.06301	1.663416	-2.71063
S	-6.99682	1.939264	3.703973
C	-8.7717	0.071422	-2.08955
H	-8.38055	-0.66108	-2.79822
C	-8.34848	-0.22067	-0.68177
C	-9.25187	-0.09462	0.380948
C	-8.8002	-0.08574	1.694649
H	-9.50076	0.100154	2.500823
C	-7.44493	-0.20233	1.995398
C	-6.54637	-0.46914	0.93304
C	-7.01499	-0.47489	-0.39065
H	-6.29949	-0.593	-1.19454
C	-6.94013	0.116835	3.367457
H	-5.93214	-0.25655	3.533883
H	-7.58748	-0.29983	4.140234
C	-8.81919	2.936004	-1.60113
H	-9.31981	3.640405	-2.26882
H	-9.59195	2.441832	-1.00829
C	-7.85439	3.704174	-0.67977
C	-8.63901	4.859745	-0.01945
H	-9.48916	4.454468	0.541576
H	-9.04721	5.520699	-0.79249
C	-7.72158	5.658333	0.923194
H	-8.2951	6.47094	1.379988
C	-7.18418	4.731978	2.02575
H	-6.54049	5.296946	2.710294
H	-8.01304	4.331283	2.617131

C	-6.38787	3.56929	1.402807
C	-7.30125	2.802097	0.434247
H	-6.73817	1.988101	-0.02049
H	-8.12878	2.362541	0.992207
C	-6.67044	4.301114	-1.46487
H	-7.04263	4.95703	-2.26079
H	-6.1108	3.494189	-1.94595
C	-5.75108	5.089804	-0.51812
H	-4.90807	5.494277	-1.08708
C	-6.54414	6.239698	0.123622
H	-6.91255	6.919939	-0.65191
H	-5.89506	6.824555	0.784256
C	-5.21704	4.156792	0.583211
H	-4.5396	4.709093	1.244296
H	-4.6377	3.340928	0.135585
C	-5.79855	2.647174	2.484217
H	-5.07623	3.209164	3.080491
H	-5.26144	1.816338	2.025824
H	-10.3058	0.049674	0.171983
H	-9.8572	0.090739	-2.18816
C	5.264437	-2.17395	0.622602
C	6.61675	-2.15157	0.629335
C	7.829503	-2.08015	0.676197
C	-3.96809	-0.81215	1.31465
C	-5.16517	-0.65056	1.16497
C	-8.86757	-3.40428	-1.13505
C	-7.58995	-3.7577	-1.70518
H	-7.51415	-3.87802	-2.77846
C	-6.49548	-3.89479	-0.92425
H	-5.53422	-4.12561	-1.36567
C	-8.96174	-3.29999	0.29867
H	-9.9229	-3.0787	0.741782
C	-7.86845	-3.44477	1.080512
H	-7.94845	-3.34079	2.153993
C	-6.57524	-3.71105	0.505271
C	-5.43758	-3.75795	1.29347
C	-9.95719	-3.12854	-1.94695
C	-5.505	-3.51901	2.692979
C	-4.15597	-4.01727	0.739505
C	-11.1772	-2.65418	-1.39777
C	-9.87603	-3.21635	-3.36186

N	-5.59631	-3.30475	3.825114
N	-3.12195	-4.23016	0.268759
N	-9.78589	-3.28111	-4.51299
N	-12.1434	-2.2294	-0.92464

AP6A-TCNQ-11

Electronic Energy (EE): -4834.580450 Hartree

EE + Thermal Free Energy Correction: -4833.298234 Hartree

E (Thermal): 935.417 kcal/mol

Entropy (S): 440.742 cal/mol-kelvin

Imaginary frequencies: 0

C	-2.90806	-3.1967	-1.39058
H	-3.56837	-3.89144	-0.86155
H	-2.29644	-3.79835	-2.06816
C	-3.7825	-2.21765	-2.20216
H	-3.17841	-1.75684	-2.99129
H	-4.11362	-1.40425	-1.55226
C	-5.01495	-2.89887	-2.79943
H	-4.70462	-3.68313	-3.50146
H	-5.55609	-3.40595	-1.99126
C	-5.97	-1.93099	-3.49895
H	-6.27204	-1.15092	-2.78866
H	-5.44577	-1.41554	-4.3134
C	-7.22092	-2.61558	-4.05576
H	-7.73364	-3.14015	-3.23992
H	-6.92058	-3.39238	-4.76866
C	-8.18667	-1.64111	-4.73149
H	-9.07387	-2.15394	-5.11389
H	-7.7085	-1.13737	-5.57683
H	-8.52129	-0.86612	-4.03635
C	0.504787	-0.56558	2.587359
H	1.374206	-1.20129	2.770802
H	-0.02291	-0.48057	3.540704
C	0.987756	0.829115	2.130242
H	0.21996	1.57486	2.364476

H	1.099892	0.839056	1.042545
C	2.324277	1.22855	2.758118
H	2.263433	1.148412	3.850467
H	3.085334	0.505753	2.441097
C	2.767274	2.640991	2.374773
H	2.722529	2.752205	1.283551
H	2.050986	3.365642	2.782242
C	4.176705	2.998661	2.853959
H	4.907202	2.366603	2.335693
H	4.270866	2.763062	3.920637
C	4.526211	4.469196	2.624256
H	5.556783	4.684994	2.913812
H	3.865917	5.122674	3.202577
H	4.413194	4.738539	1.569765
C	-2.02163	-2.52062	-0.37656
C	-2.58161	-1.96804	0.80388
C	-0.65212	-2.41144	-0.55833
H	-0.19826	-2.82333	-1.45204
C	0.172311	-1.77986	0.388934
C	-1.7543	-1.3502	1.757373
H	-2.20701	-0.94599	2.654946
C	-0.38387	-1.24319	1.578115
C	-3.98266	-2.02524	0.987317
C	1.562671	-1.65333	0.170774
C	2.757452	-1.44713	0.078165
S	-9.79232	0.087523	3.534356
S	-10.9157	0.309218	-2.88175
C	-9.58129	-1.68113	3.01789
H	-10.1836	-2.2231	3.748959
C	-10.063	-1.92408	1.621458
C	-9.15346	-2.02987	0.54442
C	-9.62827	-1.96801	-0.77531
H	-8.91076	-1.95959	-1.58631
C	-10.9809	-1.79285	-1.04445
C	-11.8833	-1.83096	0.02311
C	-11.4258	-1.89862	1.332554
H	-12.133	-1.83997	2.152222
C	-11.4344	-1.40931	-2.42045
H	-12.5157	-1.49884	-2.52769
H	-10.9638	-2.0209	-3.19219
C	-8.69244	1.017075	2.372016

H	-8.01395	1.588688	3.009333
H	-8.09051	0.287549	1.829459
C	-9.39173	1.973459	1.390693
C	-8.3043	2.763177	0.628263
H	-7.6469	2.06402	0.098979
H	-7.68088	3.318649	1.338259
C	-8.95348	3.736369	-0.37203
H	-8.1682	4.284625	-0.90204
C	-9.79721	2.949259	-1.38807
H	-10.2524	3.63673	-2.11115
H	-9.16058	2.261047	-1.95146
C	-10.8965	2.151516	-0.66004
C	-10.2335	1.209805	0.356866
H	-11.0016	0.634964	0.874635
H	-9.59704	0.504083	-0.17643
C	-10.2979	2.976378	2.130351
H	-9.70739	3.538423	2.863677
H	-11.0685	2.431644	2.683906
C	-10.9504	3.942171	1.128609
H	-11.6012	4.6385	1.667006
C	-9.85591	4.72439	0.384682
H	-9.26349	5.308939	1.096957
H	-10.308	5.433189	-0.31775
C	-11.7908	3.144514	0.11593
H	-12.2804	3.829807	-0.58557
H	-12.5828	2.595398	0.638409
C	-11.7872	1.375509	-1.64763
H	-12.374	2.082757	-2.23794
H	-12.489	0.742998	-1.0999
H	-8.54018	-1.97069	3.147662
H	-12.9467	-1.74617	-0.17176
S	8.209044	3.197319	1.905782
S	7.015287	-0.10768	-3.57464
C	8.909697	1.522876	2.266608
H	8.521118	1.296824	3.261543
C	8.473219	0.509235	1.252502
C	9.357359	0.021586	0.285506
C	8.883676	-0.69186	-0.809
H	9.571593	-0.99279	-1.59067
C	7.521877	-0.9456	-0.9739
C	6.642555	-0.58106	0.070808

C	7.133393	0.144034	1.167364
H	6.434016	0.498884	1.91374
C	6.998989	-1.43922	-2.28486
H	5.994334	-1.84972	-2.19904
H	7.648539	-2.20249	-2.71659
C	8.953983	3.633423	0.268318
H	9.470371	4.582648	0.426701
H	9.712115	2.881556	0.038573
C	7.975409	3.775546	-0.91147
C	8.746018	4.356773	-2.11719
H	9.581373	3.695361	-2.37517
H	9.17391	5.329998	-1.85088
C	7.805935	4.509815	-3.32664
H	8.368911	4.922996	-4.16941
C	7.240198	3.135059	-3.71783
H	6.580386	3.234309	-4.5879
H	8.053763	2.461508	-4.00345
C	6.458874	2.525976	-2.53794
C	7.395066	2.414702	-1.32528
H	6.842455	1.998418	-0.48425
H	8.208919	1.728256	-1.56097
C	6.81143	4.724307	-0.56922
H	7.20284	5.707039	-0.2803
H	6.264151	4.329557	0.289563
C	5.8696	4.866125	-1.77551
H	5.040815	5.530292	-1.51065
C	6.647904	5.453893	-2.96339
H	7.034759	6.44538	-2.70415
H	5.984588	5.581483	-3.8257
C	5.307938	3.485465	-2.15993
H	4.613951	3.585606	-3.00218
H	4.740582	3.065153	-1.3214
C	5.846198	1.164237	-2.90937
H	5.099789	1.307088	-3.69392
H	5.333494	0.729514	-2.05061
H	10.41408	0.253636	0.358066
H	9.996386	1.588329	2.328497
C	-5.19747	-2.0757	0.992991
C	-6.54692	-2.09572	0.901501
C	-7.75465	-2.09241	0.762502
C	4.078003	-1.16457	0.029822

C	5.263112	-0.89282	0.02078
C	6.543587	-2.88842	2.393885
C	5.835333	-3.72939	1.457609
H	4.772979	-3.88191	1.595593
C	6.47003	-4.26626	0.393643
H	5.921686	-4.85936	-0.32685
C	7.968036	-2.73011	2.222595
H	8.517684	-2.14594	2.947564
C	8.60223	-3.26708	1.159004
H	9.664399	-3.11753	1.023375
C	7.873305	-4.0175	0.164249
C	8.494441	-4.45901	-0.99054
C	5.867233	-2.20731	3.389304
C	9.850329	-4.13669	-1.26706
C	7.793188	-5.19393	-1.98443
C	6.539751	-1.27424	4.224205
C	4.464752	-2.33751	3.570475
N	10.94506	-3.83523	-1.48499
N	7.208796	-5.78392	-2.78872
N	3.323649	-2.45192	3.713389
N	7.10729	-0.49205	4.858899

AP6A-TCNQ-12

Electronic Energy (EE): -4834.580071 Hartree

EE + Thermal Free Energy Correction: -4833.298703 Hartree

E (Thermal): 935.385 kcal/mol

Entropy (S): 442.417 cal/mol-kelvin

Imaginary frequencies: 0

C	2.035078	2.597413	-1.42114
H	2.705225	3.147616	-0.75571
H	1.508719	3.339736	-2.02765
C	2.902223	1.703585	-2.33122
H	2.315279	1.382717	-3.19841
H	3.177647	0.795941	-1.78888
C	4.179267	2.417874	-2.7783
H	3.921972	3.296403	-3.38294

H	4.693408	2.799898	-1.88817
C	5.1406	1.519529	-3.55718
H	5.39361	0.647266	-2.94102
H	4.640823	1.12646	-4.45134
C	6.429689	2.234093	-3.97125
H	6.913552	2.644854	-3.07647
H	6.178233	3.097753	-4.59763
C	7.406551	1.32309	-4.71599
H	8.318733	1.856327	-4.99837
H	6.957172	0.932565	-5.6339
H	7.697051	0.46535	-4.10297
C	-1.79351	-0.33928	1.899484
H	-2.63154	0.325545	2.125024
H	-1.3333	-0.60206	2.854894
C	-2.37738	-1.60265	1.24196
H	-1.60099	-2.37312	1.18105
H	-2.67742	-1.37574	0.21476
C	-3.58577	-2.12077	2.024355
H	-3.3044	-2.25947	3.075847
H	-4.36504	-1.3484	2.017611
C	-4.16555	-3.42937	1.487168
H	-4.43459	-3.30686	0.430178
H	-3.39263	-4.20782	1.517574
C	-5.39319	-3.90194	2.271255
H	-6.19521	-3.16236	2.166602
H	-5.14684	-3.93111	3.339211
C	-5.89553	-5.27699	1.830197
H	-6.79881	-5.56562	2.3735
H	-5.13747	-6.04628	2.006348
H	-6.13334	-5.28644	0.763428
C	1.044179	1.838544	-0.58027
C	1.487737	1.064833	0.524575
C	-0.31683	1.882168	-0.84067
H	-0.68141	2.47886	-1.66838
C	-1.24746	1.195201	-0.04431
C	0.558972	0.362055	1.308653
H	0.920693	-0.22184	2.146784
C	-0.80385	0.408202	1.049253
C	2.871267	1.020203	0.810969
C	-2.63293	1.335513	-0.27536
C	-3.84158	1.449519	-0.34023

S	8.705665	-1.29294	3.452695
S	10.0817	-0.70829	-2.89185
C	8.401766	0.506857	3.125624
H	8.935401	0.997517	3.941355
C	8.925426	0.935089	1.790348
C	8.05633	1.119473	0.691255
C	8.585501	1.235475	-0.60381
H	7.902607	1.28603	-1.4426
C	9.955344	1.165201	-0.82826
C	10.8102	1.127076	0.277842
C	10.29782	1.016047	1.563893
H	10.97412	0.900082	2.403268
C	10.48276	0.970454	-2.21748
H	11.56046	1.128817	-2.26575
H	10.00723	1.642473	-2.93384
C	7.717595	-2.15	2.143505
H	7.053135	-2.83041	2.680891
H	7.091051	-1.40166	1.656487
C	8.517639	-2.94444	1.096404
C	7.517011	-3.71368	0.20521
H	6.83178	-3.00453	-0.27315
H	6.908849	-4.38506	0.822183
C	8.269213	-4.52384	-0.86591
H	7.544406	-5.06016	-1.48647
C	9.09151	-3.57354	-1.75196
H	9.619822	-4.14407	-2.52521
H	8.428877	-2.86991	-2.26416
C	10.10488	-2.7918	-0.8937
C	9.340891	-2.01585	0.190451
H	10.04782	-1.45472	0.801703
H	8.676336	-1.29703	-0.28864
C	9.464766	-3.9617	1.761511
H	8.889264	-4.64042	2.402242
H	10.17427	-3.43273	2.404672
C	10.22005	-4.76364	0.6896
H	10.89829	-5.4713	1.176831
C	9.211095	-5.52827	-0.18253
H	8.636035	-6.22685	0.434937
H	9.737222	-6.12251	-0.93761
C	11.03794	-3.80389	-0.19214
H	11.60002	-4.37251	-0.94188

H	11.76965	-3.26522	0.421283
C	10.97608	-1.85575	-1.75088
H	11.62688	-2.45496	-2.39155
H	11.61727	-1.24966	-1.10731
H	7.339397	0.717288	3.234803
H	11.88377	1.12397	0.124499
S	-9.54112	-1.84865	2.312667
S	-8.57692	0.777794	-3.56823
C	-10.039	-0.07978	2.563533
H	-9.54289	0.187577	3.498357
C	-9.60269	0.79395	1.426324
C	-10.5196	1.264148	0.480739
C	-10.0818	1.816708	-0.71652
H	-10.8047	2.101616	-1.47261
C	-8.72392	1.901391	-1.0174
C	-7.79671	1.551255	-0.01134
C	-8.24938	1.01784	1.205195
H	-7.51692	0.700911	1.936609
C	-8.27122	2.195349	-2.41355
H	-7.21759	2.467351	-2.45186
H	-8.8516	3.004337	-2.86053
C	-10.5028	-2.34431	0.81246
H	-11.0977	-3.20833	1.116931
H	-11.1964	-1.53184	0.585235
C	-9.68931	-2.705	-0.44318
C	-10.666	-3.26886	-1.4994
H	-11.4357	-2.52147	-1.72457
H	-11.1786	-4.15068	-1.09803
C	-9.90707	-3.64273	-2.78445
H	-10.6155	-4.0391	-3.51881
C	-9.22223	-2.39329	-3.35992
H	-8.68896	-2.64887	-4.2834
H	-9.97153	-1.63895	-3.61714
C	-8.23413	-1.8054	-2.33371
C	-8.99638	-1.47093	-1.04204
H	-8.30159	-1.06311	-0.30783
H	-9.74051	-0.703	-1.25362
C	-8.62184	-3.77395	-0.14228
H	-9.0954	-4.66829	0.279618
H	-7.92801	-3.39104	0.610208
C	-7.85997	-4.13705	-1.42706

H	-7.09716	-4.88697	-1.19729
C	-8.84398	-4.70467	-2.46245
H	-9.31904	-5.61043	-2.07005
H	-8.31002	-4.98966	-3.37549
C	-7.17735	-2.88095	-1.99645
H	-6.60843	-3.14019	-2.89665
H	-6.46427	-2.47938	-1.26699
C	-7.50525	-0.57413	-2.89875
H	-6.87414	-0.88292	-3.73521
H	-6.85181	-0.13761	-2.14273
H	-11.5825	1.143299	0.659634
H	-11.1164	-0.02958	2.723565
C	4.082869	1.045345	0.908883
C	5.434937	1.069674	0.911466
C	6.648313	1.10248	0.846814
C	-5.18868	1.547295	-0.32408
C	-6.39842	1.603746	-0.22502
C	-0.20942	4.843396	0.679981
C	-1.6394	4.802699	0.479215
H	-2.06303	5.351562	-0.35206
C	-2.44074	4.102534	1.310449
H	-3.51045	4.079729	1.150583
C	0.342881	4.087946	1.778675
H	1.415201	4.086456	1.923976
C	-0.45796	3.382006	2.605706
H	-0.0314	2.80939	3.418346
C	-1.88929	3.3624	2.421086
C	-2.70521	2.647552	3.279204
C	0.605808	5.5901	-0.15202
C	-2.16613	1.925382	4.379428
C	-4.11371	2.56358	3.103341
C	2.006919	5.689808	0.06593
C	0.084648	6.291121	-1.27329
N	-1.70999	1.33257	5.260628
N	-5.2582	2.472569	2.968132
N	-0.34991	6.843246	-2.19141
N	3.145174	5.765816	0.252715

4.0 APnA-TCNQ coordinates

APnA

Electronic Energy (EE): -4867.483758 Hartree

EE + Thermal Free Energy Correction: -4866.467931 Hartree

E (Thermal): 738.339 kcal/mol

Entropy (S): 340.401 cal/mol-kelvin

Imaginary Frequencies: 0

S	-1.27993	-2.55686	3.487505
C	-2.07109	-3.74014	2.319519
C	-1.30194	-4.07093	1.058133
C	0.088599	-4.16085	1.042267
C	0.773711	-4.34423	-0.15297
C	0.088385	-4.44582	-1.36331
C	-1.30823	-4.44827	-1.33637
C	-1.99308	-4.2625	-0.14249
C	-1.36208	-0.93576	2.600027
H	-3.06666	-3.37248	2.067692
H	-2.20317	-4.63096	2.940647
H	0.64116	-4.01494	1.961786
H	1.858453	-4.34987	-0.14972
H	-1.86153	-4.52851	-2.26585
H	-3.07568	-4.20662	-0.15013
H	-2.40621	-0.65824	2.467325
H	-0.91316	-0.24521	3.316039
C	0.842478	-4.42346	-2.66169
C	-0.61664	-0.92215	1.297021
S	0.742316	-2.80776	-3.5616
H	1.895206	-4.66884	-2.5133
H	0.429572	-5.13047	-3.38419
C	-1.30435	-1.0385	0.0676
C	0.768289	-0.91154	1.276021
C	1.562138	-1.65363	-2.39188
C	-0.58111	-1.22127	-1.12189
C	1.492141	-1.10975	0.088706
H	1.314865	-0.82689	2.207407
C	0.799139	-1.31015	-1.13032
H	2.54464	-2.05204	-2.13816

H	1.734214	-0.75615	-2.99275
H	-1.12536	-1.38118	-2.04237
S	10.47449	1.199065	3.109066
S	8.577426	-0.07289	-2.99561
C	10.77093	-0.62271	2.94254
H	11.84437	-0.81463	2.948939
H	10.34916	-1.02637	3.865012
C	10.11385	-1.19734	1.723936
C	10.86879	-1.54683	0.600314
C	10.2484	-1.7999	-0.61616
H	10.85107	-1.98386	-1.49845
C	8.865409	-1.70785	-0.75466
C	8.08886	-1.49699	0.408855
C	8.72733	-1.24895	1.633716
H	8.120632	-1.00153	2.496331
C	8.244656	-1.67147	-2.11652
H	7.170975	-1.84526	-2.0822
H	8.692824	-2.41286	-2.78019
C	11.3059	1.90408	1.614723
H	12.02533	2.631661	1.996977
H	11.8726	1.098236	1.14324
C	10.39786	2.585136	0.574842
C	11.30188	3.278307	-0.46905
H	11.95887	2.535815	-0.93695
H	11.94585	4.013782	0.026746
C	10.445	3.971006	-1.54331
H	11.10323	4.454874	-2.27191
C	9.572601	2.927358	-2.25891
H	8.967617	3.412009	-3.03452
H	10.20507	2.186768	-2.75721
C	8.651823	2.218816	-1.24706
C	9.517348	1.562566	-0.16017
H	8.872044	1.064785	0.563275
H	10.14936	0.800785	-0.61701
C	9.489852	3.649674	1.220414
H	10.10249	4.395688	1.74078
H	8.849393	3.176061	1.970096
C	8.628511	4.332853	0.1453
H	7.978835	5.074758	0.620268
C	9.542782	5.02368	-0.87936
H	10.15274	5.786667	-0.38339

H	8.941488	5.534678	-1.63916
C	7.759145	3.281327	-0.5676
H	7.124188	3.76794	-1.31682
H	7.093099	2.793973	0.153442
C	7.737082	1.193645	-1.93966
H	7.050648	1.718284	-2.6081
H	7.131794	0.664127	-1.20312
H	11.95121	-1.56065	0.665682
C	6.674477	-1.44063	0.34311
C	5.462553	-1.35953	0.290074
C	4.11401	-1.27096	0.219705
C	2.902742	-1.18569	0.148412
S	-10.657	0.358957	-2.83145
S	-8.02675	1.027987	3.093769
C	-10.8768	-1.32034	-2.07962
H	-11.9375	-1.4989	-1.90054
H	-10.55	-1.99138	-2.87629
C	-10.0687	-1.49413	-0.82925
C	-10.6814	-1.46847	0.427017
C	-9.9207	-1.33673	1.581343
H	-10.4142	-1.23045	2.540841
C	-8.53394	-1.22136	1.522966
C	-7.90033	-1.39162	0.268957
C	-8.67978	-1.52958	-0.88963
H	-8.18201	-1.56917	-1.85095
C	-7.76402	-0.77081	2.725285
H	-6.69796	-0.9647	2.626523
H	-8.12028	-1.25962	3.633608
C	-11.3247	1.499193	-1.53649
H	-12.1057	2.078948	-2.03335
H	-11.8069	0.884575	-0.7733
C	-10.3176	2.460319	-0.87927
C	-11.1066	3.46093	-0.00517
H	-11.6851	2.91424	0.748753
H	-11.8225	4.012434	-0.62548
C	-10.1438	4.443713	0.684495
H	-10.7215	5.142675	1.297635
C	-9.16804	3.665393	1.581555
H	-8.48639	4.360374	2.086302
H	-9.71883	3.129207	2.36003
C	-8.35824	2.660209	0.740468

C	-9.33147	1.70847	0.028074
H	-8.76547	0.998433	-0.57438
H	-9.88631	1.139365	0.7743
C	-9.51902	3.253814	-1.93136
H	-10.2072	3.807983	-2.581
H	-8.96244	2.558683	-2.56643
C	-8.55107	4.226729	-1.23848
H	-7.98039	4.770889	-1.99761
C	-9.35176	5.219486	-0.38042
H	-10.034	5.797259	-1.0136
H	-8.67594	5.933945	0.10206
C	-7.57815	3.44024	-0.34141
H	-6.86964	4.128291	0.133841
H	-6.99202	2.73997	-0.94731
C	-7.34451	1.890032	1.60473
H	-6.59753	2.587336	1.990779
H	-6.81689	1.148904	1.003559
H	-11.7635	-1.49079	0.495172
C	-6.48901	-1.33265	0.157914
C	-5.27878	-1.24598	0.079424
C	-3.92979	-1.15097	0.035562
C	-2.71663	-1.07123	0.03372

APnA-TCNQ-1

Electronic Energy (EE):	-5546.317016	Hartree
EE + Thermal Free Energy Correction:	-5545.187559	Hartree
E (Thermal):	828.939	kcal/mol
Entropy (S):	405.121	cal/mol-kelvin

Imaginary Frequencies: 0

S	-1.67053	3.20209	-3.52321
C	-2.38113	4.515483	-2.44655
C	-1.49343	5.052016	-1.34342
C	-0.11644	5.194967	-1.50497
C	0.686574	5.5785	-0.43735
C	0.136789	5.832884	0.819506

C	-1.25214	5.778661	0.957571
C	-2.05472	5.39172	-0.10824
C	-1.58224	1.730663	-2.40896
H	-3.32306	4.154732	-2.03094
H	-2.6269	5.302024	-3.16594
H	0.336953	4.938421	-2.45411
H	1.761964	5.628868	-0.57063
H	-1.70232	5.976385	1.924378
H	-3.12517	5.297233	0.03575
H	-2.5908	1.44943	-2.11247
H	-1.19084	0.95026	-3.06363
C	1.032002	6.031791	2.00885
C	-0.6901	1.941898	-1.2217
S	1.151458	4.553652	3.120202
H	2.041536	6.310817	1.703364
H	0.65421	6.808842	2.676272
C	-1.23139	2.23091	0.05276
C	0.685197	1.952765	-1.36376
C	1.888281	3.29374	2.010496
C	-0.38359	2.635971	1.098954
C	1.535806	2.327755	-0.31002
H	1.125948	1.716798	-2.32441
C	0.983054	2.738188	0.931047
H	2.791383	3.706562	1.559111
H	2.222915	2.500951	2.685148
H	-0.82434	2.934381	2.039932
S	8.652473	-3.15378	-2.70807
S	8.821925	1.083631	2.235087
C	9.472052	-1.66578	-3.44467
H	10.51853	-1.89298	-3.65139
H	8.955981	-1.54861	-4.39945
C	9.333631	-0.44873	-2.57999
C	10.42082	0.033989	-1.84386
C	10.23539	0.967806	-0.83279
H	11.0741	1.261508	-0.21185
C	8.966473	1.45088	-0.52196
C	7.890265	1.079494	-1.36065
C	8.086901	0.13166	-2.37864
H	7.225738	-0.22067	-2.93295
C	8.730721	2.196105	0.754636
H	7.774977	2.717144	0.755244

H	9.520451	2.924895	0.944373
C	9.604948	-3.43997	-1.14948
H	9.968104	-4.46825	-1.21324
H	10.47725	-2.7828	-1.16877
C	8.835542	-3.25402	0.170682
C	9.7373	-3.74716	1.324249
H	10.67586	-3.18043	1.325357
H	9.995939	-4.80105	1.169192
C	9.020911	-3.57637	2.675309
H	9.675978	-3.93088	3.47755
C	8.695102	-2.09145	2.902212
H	8.193751	-1.95969	3.868359
H	9.618255	-1.50503	2.933325
C	7.790616	-1.56625	1.771144
C	8.499548	-1.77623	0.424149
H	7.857351	-1.41728	-0.37963
H	9.416315	-1.18622	0.406152
C	7.526876	-4.06831	0.187299
H	7.746176	-5.12918	0.016059
H	6.879538	-3.73246	-0.62785
C	6.811299	-3.88906	1.53646
H	5.877562	-4.46088	1.528551
C	7.717727	-4.39183	2.671109
H	7.93747	-5.45625	2.532928
H	7.208814	-4.2893	3.635769
C	6.490315	-2.40059	1.755607
H	5.945623	-2.26302	2.695478
H	5.843922	-2.04275	0.948734
C	7.41269	-0.09099	1.987967
H	6.791739	0.001838	2.880146
H	6.820205	0.276168	1.150283
H	11.40992	-0.37301	-2.02355
C	6.579693	1.558218	-1.13097
C	5.425852	1.892168	-0.9425
C	4.119442	2.146668	-0.72457
C	2.927494	2.29004	-0.51974
S	-9.08771	-2.24901	3.293175
S	-8.22114	0.318028	-2.62988
C	-9.80295	-0.5487	3.461356
H	-10.8842	-0.62168	3.583245
H	-9.37896	-0.19437	4.402803

C	-9.43633	0.339674	2.310878
C	-10.3734	0.652982	1.320959
C	-9.97227	1.221425	0.119082
H	-10.6973	1.372752	-0.67256
C	-8.63084	1.496155	-0.13609
C	-7.70371	1.322418	0.917844
C	-8.11899	0.744186	2.127667
H	-7.37339	0.526248	2.882467
C	-8.17175	1.806942	-1.52648
H	-7.17044	2.233291	-1.54111
H	-8.84899	2.502642	-2.02484
C	-9.90884	-2.90627	1.772019
H	-10.398	-3.83293	2.080712
H	-10.6928	-2.20006	1.489632
C	-8.99749	-3.19025	0.564861
C	-9.84039	-3.91102	-0.51102
H	-10.6927	-3.28148	-0.79284
H	-10.2476	-4.84277	-0.1015
C	-8.98063	-4.2146	-1.75056
H	-9.59604	-4.72378	-2.4992
C	-8.44427	-2.9004	-2.34101
H	-7.83868	-3.10753	-3.23108
H	-9.27645	-2.26358	-2.65542
C	-7.59263	-2.15666	-1.29459
C	-8.45069	-1.89166	-0.04812
H	-7.85007	-1.37165	0.698284
H	-9.28204	-1.24046	-0.31879
C	-7.81063	-4.09601	0.947298
H	-8.18193	-5.03303	1.379837
H	-7.20576	-3.6004	1.712117
C	-6.95109	-4.39112	-0.29282
H	-6.10474	-5.02234	-0.00322
C	-7.80192	-5.11548	-1.34785
H	-8.17169	-6.06544	-0.94613
H	-7.19324	-5.35105	-2.22756
C	-6.41909	-3.07201	-0.87949
H	-5.78264	-3.27548	-1.7462
H	-5.80561	-2.55839	-0.13327
C	-7.00714	-0.85207	-1.8637
H	-6.29144	-1.08695	-2.65314
H	-6.46699	-0.30941	-1.08674

H	-11.4152	0.390948	1.469748
C	-6.33499	1.636153	0.74344
C	-5.149	1.861044	0.601514
C	-3.82281	2.044653	0.427964
C	-2.62241	2.153786	0.266778
C	1.634368	-0.93227	0.056838
C	0.731632	-0.65475	1.143675
H	1.142851	-0.33343	2.091125
C	-0.60584	-0.78693	0.995295
H	-1.27122	-0.5718	1.821225
C	1.063766	-1.35606	-1.19919
H	1.730686	-1.58086	-2.02214
C	-0.27539	-1.45869	-1.35716
H	-0.69036	-1.76799	-2.30813
C	-1.17799	-1.18168	-0.26577
C	-2.55117	-1.30069	-0.4209
C	3.004649	-0.78806	0.215401
C	-3.13034	-1.56884	-1.6895
C	-3.44947	-1.18638	0.673488
C	3.923907	-1.09224	-0.82277
C	3.544823	-0.30655	1.438507
N	-3.59293	-1.7629	-2.73149
N	-4.18863	-1.13816	1.561436
N	3.94362	0.101332	2.443993
N	4.674116	-1.34438	-1.66585

APnA-TCNQ-2

Electronic Energy (EE):	-5546.315143	Hartree
EE + Thermal Free Energy Correction:	-5545.185121	Hartree
E (Thermal):	829.253	kcal/mol
Entropy (S):	404.983	cal/mol-kelvin

Imaginary Frequencies: 0

S	-1.74252	3.098178	2.795911
C	-2.36743	1.68775	1.795876

C	-1.42002	1.13693	0.753022
C	-0.04709	1.038276	0.980757
C	0.818055	0.644261	-0.0353
C	0.333397	0.330947	-1.30411
C	-1.0511	0.342102	-1.50767
C	-1.91072	0.743893	-0.49531
C	-1.73373	4.503287	1.59273
H	-3.31162	1.974335	1.33273
H	-2.58941	0.938983	2.560435
H	0.35342	1.348763	1.937873
H	1.887501	0.640558	0.144791
H	-1.45147	0.105724	-2.48725
H	-2.9708	0.808011	-0.69452
H	-2.75586	4.703121	1.275813
H	-1.39536	5.342796	2.202653
C	1.281602	0.12733	-2.45086
C	-0.82307	4.290737	0.417629
S	1.223519	1.484527	-3.70347
H	2.308315	0.007917	-2.10149
H	1.02605	-0.75412	-3.043
C	-1.34052	3.933778	-0.84802
C	0.552899	4.31762	0.580948
C	1.82463	2.94042	-2.75544
C	-0.46407	3.555294	-1.8794
C	1.427065	3.911878	-0.43938
H	0.96924	4.576545	1.546843
C	0.903962	3.489003	-1.68729
H	2.797744	2.700685	-2.32916
H	1.996847	3.689335	-3.5335
H	-0.88252	3.218456	-2.81834
S	8.921687	-0.43455	-2.71187
S	6.523592	0.266865	3.314862
C	10.11375	0.73931	-1.91461
H	11.06804	0.235255	-1.75866
H	10.25629	1.503649	-2.68114
C	9.574564	1.312614	-0.63812
C	10.08382	0.919072	0.602925
C	9.398548	1.216103	1.774355
H	9.764753	0.835572	2.72122
C	8.178391	1.88866	1.747542
C	7.723793	2.39517	0.50852

C	8.441803	2.11702	-0.66419
H	8.029705	2.436197	-1.61363
C	7.303229	1.911599	2.962719
H	6.523052	2.667437	2.891511
H	7.884799	2.104116	3.865911
C	8.788188	-1.80805	-1.48056
H	9.065454	-2.71309	-2.02543
H	9.549771	-1.63905	-0.7161
C	7.416179	-2.02049	-0.81318
C	7.473545	-3.35296	-0.03323
H	8.278433	-3.31068	0.709512
H	7.708543	-4.17486	-0.71905
C	6.131119	-3.62098	0.666964
H	6.190678	-4.56878	1.211132
C	5.82772	-2.4829	1.652617
H	4.883643	-2.67036	2.177073
H	6.605859	-2.43053	2.419814
C	5.756063	-1.13546	0.909328
C	7.089373	-0.89462	0.182606
H	7.039243	0.049302	-0.35782
H	7.890864	-0.81143	0.917675
C	6.280544	-2.11125	-1.85217
H	6.487525	-2.91867	-2.56399
H	6.233336	-1.18018	-2.4238
C	4.936198	-2.36471	-1.14794
H	4.142706	-2.41272	-1.90139
C	5.012391	-3.69748	-0.38392
H	5.199544	-4.51764	-1.08468
H	4.061702	-3.91781	0.114297
C	4.649952	-1.2144	-0.16627
H	3.674847	-1.35192	0.310631
H	4.606129	-0.26307	-0.70971
C	5.39546	0.006278	1.875126
H	4.419297	-0.20021	2.319185
H	5.321764	0.950429	1.337968
H	10.99093	0.326167	0.646
C	6.45424	3.012239	0.3863
C	5.309081	3.387356	0.227106
C	4.001046	3.668525	0.029086
C	2.813297	3.827248	-0.17726
S	-9.07558	0.320669	2.595209

S	-6.21512	-0.94603	-3.1254
C	-10.1593	1.192091	1.37076
H	-11.1188	0.678158	1.306556
H	-10.3264	2.163062	1.841191
C	-9.5107	1.320436	0.025273
C	-9.93431	0.535154	-1.05096
C	-9.16072	0.434719	-2.19955
H	-9.4608	-0.24236	-2.99133
C	-7.93832	1.094302	-2.30805
C	-7.56984	1.99216	-1.27879
C	-8.37196	2.102274	-0.13198
H	-8.02886	2.725396	0.684945
C	-6.98067	0.720808	-3.397
H	-6.19138	1.459715	-3.52233
H	-7.49343	0.602147	-4.35299
C	-8.88124	-1.37627	1.882712
H	-9.22171	-2.05825	2.664905
H	-9.57516	-1.46526	1.043949
C	-7.46246	-1.77967	1.437884
C	-7.45964	-3.29617	1.140209
H	-8.19877	-3.52101	0.362672
H	-7.7551	-3.85115	2.037898
C	-6.0624	-3.74951	0.678678
H	-6.08239	-4.82454	0.474205
C	-5.67206	-2.99341	-0.60036
H	-4.68465	-3.31932	-0.94848
H	-6.3804	-3.21654	-1.40384
C	-5.65958	-1.47595	-0.33891
C	-7.04763	-1.04016	0.154914
H	-7.03921	0.030815	0.351854
H	-7.78426	-1.22538	-0.62781
C	-6.42076	-1.50068	2.539569
H	-6.69593	-2.03483	3.456553
H	-6.41526	-0.43331	2.776489
C	-5.02498	-1.94166	2.07138
H	-4.29497	-1.71641	2.85645
C	-5.03252	-3.45096	1.781012
H	-5.27858	-4.00596	2.692475
H	-4.0375	-3.78253	1.461783
C	-4.65398	-1.17252	0.793209
H	-3.64345	-1.43649	0.471338

H	-4.64957	-0.09676	0.996338
C	-5.21305	-0.70449	-1.59306
H	-4.20392	-1.02008	-1.86649
H	-5.1846	0.365267	-1.38953
H	-10.8454	-0.04657	-0.96311
C	-6.32131	2.66244	-1.30549
C	-5.20212	3.13624	-1.26331
C	-3.91503	3.544015	-1.17557
C	-2.73314	3.800338	-1.04905
C	0.372785	-2.17091	1.873713
C	1.458024	-2.73403	1.105338
H	2.428713	-2.834	1.572117
C	1.283096	-3.09226	-0.18414
H	2.112767	-3.47674	-0.76203
C	-0.93171	-2.11357	1.262581
H	-1.76684	-1.7448	1.841568
C	-1.10838	-2.48375	-0.02276
H	-2.08513	-2.4176	-0.47928
C	-0.0017	-2.94314	-0.82351
C	-0.16524	-3.21519	-2.16997
C	0.58974	-1.66469	3.142408
C	-1.42225	-3.01665	-2.80209
C	0.92249	-3.6419	-2.97852
C	-0.45979	-1.02954	3.859036
C	1.880731	-1.68116	3.734645
N	-2.45978	-2.82381	-3.27453
N	1.831554	-3.97079	-3.61283
N	2.948979	-1.69947	4.176793
N	-1.3386	-0.50117	4.39296

APnA-TCNQ-3

Electronic Energy (EE):	-5546.313489	Hartree
EE + Thermal Free Energy Correction:	-5545.184828	Hartree
E (Thermal):	829.166	kcal/mol
Entropy (S):	407.556	cal/mol-kelvin

Imaginary Frequencies: 0

S	2.791941	2.152686	-2.8913

C	3.407336	0.53393	-2.26935
C	2.363581	-0.4843	-1.86342
C	1.141998	-0.59934	-2.52439
C	0.157856	-1.45973	-2.05103
C	0.368078	-2.22952	-0.90662
C	1.625096	-2.18242	-0.29769
C	2.60732	-1.32182	-0.76978
C	2.113557	2.973914	-1.37981
H	4.096735	0.716101	-1.44473
H	4.003684	0.167991	-3.1102
H	0.935055	0.038889	-3.37411
H	-0.80605	-1.49223	-2.54763
H	1.81313	-2.77502	0.591186
H	3.553803	-1.25518	-0.24887
H	2.920917	3.151054	-0.67232
H	1.779525	3.938816	-1.76615
C	-0.77144	-2.98242	-0.28355
C	0.978071	2.219492	-0.75501
S	-1.46945	-2.1617	1.224347
H	-1.58366	-3.13654	-0.99574
H	-0.4589	-3.95923	0.090594
C	1.182516	1.415123	0.390199
C	-0.27221	2.202463	-1.35544
C	-2.08427	-0.57842	0.53915
C	0.166806	0.548612	0.825493
C	-1.28999	1.34846	-0.91356
H	-0.4515	2.816003	-2.23001
C	-1.04581	0.459131	0.166941
H	-2.71779	-0.79542	-0.32006
H	-2.74769	-0.20289	1.32021
H	0.37005	-0.12299	1.648195
S	-9.98662	0.877659	1.08314
S	-6.15865	-3.33165	-2.1087
C	-10.6316	0.965182	-0.65115
H	-11.6259	0.519016	-0.69269
H	-10.7338	2.038586	-0.82162
C	-9.69705	0.326333	-1.63439
C	-9.99888	-0.90814	-2.21695
C	-9.02062	-1.63638	-2.88307
H	-9.24917	-2.62907	-3.25434
C	-7.71298	-1.16638	-2.97802

C	-7.43996	0.13881	-2.50702
C	-8.43734	0.874202	-1.85113
H	-8.16975	1.819913	-1.39773
C	-6.60653	-2.08129	-3.40559
H	-5.7125	-1.53302	-3.69669
H	-6.91313	-2.70751	-4.24516
C	-9.93145	-0.93813	1.426345
H	-10.5476	-1.08613	2.31602
H	-10.4269	-1.44681	0.5963
C	-8.54172	-1.55699	1.665907
C	-8.74992	-3.00185	2.172531
H	-9.32349	-3.57195	1.432048
H	-9.33852	-2.9886	3.097077
C	-7.39435	-3.68395	2.424053
H	-7.56519	-4.7043	2.782086
C	-6.59342	-3.73121	1.11364
H	-5.62917	-4.22583	1.280144
H	-7.13368	-4.31556	0.362416
C	-6.35836	-2.30454	0.583265
C	-7.71565	-1.61483	0.370354
H	-7.55067	-0.59888	0.013474
H	-8.27576	-2.15003	-0.39753
C	-7.73913	-0.77643	2.725356
H	-8.30643	-0.72807	3.662599
H	-7.59067	0.251714	2.382404
C	-6.38089	-1.45552	2.967551
H	-5.81378	-0.88515	3.706958
C	-6.60642	-2.88803	3.476919
H	-7.15613	-2.86593	4.424259
H	-5.64456	-3.37383	3.672837
C	-5.58391	-1.50342	1.652899
H	-4.60315	-1.95902	1.825765
H	-5.41258	-0.48668	1.281816
C	-5.51256	-2.31925	-0.70049
H	-4.52995	-2.73899	-0.47046
H	-5.36035	-1.30503	-1.06556
H	-10.9905	-1.32961	-2.09323
C	-6.11502	0.639079	-2.50542
C	-4.95957	0.98091	-2.35238
C	-3.67946	1.213452	-1.98591
C	-2.54847	1.328478	-1.55467

S	10.35907	1.363483	-0.69563
S	6.650998	-3.13834	2.215941
C	10.66618	1.665035	1.106602
H	11.68755	1.373522	1.353267
H	10.59423	2.751125	1.19066
C	9.665092	0.964535	1.975517
C	10.01939	-0.17033	2.711061
C	9.042479	-0.98256	3.272699
H	9.329674	-1.90289	3.76858
C	7.68787	-0.70249	3.10759
C	7.3296	0.513021	2.479583
C	8.326449	1.33652	1.934767
H	8.027681	2.217893	1.380612
C	6.658489	-1.73983	3.434526
H	5.657371	-1.31798	3.50069
H	6.883365	-2.23976	4.37812
C	10.62082	-0.45885	-0.87489
H	11.39608	-0.56741	-1.63651
H	11.03296	-0.82646	0.067455
C	9.395248	-1.30072	-1.27572
C	9.884146	-2.73053	-1.59876
H	10.39143	-3.15266	-0.7234
H	10.61744	-2.69674	-2.41259
C	8.697807	-3.62499	-1.99848
H	9.065177	-4.6313	-2.22302
C	7.693163	-3.69634	-0.83786
H	6.850065	-4.34248	-1.10988
H	8.167323	-4.13645	0.04464
C	7.177665	-2.28636	-0.49341
C	8.373043	-1.39078	-0.13117
H	8.015013	-0.38856	0.101838
H	8.859019	-1.78337	0.762816
C	8.693037	-0.73102	-2.52399
H	9.404198	-0.67015	-3.35633
H	8.349326	0.286447	-2.31663
C	7.501619	-1.62155	-2.91406
H	7.004454	-1.19569	-3.79154
C	8.004888	-3.03722	-3.23874
H	8.703282	-3.00239	-4.08187
H	7.168005	-3.67711	-3.53883
C	6.501138	-1.68708	-1.74682

H	5.631235	-2.29089	-2.02923
H	6.134604	-0.68112	-1.51461
C	6.132525	-2.33379	0.633477
H	5.266373	-2.90418	0.290409
H	5.788503	-1.32829	0.874643
H	11.06383	-0.45068	2.792562
C	5.972508	0.844826	2.246615
C	4.821744	1.083525	1.936293
C	3.555688	1.272614	1.50145
C	2.441198	1.378356	1.028964
C	-0.75446	4.847536	0.459626
C	-2.00432	4.899465	-0.26336
H	-2.08504	5.564275	-1.11391
C	-3.05108	4.127481	0.098838
H	-3.97716	4.161999	-0.45948
C	-0.65954	3.951941	1.586184
H	0.274563	3.894513	2.128818
C	-1.7089	3.183938	1.951917
H	-1.62173	2.509331	2.793245
C	-2.9574	3.235513	1.231089
C	-4.03565	2.457161	1.614817
C	0.324431	5.624231	0.075193
C	-3.96011	1.601877	2.747766
C	-5.27068	2.478848	0.912515
C	1.568351	5.555516	0.758958
C	0.258354	6.49058	-1.04923
N	-3.87364	0.909498	3.669637
N	-6.27366	2.50558	0.338794
N	0.195271	7.178501	-1.97641
N	2.580129	5.471603	1.311854

APnA-TCNQ-4

Electronic Energy (EE):	-5546.313447	Hartree
EE + Thermal Free Energy Correction:	-5545.185575	Hartree
E (Thermal):	829.161	kcal/mol
Entropy (S):	409.200	cal/mol-kelvin

Imaginary Frequencies: 0

S	1.115655	6.260423	1.290018
C	2.090191	6.095774	-0.26269
C	1.441803	5.352203	-1.41198
C	0.071995	5.419797	-1.6631
C	-0.50524	4.639289	-2.6578
C	0.267037	3.764683	-3.42496
C	1.650609	3.764146	-3.23321
C	2.228668	4.548016	-2.24248
C	1.108395	4.548246	1.988646
H	3.055834	5.648162	-0.02283
H	2.278198	7.138811	-0.53378
H	-0.55419	6.038835	-1.03305
H	-1.57978	4.671422	-2.80285
H	2.272645	3.098617	-3.82225
H	3.297763	4.490426	-2.07212
H	2.135319	4.246555	2.192994
H	0.586706	4.670092	2.938298
C	-0.39431	2.766568	-4.33035
C	0.405911	3.566298	1.099444
S	-0.58934	1.077062	-3.58208
H	-1.38342	3.105476	-4.64172
H	0.197926	2.575266	-5.22672
C	1.139474	2.73196	0.229501
C	-0.97942	3.531164	1.028659
C	-1.61183	1.455487	-2.1128
C	0.474919	2.017261	-0.77964
C	-1.64528	2.786381	0.042865
H	-1.56473	4.131137	1.714674
C	-0.89627	2.080444	-0.93197
H	-2.43252	2.104304	-2.42362
H	-2.07599	0.505501	-1.83985
H	1.059694	1.45616	-1.49408
S	-9.76083	-1.04923	2.886412
S	-7.02469	-0.88542	-3.0255
C	-10.7351	0.26302	2.012374
H	-11.7492	-0.09704	1.836207
H	-10.7842	1.066273	2.750123
C	-10.0759	0.704459	0.740287
C	-10.5837	0.318441	-0.50372
C	-9.82315	0.468999	-1.65618

H	-10.197	0.088228	-2.59991
C	-8.53276	0.991318	-1.60658
C	-8.06896	1.505865	-0.373
C	-8.85427	1.366599	0.780866
H	-8.44395	1.683769	1.73181
C	-7.6173	0.854553	-2.78378
H	-6.75849	1.519313	-2.71392
H	-8.14005	1.066304	-3.71807
C	-9.79073	-2.46714	1.698117
H	-10.2218	-3.30292	2.253717
H	-10.4882	-2.20996	0.897934
C	-8.44007	-2.89843	1.097651
C	-8.66113	-4.22053	0.328707
H	-9.4164	-4.07013	-0.45158
H	-9.04837	-4.98635	1.010771
C	-7.34274	-4.69902	-0.30417
H	-7.52122	-5.63548	-0.84228
C	-6.82696	-3.63613	-1.28665
H	-5.8928	-3.97282	-1.75056
H	-7.55292	-3.48416	-2.09113
C	-6.5887	-2.30286	-0.55228
C	-7.90164	-1.85333	0.107666
H	-7.73511	-0.91678	0.639156
H	-8.64767	-1.66625	-0.66559
C	-7.37932	-3.13969	2.189293
H	-7.74091	-3.89255	2.900129
H	-7.21811	-2.21472	2.750669
C	-6.06026	-3.60694	1.551693
H	-5.31376	-3.75965	2.338078
C	-6.29606	-4.9263	0.798598
H	-6.64039	-5.69947	1.494376
H	-5.35904	-5.28211	0.356846
C	-5.54981	-2.53879	0.567816
H	-4.59773	-2.85231	0.128313
H	-5.36847	-1.59666	1.09912
C	-6.02707	-1.2341	-1.50535
H	-5.04819	-1.55299	-1.86561
H	-5.88898	-0.29064	-0.97816
H	-11.5562	-0.15838	-0.55982
C	-6.75483	2.020552	-0.25406
C	-5.58875	2.346361	-0.14771

C	-4.26242	2.595467	-0.06073
C	-3.05676	2.74106	0.003341
S	9.161409	-1.19718	-2.73832
S	6.375089	-1.55587	3.156364
C	10.14284	0.006023	-1.72729
H	11.14964	-0.3852	-1.5777
H	10.21268	0.878478	-2.37988
C	9.475557	0.329287	-0.42398
C	9.967882	-0.17609	0.782888
C	9.19908	-0.12249	1.938582
H	9.560473	-0.59258	2.846197
C	7.916208	0.42129	1.92936
C	7.470426	1.053641	0.745687
C	8.261654	1.006458	-0.41161
H	7.863981	1.41713	-1.33166
C	6.989202	0.194302	3.082479
H	6.140293	0.874119	3.084112
H	7.507264	0.303159	4.036756
C	9.173596	-2.72875	-1.70137
H	9.593002	-3.50912	-2.34011
H	9.875906	-2.56351	-0.88131
C	7.819089	-3.20327	-1.14486
C	8.032213	-4.59085	-0.49883
H	8.788214	-4.51634	0.291714
H	8.415224	-5.29359	-1.24755
C	6.711034	-5.11808	0.087342
H	6.884274	-6.0997	0.539643
C	6.203385	-4.14489	1.163344
H	5.268084	-4.51923	1.596915
H	6.930176	-4.06932	1.977645
C	5.971413	-2.75083	0.550744
C	7.286136	-2.24834	-0.06488
H	7.124403	-1.26672	-0.50955
H	8.031104	-2.13449	0.723246
C	6.757591	-3.3372	-2.25446
H	7.115246	-4.023	-3.03161
H	6.60095	-2.36408	-2.72902
C	5.435506	-3.85614	-1.66458
H	4.681951	-3.9264	-2.4531
C	5.664664	-5.23841	-1.03268
H	6.006214	-5.94689	-1.79499

H	4.724182	-5.62882	-0.62902
C	4.933288	-2.88286	-0.58507
H	3.982374	-3.24924	-0.18194
H	4.743624	-1.89899	-1.03064
C	5.41367	-1.76563	1.591707
H	4.429697	-2.11855	1.909888
H	5.287765	-0.77892	1.144269
H	10.93436	-0.66774	0.802446
C	6.172703	1.616583	0.662565
C	5.031744	2.020113	0.552943
C	3.731286	2.371444	0.434863
C	2.541652	2.601544	0.340029
C	0.37682	-1.10368	0.326868
C	1.762724	-0.96067	0.701785
H	2.519729	-1.45494	0.107604
C	2.119488	-0.21138	1.765788
H	3.161242	-0.10171	2.030681
C	-0.62072	-0.45399	1.146268
H	-1.66509	-0.56362	0.884052
C	-0.26273	0.302384	2.206112
H	-1.01633	0.808555	2.794356
C	1.127858	0.486869	2.547052
C	1.51064	1.343889	3.562008
C	0.022941	-1.83858	-0.7912
C	0.555525	2.092745	4.29996
C	2.880263	1.573205	3.863765
C	-1.33414	-2.00474	-1.17762
C	1.001561	-2.46325	-1.61246
N	-0.23329	2.717247	4.869377
N	3.998448	1.759924	4.088188
N	1.809447	-2.96832	-2.26681
N	-2.44224	-2.12505	-1.48436

APnA-TCNQ-5

Electronic Energy (EE):	-5546.311137	Hartree
EE + Thermal Free Energy Correction:	-5545.185961	Hartree
E (Thermal):	828.865	kcal/mol

Entropy (S): 413.881 cal/mol-kelvin

Imaginary Frequencies: 0

S	0.928211	-1.51538	-3.69581
C	2.198452	-2.16628	-2.53296
C	1.843421	-2.1778	-1.06064
C	0.544347	-2.41144	-0.6123
C	0.229762	-2.30032	0.736944
C	1.20214	-1.94959	1.674785
C	2.521212	-1.80465	1.237748
C	2.836351	-1.91925	-0.11021
C	0.904647	0.302125	-3.35252
H	3.129738	-1.62062	-2.69116
H	2.355498	-3.18381	-2.90266
H	-0.23762	-2.61704	-1.3323
H	-0.79772	-2.43602	1.057263
H	3.296955	-1.53807	1.947474
H	3.856123	-1.749	-0.43394
H	1.883772	0.722393	-3.57965
H	0.191732	0.680886	-4.08667
C	0.80481	-1.61381	3.082754
C	0.474911	0.629401	-1.95353
S	0.679612	0.206547	3.417003
H	-0.15012	-2.07054	3.346747
H	1.551438	-1.94572	3.806512
C	1.438627	0.923463	-0.96321
C	-0.85278	0.549677	-1.56723
C	-0.63645	0.712732	2.247763
C	1.059101	0.992945	0.388492
C	-1.23621	0.654593	-0.21806
H	-1.61733	0.351292	-2.30859
C	-0.24679	0.802311	0.787116
H	-1.48472	0.036323	2.36168
H	-0.97514	1.680104	2.626551
H	1.826285	1.12689	1.137456
S	-10.1092	-2.58224	2.653175
S	-8.59206	1.580934	-2.12349
C	-10.1726	-0.93086	3.491764
H	-11.2064	-0.69591	3.746729

H	-9.62584	-1.10448	4.420593
C	-9.54773	0.151217	2.663859
C	-10.3381	1.095848	2.00204
C	-9.79445	1.907861	1.01545
H	-10.438	2.575783	0.454263
C	-8.45489	1.800958	0.647215
C	-7.62295	0.948761	1.411008
C	-8.18045	0.141245	2.414006
H	-7.54229	-0.56676	2.928609
C	-7.96272	2.448412	-0.60968
H	-6.87857	2.518385	-0.6543
H	-8.36778	3.455473	-0.72167
C	-11.1489	-2.33392	1.143395
H	-11.9246	-3.10121	1.194638
H	-11.6405	-1.36333	1.239043
C	-10.4306	-2.42732	-0.21503
C	-11.5043	-2.40864	-1.32611
H	-12.0964	-1.4892	-1.24966
H	-12.1937	-3.24995	-1.19078
C	-10.8415	-2.49105	-2.71241
H	-11.6179	-2.47636	-3.48393
C	-9.90539	-1.28776	-2.90825
H	-9.43858	-1.33253	-3.89946
H	-10.476	-0.35556	-2.86108
C	-8.81535	-1.27767	-1.81939
C	-9.49015	-1.23299	-0.43951
H	-8.72496	-1.23367	0.336371
H	-10.0532	-0.30409	-0.34661
C	-9.6177	-3.73023	-0.34209
H	-10.2756	-4.59548	-0.1967
H	-8.85875	-3.76417	0.444921
C	-8.94878	-3.80395	-1.72466
H	-8.36329	-4.72626	-1.79408
C	-10.0303	-3.79275	-2.81689
H	-10.6895	-4.66012	-2.70098
H	-9.56881	-3.86804	-3.80749
C	-8.01433	-2.59543	-1.91403
H	-7.51537	-2.65853	-2.8879
H	-7.23056	-2.60371	-1.14789
C	-7.83829	-0.10452	-2.01203
H	-7.29305	-0.24208	-2.94867

H	-7.10148	-0.08669	-1.20849
H	-11.3987	1.152991	2.221219
C	-6.24453	0.825279	1.112788
C	-5.06996	0.701516	0.826441
C	-3.76697	0.621097	0.480053
C	-2.59874	0.595724	0.141123
S	10.24212	-2.28575	-2.34683
S	7.551061	1.14905	2.488864
C	10.85872	-0.56691	-2.66345
H	11.92998	-0.52495	-2.4644
H	10.70887	-0.44485	-3.73793
C	10.10694	0.457917	-1.86838
C	10.69218	1.08408	-0.76357
C	9.916057	1.798888	0.140066
H	10.37341	2.210505	1.032614
C	8.534693	1.896999	-0.01228
C	7.960302	1.377725	-1.19723
C	8.757782	0.679937	-2.11656
H	8.282391	0.214945	-2.97155
C	7.682145	2.389791	1.115853
H	6.685508	2.685946	0.797969
H	8.141492	3.246611	1.61141
C	10.62139	-2.55174	-0.55575
H	11.24912	-3.44483	-0.52094
H	11.23015	-1.70928	-0.22027
C	9.420038	-2.7391	0.389272
C	9.963615	-3.1755	1.768466
H	10.65718	-2.41594	2.147616
H	10.52984	-4.10832	1.665859
C	8.80628	-3.37065	2.763184
H	9.212306	-3.67955	3.731611
C	8.04233	-2.0479	2.92899
H	7.223101	-2.17471	3.646918
H	8.706387	-1.27609	3.329299
C	7.477535	-1.58448	1.572708
C	8.637095	-1.42943	0.575097
H	8.241815	-1.11442	-0.39011
H	9.311437	-0.6475	0.926001
C	8.453734	-3.82582	-0.12101
H	8.991443	-4.7729	-0.24867
H	8.06752	-3.53777	-1.10309

C	7.292441	-4.00975	0.870266
H	6.606301	-4.77148	0.486874
C	7.849135	-4.45086	2.233548
H	8.374939	-5.40647	2.132147
H	7.03023	-4.60697	2.944214
C	6.533506	-2.6807	1.031399
H	5.684299	-2.8158	1.710842
H	6.126128	-2.36527	0.064036
C	6.67016	-0.28331	1.719749
H	5.80608	-0.46956	2.3626
H	6.288888	0.041252	0.752597
H	11.75394	0.960779	-0.57988
C	6.559301	1.407776	-1.38657
C	5.34769	1.337509	-1.45439
C	4.005773	1.217096	-1.39763
C	2.804412	1.08047	-1.26594
C	1.338641	4.144608	-0.49002
C	0.213922	4.018668	-1.38216
H	0.400058	3.972124	-2.44695
C	-1.04955	3.939978	-0.90849
H	-1.88204	3.827468	-1.59074
C	1.06964	4.201162	0.927096
H	1.902802	4.306031	1.610622
C	-0.19432	4.122344	1.400512
H	-0.37966	4.171719	2.465981
C	-1.31786	3.974872	0.506357
C	-2.61363	3.866722	0.987969
C	2.632494	4.195766	-0.98309
C	-2.88436	3.802639	2.380653
C	-3.73567	3.822307	0.11739
C	3.763285	4.255832	-0.12611
C	2.888114	4.160732	-2.38112
N	-3.07785	3.731259	3.51856
N	-4.64799	3.813223	-0.59227
N	3.063647	4.13145	-3.5233
N	4.68241	4.289985	0.574189

APnA-TCNQ-6

Electronic Energy (EE):	-5546.310604	Hartree

EE + Thermal Free Energy Correction:	-5545.182484	Hartree
E (Thermal):	829.170	kcal/mol
Entropy (S):	408.709	cal/mol-kelvin

Imaginary Frequencies: 0

S	-1.74156	-2.30426	-3.4531
C	-2.21378	-0.86223	-2.41703
C	-1.12491	-0.2707	-1.54435
C	0.218148	-0.28526	-1.91647
C	1.205275	0.139442	-1.03436
C	0.87799	0.590317	0.246733
C	-0.47272	0.687887	0.588862
C	-1.45863	0.268249	-0.29477
C	-1.54248	-3.67399	-2.22415
H	-3.07401	-1.13488	-1.80409
H	-2.57084	-0.13604	-3.15215
H	0.497725	-0.7013	-2.87582
H	2.247884	0.066188	-1.32431
H	-0.7503	1.033665	1.578363
H	-2.49819	0.293107	0.012875
H	-2.50689	-3.86808	-1.75804
H	-1.28725	-4.52859	-2.85288
C	1.960467	0.834115	1.257647
C	-0.47561	-3.41759	-1.20026
S	2.12056	-0.50715	2.519603
H	2.930232	0.966103	0.777235
H	1.760808	1.717643	1.86738
C	-0.81243	-2.97343	0.098629
C	0.866471	-3.51641	-1.53377
C	2.603814	-1.94989	1.4927
C	0.199193	-2.54248	0.972476
C	1.878269	-3.09808	-0.65653
H	1.147378	-3.8696	-2.51868
C	1.531406	-2.54505	0.603347
H	3.474134	-1.67321	0.89835
H	2.94938	-2.68466	2.225299
H	-0.0826	-2.13336	1.933025
S	9.692964	-1.66126	2.88034

S	7.835998	0.707553	-2.89654
C	10.74125	-2.60098	1.675262
H	11.77741	-2.27299	1.764518
H	10.67909	-3.62763	2.041797
C	10.23921	-2.47304	0.267585
C	10.92954	-1.71429	-0.68237
C	10.32129	-1.35663	-1.87891
H	10.83986	-0.70049	-2.56881
C	9.004299	-1.71844	-2.15761
C	8.348237	-2.58687	-1.25712
C	8.985269	-2.96878	-0.06674
H	8.430517	-3.55887	0.652288
C	8.261531	-1.05603	-3.27685
H	7.352062	-1.59405	-3.53932
H	8.8819	-0.97404	-4.17095
C	9.926788	0.099395	2.362888
H	10.29913	0.614588	3.251036
H	10.72314	0.116769	1.615345
C	8.690758	0.845332	1.826729
C	9.068869	2.337405	1.68753
H	9.926744	2.437164	1.012105
H	9.376163	2.734025	2.662036
C	7.87727	3.144155	1.144842
H	8.165583	4.196409	1.055426
C	7.480788	2.604752	-0.2383
H	6.64201	3.180829	-0.64428
H	8.315157	2.712889	-0.93828
C	7.08784	1.118431	-0.13811
C	8.268607	0.324247	0.443613
H	7.986854	-0.7241	0.533711
H	9.113792	0.380458	-0.24401
C	7.486896	0.731133	2.782951
H	7.761559	1.105622	3.776219
H	7.211834	-0.32143	2.898283
C	6.29468	1.532909	2.232435
H	5.442924	1.428419	2.911671
C	6.687859	3.013854	2.110049
H	6.95492	3.411402	3.09518
H	5.840406	3.60444	1.75005
C	5.903395	0.992172	0.846334
H	5.046239	1.551898	0.456946

H	5.599586	-0.0586	0.923966
C	6.632009	0.568966	-1.49993
H	5.751491	1.120195	-1.83132
H	6.354087	-0.47991	-1.41096
H	11.92642	-1.35166	-0.45654
C	6.980294	-2.92584	-1.41205
C	5.778056	-3.10379	-1.39542
C	4.433914	-3.1732	-1.25732
C	3.237939	-3.16995	-1.03791
S	-10.2314	-2.31064	-1.37822
S	-6.85802	0.845322	3.24022
C	-10.428	-3.39048	0.114047
H	-11.4619	-3.34706	0.457997
H	-10.2443	-4.39208	-0.27971
C	-9.46669	-3.02982	1.206675
C	-9.90264	-2.37132	2.360232
C	-8.98965	-1.7853	3.227484
H	-9.34655	-1.20629	4.071722
C	-7.62101	-1.82475	2.970602
C	-7.16864	-2.60332	1.880315
C	-8.10038	-3.20309	1.019313
H	-7.7381	-3.72114	0.139756
C	-6.68531	-0.93517	3.728427
H	-5.64689	-1.24035	3.612701
H	-6.92299	-0.9221	4.793449
C	-10.6809	-0.62984	-0.74968
H	-11.4887	-0.28463	-1.39875
H	-11.0963	-0.75451	0.252846
C	-9.5618	0.427001	-0.72735
C	-10.2037	1.792324	-0.39281
H	-10.7173	1.730609	0.573859
H	-10.9603	2.040433	-1.14619
C	-9.12847	2.89156	-0.34469
H	-9.60246	3.848962	-0.10612
C	-8.09319	2.553027	0.739579
H	-7.33277	3.338182	0.794878
H	-8.57918	2.501242	1.718818
C	-7.42629	1.198804	0.429728
C	-8.51016	0.113755	0.349892
H	-8.04577	-0.84457	0.120491
H	-8.99891	0.020804	1.320263

C	-8.85594	0.541475	-2.09285
H	-9.59019	0.776337	-2.87276
H	-8.40436	-0.42045	-2.35232
C	-7.77555	1.634162	-2.04066
H	-7.26649	1.692039	-3.00726
C	-8.42811	2.986238	-1.71026
H	-9.15027	3.25425	-2.48927
H	-7.6697	3.776157	-1.68656
C	-6.74587	1.288199	-0.95283
H	-5.9688	2.055596	-0.92808
H	-6.25546	0.336535	-1.18813
C	-6.33982	0.859516	1.464755
H	-5.54986	1.610939	1.406222
H	-5.89237	-0.11001	1.243136
H	-10.9657	-2.26595	2.54684
C	-5.79069	-2.70096	1.566045
C	-4.62107	-2.76218	1.240274
C	-3.32249	-2.82772	0.86647
C	-2.1612	-2.89576	0.513023
C	0.987991	3.733831	-0.54902
C	0.052718	4.24231	0.426854
H	0.438668	4.693565	1.332193
C	-1.2788	4.151778	0.222955
H	-1.97293	4.529097	0.963112
C	0.460286	3.149992	-1.75743
H	1.151691	2.777296	-2.50008
C	-0.87104	3.047085	-1.95509
H	-1.25357	2.591395	-2.85778
C	-1.80596	3.533638	-0.97184
C	-3.17032	3.408089	-1.16308
C	2.351495	3.79291	-0.32451
C	-3.69393	2.739768	-2.3028
C	-4.10551	3.907918	-0.21814
C	3.275487	3.268206	-1.26594
C	2.896495	4.329971	0.872334
N	-4.09104	2.172075	-3.22826
N	-4.85456	4.31804	0.561275
N	3.333987	4.751331	1.856053
N	4.012734	2.823438	-2.03717

APnA-TCNQ-7

Electronic Energy (EE):	-5546.306936	Hartree
EE + Thermal Free Energy Correction:	-5545.181493	Hartree
E (Thermal):	829.012	kcal/mol
Entropy (S):	413.812	cal/mol-kelvin

Imaginary Frequencies: 0

S	0.021999	-3.48272	2.557862
C	0.555571	-1.75505	2.216082
C	-0.29891	-0.95381	1.256242
C	-1.68321	-1.10432	1.194779
C	-2.42623	-0.45096	0.218041
C	-1.80609	0.371258	-0.72305
C	-0.43259	0.598472	-0.6023
C	0.308775	-0.05432	0.374063
C	0.37159	-4.37843	0.978058
H	1.591757	-1.76995	1.875978
H	0.549263	-1.30365	3.212564
H	-2.173	-1.79443	1.870122
H	-3.49264	-0.63315	0.15033
H	0.070935	1.239524	-1.31769
H	1.382068	0.090696	0.415844
H	1.444434	-4.36346	0.797693
H	0.085348	-5.40435	1.217058
C	-2.58096	0.887034	-1.90116
C	-0.41401	-3.86082	-0.1926
S	-2.22899	-0.00829	-3.48398
H	-3.65433	0.849012	-1.71771
H	-2.31592	1.918717	-2.14193
C	0.192895	-3.03636	-1.16485
C	-1.78303	-4.06255	-0.27371
C	-2.81382	-1.71151	-3.11791
C	-0.58993	-2.41003	-2.14867
C	-2.57446	-3.39207	-1.22
H	-2.2761	-4.66756	0.477288
C	-1.96682	-2.53009	-2.16701
H	-3.84034	-1.65813	-2.7568
H	-2.85089	-2.18627	-4.10233

H	-0.1106	-1.72931	-2.83875
S	-10.248	1.338623	-2.4245
S	-7.53547	-0.86675	3.072439
C	-11.3911	-0.02617	-1.91028
H	-12.3408	0.400312	-1.5858
H	-11.5639	-0.56687	-2.8429
C	-10.7872	-0.90155	-0.85329
C	-11.2305	-0.84164	0.471046
C	-10.487	-1.41999	1.492039
H	-10.8014	-1.29419	2.521999
C	-9.27435	-2.05498	1.232467
C	-8.88818	-2.22991	-0.11702
C	-9.66168	-1.66469	-1.14157
H	-9.30185	-1.73052	-2.16107
C	-8.33792	-2.37815	2.356356
H	-7.56543	-3.0839	2.057224
H	-8.8733	-2.79664	3.21029
C	-10.0602	2.344787	-0.88341
H	-10.3721	3.355005	-1.15735
H	-10.7788	1.96588	-0.15341
C	-8.65509	2.401522	-0.25488
C	-8.67272	3.481791	0.850043
H	-9.43642	3.232576	1.595992
H	-8.94741	4.450464	0.416997
C	-7.29401	3.58108	1.525597
H	-7.32834	4.35102	2.302839
C	-6.9354	2.22916	2.161891
H	-5.9593	2.293555	2.657087
H	-7.67084	1.966097	2.928309
C	-6.89971	1.127532	1.086001
C	-8.26913	1.060611	0.390535
H	-8.24409	0.29171	-0.38069
H	-9.029	0.772456	1.118096
C	-7.57847	2.780172	-1.29088
H	-7.82981	3.740969	-1.75573
H	-7.55918	2.030464	-2.08706
C	-6.20035	2.867984	-0.61431
H	-5.44464	3.121427	-1.36501
C	-6.23255	3.950828	0.476172
H	-6.46365	4.924609	0.030849
H	-5.24973	4.037863	0.952021

C	-5.84768	1.512822	0.022609
H	-4.85388	1.561659	0.48101
H	-5.81321	0.733844	-0.74844
C	-6.49013	-0.2271	1.6873
H	-5.48159	-0.14428	2.099453
H	-6.46368	-0.99377	0.914375
H	-12.1316	-0.28718	0.709501
C	-7.6349	-2.80335	-0.44624
C	-6.49706	-3.14342	-0.70528
C	-5.18384	-3.36971	-0.93835
C	-3.98248	-3.4537	-1.10633
S	5.461486	2.572445	3.562012
S	5.804554	2.008225	-2.91464
C	6.95883	1.512382	3.312523
H	7.85331	2.07245	3.586686
H	6.81932	0.709252	4.039331
C	7.04006	0.987221	1.910321
C	7.998477	1.452947	1.00529
C	7.881832	1.175692	-0.35206
H	8.589713	1.609569	-1.0489
C	6.809638	0.435183	-0.85111
C	5.91741	-0.15004	0.073433
C	6.050948	0.130704	1.440964
H	5.302092	-0.24498	2.126412
C	6.523879	0.407285	-2.31897
H	5.859671	-0.41127	-2.59235
H	7.439335	0.319055	-2.90625
C	5.753197	4.001096	2.422301
H	5.71207	4.890064	3.055373
H	6.773852	3.916375	2.042442
C	4.768294	4.166805	1.25004
C	5.056416	5.525921	0.573539
H	6.097237	5.553026	0.23039
H	4.93466	6.336007	1.301553
C	4.106428	5.743071	-0.61715
H	4.326239	6.709759	-1.08093
C	4.312905	4.624103	-1.65022
H	3.649816	4.778924	-2.5096
H	5.340258	4.644524	-2.02633
C	4.025511	3.251683	-1.0123
C	4.949972	3.059522	0.200345

H	4.733478	2.099024	0.665159
H	5.98773	3.039248	-0.13576
C	3.30249	4.157186	1.726177
H	3.146476	4.947981	2.469425
H	3.084254	3.204157	2.216844
C	2.356109	4.36424	0.531574
H	1.319961	4.337222	0.882553
C	2.650414	5.723274	-0.12374
H	2.484135	6.531115	0.59708
H	1.968003	5.894515	-0.96333
C	2.568099	3.241803	-0.49955
H	1.876064	3.371644	-1.33963
H	2.344923	2.270906	-0.04423
C	4.178977	2.113837	-2.03581
H	3.434059	2.238425	-2.8251
H	3.99215	1.148886	-1.56503
H	8.806533	2.085564	1.355491
C	4.821692	-0.94317	-0.34236
C	3.82955	-1.58549	-0.62615
C	2.665838	-2.2137	-0.90122
C	1.554315	-2.67623	-1.06321
C	6.962181	-3.04234	1.01169
C	6.752639	-3.49275	-0.34441
H	5.89042	-4.10969	-0.56011
C	7.582872	-3.10754	-1.33736
H	7.3961	-3.41885	-2.35711
C	8.144788	-2.26321	1.293065
H	8.340001	-1.964	2.313656
C	8.9701	-1.87197	0.299301
H	9.83206	-1.25581	0.515533
C	8.701066	-2.23383	-1.07202
C	9.473684	-1.73343	-2.10471
C	6.022818	-3.28774	1.996349
C	10.51575	-0.799	-1.85997
C	9.221124	-2.07299	-3.46141
C	6.173713	-2.74283	3.300465
C	4.820466	-3.99579	1.728947
N	11.33859	-0.01696	-1.64079
N	8.998693	-2.3503	-4.56148
N	3.839869	-4.56206	1.49824
N	6.310312	-2.2509	4.337801

APnA-TCNQ-8

Electronic Energy (EE):	-5546.306812	Hartree
EE + Thermal Free Energy Correction:	-5545.181664	Hartree
E (Thermal):	829.009	kcal/mol
Entropy (S):	414.424	cal/mol-kelvin

Imaginary Frequencies: 0

S	0.147667	-1.27752	3.462148
C	0.52969	-0.08484	2.112224
C	-0.45089	-0.02216	0.961026
C	-1.82428	-0.16851	1.147519
C	-2.68551	-0.23322	0.057461
C	-2.19743	-0.15077	-1.24673
C	-0.83358	0.091281	-1.43069
C	0.025933	0.153249	-0.34198
C	0.423801	-2.93704	2.692155
H	1.535358	-0.28402	1.740518
H	0.567733	0.869436	2.645807
H	-2.21433	-0.3071	2.148128
H	-3.7413	-0.41382	0.223693
H	-0.43188	0.159843	-2.43595
H	1.087884	0.280896	-0.50749
H	1.474731	-3.03679	2.426865
H	0.214986	-3.62141	3.516344
C	-3.09761	-0.44685	-2.41219
C	-0.47129	-3.21984	1.519908
S	-2.77166	-2.08348	-3.21254
H	-4.14751	-0.40603	-2.12304
H	-2.9464	0.258591	-3.23209
C	0.019664	-3.14056	0.197019
C	-1.82826	-3.4277	1.701932
C	-3.18948	-3.27294	-1.87571
C	-0.86645	-3.22849	-0.88915
C	-2.72188	-3.45922	0.619065
H	-2.23168	-3.46079	2.706532

C	-2.23256	-3.34071	-0.70553
H	-4.20004	-3.06583	-1.52564
H	-3.22497	-4.2351	-2.39409
H	-0.47651	-3.10171	-1.88993
S	-10.7962	-0.29631	-2.27046
S	-7.41564	1.125607	3.114759
C	-11.7851	-1.10751	-0.92951
H	-12.728	-0.57467	-0.80261
H	-12.0061	-2.09178	-1.3471
C	-11.0225	-1.19846	0.358224
C	-11.3403	-0.37647	1.443279
C	-10.4634	-0.24725	2.512972
H	-10.686	0.456359	3.307229
C	-9.23952	-0.91262	2.528107
C	-8.97326	-1.84344	1.49668
C	-9.87855	-1.98447	0.434799
H	-9.61459	-2.63397	-0.39084
C	-8.17915	-0.51825	3.51011
H	-7.39319	-1.26685	3.588318
H	-8.5999	-0.35707	4.504072
C	-10.5381	1.420276	-1.63119
H	-10.9482	2.080434	-2.39853
H	-11.1545	1.533796	-0.73672
C	-9.08801	1.839422	-1.32489
C	-9.08199	3.360195	-1.05058
H	-9.75101	3.585335	-0.21182
H	-9.46818	3.89611	-1.92518
C	-7.65692	3.841384	-0.7273
H	-7.6751	4.919245	-0.53761
C	-7.13973	3.112092	0.522422
H	-6.12883	3.458154	0.768505
H	-7.77795	3.338012	1.38208
C	-7.12334	1.590752	0.281964
C	-8.5434	1.124343	-0.07762
H	-8.53384	0.050771	-0.26233
H	-9.20728	1.306943	0.768301
C	-8.14553	1.553163	-2.51037
H	-8.51052	2.064504	-3.40904
H	-8.14578	0.480696	-2.72541
C	-6.71991	2.025284	-2.17919
H	-6.05946	1.800614	-3.02301

C	-6.72989	3.539876	-1.91633
H	-7.07382	4.072968	-2.80931
H	-5.71581	3.892938	-1.69969
C	-6.20842	1.293089	-0.92647
H	-5.18152	1.603405	-0.70399
H	-6.19014	0.21153	-1.10634
C	-6.56061	0.839062	1.49973
H	-5.52493	1.147193	1.661999
H	-6.55577	-0.23428	1.31528
H	-12.2517	0.210968	1.421024
C	-7.72179	-2.50307	1.419657
C	-6.59395	-2.93534	1.283457
C	-5.29614	-3.26251	1.088467
C	-4.11109	-3.43684	0.879051
S	7.132947	2.39071	2.580816
S	4.250952	1.855288	-3.22878
C	8.32414	1.36178	1.606582
H	9.225488	1.939318	1.401097
H	8.585187	0.565362	2.306393
C	7.708205	0.831044	0.34696
C	8.078909	1.338539	-0.90448
C	7.314281	1.06612	-2.03195
H	7.564184	1.538168	-2.97537
C	6.161955	0.286479	-1.95493
C	5.864315	-0.34067	-0.71985
C	6.645779	-0.06084	0.411468
H	6.346202	-0.47484	1.366078
C	5.185929	0.260575	-3.09046
H	4.485253	-0.56743	-3.01598
H	5.693703	0.184515	-4.05276
C	6.787034	3.824678	1.46354
H	7.045221	4.711764	2.045767
H	7.487156	3.767198	0.627194
C	5.34632	3.96326	0.937664
C	5.223877	5.335263	0.237348
H	5.955919	5.400119	-0.57607
H	5.457846	6.136454	0.947512
C	3.802541	5.526844	-0.32033
H	3.737206	6.503174	-0.81061
C	3.497434	4.42143	-1.34335
H	2.490572	4.558032	-1.75522

H	4.200365	4.478005	-2.18012
C	3.602764	3.036722	-0.67695
C	5.013208	2.869934	-0.08972
H	5.085738	1.897785	0.395857
H	5.745453	2.892198	-0.89807
C	4.311262	3.901959	2.077974
H	4.524234	4.683499	2.816874
H	4.391551	2.939849	2.59224
C	2.891646	4.082634	1.514786
H	2.16768	4.021183	2.333491
C	2.784177	5.454166	0.829094
H	2.974851	6.252042	1.554898
H	1.770794	5.605196	0.44186
C	2.59405	2.971842	0.491958
H	1.571336	3.072915	0.112602
H	2.660961	1.991378	0.977066
C	3.259145	1.912577	-1.6683
H	2.220227	2.022063	-1.98724
H	3.351506	0.939594	-1.18665
H	8.940161	1.992411	-0.9811
C	4.712666	-1.1459	-0.5716
C	3.683129	-1.77445	-0.41607
C	2.489518	-2.37676	-0.23374
C	1.36916	-2.80923	-0.04588
C	9.633788	-1.84957	0.266673
C	8.870362	-2.79149	1.05029
H	9.146764	-2.95236	2.084644
C	7.802506	-3.42915	0.522061
H	7.213419	-4.10547	1.128434
C	9.289816	-1.69146	-1.12353
H	9.883191	-1.0247	-1.73377
C	8.22586	-2.33416	-1.65359
H	7.962575	-2.18735	-2.69218
C	7.4031	-3.19949	-0.84601
C	6.242737	-3.7582	-1.35175
C	10.63535	-1.08779	0.847109
C	5.810511	-3.47423	-2.67568
C	5.407501	-4.59908	-0.56811
C	11.29706	-0.06774	0.113885
C	10.98171	-1.22556	2.217569
N	5.477442	-3.20716	-3.7498

N	4.735102	-5.28045	0.079657
N	11.24175	-1.34241	3.33826
N	11.78787	0.783863	-0.49551

APnA-TCNQ-9

Electronic Energy (EE):	-5546.306459	Hartree
EE + Thermal Free Energy Correction:	-5545.180272	Hartree
E (Thermal):	829.193	kcal/mol
Entropy (S):	412.856	cal/mol-kelvin

Imaginary Frequencies: 0

S	-1.19409	-0.5872	-2.67397
C	-1.7461	1.06379	-2.08857
C	-1.01448	1.635442	-0.8937
C	0.317557	1.335765	-0.62361
C	0.922858	1.778087	0.547191
C	0.214501	2.532556	1.481191
C	-1.09272	2.912927	1.169224
C	-1.69724	2.47242	-0.00185
C	-1.78154	-1.73723	-1.34572
H	-2.81686	1.026902	-1.88585
H	-1.61109	1.705359	-2.96469
H	0.863635	0.692006	-1.30009
H	1.944449	1.484018	0.761232
H	-1.65762	3.516343	1.870277
H	-2.72968	2.734843	-0.20197
H	-2.87001	-1.72943	-1.33425
H	-1.44934	-2.71232	-1.7057
C	0.804068	2.807424	2.833209
C	-1.21384	-1.44159	0.012611
S	0.107134	1.73204	4.167081
H	1.88938	2.692356	2.82517
H	0.569306	3.81577	3.180585
C	-1.97389	-0.73909	0.976001
C	0.107987	-1.73707	0.306487
C	0.647392	0.054325	3.646394

C	-1.36753	-0.31623	2.169764
C	0.727278	-1.27527	1.479245
H	0.712479	-2.25737	-0.4267
C	-0.02395	-0.52993	2.421998
H	1.729167	0.059118	3.518623
H	0.437603	-0.56146	4.525389
H	-1.94919	0.270047	2.868179
S	9.773324	1.65201	0.042785
S	8.251169	-4.60617	0.937855
C	9.811236	1.488943	1.889192
H	10.83848	1.599623	2.237668
H	9.234152	2.352353	2.222709
C	9.201171	0.19866	2.346799
C	9.99146	-0.87394	2.770694
C	9.444896	-2.14417	2.906482
H	10.08846	-2.98234	3.148854
C	8.102683	-2.38871	2.622763
C	7.270792	-1.28188	2.341724
C	7.832495	-0.00368	2.213088
H	7.195918	0.823051	1.931055
C	7.600975	-3.79067	2.470118
H	6.513187	-3.83576	2.468602
H	7.970481	-4.43852	3.267017
C	10.87154	0.272746	-0.52109
H	11.65681	0.744285	-1.11619
H	11.34319	-0.15268	0.367321
C	10.20451	-0.84021	-1.3473
C	11.30695	-1.79772	-1.85169
H	11.85235	-2.21608	-0.9974
H	12.03175	-1.24303	-2.4588
C	10.6861	-2.93473	-2.6836
H	11.48186	-3.60089	-3.03233
C	9.701831	-3.73116	-1.81192
H	9.264366	-4.55136	-2.39384
H	10.22925	-4.17995	-0.96514
C	8.583598	-2.80717	-1.2918
C	9.218303	-1.6561	-0.4971
H	8.4335	-0.99518	-0.12792
H	9.740127	-2.06477	0.367799
C	9.460148	-0.26772	-2.56727
H	10.15905	0.299755	-3.19416

H	8.683701	0.42303	-2.2354
C	8.828912	-1.40597	-3.38494
H	8.284033	-0.97945	-4.23243
C	9.937525	-2.3433	-3.88948
H	10.6322	-1.79181	-4.53278
H	9.508001	-3.14961	-4.49431
C	7.845285	-2.19765	-2.50391
H	7.378958	-2.99588	-3.09347
H	7.045648	-1.53532	-2.15941
C	7.560698	-3.57992	-0.44021
H	7.016868	-4.28122	-1.07699
H	6.82826	-2.89433	-0.01081
H	11.05309	-0.72525	2.935091
C	5.876232	-1.42724	2.127347
C	4.675527	-1.47654	1.943459
C	3.332119	-1.48312	1.772708
C	2.12371	-1.4404	1.641706
S	-11.5897	0.760874	2.26915
S	-8.55644	-1.47215	-3.05022
C	-11.2445	2.302065	1.300028
H	-12.1846	2.715482	0.933504
H	-10.8458	2.98359	2.0539
C	-10.2723	2.065219	0.183986
C	-10.7059	2.006775	-1.14387
C	-9.87528	1.50498	-2.13722
H	-10.2553	1.385191	-3.14549
C	-8.59536	1.041254	-1.84232
C	-8.10182	1.231197	-0.53014
C	-8.94828	1.746115	0.463595
H	-8.58276	1.802155	1.481739
C	-7.83942	0.225606	-2.84459
H	-6.78397	0.141482	-2.59336
H	-7.92142	0.648544	-3.84721
C	-12.3906	-0.35284	1.028058
H	-13.3552	-0.62608	1.461408
H	-12.5891	0.240857	0.133135
C	-11.6223	-1.63223	0.650146
C	-12.5514	-2.51027	-0.2179
H	-12.8584	-1.95121	-1.10957
H	-13.4622	-2.75348	0.341203
C	-11.8296	-3.80426	-0.63355

H	-12.5034	-4.41088	-1.24691
C	-10.5745	-3.45504	-1.44908
H	-10.0614	-4.37289	-1.76012
H	-10.8552	-2.9166	-2.35911
C	-9.62017	-2.58334	-0.61032
C	-10.3635	-1.31233	-0.17088
H	-9.69662	-0.69332	0.428867
H	-10.6424	-0.7374	-1.05419
C	-11.2109	-2.43905	1.896857
H	-12.0994	-2.6871	2.489801
H	-10.5602	-1.8272	2.528227
C	-10.4816	-3.72621	1.47778
H	-10.1823	-4.27998	2.373207
C	-11.4217	-4.59091	0.622585
H	-12.3097	-4.86572	1.202213
H	-10.9225	-5.52271	0.335338
C	-9.22693	-3.3686	0.660993
H	-8.68988	-4.28258	0.382552
H	-8.54361	-2.76441	1.268577
C	-8.33366	-2.24889	-1.38527
H	-7.77472	-3.16949	-1.568
H	-7.69377	-1.59225	-0.79476
H	-11.7209	2.299865	-1.38867
C	-6.7917	0.822501	-0.17826
C	-5.68001	0.441895	0.13378
C	-4.43547	0.017125	0.452001
C	-3.31073	-0.36402	0.712057
C	4.132705	3.337693	-0.73882
C	3.569563	4.25512	0.222691
H	4.156282	4.522672	1.092347
C	2.3227	4.751004	0.062648
H	1.901169	5.419518	0.80206
C	3.348727	3.017211	-1.90718
H	3.767857	2.347519	-2.64686
C	2.107523	3.522396	-2.07268
H	1.525133	3.263261	-2.94699
C	1.522268	4.400471	-1.08722
C	0.236985	4.883767	-1.23869
C	5.379335	2.764578	-0.54128
C	-0.55532	4.540369	-2.36795
C	-0.3551	5.739642	-0.27044

C	5.901546	1.815389	-1.46023
C	6.174581	3.078809	0.592696
N	-1.17695	4.253641	-3.2994
N	-0.81606	6.437811	0.526856
N	6.806988	3.337996	1.525053
N	6.273748	1.039173	-2.23119

APnA-TCNQ-10

Electronic Energy (EE):	-5546.306459	Hartree
EE + Thermal Free Energy Correction:	-5545.180272	Hartree
E (Thermal):	829.193	kcal/mol
Entropy (S):	412.856	cal/mol-kelvin

Imaginary Frequencies: 0

S	-0.40767	-0.62138	3.473922
C	-1.33265	-1.84226	2.454664
C	-0.5851	-2.46167	1.293441
C	0.764883	-2.79984	1.371016
C	1.44791	-3.24844	0.245961
C	0.803793	-3.3766	-0.98542
C	-0.5678	-3.11825	-1.04692
C	-1.24545	-2.66647	0.078446
C	-0.21941	0.838318	2.354698
H	-2.25302	-1.37408	2.102286
H	-1.62168	-2.59419	3.194036
H	1.300613	-2.64273	2.298814
H	2.511974	-3.44784	0.315081
H	-1.08787	-3.1937	-1.99652
H	-2.28354	-2.37615	-0.00647
H	-1.20654	1.230252	2.114871
H	0.289804	1.565437	2.989695
C	1.598328	-3.65953	-2.22888
C	0.579142	0.554893	1.115037
S	1.822231	-2.19299	-3.33647
H	2.583298	-4.06094	-1.98555
H	1.091572	-4.37667	-2.87777

C	-0.0587	0.360214	-0.13047
C	1.948623	0.356801	1.187145
C	2.780495	-1.04005	-2.27678
C	0.681593	-0.09283	-1.23451
C	2.685297	-0.12242	0.09152
H	2.457647	0.493443	2.133439
C	2.028018	-0.39102	-1.13455
H	3.660374	-1.55975	-1.89696
H	3.14319	-0.28331	-2.97815
H	0.161208	-0.30676	-2.15785
S	11.8693	1.308679	3.060652
S	9.87246	-0.82616	-2.76557
C	11.87637	-0.53394	3.257501
H	12.90579	-0.88264	3.345939
H	11.38501	-0.68082	4.221233
C	11.1515	-1.22794	2.143991
C	11.85529	-1.90154	1.141257
C	11.21794	-2.28958	-0.02999
H	11.79481	-2.73249	-0.83404
C	9.868908	-2.01563	-0.24307
C	9.121473	-1.46475	0.824299
C	9.775599	-1.08327	2.005809
H	9.205175	-0.58203	2.778308
C	9.279183	-2.15167	-1.61237
H	8.19117	-2.14922	-1.59225
H	9.612664	-3.0683	-2.1018
C	12.81813	1.573934	1.49511
H	13.63823	2.244425	1.761496
H	13.25734	0.615456	1.210031
C	12.04048	2.169447	0.307534
C	13.05456	2.504171	-0.80925
H	13.59374	1.595277	-1.10088
H	13.79894	3.215951	-0.43419
C	12.32965	3.097658	-2.03008
H	13.06445	3.327358	-2.80824
C	11.31507	2.078401	-2.57251
H	10.80314	2.489227	-3.45089
H	11.83124	1.16912	-2.89442
C	10.28199	1.725284	-1.48531
C	11.02026	1.168894	-0.25785
H	10.29589	0.924091	0.518644

H	11.53092	0.246408	-0.5348
C	11.30141	3.462957	0.701117
H	12.0162	4.194858	1.096114
H	10.58594	3.245165	1.499324
C	10.57013	4.046982	-0.51886
H	10.03777	4.955564	-0.22026
C	11.59346	4.381923	-1.61591
H	12.30809	5.126171	-1.24773
H	11.0886	4.820666	-2.48342
C	9.556898	3.020729	-1.05712
H	9.015236	3.443779	-1.91088
H	8.813789	2.787285	-0.28615
C	9.227414	0.735325	-2.0101
H	8.642238	1.217122	-2.79667
H	8.535449	0.458448	-1.21398
H	12.92092	-2.06785	1.254609
C	7.735619	-1.20348	0.686764
C	6.554058	-0.94545	0.562611
C	5.240003	-0.66172	0.407924
C	4.061527	-0.40272	0.254961
S	-9.59447	1.512943	-2.98789
S	-6.96315	1.858789	2.952406
C	-9.62304	-0.24822	-2.42053
H	-10.6546	-0.55571	-2.24574
H	-9.24457	-0.80014	-3.28233
C	-8.77389	-0.45268	-1.202
C	-9.35388	-0.64789	0.05524
C	-8.58972	-0.54597	1.211446
H	-9.06725	-0.62107	2.181457
C	-7.2285	-0.2477	1.153707
C	-6.60894	-0.19131	-0.11728
C	-7.39161	-0.29874	-1.27627
H	-6.91852	-0.16207	-2.24086
C	-6.49507	0.154631	2.394001
H	-5.41513	0.094387	2.273977
H	-6.78564	-0.45979	3.247105
C	-10.38	2.426203	-1.58313
H	-11.2288	2.958105	-2.01836
H	-10.7794	1.683056	-0.88949
C	-9.48649	3.425595	-0.8267
C	-10.3751	4.21704	0.158909

H	-10.8685	3.522366	0.848772
H	-11.1644	4.741967	-0.39133
C	-9.52623	5.227416	0.950652
H	-10.1729	5.776769	1.641981
C	-8.44626	4.480051	1.749242
H	-7.84527	5.192851	2.326279
H	-8.91342	3.796625	2.464479
C	-7.53571	3.682911	0.795524
C	-8.39913	2.704681	-0.01537
H	-7.76183	2.144337	-0.69871
H	-8.86633	1.990866	0.663713
C	-8.80975	4.423287	-1.78632
H	-9.572	4.95718	-2.36617
H	-8.18423	3.877217	-2.49844
C	-7.9551	5.424872	-0.99233
H	-7.46929	6.116493	-1.68762
C	-8.85495	6.208953	-0.02342
H	-9.61433	6.765044	-0.58382
H	-8.26259	6.943065	0.533377
C	-6.87689	4.668991	-0.19485
H	-6.24892	5.381584	0.351922
H	-6.22112	4.118738	-0.87924
C	-6.41931	2.951412	1.56115
H	-5.75303	3.687068	2.017266
H	-5.81883	2.349897	0.877956
H	-10.4224	-0.81618	0.131429
C	-5.21562	0.031377	-0.23081
C	-4.01777	0.229826	-0.29314
C	-2.67598	0.395171	-0.30043
C	-1.46333	0.457728	-0.2442
C	-5.88475	-3.40014	-1.36427
C	-7.18033	-3.4391	-0.73
H	-8.06647	-3.41396	-1.34867
C	-7.28909	-3.47102	0.616123
H	-8.26305	-3.47424	1.08477
C	-4.71578	-3.52665	-0.52818
H	-3.74342	-3.57513	-0.99799
C	-4.82237	-3.54412	0.816102
H	-3.93367	-3.59948	1.431151
C	-6.11381	-3.45655	1.454951
C	-6.21274	-3.33745	2.829734

C	-5.7498	-3.20888	-2.72745
C	-5.0539	-3.29896	3.652331
C	-7.47175	-3.18188	3.468608
C	-4.46778	-3.16458	-3.34075
C	-6.88199	-2.97954	-3.55385
N	-4.09122	-3.26246	4.291242
N	-8.50755	-3.02404	3.957411
N	-7.82093	-2.76501	-4.19319
N	-3.40836	-3.13508	-3.80227

APnA-TCNQ-11

Electronic Energy (EE):	-5546.304602	Hartree
EE + Thermal Free Energy Correction:	-5545.179288	Hartree
E (Thermal):	829.028	kcal/mol
Entropy (S):	414.139	cal/mol-kelvin

Imaginary Frequencies: 0

S	-0.65349	-2.53678	-3.42486
C	0.223911	-3.66685	-2.26154
C	-0.27204	-3.68457	-0.83536
C	-1.62774	-3.81415	-0.53245
C	-2.06926	-3.7484	0.782629
C	-1.17099	-3.55139	1.833655
C	0.19286	-3.4895	1.539769
C	0.631761	-3.55467	0.222582
C	-0.25916	-0.8599	-2.76459
H	1.28872	-3.44426	-2.30291
H	0.079333	-4.64203	-2.73535
H	-2.34693	-3.90862	-1.33749
H	-3.13242	-3.80439	0.991271
H	0.915089	-3.33214	2.333331
H	1.688491	-3.45825	0.009449
H	0.822565	-0.73604	-2.72851
H	-0.64958	-0.18878	-3.53252
C	-1.68709	-3.31505	3.224361
C	-0.88855	-0.58416	-1.42627

S	-1.62645	-1.54946	3.783343
H	-2.71398	-3.66901	3.327237
H	-1.07718	-3.82451	3.973048
C	-0.09866	-0.38001	-0.2747
C	-2.26441	-0.64438	-1.27032
C	-2.67298	-0.70234	2.534364
C	-0.70275	-0.36091	0.995293
C	-2.86787	-0.63197	-0.00284
H	-2.89079	-0.80425	-2.13879
C	-2.06171	-0.54112	1.159154
H	-3.62857	-1.22213	2.461681
H	-2.8799	0.272933	2.983558
H	-0.06875	-0.29642	1.868766
S	-12.1151	-0.26952	-2.97681
S	-9.9898	0.77562	3.096713
C	-12.2045	-1.9486	-2.19808
H	-13.2499	-2.23605	-2.0814
H	-11.7577	-2.59609	-2.95498
C	-11.4649	-2.01129	-0.89571
C	-12.1541	-2.03902	0.320471
C	-11.4866	-1.80579	1.515803
H	-12.0483	-1.74313	2.441059
C	-10.1207	-1.53318	1.539416
C	-9.39527	-1.64681	0.330472
C	-10.0805	-1.88869	-0.87032
H	-9.52171	-1.88462	-1.79828
C	-9.48356	-0.97924	2.775652
H	-8.39751	-1.03834	2.738605
H	-9.82668	-1.5006	3.67088
C	-12.9965	0.809025	-1.76013
H	-13.7978	1.290923	-2.32468
H	-13.4638	0.155534	-1.02026
C	-12.1556	1.886623	-1.0522
C	-13.1185	2.792274	-0.25188
H	-13.6801	2.185758	0.468246
H	-13.8488	3.24893	-0.92978
C	-12.3318	3.888646	0.487325
H	-13.0309	4.517848	1.047342
C	-11.3368	3.240007	1.462768
H	-10.781	4.015528	2.00323
H	-11.8742	2.647158	2.208838

C	-10.3544	2.333111	0.696618
C	-11.1538	1.265376	-0.06642
H	-10.4657	0.623908	-0.6166
H	-11.688	0.639354	0.648487
C	-11.3842	2.761545	-2.05906
H	-12.085	3.223275	-2.76492
H	-10.7042	2.133043	-2.64143
C	-10.5916	3.849861	-1.31607
H	-10.0375	4.452842	-2.04233
C	-11.5646	4.745693	-0.53238
H	-12.2644	5.231835	-1.22097
H	-11.0151	5.540299	-0.01601
C	-9.59845	3.193415	-0.3406
H	-9.0135	3.965569	0.172112
H	-8.89063	2.564589	-0.89251
C	-9.31779	1.698946	1.640499
H	-8.68718	2.484957	2.062052
H	-8.66574	1.020484	1.089002
H	-13.2289	-2.18378	0.323648
C	-7.9951	-1.43367	0.290498
C	-6.7979	-1.23129	0.22635
C	-5.46446	-1.013	0.156892
C	-4.26554	-0.8176	0.092966
S	8.367601	2.736384	3.249694
S	7.243982	1.628754	-3.07583
C	8.784054	0.941886	3.058467
H	9.858088	0.800481	3.181698
H	8.280583	0.47913	3.909429
C	8.301172	0.392412	1.750417
C	9.199285	0.136769	0.708589
C	8.738301	-0.08807	-0.58293
H	9.448972	-0.19652	-1.39438
C	7.37859	-0.07332	-0.87866
C	6.462842	0.037305	0.194716
C	6.940749	0.268205	1.49696
H	6.223048	0.438386	2.29029
C	6.912784	-0.02054	-2.29939
H	5.855383	-0.26194	-2.39444
H	7.480879	-0.70616	-2.93031
C	9.323107	3.543554	1.886353
H	9.952552	4.290117	2.375357

H	9.985455	2.789284	1.456027
C	8.500973	4.220568	0.774764
C	9.476471	5.002386	-0.1336
H	10.22164	4.315127	-0.55093
H	10.01952	5.747451	0.458842
C	8.707552	5.696163	-1.27183
H	9.415249	6.243487	-1.90227
C	7.980229	4.64143	-2.12106
H	7.440304	5.127609	-2.94219
H	8.705262	3.956584	-2.57072
C	6.992363	3.844361	-1.24747
C	7.765981	3.18798	-0.09367
H	7.071638	2.627769	0.532476
H	8.487226	2.481186	-0.50411
C	7.468844	5.20897	1.35109
H	7.977338	5.962637	1.964004
H	6.775842	4.672578	2.005815
C	6.696258	5.892038	0.210649
H	5.957201	6.578457	0.635351
C	7.679542	6.671992	-0.67699
H	8.186732	7.443136	-0.08712
H	7.139717	7.183778	-1.48101
C	5.971434	4.829559	-0.63533
H	5.398478	5.315345	-1.43327
H	5.256756	4.278582	-0.01335
C	6.219598	2.80236	-2.07533
H	5.582405	3.316068	-2.79845
H	5.567584	2.210209	-1.43179
H	10.26553	0.171655	0.90197
C	5.068401	-0.02176	-0.02394
C	3.860784	-0.08475	-0.16479
C	2.519332	-0.1802	-0.27488
C	1.307678	-0.27603	-0.33246
C	9.284889	-3.22002	-0.21412
C	8.49975	-3.46014	-1.40083
H	9.013035	-3.62982	-2.33885
C	7.149768	-3.43265	-1.35633
H	6.570403	-3.58073	-2.25885
C	8.585543	-3.04206	1.033925
H	9.163224	-2.88534	1.934838
C	7.235013	-3.04879	1.083781

H	6.722018	-2.90088	2.024822
C	6.450297	-3.20944	-0.11324
C	5.068371	-3.13866	-0.07735
C	10.66563	-3.12292	-0.27981
C	4.349298	-2.99992	1.140783
C	4.293552	-3.19605	-1.26605
C	11.42896	-2.76731	0.863614
C	11.36883	-3.28715	-1.50262
N	3.760328	-2.91017	2.131257
N	3.672663	-3.2258	-2.24047
N	11.92318	-3.41524	-2.50936
N	12.01831	-2.44023	1.803455

APnA-TCNQ-12

Electronic Energy (EE):	-5546.298623	Hartree
EE + Thermal Free Energy Correction:	-5545.173355	Hartree
E (Thermal):	829.273	kcal/mol
Entropy (S):	415.056	cal/mol-kelvin

Imaginary Frequencies: 0

S	1.42437	1.617855	3.50702
C	2.187838	2.700704	2.235229
C	1.380564	2.941354	0.97509
C	-0.01472	2.93491	0.971663
C	-0.72515	3.018033	-0.2198
C	-0.06283	3.121228	-1.44184
C	1.33317	3.224225	-1.43359
C	2.042348	3.132479	-0.24272
C	1.531274	-0.06582	2.745214
H	3.168478	2.300605	1.975282
H	2.350392	3.631517	2.783659
H	-0.54344	2.782134	1.903515
H	-1.80683	2.944852	-0.20108
H	1.867249	3.313651	-2.37299
H	3.126148	3.147382	-0.2647
H	2.580471	-0.33315	2.631039

H	1.095556	-0.70891	3.511594
C	-0.82199	2.985313	-2.72917
C	0.785718	-0.19085	1.448334
S	-0.65143	1.320948	-3.51772
H	-1.88338	3.200137	-2.59412
H	-0.43552	3.652371	-3.50161
C	1.467445	-0.12994	0.211395
C	-0.59871	-0.25084	1.432862
C	-1.42194	0.211435	-2.27407
C	0.735711	-0.05475	-0.98499
C	-1.33139	-0.15526	0.238328
H	-1.14033	-0.29311	2.370135
C	-0.6468	-0.01688	-0.99434
H	-2.4224	0.579967	-2.04748
H	-1.55144	-0.72891	-2.81735
H	1.273299	0.060443	-1.91616
S	-10.369	-2.60721	3.028879
S	-8.32451	-1.18539	-2.99367
C	-10.6752	-0.78434	2.897508
H	-11.7498	-0.59988	2.88029
H	-10.2804	-0.39913	3.83954
C	-9.99088	-0.17776	1.709833
C	-10.7185	0.191391	0.574499
C	-10.0688	0.475524	-0.61959
H	-10.6497	0.674913	-1.51302
C	-8.6821	0.395825	-0.72389
C	-7.93523	0.164716	0.455128
C	-8.60294	-0.11429	1.65746
H	-8.0171	-0.37559	2.530299
C	-8.02593	0.394387	-2.06946
H	-6.95473	0.574424	-2.00342
H	-8.46178	1.147979	-2.72756
C	-11.1567	-3.28287	1.497699
H	-11.8806	-4.02354	1.844545
H	-11.7169	-2.47026	1.030054
C	-10.2171	-3.93455	0.467056
C	-11.0887	-4.61139	-0.61466
H	-11.7388	-3.8636	-1.08368
H	-11.7398	-5.36168	-0.15156
C	-10.1993	-5.27512	-1.68068
H	-10.8349	-5.74764	-2.43624

C	-9.31641	-4.21056	-2.35126
H	-8.68807	-4.67439	-3.12096
H	-9.94128	-3.46382	-2.84998
C	-8.42743	-3.51762	-1.30073
C	-9.32568	-2.89055	-0.22329
H	-8.70326	-2.40339	0.527011
H	-9.9513	-2.12418	-0.68127
C	-9.31819	-5.00661	1.112989
H	-9.93858	-5.76782	1.601084
H	-8.70074	-4.5451	1.889062
C	-8.42446	-5.66073	0.046402
H	-7.78181	-6.40818	0.522217
C	-9.30687	-6.33572	-1.01609
H	-9.92385	-7.11319	-0.55244
H	-8.68249	-6.82628	-1.77072
C	-7.54479	-4.58839	-0.62124
H	-6.8873	-5.05455	-1.36401
H	-6.90108	-4.1122	0.1271
C	-7.50256	-2.47123	-1.94661
H	-6.7964	-2.97605	-2.60977
H	-6.91921	-1.95566	-1.18292
H	-11.8023	0.196605	0.611768
C	-6.51945	0.118829	0.424859
C	-5.30615	0.04594	0.39909
C	-3.95602	-0.03167	0.352241
C	-2.74327	-0.10685	0.295738
S	10.53082	-1.63148	-3.06595
S	8.489272	-1.62522	3.12268
C	10.80321	0.121268	-2.53023
H	11.87415	0.317633	-2.47009
H	10.39928	0.699234	-3.36365
C	10.11047	0.435473	-1.23849
C	10.83463	0.561978	-0.04926
C	10.18422	0.561379	1.177737
H	10.76476	0.572656	2.093292
C	8.800178	0.428065	1.261624
C	8.053446	0.443357	0.060128
C	8.721745	0.452797	-1.17391
H	8.137984	0.374142	-2.08299
C	8.151513	0.113598	2.573792
H	7.07633	0.280053	2.550719

H	8.574954	0.711889	3.382258
C	11.33469	-2.61914	-1.72409
H	12.06937	-3.25099	-2.22806
H	11.88334	-1.92266	-1.0863
C	10.40927	-3.50083	-0.866
C	11.29474	-4.39181	0.033874
H	11.9367	-3.76019	0.659024
H	11.95387	-5.00876	-0.58762
C	10.41887	-5.29161	0.923425
H	11.06395	-5.91354	1.552093
C	9.52478	-4.41804	1.817795
H	8.90654	-5.05215	2.464367
H	10.14169	-3.79346	2.470698
C	8.621335	-3.51949	0.951714
C	9.505925	-2.65235	0.042471
H	8.873318	-2.01887	-0.57874
H	10.12244	-1.99884	0.660098
C	9.52258	-4.41215	-1.73649
H	10.15132	-5.03463	-2.38437
H	8.895862	-3.79598	-2.38759
C	8.641933	-5.30268	-0.84458
H	8.007783	-5.93252	-1.47637
C	9.537636	-6.18726	0.037641
H	10.163	-6.83169	-0.58983
H	8.922904	-6.84373	0.663012
C	7.750597	-4.42153	0.048731
H	7.102486	-5.05262	0.667551
H	7.09756	-3.79754	-0.57194
C	7.683378	-2.66123	1.818153
H	6.991267	-3.31477	2.353992
H	7.085244	-1.99973	1.190737
H	11.91789	0.599857	-0.08387
C	6.63996	0.347671	0.07815
C	5.430664	0.221831	0.086112
C	4.085531	0.079634	0.116219
C	2.877122	-0.04654	0.161098
C	-0.73809	6.324268	-1.32936
C	0.451269	6.234205	-0.51888
H	1.415827	6.255016	-1.00645
C	0.368418	6.097693	0.821744
H	1.267229	6.012401	1.416377

C	-2.01822	6.330518	-0.65986
H	-2.91644	6.425027	-1.25675
C	-2.1007	6.197536	0.681164
H	-3.06568	6.18267	1.171695
C	-0.9113	6.04096	1.485585
C	-1.00096	5.815288	2.847326
C	-0.65939	6.383763	-2.70927
C	-2.2521	5.753125	3.51801
C	0.161848	5.57971	3.629296
C	-1.82204	6.461038	-3.52267
C	0.59028	6.299802	-3.37988
N	-3.27771	5.703472	4.049093
N	1.12458	5.369344	4.233537
N	1.614859	6.196476	-3.90555
N	-2.77831	6.512293	-4.17034

5.0 AFA-TCNQ coordinates

AFA

Electronic Energy (EE): -3953.054082 Hartree

EE + Thermal Free Energy Correction: -3952.120348 Hartree

E (Thermal): 678.558 kcal/mol

Entropy (S): 312.673 cal/mol-kelvin

Imaginary frequencies: 0

0	1		
H	1.32506500	-3.17901400	-2.90549400
C	1.64799900	-2.84830800	-1.92529700
C	2.49642000	-1.98812000	0.62418900
C	0.71761800	-2.51385900	-0.94021200
C	3.00091000	-2.75344700	-1.63615200
C	3.44310500	-2.32499800	-0.36640400
C	1.15065800	-2.08481600	0.33196900
H	3.73983000	-3.00820000	-2.38516500
H	2.84090800	-1.65986600	1.59743000

C	-0.74243300	-2.51924100	-0.96016900
C	-3.48391800	-2.35132600	-0.46085200
C	-1.64305600	-2.85955200	-1.97057700
C	-1.21324000	-2.09421600	0.29988700
C	-2.56716100	-2.00860900	0.55557900
C	-3.00403100	-2.77449100	-1.71860700
H	-1.29099700	-3.18697000	-2.94181200
H	-2.94048900	-1.68411600	1.51941300
H	-3.72034000	-3.03364000	-2.48779200
C	-0.04464200	-1.78697600	1.21092000
H	-0.05429300	-2.41213500	2.10999000
H	-0.05342300	-0.74448300	1.54619400
C	-4.87594700	-2.27162100	-0.22107400
C	4.82751000	-2.23409900	-0.08935500
C	-6.07187600	-2.20301100	-0.01349400
C	-7.40290200	-2.12032000	0.22005200
C	6.01701100	-2.15570800	0.14936000
C	7.34228200	-2.06394700	0.41060200
C	8.53417100	-1.98176000	0.63558100
S	-9.61972000	0.83277700	3.15207400
S	-12.79580900	-0.26308600	-2.43692000
C	-9.57270600	-1.01533500	3.00123100
H	-9.89154800	-1.34917500	3.99014500
C	-10.48927300	-1.51896600	1.93006300
C	-9.98942400	-1.91757200	0.66813600
C	-10.87907600	-2.10712300	-0.40045800
H	-10.47482000	-2.32486000	-1.38156200
C	-12.24527600	-1.90119800	-0.24444900
C	-12.73967700	-1.64580500	1.03799500
C	-11.87004800	-1.46164000	2.10496100
H	-12.26259600	-1.17812000	3.07505400
C	-13.13450600	-1.79251800	-1.44613200
H	-14.18912900	-1.81533900	-1.16964600
H	-12.95490500	-2.59549300	-2.16362500
C	-8.98236800	1.42270300	1.51792300
H	-8.13763800	2.07367700	1.75443200
H	-8.58638300	0.55793500	0.98432800
C	-9.97991700	2.19000800	0.63256100
C	-9.21367500	2.74134300	-0.59066100
H	-8.75252800	1.91194200	-1.13873100
H	-8.40279900	3.39873200	-0.25650600
C	-10.16978500	3.51684400	-1.51464700
H	-9.60944500	3.89763900	-2.37440800
C	-11.28506500	2.58113000	-2.00969100
H	-11.96235900	3.12549800	-2.67873900

H	-10.85545600	1.75564400	-2.58456300
C	-12.07387800	2.01617500	-0.81268000
C	-11.09980900	1.27363400	0.11544000
H	-11.64754700	0.86335000	0.96394600
H	-10.65973200	0.43780600	-0.42794200
C	-10.61212000	3.37426700	1.38900100
H	-9.82437600	4.04449200	1.75338600
H	-11.15094700	3.00145800	2.26509800
C	-11.57172900	4.14258900	0.46640900
H	-12.02322500	4.97109800	1.02145100
C	-10.79289000	4.68984700	-0.74064600
H	-10.01066400	5.37757300	-0.40125700
H	-11.46122800	5.25903700	-1.39606700
C	-12.68245500	3.19764000	-0.02434800
H	-13.38563100	3.74657000	-0.66131600
H	-13.25144400	2.81455700	0.83085800
C	-13.22737900	1.10454200	-1.26892800
H	-13.97755100	1.70541700	-1.78792300
H	-13.71174900	0.65370500	-0.40004200
H	-8.54477500	-1.33621800	2.84457100
H	-13.80765500	-1.53085000	1.18741300
C	-8.59638500	-2.04086200	0.43744300
S	11.59906600	1.63837500	3.19446400
S	10.80430400	-1.13627100	-2.65064700
C	12.08467500	-0.12551800	3.49095400
H	11.56352200	-0.37194500	4.41804600
C	11.68135100	-1.02464400	2.36149700
C	12.63108600	-1.50888300	1.45712600
C	12.23279900	-2.09109200	0.26083500
H	12.97944000	-2.38277400	-0.46925200
C	10.88553700	-2.20373700	-0.07479600
C	9.92083500	-1.84738400	0.89657000
C	10.33639200	-1.26611600	2.10436000
H	9.58288900	-0.91695200	2.79986700
C	10.48388200	-2.53718800	-1.47795000
H	9.43788800	-2.82944300	-1.54594800
H	11.09472200	-3.34324700	-1.88806400
C	12.58381500	2.10522700	1.70010600
H	13.16465200	2.98184500	1.99557700
H	13.28905700	1.29443900	1.50500600
C	11.79007400	2.42922800	0.42155900
C	12.77816100	2.99229200	-0.62458200
H	13.56623700	2.25560900	-0.81971600
H	13.26566300	3.89101200	-0.22941000
C	12.03951300	3.32804100	-1.93204000

H	12.75597500	3.72464800	-2.65849400
C	11.39102600	2.05505300	-2.49880600
H	10.87304600	2.28360000	-3.43794000
H	12.15995100	1.31076100	-2.72601000
C	10.39246900	1.46816400	-1.48260100
C	11.13371600	1.17187300	-0.16956500
H	10.43156400	0.76295100	0.55657700
H	11.89675600	0.41499000	-0.35201500
C	10.69761500	3.48471500	0.68041700
H	11.14956800	4.39531100	1.09183900
H	9.99461000	3.10549500	1.42772200
C	9.95513100	3.80973300	-0.62634200
H	9.17309500	4.54831000	-0.42358900
C	10.95044200	4.37607300	-1.65194600
H	11.40143500	5.29762200	-1.26775200
H	10.43085600	4.63393400	-2.58123100
C	9.30963300	2.53034700	-1.18839500
H	8.75758800	2.76317300	-2.10619900
H	8.58694200	2.12767700	-0.46969800
C	9.69628900	0.21275500	-2.03640400
H	9.07848300	0.49267100	-2.89269300
H	9.03365700	-0.21815300	-1.28505600
H	13.68681100	-1.37336800	1.66501500
H	13.15713200	-0.17583700	3.68194300

AFA-TCNQ-1

Electronic Energy (EE): -4631.888876 Hartree

EE + Thermal Free Energy Correction: -4630.841166 Hartree

E (Thermal): 769.183 kcal/mol

Entropy (S): 379.747 cal/mol-kelvin

Imaginary frequencies: 0

0	1		
H	1.18440200	-3.71347500	-2.76486900
C	1.56521400	-3.71441800	-1.75065600
C	2.55658000	-3.70562300	0.89767100
C	0.69335800	-3.74068800	-0.65761400

C	2.92793300	-3.67057100	-1.52071000
C	3.44089400	-3.64372700	-0.20255600
C	1.19828800	-3.75070200	0.66428900
H	3.62361400	-3.61693000	-2.34743100
H	2.96084200	-3.68125600	1.90190300
C	-0.75451400	-3.71563300	-0.60570400
C	-3.45874500	-3.53623900	0.04525200
C	-1.70088900	-3.65837200	-1.63354200
C	-1.16393100	-3.71074200	0.74901400
C	-2.50016900	-3.62425800	1.07923800
C	-3.04143500	-3.57276900	-1.30637300
H	-1.39395000	-3.66688200	-2.67245300
H	-2.83036100	-3.59122600	2.10994900
H	-3.79258300	-3.49670800	-2.08115300
C	0.04993100	-3.79398200	1.64952400
H	0.05465700	-4.72784900	2.22193900
H	0.08976300	-2.97645000	2.37413000
C	-4.82583800	-3.35253700	0.34007000
C	4.82693300	-3.48075500	0.00058700
C	-5.99173800	-3.07457100	0.54172300
C	-7.26694800	-2.66704600	0.72472300
C	5.99867700	-3.19604900	0.15159500
C	7.27289800	-2.77071300	0.29379800
C	8.37647300	-2.26844600	0.37863400
S	-8.31812200	0.97578000	3.36145000
S	-10.61946000	0.93731400	-2.72937100
C	-9.16079500	-0.67085200	3.24146100
H	-9.75339000	-0.71648300	4.15672000
C	-10.02892600	-0.77493300	2.02514300
C	-9.60115500	-1.47423100	0.87355100
C	-10.29610700	-1.31854300	-0.33631000
H	-9.89728200	-1.78716600	-1.22754600
C	-11.38015300	-0.45577000	-0.43510000
C	-11.87055900	0.13315700	0.73444400
C	-11.20819900	-0.03660100	1.94325600
H	-11.55398400	0.49401000	2.82315300
C	-11.88193100	-0.03485000	-1.78447600
H	-12.80310800	0.54382800	-1.70796700
H	-12.07313900	-0.88965500	-2.43639500
C	-7.15801300	0.95941600	1.92168800
H	-6.16845200	1.14208400	2.34225700
H	-7.14611300	-0.05400100	1.52476800
C	-7.43459600	1.97153300	0.79947000
C	-6.31177500	1.82855700	-0.25233300
H	-6.30053800	0.80531700	-0.64118000

H	-5.33857700	2.00699800	0.21928500
C	-6.52649700	2.82473600	-1.40430200
H	-5.72404300	2.70132900	-2.13791000
C	-7.87854400	2.54857900	-2.08367500
H	-8.03112400	3.24894500	-2.91379000
H	-7.88488500	1.53955000	-2.50512200
C	-9.02422500	2.68865100	-1.06217700
C	-8.77486400	1.70639100	0.09370300
H	-9.58580600	1.78044200	0.82000200
H	-8.77716200	0.69165900	-0.30135200
C	-7.42753500	3.41582800	1.33430600
H	-6.47184600	3.61995100	1.83151600
H	-8.21467300	3.53064300	2.08631000
C	-7.64652800	4.41016000	0.18323200
H	-7.65030500	5.43076100	0.57943200
C	-6.51661300	4.25813600	-0.84881600
H	-5.55043500	4.47591000	-0.38020500
H	-6.64979000	4.97836500	-1.66365700
C	-8.99941000	4.12444400	-0.49091000
H	-9.17583400	4.84671600	-1.29638500
H	-9.81124800	4.24550300	0.23600400
C	-10.40404100	2.46587100	-1.70949300
H	-10.62136200	3.29574800	-2.38554800
H	-11.17904800	2.45828100	-0.93965500
H	-8.40978700	-1.45813100	3.27930800
H	-12.74148900	0.77758500	0.68454500
C	-8.37538700	-2.18287900	0.84684100
S	9.75724800	1.97060200	3.22605300
S	9.08585400	-0.07523000	-2.92053700
C	11.15474000	0.77062900	3.02576400
H	11.10572500	0.17992600	3.94266400
C	11.00092000	-0.07822700	1.79928300
C	11.80780300	0.12635600	0.67580500
C	11.47682800	-0.44368900	-0.54684200
H	12.06519000	-0.20712200	-1.42618800
C	10.32796300	-1.21761600	-0.69567400
C	9.58356900	-1.53415500	0.46466200
C	9.94063400	-0.97086400	1.69928600
H	9.30201400	-1.14893500	2.55577200
C	9.81357900	-1.54894600	-2.06267300
H	9.07678700	-2.34974300	-2.04089900
H	10.62456700	-1.84613300	-2.72974000
C	9.92166800	3.06894700	1.74658400
H	10.01454800	4.08121200	2.14631000
H	10.86636100	2.82163200	1.25710100

C	8.77434200	3.03299800	0.72000800
C	9.00412800	4.18400600	-0.28528500
H	9.98062500	4.06100300	-0.76840800
H	9.02466000	5.14228100	0.24663600
C	7.89230500	4.19761800	-1.34849500
H	8.07377900	5.01962500	-2.04843400
C	7.89699400	2.86484900	-2.11363300
H	7.11567800	2.86923900	-2.88274100
H	8.85467700	2.72828800	-2.62540000
C	7.66110500	1.69264500	-1.14266400
C	8.75421300	1.70963600	-0.06161100
H	8.58121800	0.89142000	0.63645100
H	9.72635200	1.54659100	-0.52881200
C	7.39961300	3.23239000	1.38756900
H	7.38893600	4.17743800	1.94408500
H	7.22304400	2.42839200	2.10758300
C	6.29036500	3.23584900	0.32227000
H	5.32033500	3.36187000	0.81387100
C	6.52964000	4.39113400	-0.66305900
H	6.50592300	5.34943000	-0.13237400
H	5.73335000	4.41819600	-1.41503600
C	6.30015600	1.89984700	-0.44091400
H	5.49584900	1.88739000	-1.18488900
H	6.11665700	1.07364300	0.25303900
C	7.60942400	0.34934300	-1.88879700
H	6.76241600	0.34690900	-2.57696200
H	7.45246700	-0.46813500	-1.18662500
H	12.66585700	0.78590000	0.74663500
H	12.10081200	1.31303300	3.02329000
C	1.46987900	-0.52888500	-0.40825400
C	0.68327600	-0.53284400	-1.61569300
H	1.19778800	-0.54474400	-2.56786100
C	-0.67034300	-0.50935400	-1.57046800
H	-1.24744500	-0.50198700	-2.48608100
C	0.76514100	-0.52210600	0.84831300
H	1.34211700	-0.52311700	1.76407200
C	-0.58665000	-0.50124300	0.89349600
H	-1.10059000	-0.48556200	1.84586100
C	-1.37383400	-0.48263800	-0.31322900
C	-2.75921700	-0.42329200	-0.25614700
C	2.85683600	-0.51521000	-0.44367500
C	-3.44254100	-0.37549200	0.98725900
C	-3.55765400	-0.37709000	-1.42925100
C	3.62835100	-0.47998200	0.74789000
C	3.57651300	-0.50436900	-1.66747500

N	-3.97487600	-0.33636100	2.01285800
N	-4.19949200	-0.33583300	-2.39022100
N	4.15804300	-0.49177300	-2.66683200
N	4.24458600	-0.44689300	1.72563300

AFA-TCNQ-2

Electronic Energy (EE): -4631.885242 Hartree

EE + Thermal Free Energy Correction: -4630.838802 Hartree

E (Thermal): 769.271 kcal/mol

Entropy (S): 379.717 cal/mol-kelvin

Imaginary frequencies: 0

0	1		
H	-0.58416900	-1.26165400	-3.96601800
C	-1.06977200	-0.70786300	-3.17160100
C	-2.33243800	0.72793600	-1.09015900
C	-0.37355000	0.26303900	-2.44531800
C	-2.39090500	-0.96698400	-2.85263300
C	-3.03311900	-0.27128200	-1.80272000
C	-1.01790300	0.98718000	-1.41418600
H	-2.94310000	-1.73255400	-3.38098200
H	-2.83435400	1.25286800	-0.28702400
C	1.01049600	0.68863600	-2.52004800
C	3.56125900	1.75344900	-2.15931700
C	2.05596200	0.24652900	-3.33662800
C	1.23702200	1.68335000	-1.53965900
C	2.49534300	2.21770800	-1.35720600
C	3.31916600	0.77928200	-3.15634700
H	1.88814700	-0.51190900	-4.09179600
H	2.69291300	2.95935100	-0.59341700
H	4.15231200	0.43293000	-3.75320500
C	-0.05331400	1.98318700	-0.80778700
H	-0.38175800	3.01226900	-0.98921000
H	0.04334400	1.86628200	0.27479700
C	4.87974500	2.20393800	-1.93852800
C	-4.35686600	-0.59789100	-1.43896500
C	6.04087500	2.47445600	-1.70275500
C	7.34211400	2.67750800	-1.40034100

C	-5.46648000	-0.91427900	-1.05802800
C	-6.68836900	-1.26758500	-0.59989600
C	-7.77245900	-1.58658300	-0.15268900
S	8.93072700	2.27242500	2.91285600
S	11.99635800	-1.13444800	-1.71428700
C	9.31724400	3.55455800	1.63118100
H	9.73916300	4.37371100	2.21593800
C	10.29212000	3.05771400	0.60820100
C	9.86411800	2.63414700	-0.67077800
C	10.74168100	1.91619400	-1.49846700
H	10.36521100	1.51899400	-2.43307400
C	12.01874400	1.57883700	-1.06851600
C	12.47455300	2.10794400	0.14285600
C	11.62411500	2.84369300	0.95789600
H	11.96816900	3.17563600	1.93092300
C	12.80310700	0.53090600	-1.80067200
H	13.82501300	0.45931500	-1.42667800
H	12.84895000	0.72725200	-2.87378400
C	8.02917300	0.97459600	1.95223900
H	7.07403100	0.84508500	2.46255900
H	7.80553500	1.39141000	0.97187600
C	8.72537200	-0.38765900	1.80980300
C	7.77018900	-1.32872600	1.04179000
H	7.54222800	-0.90061100	0.06038100
H	6.82238000	-1.41714000	1.58513100
C	8.40893800	-2.71770000	0.87206500
H	7.71839800	-3.36423100	0.32195600
C	9.72164700	-2.59178200	0.08052700
H	10.17497200	-3.58141600	-0.05374000
H	9.51891000	-2.18969000	-0.91594700
C	10.70201200	-1.66243600	0.82271800
C	10.03372700	-0.29042900	1.00794600
H	10.72038600	0.38645700	1.51871400
H	9.82201900	0.13260300	0.02710900
C	9.02638600	-1.00863200	3.18679300
H	8.09798000	-1.09235700	3.76406200
H	9.69715100	-0.34886200	3.74625800
C	9.66859600	-2.39424000	3.01595900
H	9.88749600	-2.81814200	4.00136300
C	8.70190900	-3.31823200	2.25653100
H	7.77163900	-3.43343400	2.82381600
H	9.13997400	-4.31667700	2.14852200
C	10.97886600	-2.25802900	2.22174900
H	11.45724000	-3.23891300	2.11859500
H	11.67877400	-1.61205800	2.76487900

C	12.05113100	-1.54377100	0.08909400
H	12.57849000	-2.49823200	0.15152800
H	12.67514200	-0.79173000	0.57735500
H	8.38772200	3.89802100	1.18040400
H	13.48333400	1.88986800	0.47619200
C	8.51134400	2.74504300	-1.07458800
S	-12.88690600	-1.02855300	-1.76470700
S	-8.87627300	0.60904700	3.10359800
C	-12.50764500	-2.65520000	-0.96156800
H	-12.28870300	-3.30484200	-1.81113800
C	-11.35309100	-2.56352700	-0.01011700
C	-11.56063600	-2.57150500	1.37250800
C	-10.54441400	-2.19812600	2.24236600
H	-10.74618300	-2.12711500	3.30520600
C	-9.29715700	-1.79908200	1.76735000
C	-9.03866900	-1.92797800	0.38228900
C	-10.07080800	-2.31403400	-0.48607400
H	-9.87885900	-2.32305700	-1.55211500
C	-8.32738100	-1.11053900	2.67705200
H	-7.32243200	-1.07563400	2.26116500
H	-8.27814300	-1.59957300	3.65131800
C	-13.39224900	0.04000000	-0.34210900
H	-14.39082800	0.40550300	-0.59172800
H	-13.48798100	-0.60422000	0.53459100
C	-12.47983300	1.23637800	-0.01685300
C	-13.18791900	2.10196400	1.04938700
H	-13.38649800	1.49673500	1.94161900
H	-14.15697000	2.44325800	0.66739500
C	-12.31470700	3.31311600	1.42165800
H	-12.83340600	3.91196800	2.17704100
C	-10.97289200	2.82570300	1.99118900
H	-10.34973100	3.68362000	2.27070300
H	-11.14057800	2.23799100	2.89852400
C	-10.23460100	1.96253600	0.95009200
C	-11.13144000	0.77749800	0.55973500
H	-10.61810800	0.16521200	-0.18136300
H	-11.30473000	0.15571200	1.43816700
C	-12.21785300	2.10867600	-1.25983900
H	-13.17010600	2.45602000	-1.67840600
H	-11.72540700	1.50741600	-2.02959700
C	-11.33706100	3.31240900	-0.88595500
H	-11.14726200	3.91313800	-1.78110200
C	-12.05933600	4.16625600	0.16864900
H	-13.00696900	4.53902600	-0.23535100
H	-11.45266100	5.04072000	0.42808000

C	-9.99622000	2.81629600	-0.31542000
H	-9.35504200	3.67130300	-0.07196600
H	-9.46716600	2.21872200	-1.06652500
C	-8.86807600	1.48984300	1.47643500
H	-8.21860000	2.35656600	1.61906100
H	-8.38381700	0.83828800	0.74824400
H	-12.54223500	-2.81401100	1.76474100
H	-13.40530000	-3.03171000	-0.47007200
C	-0.25772100	-2.06783100	-0.11421100
C	0.66632800	-2.61211600	-1.07726800
H	0.31080800	-3.36933400	-1.76393300
C	1.95563700	-2.20051100	-1.12232900
H	2.64009700	-2.62549800	-1.84502700
C	0.23460100	-1.05680200	0.78666000
H	-0.44772900	-0.63717800	1.51458500
C	1.52150000	-0.64275700	0.73897800
H	1.87799500	0.11114200	1.42890100
C	2.45059800	-1.19931000	-0.21172200
C	3.77640200	-0.79073400	-0.23253600
C	-1.57125500	-2.50795000	-0.04181400
C	4.25826700	0.18895000	0.67498100
C	4.71962100	-1.34473000	-1.13776200
C	-2.47483700	-1.99563900	0.92696400
C	-2.06839700	-3.50688400	-0.92073000
N	4.62535400	0.98937500	1.42418500
N	5.48276500	-1.80460700	-1.87476700
N	-2.45354200	-4.31629700	-1.65100700
N	-3.19783800	-1.56686300	1.72089300

AFA-TCNQ-3

Electronic Energy (EE): -4631.881996 Hartree

EE + Thermal Free Energy Correction: -4630.836742 Hartree

E (Thermal): 769.283 kcal/mol

Entropy (S):382.253 cal/mol-kelvin

Imaginary frequencies: 0

0	1		

H	0.23691100	-1.91217200	-3.46312900
C	-0.10591100	-1.73357400	-2.45114700
C	-0.99679600	-1.23738600	0.18504500
C	0.79620700	-1.68327100	-1.38597200
C	-1.45326000	-1.53323400	-2.20136100
C	-1.91257800	-1.28548500	-0.88895300
C	0.34313200	-1.43354300	-0.07223800
H	-2.16913500	-1.53935900	-3.01224600
H	-1.35617500	-1.01830300	1.18196200
C	2.24691700	-1.83372300	-1.35762200
C	4.96359200	-2.01894600	-0.75131100
C	3.15897100	-2.08803900	-2.38344300
C	2.69285400	-1.67190700	-0.02881400
C	4.03576600	-1.76208400	0.27982100
C	4.50766000	-2.17960900	-2.07792400
H	2.82451900	-2.21219200	-3.40666700
H	4.39145800	-1.63806000	1.29545400
H	5.23365600	-2.37539300	-2.85660200
C	1.51543600	-1.40367300	0.88256800
H	1.59876100	-0.43350900	1.38346400
H	1.42467900	-2.16276900	1.66648900
C	6.34542500	-2.11283600	-0.46377100
C	-3.27788700	-1.02599600	-0.66040400
C	7.53559400	-2.19234600	-0.22883800
C	8.86389000	-2.27381200	0.01915800
C	-4.44567600	-0.73457900	-0.47146000
C	-5.71132300	-0.31140300	-0.28969200
C	-6.82828200	0.15529400	-0.15685700
S	12.30740000	-0.70560900	-2.75849300
S	13.46395400	0.12925000	3.60177900
C	11.91072200	-2.37039900	-2.04280800
H	12.45012000	-3.05342300	-2.70124300
C	12.36936700	-2.50033100	-0.62370600
C	11.45818700	-2.38683800	0.45277400
C	11.94525400	-2.22297500	1.75858000
H	11.23768900	-2.04802500	2.55994000
C	13.31071300	-2.16158300	2.01428400
C	14.19830100	-2.41612900	0.96467200
C	13.72858600	-2.58482600	-0.33124000
H	14.43318300	-2.69427500	-1.14799900
C	13.81070600	-1.67234100	3.33991200
H	14.87940800	-1.85397600	3.45839000
H	13.29176600	-2.14685200	4.17498200
C	11.32243400	0.46726900	-1.71928300
H	10.70671800	1.03125300	-2.42357900

H	10.64767200	-0.12419600	-1.09958000
C	12.12799400	1.44948700	-0.85086500
C	11.13899900	2.44241900	-0.20025000
H	10.40718800	1.89034300	0.40030900
H	10.58185400	2.97647200	-0.97849200
C	11.89658800	3.44789100	0.68563100
H	11.17990900	4.14089700	1.13753900
C	12.64638800	2.69590800	1.79753900
H	13.17834600	3.40813600	2.43976200
H	11.93639400	2.15593600	2.43057600
C	13.64655100	1.69806400	1.18241500
C	12.87965700	0.72326900	0.27533800
H	13.57716400	0.00929500	-0.16306600
H	12.16839100	0.16089600	0.87975500
C	13.14099200	2.24800900	-1.69343900
H	12.61707200	2.78266300	-2.49474600
H	13.84327500	1.55757500	-2.16983700
C	13.90148900	3.24537700	-0.80493500
H	14.62668000	3.79432900	-1.41406800
C	12.90493900	4.22975000	-0.17168100
H	12.38215800	4.78931800	-0.95499500
H	13.43643900	4.96074000	0.44748100
C	14.64696900	2.48357200	0.30504400
H	15.21298400	3.18751300	0.92599700
H	15.37008900	1.78960900	-0.13923100
C	14.44511000	0.95160000	2.26650800
H	15.10259900	1.65666100	2.77999800
H	15.07686100	0.18916000	1.80566300
H	10.84464300	-2.56109300	-2.14807200
H	15.26586000	-2.42134300	1.15546700
C	10.06045300	-2.34326400	0.22258200
S	-10.77887700	0.16214400	3.54188700
S	-9.68872400	-1.24153200	-2.73444600
C	-10.40703900	1.81510300	2.79534500
H	-9.70865200	2.26785300	3.50068700
C	-9.81612900	1.68260700	1.42574100
C	-10.56917900	1.99069500	0.28734700
C	-10.13747700	1.60743500	-0.97661500
H	-10.77015400	1.79017900	-1.83682600
C	-8.94174500	0.91906500	-1.15020200
C	-8.10256800	0.74129500	-0.02112300
C	-8.55553500	1.12622200	1.25223800
H	-7.93813600	0.90422000	2.11374700
C	-8.63270400	0.25672200	-2.45501700
H	-7.58313600	-0.01848100	-2.53811400

H	-8.89155000	0.89841200	-3.29893400
C	-12.07172900	-0.52977200	2.41289400
H	-12.92515700	-0.75907200	3.05465600
H	-12.37833100	0.27008800	1.73541400
C	-11.69076300	-1.78571800	1.60820100
C	-12.96263500	-2.30487000	0.90050300
H	-13.36695900	-1.52123600	0.24920800
H	-13.73340800	-2.53662100	1.64434000
C	-12.63947700	-3.56057200	0.07168600
H	-13.55208100	-3.91133200	-0.42030400
C	-11.58610000	-3.21908900	-0.99443700
H	-11.36007700	-4.10691500	-1.59686100
H	-11.97460900	-2.45592900	-1.67524100
C	-10.29884100	-2.70266100	-0.32329700
C	-10.64172700	-1.47193000	0.53025400
H	-9.73638300	-1.10575800	1.01372900
H	-11.01483100	-0.67935900	-0.11860800
C	-11.14662000	-2.90264500	2.51968100
H	-11.88810600	-3.14960900	3.28874700
H	-10.25008800	-2.54749700	3.03622200
C	-10.81638500	-4.15202100	1.68644800
H	-10.41739200	-4.93021400	2.34458300
C	-12.09330600	-4.65959700	0.99735000
H	-12.84332600	-4.92891500	1.74889400
H	-11.87719600	-5.56412600	0.41856500
C	-9.76200800	-3.80175300	0.62117200
H	-9.50434000	-4.69620200	0.04267700
H	-8.84210600	-3.45338700	1.10466200
C	-9.20745900	-2.38627500	-1.36157600
H	-8.89874800	-3.31145200	-1.85320300
H	-8.32558300	-1.96936000	-0.87312400
H	-11.52656900	2.48681900	0.39880100
H	-11.30937200	2.42561500	2.78571400
C	-7.35091700	3.87355700	-0.30135300
C	-6.91209200	3.48120800	-1.61792800
H	-7.57819700	3.64400700	-2.45515500
C	-5.69387800	2.92573600	-1.81290900
H	-5.37875900	2.63786200	-2.80777700
C	-6.46055800	3.61947100	0.80429000
H	-6.78466000	3.88624900	1.80172500
C	-5.23877000	3.07387700	0.60752700
H	-4.57822100	2.89877600	1.44709000
C	-4.78397000	2.71890100	-0.71374300
C	-3.51165000	2.20794600	-0.92014900
C	-8.58240000	4.47858700	-0.10439900

C	-2.59555800	2.02746100	0.15092600
C	-3.05594500	1.83653100	-2.21357800
C	-9.03374100	4.83073300	1.19551600
C	-9.45501100	4.76086200	-1.18877600
N	-1.85278300	1.88051100	1.02478600
N	-2.69475700	1.52704000	-3.26742200
N	-10.15415100	4.97960700	-2.08346700
N	-9.38749600	5.09515100	2.26440500

AFA-TCNQ-4

Electronic Energy (EE): -4631.881957 Hartree

EE + Thermal Free Energy Correction: -4630.836613 Hartree

E (Thermal): 769.287 kcal/mol

Entropy (S): 382.079 cal/mol-kelvin

Imaginary frequencies: 0

0	1		
H	-0.25647300	-1.27950000	-3.79704000
C	0.05753600	-1.03388400	-2.78975500
C	0.88037700	-0.42075900	-0.15605400
C	-0.86897700	-0.95912400	-1.74720300
C	1.39591600	-0.80395500	-2.51789200
C	1.82068600	-0.49322800	-1.20731400
C	-0.44920000	-0.65578100	-0.43368600
H	2.13707200	-0.88083000	-3.30205900
H	1.22082800	-0.21089600	0.84945800
C	-2.31350800	-1.16249600	-1.73921300
C	-5.03090400	-1.43982800	-1.17246300
C	-3.19787900	-1.47298100	-2.77373900
C	-2.78720400	-0.99277300	-0.42106000
C	-4.13053100	-1.12947100	-0.13179000
C	-4.54719800	-1.60901000	-2.48806600
H	-2.84172700	-1.60631900	-3.78845200
H	-4.50709500	-1.00172200	0.87584000
H	-5.25218400	-1.84791700	-3.27396200
C	-1.63584600	-0.66407300	0.50370800
H	-1.51748800	-1.41488200	1.29201500
H	-1.77332700	0.30248700	0.99944000

C	-6.41334700	-1.57725900	-0.90549600
C	3.19069900	-0.30780100	-0.93853300
C	-7.60408600	-1.69116400	-0.68849200
C	-8.93222400	-1.80827100	-0.45373500
C	4.37964400	-0.20835400	-0.69212100
C	5.69648000	-0.20214000	-0.40954600
C	6.88385800	-0.28809000	-0.15335800
S	-11.40083700	-0.26970900	3.27749200
S	-14.42021700	0.45712700	-2.45548900
C	-11.19917000	-1.93421700	2.48485900
H	-11.49495500	-2.62114300	3.27964500
C	-12.06406700	-2.09168100	1.27309300
C	-11.52481500	-1.98036200	-0.02962600
C	-12.38814700	-1.84451600	-1.12772400
H	-11.95993700	-1.67046000	-2.10735400
C	-13.76744400	-1.80593900	-0.95808600
C	-14.29027700	-2.05736100	0.31389200
C	-13.44641400	-2.20069100	1.40751900
H	-13.86803300	-2.30876200	2.40053700
C	-14.65199800	-1.34198000	-2.07584100
H	-15.70428200	-1.53168800	-1.86166200
H	-14.40610400	-1.82745900	-3.02223900
C	-10.79520000	0.90638800	1.98367200
H	-10.00996500	1.49098000	2.46839900
H	-10.32270700	0.31855400	1.19587300
C	-11.84122400	1.86317100	1.38562100
C	-11.10671700	2.86197000	0.46347800
H	-10.57489600	2.31278900	-0.32172300
H	-10.35446100	3.41466200	1.03794200
C	-12.10977400	3.84422400	-0.16720800
H	-11.57100900	4.54149700	-0.81660200
C	-13.14114400	3.06697700	-1.00171000
H	-13.85154700	3.76303500	-1.46376600
H	-12.64049100	2.52903700	-1.81197400
C	-13.89716100	2.06314600	-0.10958400
C	-12.87905900	1.11121600	0.53776400
H	-13.40308900	0.39259000	1.16812300
H	-12.36799600	0.55125800	-0.24508600
C	-12.57436800	2.65782500	2.48343600
H	-11.84675100	3.21016700	3.09006500
H	-13.09238100	1.96348900	3.15160600
C	-13.58054800	3.63277700	1.85136900
H	-14.10275500	4.17921900	2.64320400
C	-12.83302500	4.62293200	0.94352100
H	-12.11173100	5.19994000	1.53244600

H	-13.53640300	5.33827900	0.50337600
C	-14.60693500	2.84627800	1.01766900
H	-15.34323900	3.53386900	0.58577400
H	-15.15454300	2.14814600	1.66157400
C	-14.97016200	1.29287200	-0.90009000
H	-15.75907400	1.98451100	-1.20401400
H	-15.42774100	0.53264400	-0.26326900
H	-10.14707500	-2.09990000	2.26115000
H	-15.36512500	-2.08008200	0.45645600
C	-10.12561500	-1.90736900	-0.24415300
S	10.40585400	1.99387800	3.25150200
S	9.46183900	0.88463500	-3.10760900
C	10.54305500	0.15253900	3.11441000
H	9.94015700	-0.21545900	3.94592400
C	10.04068100	-0.34288600	1.79347200
C	10.92981300	-0.79582400	0.81254000
C	10.50746300	-0.98629700	-0.49741900
H	11.22742500	-1.26658500	-1.25684500
C	9.18980000	-0.74471900	-0.87075300
C	8.25280800	-0.43446900	0.14706000
C	8.69516400	-0.23759100	1.46631600
H	7.97979800	0.08425400	2.21284000
C	8.81645900	-0.66324100	-2.31662000
H	7.74060600	-0.72661300	-2.46786200
H	9.29622600	-1.45293300	-2.89728600
C	11.54682500	2.60376800	1.92803300
H	12.25766300	3.25819800	2.43715000
H	12.10557900	1.74328000	1.55371200
C	10.90581600	3.36714600	0.75457000
C	12.04233300	3.95864600	-0.10923900
H	12.69223600	3.15084900	-0.46534000
H	12.66162400	4.62770400	0.49895900
C	11.45846500	4.72874900	-1.30678600
H	12.27830300	5.13889300	-1.90476900
C	10.62252100	3.77546500	-2.17606700
H	10.21407200	4.31495500	-3.03885000
H	11.25393600	2.97155600	-2.56597500
C	9.47303700	3.17069600	-1.34728000
C	10.06684300	2.43654100	-0.13512900
H	9.25838300	2.01311600	0.46009500
H	10.68821300	1.61193200	-0.48519000
C	10.01168600	4.52178400	1.24600200
H	10.59692400	5.20442600	1.87348500
H	9.20665500	4.11993300	1.86814600
C	9.42319000	5.28322500	0.04670400

H	8.77948200	6.08985800	0.41120600
C	10.56620100	5.87159800	-0.79614900
H	11.15529200	6.57110300	-0.19321100
H	10.16045900	6.43698400	-1.64206300
C	8.58860000	4.32246300	-0.81915700
H	8.14571200	4.86739800	-1.66058300
H	7.76160900	3.90960100	-0.23011200
C	8.58995100	2.24095000	-2.19847500
H	8.08763300	2.82856200	-2.97006500
H	7.81381700	1.78394800	-1.58308600
H	11.97260800	-0.94808700	1.06664800
H	11.57337200	-0.15387100	3.29217300
C	8.34602000	-3.56035900	0.95061700
C	7.91224900	-3.75499700	-0.41101000
H	8.65344600	-4.00193100	-1.15989400
C	6.60866200	-3.63753600	-0.75373800
H	6.29895600	-3.78732300	-1.78018600
C	7.34652200	-3.20260600	1.92677900
H	7.65909200	-3.03076100	2.94847000
C	6.04213800	-3.09603300	1.58583300
H	5.30292500	-2.83610700	2.33272900
C	5.60310800	-3.32805200	0.23225500
C	4.25986100	-3.26609000	-0.10559400
C	9.67534500	-3.71610300	1.31124700
C	3.25745500	-2.98954500	0.86281500
C	3.81341800	-3.47227600	-1.43861200
C	10.11195300	-3.48612900	2.64316000
C	10.66339600	-4.09663500	0.36473600
N	2.44440500	-2.76266400	1.65300500
N	3.45732700	-3.63100600	-2.52710800
N	11.45457100	-4.40341700	-0.42079200
N	10.44756400	-3.27887400	3.73035500

AFA-TCNQ-5

Electronic Energy (EE): -4631.881643 Hartree

EE + Thermal Free Energy Correction: -4630.836785 Hartree

E (Thermal): 769.306 kcal/mol

Entropy (S): 383.165 cal/mol-kelvin

Imaginary frequencies: 0

0	1		
H	1.48355900	-2.56292300	-2.99861300
C	1.73582900	-1.94111700	-2.14811900
C	2.39361400	-0.32773600	0.07783700
C	0.73925100	-1.28646000	-1.41801400
C	3.05631600	-1.79667400	-1.76187200
C	3.40312000	-1.00424200	-0.64305000
C	1.07879100	-0.47347100	-0.31073100
H	3.84354500	-2.31339000	-2.29425700
H	2.66963800	0.27161700	0.93630400
C	-0.70428500	-1.30549800	-1.55951900
C	-3.47382700	-1.10906200	-1.30645700
C	-1.52297800	-1.98112300	-2.46949700
C	-1.27289300	-0.50785800	-0.53855400
C	-2.64147500	-0.40600200	-0.40689600
C	-2.89699800	-1.87744600	-2.34336600
H	-1.09423100	-2.58906900	-3.25703300
H	-3.09465100	0.17743100	0.38477000
H	-3.55206600	-2.41337900	-3.01704100
C	-0.17536900	0.12314500	0.29028400
H	-0.27511700	-0.10020900	1.35571300
H	-0.18201800	1.21396800	0.19116900
C	-4.87517700	-1.07680300	-1.14120200
C	4.75149800	-0.91128900	-0.23767700
C	-6.07169800	-1.10019200	-0.93133300
C	-7.39628500	-1.13620000	-0.66251400
C	5.90695800	-0.87039200	0.13698200
C	7.19331100	-0.82929200	0.55113100
C	8.35180600	-0.80478400	0.91864900
S	-9.53462100	0.91283600	2.98867300
S	-13.07316800	0.93195800	-2.48464500
C	-9.39663700	-0.84102400	2.40144600
H	-9.61887600	-1.41789300	3.30068100
C	-10.36924000	-1.14201200	1.30340500
C	-9.95414200	-1.20358300	-0.04772500
C	-10.91321100	-1.20141000	-1.07170700
H	-10.57691100	-1.16041700	-2.10058000
C	-12.27120500	-1.12913700	-0.78269500
C	-12.67338900	-1.21253700	0.55369500
C	-11.73308300	-1.22231100	1.57567300
H	-12.06200300	-1.20075500	2.60849500
C	-13.25722000	-0.80455600	-1.86396100
H	-14.28419400	-0.96107600	-1.53236700

H	-13.09542100	-1.40571700	-2.76072600
C	-9.05554400	1.91033400	1.50583100
H	-8.22991900	2.54442300	1.83693500
H	-8.65714500	1.22350800	0.75824200
C	-10.15669400	2.79258000	0.89192900
C	-9.51774700	3.66322800	-0.21303000
H	-9.05899500	3.01669900	-0.96979200
H	-8.71803800	4.27900700	0.21441000
C	-10.58140000	4.56467700	-0.86508600
H	-10.11009900	5.17182500	-1.64448300
C	-11.68215100	3.69522200	-1.49509200
H	-12.43668600	4.33229300	-1.97203900
H	-11.25684600	3.05688500	-2.27496600
C	-12.34527600	2.81596700	-0.41725100
C	-11.26547900	1.94693600	0.24620000
H	-11.72399700	1.31652600	1.00819700
H	-10.82720500	1.28996300	-0.50482500
C	-10.78818800	3.72311800	1.94523100
H	-10.01008000	4.34293400	2.40661700
H	-11.23666700	3.12102400	2.74094500
C	-11.85510500	4.61691800	1.29308500
H	-12.30392200	5.26154400	2.05552000
C	-11.20186200	5.48078200	0.20207000
H	-10.43196400	6.12289300	0.64367400
H	-11.94757300	6.13942400	-0.25651100
C	-12.95076900	3.73829400	0.66453300
H	-13.72838000	4.37077500	0.22095900
H	-13.43133400	3.12957400	1.43935900
C	-13.48227500	1.95822900	-1.00130300
H	-14.30101900	2.60999300	-1.31427300
H	-13.87285900	1.28583000	-0.23440400
H	-8.36776700	-1.04403800	2.11097700
H	-13.73061300	-1.20910900	0.79547300
C	-8.57870800	-1.17932200	-0.38521500
S	11.59128500	3.05081000	2.84456200
S	11.09759300	-1.18462100	-2.08437700
C	11.77194200	1.40526900	3.67757200
H	11.11336100	1.49351500	4.54371000
C	11.37701100	0.26900200	2.78336400
C	12.34488300	-0.55510800	2.20109800
C	12.01093700	-1.41054600	1.15933000
H	12.78799300	-1.97906300	0.66100100
C	10.71140600	-1.46902500	0.66124400
C	9.70243100	-0.74670800	1.34069000
C	10.05133200	0.10980000	2.39637600

H	9.27913000	0.72031200	2.84832200
C	10.43364700	-2.14659200	-0.64437300
H	9.37172000	-2.33090200	-0.79402800
H	10.96204000	-3.09826900	-0.72139700
C	12.80542600	2.95869100	1.45224100
H	13.46990200	3.81499500	1.58752900
H	13.40163400	2.05486100	1.59524800
C	12.22611600	2.98551500	0.02625700
C	13.40348800	3.11412900	-0.96616200
H	14.09211500	2.27196400	-0.83061000
H	13.96752700	4.03040700	-0.75704000
C	12.88292300	3.13738000	-2.41405800
H	13.73108100	3.22863000	-3.10001500
C	12.12606500	1.83283800	-2.71053400
H	11.76280700	1.83621300	-3.74516900
H	12.79847700	0.97620800	-2.60619600
C	10.93832900	1.67433200	-1.74178500
C	11.46692200	1.68975700	-0.29904200
H	10.63134400	1.58474400	0.39267200
H	12.12706400	0.83503400	-0.15073100
C	11.27786600	4.18220100	-0.18161700
H	11.80664400	5.11829500	0.03456300
H	10.44286000	4.11157500	0.52140900
C	10.75214400	4.19618900	-1.62653400
H	10.06888600	5.04176600	-1.75435200
C	11.93456600	4.33293500	-2.59928800
H	12.46754000	5.27161100	-2.41224100
H	11.57269800	4.36750700	-3.63266800
C	9.99669800	2.88676900	-1.91648500
H	9.59791100	2.90423300	-2.93726500
H	9.14239700	2.78829800	-1.23708200
C	10.13560200	0.39541600	-2.03784300
H	9.67217400	0.48187400	-3.02325200
H	9.33171900	0.27380700	-1.31088000
H	13.37675100	-0.48096700	2.52651200
H	12.79463000	1.29559300	4.03976600
C	1.24286700	-3.67798500	0.79204600
C	0.61545200	-4.42067600	-0.27200300
H	1.24510400	-4.96768200	-0.96162000
C	-0.73111100	-4.44546600	-0.40972000
H	-1.18769700	-5.01235700	-1.21058500
C	0.38590500	-2.95562100	1.69822400
H	0.84227600	-2.39399800	2.50307100
C	-0.95956900	-2.98155400	1.56146700
H	-1.58980600	-2.44085100	2.25576100

C	-1.58801600	-3.73219000	0.50388400
C	-2.96896700	-3.77346800	0.38214900
C	2.62057900	-3.66490800	0.95178300
C	-3.81657700	-3.09030800	1.29455200
C	-3.60118200	-4.51483700	-0.65145200
C	3.23452900	-2.94184200	2.00885400
C	3.47806200	-4.38414600	0.07731900
N	-4.49173600	-2.52568300	2.04436900
N	-4.09452900	-5.11973100	-1.50437100
N	4.15895300	-4.97300500	-0.64825700
N	3.70885900	-2.34102100	2.87534400

AFA-TCNQ-6

Electronic Energy (EE): -4631.875239 Hartree

EE + Thermal Free Energy Correction: -4630.833007 Hartree

E (Thermal): 769.312 kcal/mol

Entropy (S): 388.712 cal/mol-kelvin

Imaginary frequencies: 0

0	1		
H	1.28219800	-5.13168700	0.96298500
C	1.66479100	-4.45045100	0.21220500
C	2.67548000	-2.66948100	-1.72799800
C	0.79995700	-3.73947300	-0.62192000
C	3.03323900	-4.25551600	0.09076600
C	3.55414100	-3.35016200	-0.85663400
C	1.31477700	-2.86813500	-1.60510800
H	3.71830500	-4.77906200	0.74535500
H	3.08458400	-1.98175100	-2.45823500
C	-0.65663000	-3.65339000	-0.63802100
C	-3.34166500	-2.96150700	-0.95294600
C	-1.61361200	-4.23769100	0.19091800
C	-1.04378100	-2.73584700	-1.63820200
C	-2.36941300	-2.39025700	-1.80352500
C	-2.94682500	-3.88670500	0.03645300
H	-1.32820100	-4.94355900	0.96160300

H	-2.67746400	-1.67906400	-2.56044200
H	-3.69962300	-4.30682100	0.69009400
C	0.17808300	-2.22912400	-2.37268700
H	0.23578500	-1.13731400	-2.38840600
H	0.17716900	-2.55508600	-3.41875700
C	-4.70328600	-2.60104800	-1.08384100
C	4.93936600	-3.06955300	-0.89617900
C	-5.87321900	-2.28863700	-1.18894300
C	-7.17695900	-1.94102600	-1.29721500
C	6.11296400	-2.75347900	-0.90346700
C	7.40733700	-2.35938500	-0.90043800
C	8.55257300	-1.95273800	-0.89253500
S	-10.25426200	-1.28818200	2.21503400
S	-10.95003100	2.89160300	-2.73684300
C	-10.22946300	-2.39774300	0.72920200
H	-10.93422800	-3.18539300	1.00103300
C	-10.65734400	-1.68463300	-0.51567400
C	-9.70971300	-1.24403500	-1.46912700
C	-10.09898100	-0.33654500	-2.46623400
H	-9.34314200	0.05986500	-3.13329000
C	-11.40154700	0.14546100	-2.53070000
C	-12.35935500	-0.40475100	-1.67370700
C	-11.98734300	-1.30697300	-0.68568200
H	-12.72536900	-1.65768100	0.02691000
C	-11.72508400	1.33656500	-3.38117900
H	-12.80154700	1.48329300	-3.47519400
H	-11.30605100	1.24974800	-4.38549400
C	-8.98460200	0.00007200	1.82347700
H	-8.28057700	-0.02413700	2.65832500
H	-8.44425800	-0.32532300	0.93374900
C	-9.50592300	1.43620600	1.64057900
C	-8.28971600	2.37788600	1.49495300
H	-7.68384900	2.06384600	0.63734300
H	-7.65383600	2.30457200	2.38470900
C	-8.75912700	3.83149000	1.30434300
H	-7.88461200	4.48183600	1.20204200
C	-9.62060700	3.93582700	0.03479500
H	-9.94792800	4.97205200	-0.11252100
H	-9.03198700	3.65377700	-0.84296900
C	-10.84735700	3.01055800	0.14869800
C	-10.36189300	1.56974000	0.37131300
H	-11.22179500	0.90413300	0.44792100
H	-9.77714600	1.25437700	-0.49263100
C	-10.33718600	1.89391800	2.85462800
H	-9.73434500	1.81609800	3.76729200

H	-11.19821800	1.23041000	2.97783000
C	-10.81079100	3.34279200	2.65833900
H	-11.41120700	3.64830400	3.52114500
C	-9.58986100	4.26663600	2.52254200
H	-8.98139700	4.21941700	3.43224800
H	-9.91313800	5.30662500	2.40389800
C	-11.67022100	3.43642000	1.38538500
H	-12.03392200	4.46212600	1.25457800
H	-12.55076800	2.79044800	1.48126700
C	-11.75762800	3.11671800	-1.08817100
H	-12.21025100	4.11034500	-1.11826100
H	-12.56850300	2.38852900	-1.01732100
H	-9.24073900	-2.84102500	0.62899200
H	-13.39090300	-0.07728400	-1.74291600
C	-8.34992100	-1.63337800	-1.38199000
S	11.26066000	0.72681000	2.85082500
S	9.02846700	1.32047400	-3.24133400
C	12.30777200	-0.46841100	1.89701900
H	12.30172700	-1.35943000	2.52788500
C	11.75634900	-0.74153600	0.52988700
C	12.36050700	-0.20192000	-0.60963600
C	11.69713200	-0.20482200	-1.82970900
H	12.14221600	0.29251800	-2.68407800
C	10.41241200	-0.72974700	-1.95275800
C	9.85502500	-1.39756900	-0.83773700
C	10.54197500	-1.40224200	0.38581400
H	10.05965300	-1.83904800	1.25176700
C	9.59482000	-0.44336300	-3.17415800
H	8.73035600	-1.09999900	-3.25174300
H	10.18890700	-0.54715300	-4.08369600
C	11.38116400	2.28463000	1.86029700
H	11.75684700	3.04106700	2.55294100
H	12.14663100	2.12927900	1.09686500
C	10.08383000	2.79404200	1.20623500
C	10.36159500	4.20064700	0.62987200
H	11.18108100	4.14507000	-0.09626100
H	10.68518500	4.87370500	1.43235100
C	9.09865900	4.76058600	-0.04777000
H	9.31661500	5.75587700	-0.44818800
C	8.67420200	3.83032400	-1.19505600
H	7.78158000	4.22867300	-1.69164300
H	9.46653400	3.77576800	-1.94770400
C	8.38094000	2.41735500	-0.65530700
C	9.63703100	1.88362100	0.05154100
H	9.43260100	0.88812500	0.44444000

H	10.44605500	1.79196600	-0.67393700
C	8.93356600	2.90223700	2.22593400
H	9.22963300	3.55863700	3.05322300
H	8.73090600	1.91511900	2.65156900
C	7.67030400	3.45185700	1.54245800
H	6.85930700	3.50906100	2.27593300
C	7.96159200	4.85359400	0.98256600
H	8.24188200	5.53241800	1.79566700
H	7.06301600	5.26687000	0.51163900
C	7.25111600	2.51664700	0.39437400
H	6.33529100	2.88518900	-0.07929300
H	7.02786400	1.51718600	0.78450300
C	7.90972100	1.47459200	-1.77492400
H	6.95474200	1.82463700	-2.16759100
H	7.73826800	0.47270100	-1.38258400
H	13.33005200	0.27675700	-0.52554500
H	13.33088400	-0.09391100	1.85064200
C	0.46975700	-1.85200800	2.11105400
C	-0.44877300	-1.03803200	1.35101600
H	-1.51190900	-1.16180600	1.51130400
C	0.00463000	-0.16882000	0.42309500
H	-0.69402000	0.41621300	-0.16076100
C	1.88471700	-1.67564700	1.88500300
H	2.58131200	-2.26791400	2.46239400
C	2.33867200	-0.80538200	0.95823200
H	3.40100700	-0.69733000	0.78368800
C	1.41851200	-0.03011800	0.16163200
C	1.87450600	0.79720100	-0.84888300
C	0.00521100	-2.79466100	3.00988400
C	3.25712000	0.90537000	-1.15890400
C	0.97705000	1.53374600	-1.66906700
C	0.89603300	-3.67192300	3.68609600
C	-1.38055800	-2.97342000	3.26804200
N	4.38173100	0.97578700	-1.41687600
N	0.22985200	2.10751100	-2.33942800
N	-2.50876400	-3.12088300	3.47199600
N	1.63203300	-4.39796400	4.20355600

AFA-TCNQ-7

Electronic Energy (EE): -4631.874528 Hartree

EE + Thermal Free Energy Correction: -4630.832647 Hartree

E (Thermal): 769.306 kcal/mol

Entropy (S): 389.430 cal/mol-kelvin

Imaginary frequencies: 0

0	1		
H	-0.48460600	-0.52209700	-4.12762400
C	-0.11823700	-0.58572800	-3.10977400
C	0.84103900	-0.75094400	-0.45609200
C	-0.99571500	-0.79299200	-2.04410000
C	1.23844500	-0.46059700	-2.85066100
C	1.73108700	-0.54060500	-1.53166700
C	-0.50779600	-0.87421000	-0.72249400
H	1.93810500	-0.29871800	-3.66088100
H	1.23479800	-0.81903800	0.55071100
C	-2.44632800	-0.95858000	-2.01780100
C	-5.14526200	-1.32209700	-1.40625400
C	-3.38650000	-0.95833700	-3.04936500
C	-2.85580600	-1.14153300	-0.68020800
C	-4.18880200	-1.32200900	-0.36905200
C	-4.72633600	-1.13927400	-2.74145500
H	-3.08116500	-0.81863400	-4.07977100
H	-4.51562400	-1.46272900	0.65415800
H	-5.47282400	-1.14188200	-3.52543500
C	-1.65428400	-1.10128800	0.23823800
H	-1.53654000	-2.03509100	0.79788300
H	-1.73133900	-0.29570800	0.97586900
C	-6.51682300	-1.50152300	-1.10905300
C	3.11525800	-0.41677700	-1.26915200
C	-7.69479200	-1.65206700	-0.84883000
C	-9.00629700	-1.80956600	-0.55199900
C	4.29363400	-0.32357700	-0.98590700
C	5.58923800	-0.26226100	-0.60324900
C	6.73946100	-0.26623400	-0.20964100
S	-11.28906700	-0.04414300	3.16267200
S	-14.73949000	0.02375700	-2.36881300
C	-11.06641600	-1.76682300	2.51205400
H	-11.27703900	-2.38683600	3.38513400
C	-12.00283700	-2.07145400	1.38432900
C	-11.55951200	-2.05650600	0.04098300
C	-12.50151800	-2.05812200	-0.99922000
H	-12.15053300	-1.95682200	-2.01901300

C	-13.86638900	-2.06358300	-0.73373000
C	-14.28820500	-2.21994100	0.58987600
C	-13.36558900	-2.22646700	1.62779500
H	-13.71243700	-2.26101300	2.65447400
C	-14.84871300	-1.74290700	-1.81958900
H	-15.87219600	-1.96176500	-1.51338500
H	-14.64319700	-2.29876900	-2.73644300
C	-10.83083600	1.02855500	1.72606400
H	-10.04124100	1.68660200	2.09591000
H	-10.38841100	0.38858300	0.96195700
C	-11.95994400	1.88248900	1.12346500
C	-11.34123100	2.81572900	0.05881200
H	-10.84359200	2.21454200	-0.71053300
H	-10.57501400	3.44960500	0.51971100
C	-12.43181500	3.69315400	-0.58125300
H	-11.97459500	4.34529600	-1.33214300
C	-13.48489900	2.80012600	-1.25787500
H	-14.25835800	3.42070000	-1.72626200
H	-13.02091700	2.20669400	-2.05092800
C	-14.12666000	1.85783000	-0.22130900
C	-13.02131300	1.01306100	0.43147100
H	-13.46469100	0.33825600	1.16404500
H	-12.54382500	0.40109600	-0.33327700
C	-12.64685600	2.74951600	2.19605000
H	-11.90312900	3.38543100	2.69099000
H	-13.08215200	2.10276400	2.96366800
C	-13.74016400	3.61943600	1.55534200
H	-14.22792300	4.21870200	2.33078400
C	-13.10697700	4.54614400	0.50494100
H	-12.37187300	5.20443100	0.98065200
H	-13.87268100	5.18822700	0.05588400
C	-14.78773600	2.71714900	0.87975100
H	-15.58438100	3.33111000	0.44395800
H	-15.25410500	2.06342000	1.62608600
C	-15.21551400	0.97211800	-0.85365500
H	-16.05925600	1.59728600	-1.15426400
H	-15.58447800	0.25346100	-0.11869400
H	-10.02533100	-1.91149500	2.23035200
H	-15.34841100	-2.27565900	0.81123000
C	-10.18223400	-1.94310800	-0.27399300
S	9.76374200	2.79359600	3.05948400
S	9.90869300	0.01301900	-2.83397900
C	9.77783500	0.98346200	3.44934600
H	8.98922300	0.88084900	4.19750700
C	9.50408300	0.15162600	2.23322000

C	10.52162000	-0.56714800	1.59893900
C	10.32576800	-1.09570300	0.32855500
H	11.15021600	-1.57818900	-0.18352900
C	9.11495600	-0.93339400	-0.34496200
C	8.03792900	-0.33861400	0.35026000
C	8.25194800	0.19915500	1.62829100
H	7.44413500	0.73363500	2.11204400
C	9.01532300	-1.24264100	-1.80447700
H	7.98164800	-1.31818900	-2.13804100
H	9.52831900	-2.17226800	-2.05685400
C	11.17871100	2.98743500	1.88282100
H	11.83245000	3.73713000	2.33398400
H	11.72630500	2.04252400	1.86718000
C	10.82882200	3.41675200	0.44633100
C	12.14826400	3.70889900	-0.30264600
H	12.78095000	2.81365700	-0.29644800
H	12.70230000	4.50035500	0.21488900
C	11.85670300	4.13472800	-1.75255000
H	12.80252900	4.33672100	-2.26499400
C	11.11367000	3.00468100	-2.48338400
H	10.91438300	3.29634300	-3.52140400
H	11.73511800	2.10483200	-2.51340900
C	9.78687700	2.69031700	-1.76584100
C	10.08813000	2.30344700	-0.30993100
H	9.15264800	2.08838100	0.20532800
H	10.68995200	1.39453700	-0.29827200
C	9.96166700	4.69040700	0.42724500
H	10.48018000	5.50158700	0.95209400
H	9.02662000	4.50322800	0.96326500
C	9.66480400	5.10697100	-1.02292100
H	9.03635500	6.00301400	-1.02006100
C	10.98650300	5.40183500	-1.75044200
H	11.51395000	6.22250600	-1.25216700
H	10.78908600	5.72251500	-2.77906000
C	8.92171600	3.97046200	-1.74800500
H	8.68557400	4.27433800	-2.77416500
H	7.97028000	3.76360400	-1.24484900
C	8.99691800	1.58720900	-2.49241800
H	8.68160300	1.95490300	-3.47138400
H	8.09378600	1.33474300	-1.93465200
H	11.49149100	-0.66010500	2.07487500
H	10.72863500	0.71927200	3.91334900
C	8.19264700	-4.01809900	0.42631700
C	6.79818100	-3.88175500	0.07932500
H	6.45296600	-4.29194400	-0.86097700

C	5.94505100	-3.21234400	0.88394900
H	4.91214500	-3.07461600	0.59203300
C	8.62902000	-3.50439100	1.70270900
H	9.66075700	-3.64282900	1.99456700
C	7.77705300	-2.83320200	2.50627800
H	8.12062800	-2.42896400	3.44829400
C	6.40622000	-2.61064400	2.11310400
C	5.57521100	-1.79993200	2.86489200
C	9.09110400	-4.59791000	-0.45199400
C	6.07093200	-1.10226500	3.99975100
C	4.21989800	-1.56670600	2.50681900
C	10.47971900	-4.65263400	-0.15621600
C	8.68299500	-5.10614700	-1.71486800
N	6.51804800	-0.52179900	4.89417600
N	3.11561400	-1.38768500	2.21740400
N	8.33920700	-5.50876200	-2.74259500
N	11.60884900	-4.65768200	0.09251200

AFA-TCNQ-8

Electronic Energy (EE): -4621.874511 Hartree

EE + Thermal Free Energy Correction: -4630.832718 Hartree

E (Thermal): 769.293 kcal/mol

Entropy (S): 389.572 cal/mol-kelvin

Imaginary frequencies: 0

0	1		
H	0.39835700	-2.39787600	-3.42067100
C	0.04163800	-1.97005400	-2.49114100
C	-0.89279500	-0.85505700	-0.06776100
C	0.92499700	-1.68261000	-1.44916700
C	-1.30839800	-1.70004300	-2.32367200
C	-1.78868800	-1.14395300	-1.11998400
C	0.44938500	-1.12576800	-0.24293500
H	-2.01234600	-1.91430400	-3.11786000
H	-1.27500900	-0.42205300	0.84863500
C	2.37151000	-1.85467800	-1.34609700
C	5.06745900	-1.96035100	-0.63070600

C	3.30078800	-2.35920300	-2.25712400
C	2.79052700	-1.40357900	-0.07673100
C	4.12205700	-1.45197800	0.28493200
C	4.63914600	-2.41025900	-1.89787600
H	2.98819400	-2.70836600	-3.23428300
H	4.45603700	-1.10830400	1.25647800
H	5.37701500	-2.79779000	-2.58872200
C	1.59998000	-0.90975000	0.71534600
H	1.70372200	0.14388900	0.99482100
H	1.46575500	-1.47373200	1.64429200
C	6.43679100	-2.01824400	-0.27963200
C	-3.16482100	-0.86404600	-0.95150400
C	7.61181400	-2.06529700	0.02871300
C	8.92032800	-2.11313800	0.37291600
C	-4.33376800	-0.59680400	-0.75237600
C	-5.60994200	-0.24669400	-0.47346100
C	-6.72900600	0.13206500	-0.18672100
S	12.69495700	-1.75414300	-2.45301700
S	13.37833300	1.20945800	3.31441600
C	12.11690000	-3.04306600	-1.25110300
H	12.65202200	-3.93722000	-1.57543800
C	12.45058300	-2.68665400	0.16417400
C	11.46672400	-2.17030400	1.03956800
C	11.85874100	-1.57946800	2.25066800
H	11.10336800	-1.10911600	2.86839700
C	13.20010000	-1.48419400	2.60387100
C	14.14879000	-2.12279400	1.79974500
C	13.77294100	-2.71546800	0.60135000
H	14.53098800	-3.13025400	-0.05353100
C	13.62391400	-0.58152600	3.72303600
H	14.66522900	-0.74718700	4.00124800
H	13.00923100	-0.71729800	4.61497300
C	11.71633000	-0.25360400	-1.98929100
H	11.20340400	0.05205300	-2.90402800
H	10.95146300	-0.56355600	-1.27631000
C	12.51568100	0.93845100	-1.43484700
C	11.54954800	2.13358700	-1.27513900
H	10.73099900	1.85564600	-0.60154600
H	11.10100600	2.38142500	-2.24401100
C	12.29943800	3.35622400	-0.71686000
H	11.59876200	4.19037700	-0.60992700
C	12.89611000	3.01478200	0.65862900
H	13.42147400	3.88659800	1.06684300
H	12.09793100	2.75875200	1.36139700
C	13.87041100	1.82748900	0.53432700

C	13.11472500	0.62614800	-0.05464000
H	13.79346300	-0.22238700	-0.14251000
H	12.31587800	0.33927300	0.62879100
C	13.65187200	1.35139400	-2.39041100
H	13.23807800	1.58962200	-3.37758500
H	14.34064300	0.51166700	-2.52211300
C	14.40356300	2.56799700	-1.82723300
H	15.21544500	2.84031600	-2.50917500
C	13.43044300	3.74893900	-1.68133000
H	13.01733200	4.01713400	-2.65981900
H	13.95876100	4.63020800	-1.30125500
C	14.99534400	2.21714900	-0.45090400
H	15.55552000	3.07257100	-0.05598000
H	15.70215000	1.38494900	-0.54956000
C	14.52095100	1.47955600	1.88540100
H	15.17989100	2.29611500	2.18897600
H	15.13543000	0.58254200	1.78249700
H	11.05074000	-3.21071900	-1.39049000
H	15.19476700	-2.10157200	2.08543000
C	10.09632100	-2.15706600	0.67797300
S	-10.48762700	-1.20801500	3.45499400
S	-9.82066700	-0.00713200	-2.91711300
C	-10.04991600	0.59076700	3.41020300
H	-9.27441400	0.68066400	4.17352900
C	-9.55301000	1.00723600	2.05905900
C	-10.34356300	1.77194200	1.19615800
C	-9.99748500	1.90866500	-0.14298600
H	-10.66350100	2.43471600	-0.81709600
C	-8.85558000	1.29818500	-0.66209800
C	-7.97699300	0.65085000	0.23567200
C	-8.34315200	0.51176200	1.58283400
H	-7.70586500	-0.06608800	2.24025600
C	-8.65444500	1.20572400	-2.14085800
H	-7.63006800	0.94273600	-2.39961600
H	-8.90812400	2.14090700	-2.64297100
C	-11.88529300	-1.33515600	2.24922500
H	-12.71597500	-1.76918800	2.81005900
H	-12.17350500	-0.31904200	1.97109200
C	-11.63288800	-2.17470900	0.98370400
C	-12.97104400	-2.30876300	0.22313500
H	-13.35322500	-1.31221400	-0.02717000
H	-13.71747800	-2.78951900	0.86573500
C	-12.77581200	-3.13317900	-1.06163200
H	-13.73362800	-3.21606600	-1.58478000
C	-11.75648500	-2.43295200	-1.97472900

H	-11.62224000	-3.00845200	-2.89833300
H	-12.12585300	-1.44362600	-2.26038100
C	-10.40490000	-2.28766800	-1.24931000
C	-10.61979100	-1.49574800	0.04950400
H	-9.66845700	-1.39721800	0.57130200
H	-10.96903900	-0.49278900	-0.19627900
C	-11.12080500	-3.58671200	1.32788700
H	-11.83794600	-4.09292700	1.98496300
H	-10.17739800	-3.50915400	1.87626100
C	-10.91815000	-4.40393800	0.04107300
H	-10.54038500	-5.39793600	0.30017100
C	-12.25949100	-4.53475900	-0.69828800
H	-12.98742700	-5.05418200	-0.06561700
H	-12.13437200	-5.13505700	-1.60594700
C	-9.89715900	-3.69636700	-0.86800900
H	-9.73040400	-4.28975000	-1.77421000
H	-8.93264500	-3.61250700	-0.35431700
C	-9.34731100	-1.62318800	-2.14869700
H	-9.12064400	-2.28472400	-2.98773600
H	-8.41884000	-1.47041300	-1.59627900
H	-11.26653400	2.21041700	1.55904100
H	-10.91108200	1.18185000	3.72346100
C	-7.20031200	4.16349000	-0.56577700
C	-5.87896800	3.61495900	-0.75731000
H	-5.42293400	3.68481500	-1.73646800
C	-5.23864800	2.98045500	0.24805000
H	-4.26851700	2.53102300	0.08102900
C	-7.77742100	4.10100400	0.75580400
H	-8.74606300	4.55115800	0.92282200
C	-7.13869200	3.46470400	1.76040600
H	-7.59171800	3.40036400	2.73991400
C	-5.86083100	2.83008400	1.54255900
C	-5.27550200	2.05623300	2.52828500
C	-7.90561600	4.70695700	-1.62505200
C	-5.95347900	1.79917500	3.75080000
C	-4.01449300	1.42696300	2.34593800
C	-9.24096900	5.16464300	-1.46354600
C	-7.35729600	4.77495800	-2.93441500
N	-6.55030900	1.58109000	4.71676600
N	-2.98377900	0.92642800	2.19654900
N	-6.90255800	4.81725800	-3.99645600
N	-10.33691700	5.50174000	-1.31492400

AFA-TCNQ-9

Electronic Energy (EE): -4631.873032 Hartree

EE + Thermal Free Energy Correction: -4630.832530 Hartree

E (Thermal): 769.391 kcal/mol

Entropy (S): 392.615 cal/mol-kelvin

Imaginary frequencies: 0

0	1		
H	-1.41174800	3.79227100	-0.58049000
C	-1.69786100	2.74923000	-0.64062000
C	-2.45425400	0.03228700	-0.77109000
C	-0.81259500	1.78578400	-1.12198000
C	-2.95433400	2.35350200	-0.20334200
C	-3.34307600	0.99768400	-0.24731400
C	-1.20627600	0.43288400	-1.20516700
H	-3.64594500	3.08449700	0.19547400
H	-2.75552800	-1.00775200	-0.80021200
C	0.59900200	1.88297400	-1.47806200
C	3.32099300	1.45536700	-1.92182700
C	1.47396200	2.96802200	-1.44313600
C	1.07747200	0.58995900	-1.78050700
C	2.42288100	0.36728000	-1.99710800
C	2.82736300	2.75135200	-1.66211400
H	1.11822000	3.96743700	-1.22373300
H	2.80709200	-0.62607000	-2.19414600
H	3.52466700	3.57799200	-1.61469200
C	-0.06759200	-0.39838100	-1.75396300
H	0.14316000	-1.28781400	-1.15776500
H	-0.29792900	-0.75157600	-2.76593100
C	4.70972900	1.24304900	-2.08574900
C	-4.61229300	0.60639000	0.23910700
C	5.90456000	1.05512600	-2.20600000
C	7.23633500	0.84719000	-2.32859600
C	-5.69988200	0.26821100	0.66370500
C	-6.91595700	-0.10478000	1.12632800
C	-8.01210600	-0.43076600	1.53846200
S	10.59341000	2.42810300	0.55089300
S	11.63125600	-3.60531600	-1.68403200
C	10.30047100	2.60604800	-1.27191300

H	10.88860600	3.48885900	-1.52818500
C	10.75543600	1.40282000	-2.03775700
C	9.83083600	0.43658100	-2.49890300
C	10.29452400	-0.80863400	-2.95007400
H	9.57017000	-1.56905800	-3.21547100
C	11.65071300	-1.11522200	-2.94423400
C	12.56138900	-0.10506600	-2.62057800
C	12.11484400	1.13320000	-2.17792300
H	12.83201100	1.87622700	-1.84769300
C	12.10583500	-2.53278900	-3.11860700
H	13.18375600	-2.59285800	-3.27233300
H	11.61550400	-3.02009200	-3.96353300
C	9.52160900	0.99835500	1.03280600
H	8.87784400	1.37543100	1.83084300
H	8.88070200	0.76141900	0.18290700
C	10.25014500	-0.26444100	1.52467100
C	9.19044800	-1.26717200	2.03317200
H	8.49055500	-1.50388900	1.22382800
H	8.60746600	-0.81356000	2.84310300
C	9.87033500	-2.55400400	2.53467900
H	9.10431100	-3.25103900	2.88868300
C	10.65754600	-3.20600800	1.38576400
H	11.13419900	-4.12996200	1.73460200
H	9.97869600	-3.47832500	0.57253000
C	11.72817600	-2.23237000	0.85679600
C	11.03653900	-0.94155300	0.39131800
H	11.78386600	-0.24602200	0.00887200
H	10.35723200	-1.17840300	-0.42716200
C	11.21637400	0.05636900	2.68125800
H	10.66613400	0.52902800	3.50368600
H	11.96805900	0.77426100	2.33998500
C	11.89911600	-1.22899700	3.17504600
H	12.59223700	-0.98322000	3.98588900
C	10.83259000	-2.21200400	3.68381000
H	10.28145100	-1.76719500	4.51957300
H	11.30739600	-3.12493000	4.05967500
C	12.68240900	-1.87400900	2.01814200
H	13.19387100	-2.77699600	2.37093200
H	13.45485600	-1.18354900	1.65970400
C	12.56537200	-2.86338400	-0.27044400
H	13.16753700	-3.67695900	0.14013700
H	13.25301600	-2.12228200	-0.68360900
H	9.25009400	2.83051900	-1.44628800
H	13.62539800	-0.31142700	-2.65824400
C	8.43482300	0.66810800	-2.42382600

S	-11.66942900	-4.01718200	-0.12243300
S	-10.92826000	2.36759400	0.95362700
C	-11.47783400	-3.91544800	1.71789100
H	-10.76433700	-4.71242500	1.93588300
C	-10.97846200	-2.57446700	2.16428600
C	-11.83842400	-1.65574400	2.77338900
C	-11.46248900	-0.32702300	2.92062700
H	-12.17234300	0.39143200	3.31482000
C	-10.22851400	0.12968600	2.46316800
C	-9.30110500	-0.82204500	1.97831500
C	-9.69033500	-2.16336500	1.84079200
H	-8.99884900	-2.86196100	1.38562900
C	-9.96685000	1.59869600	2.34068000
H	-8.90905300	1.81724900	2.20824200
H	-10.32397900	2.14276100	3.21670700
C	-12.97808800	-2.76120300	-0.48484700
H	-13.76552600	-3.30458400	-1.01188100
H	-13.39063900	-2.43065700	0.47086500
C	-12.55372000	-1.54198400	-1.32342200
C	-13.82653200	-0.74462600	-1.68627300
H	-14.34247800	-0.43836600	-0.76873300
H	-14.51754800	-1.38325300	-2.24847500
C	-13.46151700	0.49541200	-2.52099400
H	-14.37545300	1.04536000	-2.76703800
C	-12.52274900	1.40391800	-1.71110300
H	-12.26798400	2.29644300	-2.29497400
H	-13.02319300	1.74490900	-0.79995700
C	-11.23700600	0.64197200	-1.33650000
C	-11.61681800	-0.61309300	-0.53542200
H	-10.71214700	-1.16114500	-0.27321400
H	-12.10057200	-0.31161800	0.39388100
C	-11.85177100	-1.96396000	-2.62869600
H	-12.51097600	-2.61875000	-3.21121300
H	-10.95274300	-2.53910700	-2.38912100
C	-11.47977000	-0.72183200	-3.45513300
H	-10.96897600	-1.03656700	-4.37064700
C	-12.75706600	0.05443400	-3.81423500
H	-13.42376500	-0.57631200	-4.41242000
H	-12.50905800	0.93059600	-4.42318600
C	-10.54096500	0.18337300	-2.63746700
H	-10.25262300	1.05703700	-3.23318600
H	-9.62039900	-0.35719000	-2.38968800
C	-10.25721300	1.53683600	-0.55770700
H	-9.91654500	2.34686500	-1.20658100
H	-9.37613200	0.96729600	-0.25989800

H	-12.83026500	-1.97138900	3.07793300
H	-12.42558500	-4.17037500	2.19319800
C	0.68132600	2.58786200	1.93529100
C	1.93793600	1.95856500	1.60321300
H	2.80841000	2.58083800	1.44717000
C	2.01931800	0.62114700	1.44035900
H	2.95538800	0.16234700	1.15148300
C	-0.45660400	1.74099600	2.20491500
H	-1.39064300	2.19898900	2.50020300
C	-0.37617000	0.40359600	2.04153900
H	-1.24539300	-0.21928500	2.20495500
C	0.85319500	-0.21430800	1.60351000
C	0.90207300	-1.56507500	1.31018000
C	0.55824700	3.96558500	1.94468300
C	-0.25241100	-2.38876100	1.40652500
C	2.08536200	-2.17432600	0.81139400
C	-0.68806700	4.59756100	2.20236900
C	1.65014700	4.80989200	1.60694900
N	-1.20826300	-3.03695800	1.45884800
N	3.04660600	-2.64636600	0.37581800
N	2.54274100	5.47783700	1.30062100
N	-1.71701600	5.09147900	2.38676800

AFA-TCNQ-10

Electronic Energy (EE): -4631.872966 Hartree

EE + Thermal Free Energy Correction: -4630.831628 Hartree

E (Thermal): 769.400 kcal/mol

Entropy (S): 390.886 cal/mol-kelvin

Imaginary frequencies: 0

0	1		
H	1.33004900	-2.54070600	-2.80240400
C	1.64507000	-1.87911900	-2.00456100
C	2.48260600	-0.17698100	0.08628200
C	0.71920300	-1.11991400	-1.29025500
C	2.98767100	-1.80718100	-1.65930100
C	3.42110400	-0.97564700	-0.60500400
C	1.14900700	-0.25249600	-0.26294800

H	3.71687600	-2.40958500	-2.18574800
H	2.81941000	0.45804700	0.89656100
C	-0.73987200	-1.13139000	-1.30980700
C	-3.46104000	-1.03037600	-0.69688600
C	-1.63448300	-1.90348000	-2.04977800
C	-1.21056800	-0.27169200	-0.29397500
C	-2.55395800	-0.21819200	0.02015300
C	-2.98690200	-1.85231200	-1.74099700
H	-1.28804500	-2.55886900	-2.83971500
H	-2.92228800	0.40971500	0.82220600
H	-3.69252400	-2.46445100	-2.28796400
C	-0.04480100	0.44664600	0.34899300
H	-0.05841800	0.40369600	1.43924700
H	-0.05014000	1.50978800	0.08121200
C	-4.83324500	-1.03309200	-0.35389900
C	4.78417700	-0.95491000	-0.22789500
C	-6.00697500	-1.04496100	-0.03811500
C	-7.31239100	-1.05038700	0.31943800
C	5.95033500	-0.94677300	0.11495400
C	7.24866500	-0.93460300	0.49728400
C	8.41491000	-0.92906100	0.84008900
S	-9.65645100	2.32753400	2.62949500
S	-13.15766800	-0.63192800	-2.00231500
C	-9.34645900	0.52967900	2.96257700
H	-9.51499200	0.44778400	4.03765800
C	-10.27703600	-0.35467200	2.19237200
C	-9.84885900	-1.02670100	1.02345200
C	-10.79866200	-1.60650000	0.16844400
H	-10.46278400	-2.03886000	-0.76625100
C	-12.15871800	-1.52634700	0.44573700
C	-12.55856900	-0.98655300	1.67184600
C	-11.62761500	-0.41368000	2.52836400
H	-11.96273800	0.07855100	3.43435900
C	-13.16667500	-1.85822400	-0.61283600
H	-14.17407200	-1.92812800	-0.20124300
H	-12.93958600	-2.80082500	-1.11453800
C	-9.27499600	2.51296200	0.82819000
H	-8.51688200	3.29738900	0.77187400
H	-8.80921900	1.58667100	0.48983300
C	-10.45726200	2.88465200	-0.08390900
C	-9.90601000	3.16396900	-1.50004600
H	-9.38253800	2.27574100	-1.87165600
H	-9.17334700	3.97818700	-1.46030800
C	-11.05354300	3.53775700	-2.45561400
H	-10.64368600	3.73039800	-3.45212800

C	-12.05965000	2.37769300	-2.53614700
H	-12.87322300	2.63363500	-3.22551400
H	-11.57013000	1.48242900	-2.93029200
C	-12.63354200	2.07528000	-1.13844600
C	-11.47316600	1.73660900	-0.18970600
H	-11.86759700	1.51499600	0.80208400
H	-10.96902200	0.84081600	-0.55178400
C	-11.18147300	4.14858100	0.41790700
H	-10.47065900	4.98075200	0.48618500
H	-11.56955600	3.97073800	1.42515400
C	-12.33135900	4.51521200	-0.53385500
H	-12.84477300	5.40538800	-0.15670900
C	-11.76571800	4.79668700	-1.93520000
H	-11.06555900	5.63818400	-1.89450700
H	-12.57274300	5.08097000	-2.61937200
C	-13.33129400	3.34794500	-0.60836800
H	-14.16790800	3.61387900	-1.26473800
H	-13.74962300	3.15084500	0.38562600
C	-13.67598500	0.94350100	-1.18323600
H	-14.55446900	1.28420300	-1.73577000
H	-14.00107900	0.69590900	-0.17038200
H	-8.30175400	0.30187900	2.76067500
H	-13.61220200	-0.96118200	1.92744300
C	-8.48103200	-1.04952100	0.65392000
S	11.71510100	2.88188400	2.75181400
S	11.11581900	-1.30241900	-2.20945200
C	11.89527300	1.22708700	3.56626500
H	11.25041000	1.31211200	4.44300100
C	11.47829100	0.10237400	2.66737300
C	12.43086900	-0.72373200	2.06332800
C	12.07409800	-1.56608500	1.01848700
H	12.83899100	-2.13532600	0.50240100
C	10.76664700	-1.60963400	0.53973600
C	9.77354400	-0.88621600	1.24051200
C	10.14570300	-0.04231100	2.29833400
H	9.38589900	0.57150900	2.76661800
C	10.46441700	-2.27150200	-0.76861900
H	9.39850400	-2.44367900	-0.90450500
H	10.98251300	-3.22738600	-0.86264900
C	12.90891100	2.79373300	1.34191000
H	13.58184800	3.64362400	1.47584100
H	13.49996800	1.88404300	1.46821900
C	12.31030500	2.83834700	-0.07558300
C	13.47543900	2.96541300	-1.08257900
H	14.15832100	2.11607200	-0.96362300

H	14.05032100	3.87483400	-0.87305000
C	12.93584100	3.00610600	-2.52306400
H	13.77556600	3.09590300	-3.21952300
C	12.16346200	1.71100800	-2.82081000
H	11.78656500	1.72675200	-3.85043700
H	12.82951900	0.84758000	-2.73291800
C	10.98730600	1.55450700	-1.83763900
C	11.53522700	1.55232500	-0.40202600
H	10.70796400	1.44851000	0.29981900
H	12.18963200	0.69052700	-0.27011900
C	11.37011300	4.04515300	-0.26009000
H	11.91005800	4.97456800	-0.04271200
H	10.54389200	3.97560300	0.45335700
C	10.82539400	4.07674300	-1.69771800
H	10.14813100	4.92943100	-1.80890400
C	11.99599800	4.21167000	-2.68499600
H	12.53992400	5.14382100	-2.49685100
H	11.62067600	4.25873400	-3.71306100
C	10.05441900	2.77678800	-1.98915600
H	9.64245000	2.80692300	-3.00436600
H	9.20824800	2.67987700	-1.29947600
C	10.16883400	0.28567900	-2.13379700
H	9.69173100	0.38543300	-3.11135800
H	9.37486800	0.16414200	-1.39595800
H	13.46810300	-0.66065600	2.37362300
H	12.92237700	1.10610700	3.91204100
C	-0.00658300	-4.33508200	-0.13059000
C	-1.26292500	-3.93617700	0.45905900
H	-2.18360800	-4.29249500	0.01783600
C	-1.29485500	-3.09237700	1.51202000
H	-2.24150100	-2.76636900	1.92156800
C	1.21497900	-3.90502400	0.50807300
H	2.16113700	-4.23729300	0.10347300
C	1.18383200	-3.06099000	1.56091000
H	2.10503800	-2.71099600	2.00707600
C	-0.07264700	-2.57555000	2.08096700
C	-0.10452900	-1.61105300	3.07175600
C	0.02593200	-5.06643900	-1.30424200
C	1.08903500	-1.04463100	3.59617200
C	-1.33200400	-1.07402400	3.54629900
C	1.25413200	-5.40501600	-1.93352400
C	-1.16743700	-5.43356100	-1.98265000
N	2.06824500	-0.57082800	3.98757000
N	-2.33796600	-0.62469200	3.89654200
N	-2.14890600	-5.69745700	-2.53383000

N	2.26311400	-5.64568700	-2.44415900

6.0 P6*p*PP6-TCNQ coordinates

P6*p*PP6

Electronic Energy (EE): -1943.237470 Hartree

EE + Thermal Free Energy Correction: -1942.367071 Hartree

E (Thermal): 650.422 kcal/mol

Entropy (S): 351.604 cal/mol-kelvin

Imaginary frequencies: 0

C	11.16394	-0.90181	-1.30471
H	12.08647	-0.61335	-1.81948
H	10.72223	-1.71728	-1.88656
C	11.51067	-1.41589	0.102371
H	10.58439	-1.6964	0.616777
H	11.94528	-0.59537	0.684987
C	12.47316	-2.60418	0.086399
H	12.03286	-3.41939	-0.50157
H	13.39536	-2.31729	-0.43467
C	12.82151	-3.11986	1.484095
H	13.26206	-2.30465	2.071959
H	11.89909	-3.40553	2.005289
C	13.78359	-4.31032	1.472699
H	14.70461	-4.02369	0.951307
H	13.34205	-5.12395	0.88512
C	14.1254	-4.81798	2.87465
H	14.81284	-5.66708	2.837496
H	13.22506	-5.14099	3.406167
H	14.59755	-4.03272	3.472949
C	7.488038	3.644688	-0.99797
H	6.656612	3.515528	-1.69788
H	8.009789	4.562085	-1.28701
C	6.919126	3.807257	0.422346
H	7.744483	3.993031	1.119264
H	6.457466	2.864337	0.731236
C	5.889157	4.932723	0.521752

H	6.347184	5.878655	0.205465
H	5.074671	4.735369	-0.18631
C	5.30738	5.095344	1.927221
H	4.853854	4.147023	2.24158
H	6.120725	5.293484	2.636876
C	4.264971	6.211334	2.030461
H	3.45394	6.011934	1.32
H	4.718886	7.15866	1.715902
C	3.686463	6.362798	3.438465
H	2.945282	7.165089	3.484133
H	4.4727	6.593636	4.163837
H	3.198535	5.439044	3.764107
C	10.21493	0.270379	-1.27271
C	10.69037	1.580187	-1.16012
C	8.837972	0.075085	-1.30496
H	8.436141	-0.92757	-1.39668
C	7.941222	1.15451	-1.2262
C	9.812381	2.65693	-1.08428
H	10.2065	3.664606	-1.00536
C	8.431535	2.476885	-1.11557
C	6.545514	0.912041	-1.26
C	5.343494	0.729973	-1.27519
C	4.003374	0.538726	-1.28215
C	2.798559	0.374525	-1.28451
H	11.76057	1.758136	-1.13864
C	-11.1639	0.901852	-1.30477
H	-12.0864	0.613388	-1.81953
H	-10.7222	1.717342	-1.88661
C	-11.5106	1.415878	0.10233
H	-10.5843	1.696381	0.616731
H	-11.9452	0.59533	0.68492
C	-12.4731	2.604165	0.086418
H	-12.0328	3.419384	-0.50156
H	-13.3953	2.317279	-0.43461
C	-12.8214	3.119813	1.484139
H	-13.2619	2.304582	2.072009
H	-11.899	3.405488	2.005294
C	-13.7835	4.310254	1.472806
H	-14.7045	4.023629	0.951426
H	-13.342	5.123916	0.885245
C	-14.1253	4.817865	2.874783

H	-14.8127	5.666966	2.837675
H	-13.2249	5.140846	3.406299
H	-14.5974	4.032583	3.473057
C	-7.48797	-3.64463	-0.9981
H	-6.6565	-3.51543	-1.69794
H	-8.0097	-4.56202	-1.28721
C	-6.91917	-3.80724	0.422259
H	-7.74459	-3.99295	1.119116
H	-6.45748	-2.86434	0.731174
C	-5.88928	-4.93276	0.52175
H	-6.34735	-5.87867	0.205461
H	-5.07473	-4.73548	-0.18627
C	-5.30759	-5.09539	1.927259
H	-4.85401	-4.14709	2.241613
H	-6.121	-5.29344	2.636873
C	-4.26528	-6.21146	2.030596
H	-3.45419	-6.01214	1.320181
H	-4.71925	-7.15875	1.716036
C	-3.68688	-6.36293	3.438642
H	-2.94575	-7.16527	3.484377
H	-4.47317	-6.59369	4.163968
H	-3.19891	-5.4392	3.764293
C	-10.2149	-0.27033	-1.27281
C	-10.6903	-1.58015	-1.16031
C	-8.83792	-0.07502	-1.30501
H	-8.43609	0.92764	-1.39668
C	-7.94117	-1.15445	-1.22626
C	-9.81232	-2.65689	-1.08449
H	-10.2064	-3.66457	-1.00562
C	-8.43147	-2.47683	-1.11571
H	-11.7605	-1.7581	-1.13888
C	1.397349	0.186539	-1.2855
C	-1.39729	-0.18651	-1.28549
C	0.845587	-1.10949	-1.28597
C	0.524729	1.292362	-1.28525
C	-0.84553	1.109517	-1.28554
C	-0.52467	-1.29233	-1.28568
H	1.509732	-1.9644	-1.28637
H	0.941278	2.291617	-1.28514
H	-1.50967	1.964433	-1.2856
H	-0.94122	-2.29159	-1.2859

C	-2.7985	-0.37449	-1.28451
C	-4.00332	-0.53868	-1.28215
C	-5.34344	-0.72991	-1.2752
C	-6.54546	-0.91197	-1.26003

P6*p*PP6-TCNQ-1

Electronic Energy (EE): -2622.066598 Hartree

EE + Thermal Free Energy Correction: -2621.076499 Hartree

E (Thermal): 741.224 kcal/mol

Entropy (S): 404.224 cal/mol-kelvin

Imaginary frequencies: 0

C	-11.251	0.478078	-0.7041
H	-12.1991	0.091554	-1.09245
H	-11.0832	1.444225	-1.19096
C	-11.3756	0.692299	0.813571
H	-10.4216	1.069718	1.199
H	-11.5368	-0.27731	1.298762
C	-12.5028	1.65368	1.192588
H	-12.3366	2.61959	0.699151
H	-13.4536	1.271295	0.800193
C	-12.6293	1.872042	2.701614
H	-12.7956	0.906129	3.195136
H	-11.6779	2.253126	3.0935
C	-13.7551	2.835223	3.085937
H	-14.7051	2.453314	2.693597
H	-13.5876	3.79964	2.591943
C	-13.8727	3.047023	4.596341
H	-14.683	3.738079	4.842758
H	-12.9465	3.458628	5.008823
H	-14.0718	2.102364	5.11158
C	-6.87132	-3.22214	-1.89665
H	-6.25033	-2.80507	-2.69523
H	-7.25979	-4.17576	-2.26457
C	-5.98659	-3.45848	-0.6535

H	-6.46648	-4.2017	-0.00683
H	-5.92134	-2.53911	-0.06825
C	-4.56909	-3.90619	-1.01489
H	-4.60553	-4.77393	-1.68601
H	-4.08778	-3.09913	-1.58174
C	-3.71575	-4.24614	0.208769
H	-3.78171	-3.42292	0.929864
H	-4.1388	-5.12304	0.714215
C	-2.24718	-4.51845	-0.12791
H	-1.80876	-3.6163	-0.57264
H	-2.18797	-5.29272	-0.90136
C	-1.42148	-4.95033	1.085703
H	-0.3682	-5.09661	0.831175
H	-1.79523	-5.89234	1.496496
H	-1.47648	-4.20893	1.889791
C	-10.1337	-0.47032	-1.06068
C	-10.3521	-1.8496	-1.13029
C	-8.84207	-0.00136	-1.27841
H	-8.63669	1.062099	-1.23535
C	-7.78456	-0.8825	-1.55542
C	-9.31184	-2.73217	-1.40945
H	-9.51208	-3.7968	-1.46435
C	-8.01414	-2.27947	-1.62619
C	-6.47334	-0.39704	-1.76277
C	-5.30688	-0.10139	-1.93935
C	-3.9873	0.130037	-2.0959
C	-2.78412	0.269402	-2.22026
H	-11.3528	-2.23678	-0.96852
C	11.29743	-0.12644	-0.76135
H	12.25101	0.356981	-0.99819
H	11.16404	-0.93135	-1.49146
C	11.37429	-0.73257	0.649821
H	10.41474	-1.20631	0.886299
H	11.50138	0.076073	1.378829
C	12.50521	-1.75026	0.804405
H	12.37309	-2.5535	0.068553
H	13.4617	-1.26997	0.561967
C	12.58482	-2.35734	2.206544
H	12.71701	-1.55412	2.942446
H	11.62781	-2.83626	2.448847
C	13.71423	-3.37812	2.365772

H	14.66986	-2.89839	2.122901
H	13.58063	-4.17998	1.629998
C	13.78497	-3.97888	3.7708
H	14.59886	-4.70353	3.856896
H	12.85301	-4.49234	4.026133
H	13.9498	-3.2014	4.523054
C	6.89236	3.71692	-1.10446
H	6.336972	3.543416	-2.03154
H	7.27517	4.73988	-1.15401
C	5.918243	3.57429	0.086785
H	6.311574	4.133375	0.943201
H	5.867455	2.529281	0.398259
C	4.504122	4.048823	-0.25329
H	4.532049	5.076681	-0.63745
H	4.120186	3.425135	-1.06998
C	3.539438	3.976628	0.931884
H	3.60105	2.979567	1.384324
H	3.863407	4.679588	1.709252
C	2.087067	4.279829	0.551935
H	1.741389	3.528067	-0.16937
H	2.042779	5.23913	0.023462
C	1.143993	4.321588	1.756306
H	0.107939	4.500329	1.455268
H	1.430121	5.121161	2.445404
H	1.175024	3.383514	2.318784
C	10.17571	0.872793	-0.89728
C	10.37162	2.223346	-0.5941
C	8.900113	0.465976	-1.27668
H	8.712607	-0.57352	-1.52021
C	7.837256	1.379338	-1.352
C	9.324876	3.138947	-0.66843
H	9.507069	4.181949	-0.43279
C	8.043698	2.747679	-1.04417
H	11.35924	2.56297	-0.29958
C	-1.38014	0.336499	-2.30705
C	1.432989	0.422963	-2.35489
C	-0.70443	1.566682	-2.44827
C	-0.62273	-0.84951	-2.21804
C	0.756217	-0.80777	-2.23893
C	0.675813	1.60644	-2.47875
H	-1.2818	2.479765	-2.51736

H	-1.13902	-1.79538	-2.11755
H	1.332307	-1.72	-2.15128
H	1.1923	2.55283	-2.57473
C	2.840244	0.477998	-2.29485
C	4.04741	0.562853	-2.17289
C	5.371888	0.729981	-1.97487
C	6.537936	0.958283	-1.7175
C	-1.30124	0.211969	0.959864
C	-1.03864	-1.19207	1.157287
H	-1.87632	-1.86737	1.271781
C	0.226749	-1.66719	1.179795
H	0.406512	-2.72564	1.313667
C	-0.17277	1.092457	0.806389
H	-0.35301	2.146107	0.647969
C	1.093934	0.623394	0.862329
H	1.930717	1.299067	0.746946
C	1.356958	-0.78007	1.047621
C	2.65546	-1.26305	1.112299
C	-2.59787	0.700522	0.911145
C	3.779593	-0.39505	1.130393
C	2.919047	-2.65623	1.20642
C	-2.8491	2.071502	0.63602
C	-3.7233	-0.13344	1.138316
N	4.689652	0.315155	1.195837
N	3.107819	-3.79559	1.267022
N	-4.63108	-0.81754	1.349071
N	-3.00973	3.191015	0.395911

P6*p*PP6-TCNQ-2

Electronic Energy (EE): -2622.065985 Hartree

EE + Thermal Free Energy Correction: -2621.079998 Hartree

E (Thermal): 741.397 kcal/mol

Entropy (S): 413.459 cal/mol-kelvin

Imaginary frequencies: 0

C	-10.0092	0.371973	-0.6941

H	-10.6348	0.106883	0.162358
H	-10.6584	0.296447	-1.57585
C	-9.54949	1.822065	-0.53847
H	-9.04283	2.15866	-1.45026
H	-8.8144	1.885167	0.27065
C	-10.7077	2.769272	-0.22351
H	-11.4602	2.703496	-1.01938
H	-11.1971	2.432864	0.697254
C	-10.2661	4.224521	-0.06071
H	-9.5145	4.285509	0.736716
H	-9.76585	4.560257	-0.97824
C	-11.419	5.177774	0.262731
H	-11.9182	4.838862	1.177861
H	-12.1697	5.117779	-0.53422
C	-10.9666	6.629098	0.432371
H	-11.8083	7.287424	0.662316
H	-10.4909	7.00246	-0.4798
H	-10.2397	6.721661	1.245172
C	-5.97672	-3.78457	-1.62081
H	-5.59096	-3.54577	-2.61795
H	-6.50048	-4.73962	-1.71057
C	-4.77907	-3.92407	-0.66399
H	-5.0776	-4.50407	0.216502
H	-4.50559	-2.93485	-0.30436
C	-3.5427	-4.53975	-1.32011
H	-3.73892	-5.57847	-1.61239
H	-3.3351	-3.99135	-2.24717
C	-2.30597	-4.47028	-0.42208
H	-2.15471	-3.43196	-0.1051
H	-2.47835	-5.0498	0.49396
C	-1.02453	-4.96113	-1.09932
H	-0.86598	-4.3794	-2.01616
H	-1.15648	-6.00023	-1.423
C	0.205716	-4.84972	-0.19608
H	1.112811	-5.17215	-0.71478
H	0.093706	-5.47617	0.694066
H	0.352969	-3.8221	0.146476
C	-8.91487	-0.66398	-0.82563
C	-9.20173	-2.01554	-0.58759
C	-7.61681	-0.34151	-1.20142
H	-7.33077	0.688601	-1.36383

C	-6.63907	-1.33672	-1.39313
C	-8.25351	-3.01142	-0.80721
H	-8.52447	-4.04873	-0.64653
C	-6.96801	-2.70915	-1.246
C	-5.31682	-0.96445	-1.7122
C	-4.13912	-0.71608	-1.89599
C	-2.81404	-0.49623	-2.00212
C	-1.60816	-0.3264	-2.01613
H	-10.1953	-2.29346	-0.25223
C	12.31004	-0.92398	-0.94247
H	13.27466	-0.661	-1.38913
H	11.91975	-1.77129	-1.51549
C	12.53029	-1.35896	0.515874
H	11.56169	-1.61399	0.960905
H	12.91264	-0.50609	1.08857
C	13.48873	-2.54292	0.651017
H	13.10173	-3.39066	0.071653
H	14.45433	-2.28172	0.199778
C	13.70928	-2.97975	2.10066
H	14.09608	-2.13179	2.679946
H	12.74333	-3.23977	2.551639
C	14.66704	-4.16537	2.241594
H	15.63171	-3.90433	1.790445
H	14.27937	-5.01175	1.66233
C	14.88018	-4.59385	3.694528
H	15.56697	-5.44122	3.766367
H	13.9351	-4.89035	4.15967
H	15.29753	-3.77518	4.288764
C	8.627583	3.621301	-1.19507
H	7.859281	3.452454	-1.95603
H	9.172453	4.524425	-1.48608
C	7.938525	3.854883	0.160874
H	8.699684	4.091081	0.913316
H	7.46124	2.924316	0.483114
C	6.892305	4.9685	0.108506
H	7.365808	5.902673	-0.21961
H	6.144537	4.719967	-0.65487
C	6.188752	5.198186	1.447347
H	5.722933	4.260454	1.775174
H	6.934543	5.45089	2.21175
C	5.125442	6.298014	1.396637

H	4.382194	6.043828	0.6317
H	5.59139	7.234979	1.068876
C	4.424345	6.516005	2.738625
H	3.670515	7.305037	2.674258
H	5.140936	6.801759	3.514839
H	3.922903	5.602595	3.072651
C	11.36372	0.245154	-1.05716
C	11.82962	1.560913	-0.97737
C	9.994128	0.044231	-1.19406
H	9.599485	-0.96304	-1.26353
C	9.096184	1.124158	-1.24947
C	10.95048	2.637992	-1.03406
H	11.33855	3.649444	-0.97738
C	9.576845	2.452601	-1.17037
H	12.8945	1.742971	-0.87581
C	-0.21117	-0.14965	-1.9685
C	2.571928	0.186845	-1.77806
C	0.349377	1.142796	-1.94654
C	0.639925	-1.27004	-1.89071
C	2.007379	-1.10429	-1.79848
C	1.717918	1.30597	-1.85209
H	-0.30404	2.004347	-1.98177
H	0.209294	-2.26211	-1.88872
H	2.657435	-1.96678	-1.73043
H	2.14733	2.299163	-1.82837
C	3.970473	0.359117	-1.67916
C	5.173485	0.513634	-1.59106
C	6.509856	0.697587	-1.48811
C	7.708024	0.877439	-1.38519
C	-6.48333	-0.94186	2.011337
C	-6.17968	0.41158	1.617974
H	-6.98643	1.130636	1.570055
C	-4.91794	0.779864	1.295406
H	-4.70935	1.794161	0.979762
C	-5.39515	-1.88283	2.06635
H	-5.60697	-2.9044	2.352932
C	-4.12823	-1.50479	1.783705
H	-3.32047	-2.22216	1.847518
C	-3.82572	-0.15767	1.374362
C	-2.52494	0.218573	1.074466
C	-7.77266	-1.32751	2.346218

C	-1.4426	-0.69515	1.181417
C	-2.20342	1.534468	0.647188
C	-8.08696	-2.67903	2.650394
C	-8.83376	-0.38952	2.438594
N	-0.56933	-1.44734	1.273467
N	-1.94345	2.60348	0.291769
N	-9.68645	0.385007	2.540961
N	-8.32788	-3.78597	2.882069

P6*p*PP6-TCNQ-3

Electronic Energy (EE): -2622.064915 Hartree

EE + Thermal Free Energy Correction: -2621.076414 Hartree

E (Thermal): 741.332 kcal/mol

Entropy (S): 407.952 cal/mol-kelvin

Imaginary frequencies: 0

C	11.46928	1.23231	1.597036
H	12.38651	0.635714	1.640255
H	11.12608	1.354463	2.629495
C	11.79317	2.614237	1.005561
H	10.87154	3.205571	0.957406
H	12.12832	2.489083	-0.03058
C	12.85373	3.374431	1.803026
H	12.51294	3.492055	2.839431
H	13.77115	2.774225	1.85095
C	13.17944	4.750369	1.218546
H	13.52004	4.632562	0.182046
H	12.2617	5.349973	1.169988
C	14.24009	5.515364	2.013961
H	15.15632	4.914984	2.062014
H	13.89842	5.632373	3.049223
C	14.55837	6.889182	1.421219
H	15.31741	7.413253	2.007811
H	13.66534	7.520914	1.391176
H	14.93258	6.798393	0.3969

C	7.398331	-1.53144	-1.58115
H	6.708482	-2.02341	-0.88898
H	7.861133	-2.32156	-2.17853
C	6.586402	-0.59142	-2.49885
H	7.164886	-0.36906	-3.40216
H	6.427311	0.363007	-1.99115
C	5.225895	-1.1851	-2.86593
H	5.359783	-2.11912	-3.42333
H	4.715005	-1.45288	-1.93439
C	4.324784	-0.2345	-3.65469
H	4.168203	0.677738	-3.06467
H	4.829257	0.080986	-4.5762
C	2.967609	-0.84955	-4.00814
H	2.503793	-1.2371	-3.09291
H	3.120099	-1.72386	-4.64885
C	2.013853	0.135162	-4.68556
H	1.056613	-0.33456	-4.92866
H	2.440698	0.517564	-5.61722
H	1.810516	0.99736	-4.04221
C	10.42333	0.494371	0.798995
C	10.77981	-0.31138	-0.28611
C	9.070005	0.637143	1.090886
H	8.758326	1.253334	1.926591
C	8.086961	-0.00488	0.322009
C	9.81319	-0.95878	-1.0513
H	10.11879	-1.58915	-1.87942
C	8.456267	-0.82728	-0.77194
C	6.711339	0.170168	0.606131
C	5.504663	0.279725	0.703054
C	4.158316	0.390865	0.689767
C	2.948538	0.478748	0.605093
H	11.8294	-0.44008	-0.52958
C	-10.6345	2.557883	-1.79116
H	-11.5137	2.252056	-2.36807
H	-10.0006	3.137639	-2.46985
C	-11.0826	3.456553	-0.62669
H	-10.2008	3.75531	-0.04824
H	-11.7091	2.870463	0.05557
C	-11.8444	4.700016	-1.08728
H	-11.212	5.280106	-1.77121
H	-12.7213	4.392714	-1.671

C	-12.2946	5.596301	0.068044
H	-12.9269	5.015907	0.751873
H	-11.4177	5.90276	0.652163
C	-13.0572	6.842578	-0.38827
H	-13.9326	6.534964	-0.97249
H	-12.4241	7.421797	-1.07088
C	-13.5036	7.730947	0.774284
H	-14.0448	8.612509	0.420862
H	-12.6444	8.078291	1.356178
H	-14.164	7.18518	1.455145
C	-7.77078	-2.14775	0.201823
H	-6.8708	-2.33403	-0.39013
H	-8.40881	-3.03003	0.092922
C	-7.37631	-1.98803	1.680819
H	-8.27934	-1.81975	2.278972
H	-6.7591	-1.09111	1.793177
C	-6.61198	-3.19674	2.221615
H	-7.23082	-4.09772	2.122826
H	-5.72977	-3.36944	1.59477
C	-6.17167	-3.03052	3.676268
H	-5.58756	-2.10906	3.770911
H	-7.05545	-2.90558	4.314852
C	-5.3332	-4.2016	4.192666
H	-4.47803	-4.34849	3.52268
H	-5.92389	-5.12432	4.142473
C	-4.82484	-3.98596	5.618768
H	-4.23688	-4.83852	5.969819
H	-5.65617	-3.84493	6.316588
H	-4.19138	-3.09595	5.670677
C	-9.88819	1.334293	-1.32068
C	-10.5691	0.167501	-0.96108
C	-8.50427	1.349549	-1.18407
H	-7.9439	2.236353	-1.45746
C	-7.80287	0.231934	-0.69895
C	-9.88407	-0.94464	-0.48315
H	-10.4356	-1.84074	-0.21967
C	-8.49778	-0.94609	-0.3407
H	-11.6487	0.128583	-1.06384
C	1.549397	0.537184	0.427291
C	-1.24008	0.553827	0.036813
C	0.678618	0.806062	1.500743

C	0.99975	0.296873	-0.84858
C	-0.36731	0.305899	-1.04082
C	-0.69091	0.808714	1.310941
H	1.093597	0.997594	2.482152
H	1.665161	0.100789	-1.67758
H	-0.78087	0.11618	-2.02295
H	-1.35945	0.996168	2.14138
C	-2.63852	0.521005	-0.14982
C	-3.84493	0.457948	-0.29758
C	-5.18575	0.382302	-0.45082
C	-6.39289	0.304846	-0.57299
C	1.442316	-3.1629	-0.59009
C	1.103424	-2.68648	0.727861
H	1.903418	-2.493	1.430579
C	-0.1852	-2.49364	1.088418
H	-0.4249	-2.1454	2.083535
C	0.365829	-3.3797	-1.5273
H	0.608009	-3.71459	-2.52789
C	-0.92144	-3.17969	-1.16792
H	-1.72028	-3.34985	-1.87862
C	-1.26243	-2.73675	0.162934
C	-2.58356	-2.54706	0.534576
C	2.754724	-3.43404	-0.94102
C	-3.65653	-2.82041	-0.35398
C	-2.9165	-2.06489	1.828693
C	3.090775	-3.94782	-2.22297
C	3.833856	-3.25625	-0.03389
N	-4.52127	-3.06455	-1.08113
N	-3.14141	-1.65993	2.887755
N	4.720054	-3.12484	0.696945
N	3.353807	-4.36638	-3.26798

P6*p*PP6-TCNQ-4

Electronic Energy (EE): -2622.064398 Hartree

EE + Thermal Free Energy Correction: -2621.080033 Hartree

E (Thermal): 741.032 kcal/mol

Entropy (S): 415.650 cal/mol-kelvin

Imaginary frequencies: 0

0	1		
C	11.00744	-1.01464	-1.11916
H	11.98489	-0.66886	-1.47164
H	10.67103	-1.77844	-1.82764
C	11.16787	-1.65096	0.27152
H	10.1865	-1.98898	0.623408
H	11.49621	-0.88197	0.980291
C	12.15324	-2.82035	0.2852
H	11.8204	-3.58331	-0.42982
H	13.13158	-2.47489	-0.07224
C	12.31563	-3.4584	1.6663
H	12.64824	-2.69536	2.381357
H	11.33705	-3.8029	2.023675
C	13.30042	-4.63005	1.684536
H	14.27763	-4.28437	1.326955
H	12.96683	-5.3915	0.969605
C	13.45547	-5.26078	3.069508
H	14.16322	-6.0937	3.054165
H	12.49825	-5.64336	3.436675
H	13.81849	-4.52818	3.796825
C	7.218433	3.444916	-0.89601
H	6.478632	3.386653	-1.70036
H	7.75792	4.388017	-1.02633
C	6.480137	3.46229	0.454522
H	7.215115	3.46204	1.267456
H	5.907731	2.535519	0.55921
C	5.547507	4.666649	0.592169
H	6.141543	5.589219	0.610097
H	4.907428	4.731248	-0.29339
C	4.663395	4.604338	1.838405
H	4.095778	3.666238	1.828105
H	5.291265	4.558786	2.737301
C	3.689699	5.779155	1.951915
H	3.105316	5.846016	1.028028
H	4.257582	6.71427	2.023905
C	2.746201	5.656626	3.149689
H	2.063422	6.508128	3.213677
H	3.304027	5.604571	4.089789
H	2.139655	4.747859	3.078287
C	10.03451	0.138004	-1.11584
C	10.45497	1.436828	-0.81188

C	8.682535	-0.06707	-1.36453
H	8.321473	-1.06007	-1.60644
C	7.757437	0.992222	-1.31645
C	9.54917	2.490717	-0.75966
H	9.902427	3.488996	-0.52466
C	8.190911	2.302132	-1.00806
C	6.394188	0.717587	-1.58007
C	5.230365	0.439738	-1.7993
C	3.939205	0.102046	-2.00371
C	2.777438	-0.22906	-2.15449
H	11.5062	1.623778	-0.61844
C	-10.3052	1.125377	-0.90127
H	-11.3964	1.038029	-0.90809
H	-10.027	1.662624	-1.81425
C	-9.86502	1.95091	0.319328
H	-8.77141	2.017562	0.327496
H	-10.1458	1.413359	1.232528
C	-10.4662	3.356705	0.340414
H	-10.1867	3.883128	-0.58092
H	-11.5614	3.285733	0.33067
C	-10.0231	4.184061	1.548712
H	-10.3031	3.658556	2.470522
H	-8.92803	4.251413	1.558203
C	-10.616	5.594973	1.57259
H	-11.7103	5.526092	1.562403
H	-10.3355	6.118433	0.650881
C	-10.1656	6.413777	2.783734
H	-10.6024	7.415856	2.776039
H	-9.07705	6.524642	2.800223
H	-10.4616	5.928198	3.7187
C	-7.78175	-4.15336	-0.69303
H	-7.12174	-4.31184	-1.5516
H	-8.53273	-4.94883	-0.71577
C	-6.95299	-4.26308	0.599842
H	-7.62705	-4.19284	1.461418
H	-6.27472	-3.40813	0.667749
C	-6.13759	-5.55364	0.678631
H	-6.80399	-6.4208	0.58221
H	-5.45476	-5.59483	-0.1794
C	-5.32858	-5.66808	1.972347
H	-4.69488	-4.78091	2.076402
H	-6.01429	-5.65805	2.829498
C	-4.46077	-6.92701	2.037454
H	-3.77694	-6.9357	1.180048
H	-5.09633	-7.81421	1.928261

C	-3.65522	-7.03003	3.333999
H	-3.03859	-7.93278	3.35512
H	-4.31614	-7.05732	4.205857
H	-2.99171	-6.16789	3.451763
C	-9.68542	-0.24988	-0.91407
C	-10.3883	-1.37536	-0.47505
C	-8.36398	-0.42038	-1.31417
H	-7.78104	0.429455	-1.64927
C	-7.74698	-1.68201	-1.26904
C	-9.78502	-2.63009	-0.42959
H	-10.3544	-3.48584	-0.08284
C	-8.46061	-2.81568	-0.81584
H	-11.4219	-1.26741	-0.163
C	1.426265	-0.61138	-2.26735
C	-1.30092	-1.31049	-2.34829
C	0.995943	-1.88106	-1.82507
C	0.4714	0.283784	-2.79058
C	-0.86371	-0.06071	-2.83288
C	-0.34041	-2.22398	-1.86456
H	1.72829	-2.57622	-1.43489
H	0.798743	1.253323	-3.144
H	-1.59612	0.636823	-3.21791
H	-0.6691	-3.19008	-1.50319
C	-2.67934	-1.59489	-2.29751
C	-3.88177	-1.73221	-2.16799
C	-5.21091	-1.80868	-1.94708
C	-6.39038	-1.7836	-1.65401
C	0.659703	1.452342	0.302998
C	-0.41439	2.250419	-0.23623
H	-0.17572	3.208235	-0.68074
C	-1.69712	1.825731	-0.18223
H	-2.49374	2.441388	-0.58063
C	0.319808	0.186592	0.899395
H	1.115659	-0.42666	1.301725
C	-0.95974	-0.25012	0.929271
H	-1.19505	-1.21666	1.355425
C	-2.03395	0.547502	0.394297
C	-3.34398	0.095206	0.431415
C	1.976466	1.885612	0.247329
C	-3.67736	-1.15763	1.01397
C	-4.4217	0.860648	-0.08984
C	3.02641	1.182351	0.894873
C	2.326728	3.061892	-0.46516
N	-3.92814	-2.17523	1.501057
N	-5.29741	1.486472	-0.51183

N	2.578176	4.012106	-1.07433
N	3.871587	0.628059	1.456424

P6*p*PP6-TCNQ-5

Electronic Energy (EE): -2622.061000 Hartree

EE + Thermal Free Energy Correction: -2621.079768 Hartree

E (Thermal): 741.017 kcal/mol

Entropy (S): 422.193 cal/mol-kelvin

Imaginary frequencies: 0

0	1		
C	-11.124	1.562877	0.143134
H	-11.9649	1.593912	-0.55756
H	-10.6178	2.530637	0.068108
C	-11.6633	1.38377	1.571913
H	-10.8184	1.346884	2.269085
H	-12.1613	0.410058	1.646194
C	-12.6308	2.491636	1.990603
H	-12.1268	3.462909	1.909198
H	-13.4711	2.525129	1.285613
C	-13.1697	2.319048	3.412112
H	-13.6732	1.347418	3.493551
H	-12.3291	2.285164	4.116705
C	-14.1383	3.425987	3.835572
H	-14.9775	3.458838	3.130645
H	-13.634	4.396074	3.753532
C	-14.6709	3.244217	5.258002
H	-15.3592	4.04718	5.534436
H	-13.854	3.239932	5.986136
H	-15.2074	2.295797	5.358832
C	-7.46619	-2.78762	-1.28052
H	-6.57568	-2.39299	-1.77703
H	-7.95814	-3.45229	-1.99694
C	-7.02261	-3.60339	-0.05322
H	-7.90367	-4.04821	0.424021
H	-6.57878	-2.92679	0.683639
C	-6.0113	-4.69279	-0.41274

H	-6.4671	-5.39366	-1.12381
H	-5.16623	-4.23437	-0.93762
C	-5.50131	-5.46509	0.805076
H	-5.05119	-4.76035	1.516052
H	-6.34816	-5.9258	1.329873
C	-4.47715	-6.54676	0.453056
H	-3.6354	-6.08415	-0.0759
H	-4.9279	-7.25287	-0.25431
C	-3.96055	-7.30613	1.676422
H	-3.23122	-8.07118	1.396931
H	-4.77944	-7.80393	2.204858
H	-3.4767	-6.62701	2.385342
C	-10.1737	0.461784	-0.25652
C	-10.6434	-0.72664	-0.82457
C	-8.80743	0.578347	-0.02704
H	-8.40612	1.487481	0.405802
C	-7.91913	-0.46245	-0.35092
C	-9.7722	-1.75989	-1.15533
H	-10.1628	-2.6658	-1.60618
C	-8.40071	-1.65953	-0.93306
C	-6.53794	-0.30376	-0.09161
C	-5.34824	-0.19756	0.136884
C	-4.02027	-0.10558	0.358045
C	-2.81622	-0.03392	0.523052
H	-11.7052	-0.83929	-1.01824
C	11.09178	-1.59701	0.168376
H	11.93359	-1.64741	-0.53017
H	10.57235	-2.55845	0.10295
C	11.63075	-1.40961	1.596242
H	10.78502	-1.35362	2.2912
H	12.14173	-0.44195	1.660866
C	12.58232	-2.52574	2.029161
H	12.06547	-3.491	1.957195
H	13.42357	-2.57819	1.326498
C	13.12041	-2.34484	3.449938
H	13.63651	-1.37908	3.521994
H	12.2789	-2.29227	4.152215
C	14.07352	-3.45973	3.887584
H	14.91373	-3.51122	3.184994
H	13.55665	-4.42396	3.814882
C	14.60537	-3.26953	5.309192

H	15.28245	-4.07839	5.5959
H	13.78706	-3.24664	6.035348
H	15.15407	-2.32718	5.400991
C	7.496695	2.787262	-1.31041
H	6.600948	2.397463	-1.80135
H	7.997623	3.43571	-2.03546
C	7.063066	3.625046	-0.09443
H	7.948471	4.072519	0.372183
H	6.618321	2.962886	0.654815
C	6.055607	4.713526	-0.46737
H	6.511381	5.400444	-1.19199
H	5.20583	4.249949	-0.97997
C	5.554929	5.508225	0.739859
H	5.106122	4.817033	1.464855
H	6.40606	5.974714	1.252484
C	4.532515	6.587287	0.375042
H	3.686655	6.118675	-0.14192
H	4.981902	7.280092	-0.34621
C	4.024612	7.36867	1.588158
H	3.296142	8.131219	1.299691
H	4.847725	7.87267	2.104001
H	3.542443	6.703027	2.310917
C	10.15772	-0.48725	-0.24527
C	10.64462	0.686862	-0.8282
C	8.789844	-0.58091	-0.01404
H	8.375537	-1.47855	0.430416
C	7.917144	0.468818	-0.35021
C	9.78851	1.728591	-1.1718
H	10.19213	2.622775	-1.63446
C	8.415871	1.651218	-0.9477
H	11.70785	0.781417	-1.02352
C	-1.41269	0.021784	0.622817
C	1.401497	0.121729	0.623567
C	-0.73847	1.254896	0.747945
C	-0.6515	-1.16305	0.547159
C	0.727094	-1.11423	0.54839
C	0.640831	1.303759	0.747432
H	-1.31903	2.165708	0.820351
H	-1.16584	-2.11191	0.464298
H	1.307624	-2.02415	0.465869
H	1.155293	2.253487	0.820006

C	2.805617	0.170875	0.525099
C	4.011506	0.202159	0.361458
C	5.341765	0.258833	0.141007
C	6.533488	0.336253	-0.08791
C	-1.41648	-0.09082	-2.73483
C	-0.71214	1.165041	-2.68541
H	-1.28813	2.079725	-2.63015
C	0.639168	1.209982	-2.68498
H	1.152418	2.161315	-2.62924
C	-0.63027	-1.29659	-2.81734
H	-1.14408	-2.24798	-2.87185
C	0.721397	-1.25156	-2.81834
H	1.297956	-2.16619	-2.8735
C	1.425591	0.003888	-2.73539
C	2.811053	0.051575	-2.69612
C	-2.80211	-0.1335	-2.69473
C	3.597972	-1.13063	-2.70533
C	3.508675	1.288146	-2.64762
C	-3.50691	-1.36628	-2.69454
C	-3.58212	1.053168	-2.65302
N	4.219243	-2.10561	-2.71038
N	4.060568	2.303569	-2.6212
N	-4.19811	2.031143	-2.63116
N	-4.06483	-2.37892	-2.69111

P6*p*PP6-TCNQ-6

Electronic Energy (EE): -2622.059576 Hartree

EE + Thermal Free Energy Correction: -2621.079867 Hartree

E (Thermal): 741.221 kcal/mol

Entropy (S): 426.084 cal/mol-kelvin

Imaginary frequencies: 0

C	-8.81801	0.934831	1.491383
H	-9.78083	1.155096	1.021205
H	-8.24172	1.865792	1.483471
C	-9.06473	0.513067	2.948846

H	-8.10318	0.287747	3.424011
H	-9.63645	-0.42184	2.957709
C	-9.80462	1.58247	3.753485
H	-9.23152	2.517595	3.726926
H	-10.7636	1.801895	3.26804
C	-10.0528	1.179909	5.20831
H	-10.6263	0.244623	5.233304
H	-9.09309	0.960954	5.693301
C	-10.7942	2.247915	6.016168
H	-11.7524	2.465755	5.530071
H	-10.2206	3.181805	5.989393
C	-11.038	1.837216	7.469329
H	-11.5682	2.616776	8.022432
H	-10.0936	1.644503	7.987508
H	-11.6372	0.923202	7.524635
C	-6.03608	-3.29367	-1.45182
H	-5.15193	-2.89993	-1.95931
H	-6.69407	-3.69688	-2.22749
C	-5.59895	-4.43224	-0.51335
H	-6.48485	-4.8695	-0.03879
H	-4.9876	-4.01454	0.292777
C	-4.80582	-5.51655	-1.24333
H	-5.41756	-5.93488	-2.05258
H	-3.93426	-5.05795	-1.72573
C	-4.34114	-6.64605	-0.32237
H	-3.73084	-6.22458	0.486326
H	-5.21312	-7.1041	0.161708
C	-3.5389	-7.72979	-1.04678
H	-2.6702	-7.26946	-1.53187
H	-4.14996	-8.15112	-1.85378
C	-3.07272	-8.85195	-0.11761
H	-2.50266	-9.61094	-0.65972
H	-3.9243	-9.34989	0.35622
H	-2.43348	-8.46158	0.680228
C	-8.09607	-0.12091	0.694575
C	-8.80152	-1.06346	-0.06342
C	-6.70976	-0.21843	0.73159
H	-6.1321	0.500057	1.301307
C	-6.02523	-1.22778	0.03286
C	-8.13517	-2.07416	-0.75098
H	-8.70637	-2.79072	-1.3313

C	-6.74758	-2.18365	-0.72572
C	-4.61185	-1.25991	0.070452
C	-3.39561	-1.25074	0.057488
C	-2.04619	-1.19657	0.000603
C	-0.83909	-1.0922	-0.09302
H	-9.88279	-0.99919	-0.11897
C	13.12384	-1.32844	-0.3828
H	14.02647	-1.26785	-0.9999
H	12.69796	-2.32356	-0.54718
C	13.51369	-1.18424	1.097611
H	12.60701	-1.23858	1.711021
H	13.93138	-0.18399	1.260704
C	14.51492	-2.24357	1.560284
H	14.09147	-3.24149	1.39009
H	15.41732	-2.18616	0.938495
C	14.90569	-2.10451	3.032975
H	15.32897	-1.10644	3.203073
H	14.00303	-2.16111	3.654342
C	15.90717	-3.16331	3.500858
H	16.80836	-3.10592	2.878901
H	15.48289	-4.15987	3.330415
C	16.29125	-3.01522	4.974143
H	17.00622	-3.78281	5.281589
H	15.41213	-3.10061	5.620147
H	16.74734	-2.03905	5.165417
C	9.308467	2.835563	-1.92675
H	8.46748	2.401761	-2.47663
H	9.793795	3.551042	-2.59747
C	8.763767	3.579726	-0.6951
H	9.596309	4.065308	-0.17311
H	8.341143	2.85112	0.003618
C	7.69796	4.615282	-1.05453
H	8.116856	5.342976	-1.76132
H	6.876757	4.114353	-1.58212
C	7.139176	5.353968	0.162974
H	6.725232	4.623492	0.869326
H	7.959058	5.85703	0.691286
C	6.060762	6.381358	-0.18964
H	5.243354	5.876826	-0.71834
H	6.475201	7.110763	-0.89574
C	5.505239	7.111506	1.034421

H	4.737564	7.837821	0.754749
H	6.297075	7.650449	1.563723
H	5.056268	6.407377	1.741591
C	12.13788	-0.2754	-0.82396
C	12.57236	0.966414	-1.29678
C	10.76768	-0.49405	-0.72466
H	10.39689	-1.44762	-0.36632
C	9.838225	0.497163	-1.08402
C	11.6616	1.954032	-1.6589
H	12.02431	2.906403	-2.03087
C	10.28701	1.749761	-1.56459
H	13.63638	1.15848	-1.38841
C	0.556834	-0.91195	-0.22377
C	3.329718	-0.52587	-0.49192
C	1.068569	0.34862	-0.59013
C	1.450617	-1.97524	0.002772
C	2.81372	-1.78507	-0.12795
C	2.432061	0.535399	-0.72198
H	0.379154	1.162714	-0.7746
H	1.059079	-2.94525	0.281806
H	3.498833	-2.60443	0.048174
H	2.823336	1.504075	-1.00565
C	4.723776	-0.33053	-0.62393
C	5.922345	-0.15937	-0.73564
C	7.255314	0.041367	-0.85848
C	8.450869	0.235562	-0.96388
C	-7.96738	2.125647	-1.70238
C	-6.80602	2.85165	-1.24665
H	-6.95191	3.767781	-0.68857
C	-5.55917	2.390212	-1.48684
H	-4.69569	2.93292	-1.12342
C	-7.7557	0.917428	-2.4577
H	-8.6169	0.370048	-2.81488
C	-6.50773	0.459484	-2.70459
H	-6.36326	-0.45357	-3.26608
C	-5.34797	1.167372	-2.22456
C	-4.07018	0.688722	-2.45503
C	-9.2452	2.564936	-1.39578
C	-3.84667	-0.51555	-3.17637
C	-2.91386	1.375662	-1.99578
C	-10.3883	1.803705	-1.75541

C	-9.46941	3.745875	-0.63959
N	-3.68729	-1.49736	-3.76477
N	-1.97358	1.937286	-1.62714
N	-9.63428	4.702263	-0.01086
N	-11.2998	1.146135	-2.02793

P6*p*PP6-TCNQ-7

Electronic Energy (EE): -2622.059511 Hartree

EE + Thermal Free Energy Correction: -2621.079169 Hartree

E (Thermal): 741.293 kcal/mol

Entropy (S): 424.993 cal/mol-kelvin

Imaginary frequencies: 0

C	-8.87697	2.217733	0.922158
H	-9.81419	2.302048	0.362289
H	-8.22372	3.022899	0.57131
C	-9.16338	2.42032	2.419479
H	-8.22576	2.318544	2.977755
H	-9.81773	1.614656	2.771991
C	-9.79882	3.777245	2.724852
H	-9.14008	4.574633	2.359669
H	-10.736	3.874163	2.162234
C	-10.0781	3.991174	4.213779
H	-10.7359	3.192154	4.578639
H	-9.14066	3.893193	4.775698
C	-10.7127	5.348749	4.525396
H	-11.6493	5.445586	3.963639
H	-10.0546	6.145591	4.159523
C	-10.9859	5.553803	6.01644
H	-11.4384	6.529693	6.210748
H	-10.0607	5.493806	6.597571
H	-11.667	4.788159	6.400572
C	-6.38021	-2.98146	-0.09469

H	-5.45756	-2.85154	-0.66904
H	-7.05019	-3.59914	-0.6998
C	-6.04375	-3.72906	1.205928
H	-6.96826	-3.90198	1.768173
H	-5.41308	-3.08984	1.831936
C	-5.33183	-5.0572	0.947759
H	-5.95645	-5.688	0.303045
H	-4.40984	-4.86614	0.38469
C	-4.99292	-5.81862	2.230364
H	-4.37142	-5.18384	2.874462
H	-5.91526	-6.01186	2.792682
C	-4.26922	-7.14347	1.977851
H	-3.34827	-6.94842	1.415833
H	-4.89061	-7.77581	1.332914
C	-3.93383	-7.8974	3.265863
H	-3.41797	-8.83824	3.057113
H	-4.8407	-8.13291	3.831265
H	-3.28681	-7.29913	3.914704
C	-8.23699	0.88295	0.634179
C	-8.9956	-0.22546	0.249509
C	-6.86149	0.714221	0.775155
H	-6.24472	1.556535	1.065699
C	-6.24189	-0.52271	0.529612
C	-8.39472	-1.4602	0.01633
H	-9.00772	-2.30083	-0.28968
C	-7.01865	-1.64031	0.146719
C	-4.82945	-0.61087	0.589431
C	-3.61529	-0.65434	0.549315
C	-2.26684	-0.66919	0.442869
C	-1.06022	-0.64212	0.300114
H	-10.0681	-0.12131	0.124007
C	12.86543	-1.6528	-0.4398
H	13.74257	-1.70815	-1.0931
H	12.3895	-2.63831	-0.46981
C	13.32431	-1.35716	0.997723
H	12.44313	-1.29506	1.646548
H	13.79279	-0.36656	1.025986
C	14.29607	-2.40361	1.544796
H	13.82156	-3.39242	1.509839
H	15.17271	-2.46367	0.887417
C	14.75526	-2.11337	2.975104

H	15.22985	-1.12454	3.009838
H	13.87833	-2.05217	3.631862
C	15.72714	-3.15879	3.52777
H	16.60257	-3.21935	2.870332
H	15.25154	-4.1461	3.49273
C	16.18015	-2.85894	4.957771
H	16.87242	-3.61991	5.327335
H	15.32651	-2.82457	5.641576
H	16.68737	-1.89096	5.014758
C	9.173953	2.486514	-2.31557
H	8.293553	2.033321	-2.78181
H	9.661455	3.098351	-3.08063
C	8.711684	3.389004	-1.15822
H	9.583602	3.896228	-0.72943
H	8.293924	2.76332	-0.36349
C	7.669089	4.419417	-1.59249
H	8.082257	5.04501	-2.39406
H	6.808421	3.895295	-2.02632
C	7.190141	5.312772	-0.44671
H	6.783683	4.683602	0.355042
H	8.049146	5.840607	-0.01324
C	6.132118	6.334063	-0.87127
H	5.275609	5.804471	-1.30504
H	6.538844	6.962428	-1.67261
C	5.656137	7.218824	0.282285
H	4.901402	7.937507	-0.04772
H	6.488247	7.783607	0.713848
H	5.215768	6.616962	1.083075
C	11.90877	-0.61106	-0.96443
C	12.3769	0.545589	-1.5948
C	10.53573	-0.75002	-0.78935
H	10.139	-1.63697	-0.3086
C	9.636369	0.237301	-1.22717
C	11.4959	1.528369	-2.03537
H	11.88401	2.413098	-2.52888
C	10.11897	1.403384	-1.86668
H	13.44349	0.673892	-1.74764
C	0.337913	-0.5572	0.106597
C	3.11569	-0.36411	-0.29272
C	0.894163	0.593382	-0.48692
C	1.18999	-1.60792	0.495408

C	2.555314	-1.51293	0.299281
C	2.259848	0.685291	-0.68194
H	0.235906	1.397157	-0.79103
H	0.764721	-2.49276	0.951955
H	3.207967	-2.3224	0.600311
H	2.685542	1.56986	-1.13837
C	4.511857	-0.26475	-0.49236
C	5.712034	-0.17343	-0.66449
C	7.047075	-0.05938	-0.85653
C	8.245051	0.059595	-1.02647
C	-6.60632	-1.29744	-3.1213
C	-7.35165	-0.10174	-2.80954
H	-8.42997	-0.12588	-2.88399
C	-6.72171	1.017972	-2.3956
H	-7.29085	1.90127	-2.141
C	-5.16503	-1.23546	-3.06415
H	-4.59538	-2.11813	-3.3249
C	-4.53445	-0.11641	-2.64867
H	-3.45573	-0.09451	-2.56555
C	-5.2855	1.052305	-2.25432
C	-4.65058	2.15615	-1.71608
C	-7.25387	-2.47998	-3.42915
C	-3.23783	2.222287	-1.57514
C	-5.39441	3.263847	-1.22587
C	-6.53746	-3.67394	-3.7136
C	-8.67137	-2.57505	-3.40452
N	-2.08825	2.279006	-1.47363
N	-6.0248	4.138259	-0.80782
N	-9.82452	-2.64036	-3.35333
N	-5.94381	-4.64152	-3.93216

P6*p*PP6-TCNQ-8

Electronic Energy (EE): -2622.053762 Hartree

EE + Thermal Free Energy Correction: -2621.072067 Hartree

E (Thermal): 741.434 kcal/mol

Entropy (S): 422.618 cal/mol-kelvin

Imaginary frequencies: 0

C	8.925462	-1.69635	-1.31604
H	9.1652	-2.15347	-2.28438
H	8.408358	-2.4783	-0.74934
C	10.22773	-1.33968	-0.59679
H	9.994378	-0.77626	0.311561
H	10.83309	-0.67079	-1.21786
C	11.0542	-2.5725	-0.22611
H	10.41915	-3.28563	0.310164
H	11.38483	-3.08099	-1.13987
C	12.26562	-2.24162	0.646317
H	12.92791	-1.54825	0.112205
H	11.92386	-1.71001	1.543718
C	13.05727	-3.47802	1.076775
H	13.39453	-4.01919	0.184796
H	12.3865	-4.15646	1.61522
C	14.26069	-3.13986	1.958021
H	14.80678	-4.03974	2.252332
H	13.94507	-2.62899	2.872865
H	14.96087	-2.48085	1.435214
C	5.065896	2.592684	-2.39063
H	4.486509	2.265075	-3.26056
H	5.589746	3.505189	-2.68439
C	4.078486	2.908558	-1.24466
H	4.529909	3.639296	-0.56858
H	3.886478	2.002219	-0.66498
C	2.739547	3.44232	-1.75636
H	2.8936	4.406229	-2.25654
H	2.356056	2.753716	-2.51864
C	1.688409	3.590398	-0.65602
H	1.520807	2.609431	-0.19349
H	2.069626	4.245101	0.137584
C	0.354462	4.139444	-1.16768
H	0.014289	3.522662	-2.00869
H	0.510416	5.143338	-1.57887
C	-0.73004	4.187952	-0.09002
H	-1.66985	4.585246	-0.48295
H	-0.42434	4.824003	0.74597
H	-0.93102	3.19067	0.313434

C	7.95571	-0.55694	-1.54558
C	8.361473	0.771775	-1.69161
C	6.592456	-0.83546	-1.65046
H	6.23888	-1.85257	-1.52898
C	5.650984	0.171319	-1.91029
C	7.433537	1.780271	-1.95188
H	7.776617	2.802103	-2.07336
C	6.071842	1.514114	-2.07562
C	4.271291	-0.14415	-1.97629
C	3.067204	-0.3092	-1.98117
C	1.719494	-0.42135	-1.9421
C	0.506714	-0.47702	-1.87805
H	9.40984	1.030286	-1.6086
C	-13.2569	1.553375	-0.5876
H	-14.2414	1.434984	-1.05239
H	-12.7659	2.386154	-1.10138
C	-13.4394	1.912397	0.896382
H	-12.4522	2.024594	1.35926
H	-13.9221	1.072767	1.409788
C	-14.2579	3.186631	1.108759
H	-13.7705	4.02143	0.589477
H	-15.2419	3.068468	0.637559
C	-14.4436	3.547893	2.583823
H	-14.9304	2.712642	3.103105
H	-13.4596	3.665843	3.054866
C	-15.2627	4.822587	2.800968
H	-16.2454	4.70351	2.329281
H	-14.7752	5.656246	2.281861
C	-15.4431	5.174521	4.278675
H	-16.0306	6.087633	4.404918
H	-14.4761	5.330215	4.76663
H	-15.9571	4.370646	4.814486
C	-10.1261	-3.36174	-1.15297
H	-9.34077	-3.23397	-1.90445
H	-10.772	-4.17371	-1.50057
C	-9.47418	-3.76391	0.18147
H	-10.2605	-3.95032	0.922006
H	-8.88541	-2.92189	0.558346
C	-8.57549	-4.99412	0.053597
H	-9.16148	-5.83867	-0.33097
H	-7.79981	-4.79358	-0.69595

C	-7.91127	-5.39547	1.371922
H	-7.33056	-4.54739	1.75587
H	-8.68576	-5.59835	2.122494
C	-6.99739	-6.61697	1.247231
H	-6.2252	-6.41248	0.496104
H	-7.57836	-7.46399	0.863309
C	-6.33476	-7.00735	2.569566
H	-5.68797	-7.88082	2.4523
H	-7.08485	-7.24758	3.329328
H	-5.722	-6.1885	2.958746
C	-12.4517	0.292419	-0.7807
C	-13.0674	-0.96278	-0.78239
C	-11.0678	0.341846	-0.91282
H	-10.5593	1.299174	-0.92049
C	-10.3007	-0.82869	-1.04317
C	-12.3187	-2.12817	-0.91363
H	-12.8213	-3.08957	-0.91991
C	-10.9326	-2.09445	-1.04656
H	-14.1463	-1.0265	-0.68645
C	-0.90326	-0.51642	-1.78521
C	-3.71243	-0.57706	-1.57815
C	-1.5963	-1.74193	-1.80268
C	-1.64002	0.677913	-1.66329
C	-3.01804	0.648537	-1.56097
C	-2.97488	-1.77111	-1.70135
H	-1.0362	-2.6637	-1.89502
H	-1.11359	1.622653	-1.64815
H	-3.57666	1.570829	-1.46533
H	-3.50232	-2.71635	-1.7151
C	-5.12174	-0.60919	-1.47267
C	-6.33378	-0.63966	-1.38066
C	-7.68251	-0.68329	-1.2753
C	-8.89295	-0.73486	-1.17325
C	7.96833	2.365312	1.519625
C	8.977908	1.552138	2.156299
H	9.905819	2.017772	2.462905
C	8.785252	0.229188	2.340252
H	9.555384	-0.37668	2.800091
C	6.711503	1.7437	1.178073
H	5.925767	2.352404	0.754594
C	6.51993	0.418345	1.3582

H	5.582371	-0.04002	1.074829
C	7.567245	-0.41244	1.900077
C	7.431626	-1.78671	1.974054
C	8.215452	3.695872	1.232737
C	6.272137	-2.44884	1.489411
C	8.473357	-2.60906	2.483441
C	7.267191	4.502937	0.5487
C	9.451358	4.31553	1.565093
N	5.33415	-2.97818	1.069453
N	9.338841	-3.26204	2.884866
N	10.46146	4.80704	1.838061
N	6.501052	5.15046	-0.02577

7.0 AAA-TCNQ coordinates

AAA

Electronic Energy (EE): -4987.902028 Hartree

EE + Thermal Free Energy Correction: -4986.750300 Hartree

E (Thermal): 825.631 kcal/mol

Entropy (S): 347.152 cal/mol-kelvin

Imaginary frequencies: 0

S	8.763487	1.127214	2.925554
S	7.474415	-0.67164	-3.20536
C	9.855582	-0.34925	2.676265
H	10.89702	-0.0271	2.649155
H	9.70328	-0.92571	3.590826
C	9.48489	-1.12856	1.450159
C	10.2816	-1.08745	0.302054
C	9.797403	-1.56064	-0.9108
H	10.39423	-1.44553	-1.80857
C	8.506645	-2.07292	-1.02351
C	7.748959	-2.23956	0.158573
C	8.256726	-1.77541	1.381645
H	7.625733	-1.82883	2.260414
C	7.891122	-2.27573	-2.37341

H	6.998997	-2.89762	-2.32798
H	8.59612	-2.73597	-3.06777
C	9.129127	2.180172	1.449621
H	9.451526	3.143041	1.852068
H	9.984166	1.737052	0.93427
C	7.977722	2.407271	0.452885
C	8.433474	3.480903	-0.5606
H	9.344739	3.143808	-1.06833
H	8.681876	4.408481	-0.03211
C	7.325681	3.746709	-1.59472
H	7.668371	4.509023	-2.30158
C	7.011738	2.449239	-2.35646
H	6.232015	2.632435	-3.10537
H	7.900502	2.102793	-2.89248
C	6.544439	1.357108	-1.37587
C	7.642106	1.12491	-0.32526
H	7.311552	0.361359	0.378156
H	8.540233	0.752118	-0.81889
C	6.700802	2.90847	1.155562
H	6.917013	3.829998	1.709248
H	6.369463	2.162568	1.883899
C	5.592913	3.163547	0.119779
H	4.687695	3.503019	0.63376
C	6.059113	4.238599	-0.87509
H	6.264022	5.176054	-0.34663
H	5.26938	4.445457	-1.60555
C	5.284493	1.862424	-0.64036
H	4.47598	2.033573	-1.3596
H	4.935152	1.093695	0.057288
C	6.172753	0.059566	-2.11313
H	5.31954	0.251728	-2.76782
H	5.868704	-0.70779	-1.4014
H	11.26263	-0.62733	0.347125
C	6.421896	-2.73403	0.120281
C	5.249579	-3.05506	0.108912
C	3.92439	-3.3256	0.091849
C	-5.42835	-2.91957	-0.02512
C	-6.60194	-2.60283	-0.01749
S	-9.31365	0.980037	-2.93977
S	-7.5399	-0.2648	3.149283
C	-10.2722	-0.56203	-2.56269

H	-11.3357	-0.32639	-2.51467
H	-10.1002	-1.18533	-3.44199
C	-9.79239	-1.21433	-1.30233
C	-10.5248	-1.10102	-0.11617
C	-9.95254	-1.44135	1.101984
H	-10.5026	-1.27019	2.02041
C	-8.63798	-1.89619	1.18048
C	-7.94068	-2.13929	-0.02524
C	-8.53276	-1.79888	-1.25115
H	-7.95114	-1.90715	-2.15849
C	-7.94483	-1.95496	2.506202
H	-7.03588	-2.55261	2.471683
H	-8.59701	-2.36528	3.278893
C	-9.58197	2.025689	-1.43956
H	-10.0987	2.923502	-1.7836
H	-10.2555	1.49261	-0.76582
C	-8.28498	2.414431	-0.71089
C	-8.54337	3.643083	0.180203
H	-9.44265	3.456815	0.782135
H	-8.76529	4.511125	-0.45044
C	-7.39373	3.971428	1.149874
H	-7.7431	4.781176	1.799943
C	-7.0957	2.740377	2.013006
H	-6.33252	2.978652	2.763601
H	-7.99576	2.431997	2.553165
C	-6.60618	1.588793	1.12207
C	-7.70637	1.239983	0.098175
H	-7.33318	0.482336	-0.59189
H	-8.52583	0.781778	0.657261
C	-5.33415	3.364353	-0.29707
H	-4.31023	3.704123	-0.48377
C	-6.09381	4.455429	0.47773
H	-6.30342	5.302372	-0.18406
H	-5.44176	4.838597	1.271795
C	-5.27978	2.02384	0.452845
H	-4.52498	2.10323	1.243912
H	-4.93373	1.234455	-0.22221
C	-6.25331	0.345528	1.970974
H	-5.38704	0.578386	2.593109
H	-5.97339	-0.48683	1.32557
H	-11.5286	-0.69138	-0.14413

H	-7.57122	2.700331	-1.48442
H	-5.77819	3.223285	-1.28261
S	0.958985	-1.42444	3.120735
S	-1.04874	-1.4196	-3.07874
C	1.233667	-3.1782	2.589727
H	2.301686	-3.38428	2.560312
H	0.794764	-3.75306	3.406901
C	0.566003	-3.48722	1.284237
C	1.307967	-3.56724	0.081461
C	0.629649	-3.57976	-1.14741
H	1.212168	-3.55492	-2.06019
C	-0.75067	-3.47624	-1.22149
C	-1.49524	-3.51306	-0.01832
C	-0.81734	-3.53706	1.211097
H	-1.3983	-3.47967	2.123434
C	-1.40355	-3.15383	-2.53119
H	-2.47992	-3.3105	-2.50021
H	-0.99103	-3.75743	-3.34132
C	1.790816	-0.42584	1.803177
H	2.501651	0.213088	2.332208
H	2.36961	-1.10982	1.181856
C	0.876564	0.450816	0.929459
C	1.775295	1.328687	0.031768
H	2.416085	0.687242	-0.5823
H	2.436981	1.942277	0.653214
C	0.915737	2.230922	-0.86996
H	1.572344	2.844254	-1.49566
C	0.022957	1.359519	-1.76824
H	-0.58556	1.994589	-2.42311
H	0.640983	0.727183	-2.41256
C	-0.89392	0.472433	-0.90442
C	-0.02211	-0.39749	0.0152
H	-0.66331	-1.02821	0.6312
H	0.59935	-1.05194	-0.59634
C	-0.01154	1.372965	1.786636
H	0.617014	1.992788	2.43721
H	-0.6493	0.765175	2.435152
C	-0.87622	2.265766	0.882406
H	-1.513	2.904006	1.503705
C	0.033789	3.138772	0.003017
H	0.659026	3.781757	0.632125

H	-0.5708	3.796345	-0.63123
C	-1.76346	1.385167	-0.01316
H	-2.40374	2.014947	-0.64018
H	-2.42529	0.769244	0.604784
C	-1.83988	-0.37797	-1.77015
H	-2.5278	0.28222	-2.30333
H	-2.44242	-1.03449	-1.14167
C	-2.90444	-3.39301	-0.02649
C	-4.10505	-3.20065	-0.02246
C	2.720123	-3.49288	0.089735

AAA-TCNQ-1

Electronic Energy (EE): -5666.727361 Hartree

EE + Thermal Free Energy Correction: -5665.466017 Hartree

E (Thermal): 916.117 kcal/mol

Entropy (S): 419.936 cal/mol-kelvin

Imaginary frequencies: 0

S	8.692991	-3.04124	-3.04881
S	8.682927	0.196526	2.601338
C	9.472584	-1.41857	-3.48192
H	10.52779	-1.57223	-3.71081
H	8.962668	-1.14004	-4.40609
C	9.286733	-0.38975	-2.40727
C	10.35078	-0.02071	-1.57754
C	10.12591	0.700996	-0.41256
H	10.94903	0.898639	0.264684
C	8.839194	1.078245	-0.03547
C	7.783214	0.837194	-0.94454
C	8.019859	0.103833	-2.11896
H	7.175841	-0.16365	-2.74286
C	8.568641	1.563893	1.354331
H	7.596717	2.04671	1.438971
H	9.332903	2.267626	1.688118
C	9.637692	-3.58613	-1.55558
H	10.03434	-4.57297	-1.80468
H	10.48843	-2.91131	-1.43726

C	8.848258	-3.67375	-0.23695
C	9.750437	-4.35152	0.818399
H	10.67167	-3.7702	0.942689
H	10.04198	-5.34988	0.472133
C	9.012899	-4.4581	2.164817
H	9.668418	-4.94021	2.897107
C	8.640936	-3.05194	2.661798
H	8.124265	-3.11837	3.626577
H	9.546107	-2.45773	2.819061
C	7.735227	-2.34672	1.634422
C	8.466362	-2.27986	0.284608
H	7.823223	-1.79306	-0.44783
H	9.365661	-1.67284	0.393504
C	7.564166	-4.51119	-0.39634
H	7.816753	-5.51458	-0.7601
H	6.916954	-4.04553	-1.14498
C	6.827058	-4.6085	0.949635
H	5.910704	-5.19312	0.817812
C	7.734198	-5.29216	1.984776
H	7.986526	-6.3052	1.652476
H	7.211224	-5.38678	2.942698
C	6.459952	-3.19691	1.439047
H	5.900284	-3.25348	2.378487
H	5.813027	-2.71015	0.702955
C	7.311823	-0.94969	2.118956
H	6.680526	-1.04334	3.003648
H	6.715863	-0.44818	1.356849
H	11.35424	-0.35648	-1.81543
C	6.455854	1.223942	-0.64817
C	5.291541	1.484543	-0.41483
C	3.978226	1.668634	-0.16909
C	-5.33476	1.213309	0.869196
C	-6.52837	0.998805	0.95155
S	-9.61847	-2.88352	3.169893
S	-8.34743	-0.11754	-2.53364
C	-10.1979	-1.14383	3.439207
H	-11.2833	-1.13372	3.543217
H	-9.76019	-0.87919	4.403269
C	-9.74028	-0.23243	2.342009
C	-10.6234	0.164085	1.331523
C	-10.1503	0.756959	0.169262

H	-10.8361	0.975107	-0.64136
C	-8.78893	0.981987	-0.02239
C	-7.91169	0.720911	1.055618
C	-8.39882	0.108961	2.221512
H	-7.69504	-0.17279	2.995082
C	-8.26846	1.330358	-1.38152
H	-7.24983	1.712272	-1.34901
H	-8.89858	2.073701	-1.87262
C	-10.3435	-3.31985	1.527164
H	-11.017	-4.1604	1.704912
H	-10.9483	-2.47804	1.184821
C	-9.29971	-3.70082	0.46531
C	-9.99205	-4.46004	-0.68165
H	-10.8747	-3.88767	-0.99673
H	-10.3615	-5.4231	-0.31188
C	-9.10781	-4.67257	-1.92377
H	-9.74552	-5.10908	-2.70057
C	-8.59316	-3.31492	-2.41572
H	-8.02273	-3.43855	-3.34392
H	-9.43509	-2.65313	-2.63852
C	-7.70186	-2.6769	-1.33923
C	-8.52967	-2.47386	-0.05303
H	-7.88121	-2.09797	0.739954
H	-9.25811	-1.68856	-0.2679
C	-6.80131	-5.067	-0.82327
H	-5.89458	-5.66452	-0.96321
C	-7.91888	-5.63032	-1.71743
H	-8.26972	-6.5872	-1.31628
H	-7.50021	-5.8454	-2.70785
C	-6.47264	-3.59553	-1.12528
H	-5.87265	-3.55526	-2.04081
H	-5.85163	-3.18947	-0.32209
C	-7.13559	-1.31823	-1.81691
H	-6.40448	-1.4891	-2.60725
H	-6.61595	-0.81808	-0.99856
H	-11.6821	-0.04702	1.434854
H	-8.5999	-4.38122	0.951837
H	-7.06503	-5.18213	0.228132
S	1.300407	4.309838	2.935916
S	-1.50922	3.279118	-2.86618
C	1.608783	2.507656	2.641255

H	2.673097	2.333202	2.495486
H	1.314144	2.046303	3.584997
C	0.794281	1.991435	1.496269
C	1.386476	1.757676	0.22958
C	0.563293	1.557516	-0.89073
H	1.027851	1.474723	-1.86529
C	-0.81625	1.562531	-0.7949
C	-1.40095	1.63983	0.494
C	-0.57989	1.864832	1.612318
H	-1.04856	2.01785	2.576735
C	-1.65195	1.630308	-2.0353
H	-2.6986	1.408956	-1.83862
H	-1.29457	0.936928	-2.79847
C	1.882583	5.114176	1.374004
H	2.624498	5.850855	1.689761
H	2.406722	4.359433	0.785511
C	0.808176	5.811814	0.520427
C	1.522922	6.577265	-0.61567
H	2.114599	5.875988	-1.21531
H	2.220836	7.307221	-0.19011
C	0.493603	7.294119	-1.50737
H	1.019152	7.829247	-2.30442
C	-0.45897	6.261229	-2.13142
H	-1.18721	6.764644	-2.77829
H	0.103313	5.563071	-2.75845
C	-1.19513	5.480754	-1.02529
C	-0.15382	4.800632	-0.12271
H	-0.66561	4.245651	0.663766
H	0.417869	4.087939	-0.71686
C	-0.01091	6.818049	1.351131
H	0.659752	7.555888	1.807234
H	-0.51672	6.292352	2.166306
C	-1.04343	7.525816	0.45872
H	-1.62628	8.226015	1.065247
C	-0.31537	8.28899	-0.65955
H	0.348679	9.044922	-0.22661
H	-1.03851	8.817566	-1.29006
C	-1.99183	6.484554	-0.16204
H	-2.74643	6.987	-0.77779
H	-2.52635	5.945925	0.628884
C	-2.18786	4.461391	-1.6131

H	-2.98976	4.99504	-2.12785
H	-2.64841	3.874357	-0.81683
C	-2.79886	1.536172	0.64971
C	-4.00367	1.408476	0.758168
C	2.783495	1.754765	0.04811
C	1.526359	-1.5084	0.038719
C	0.954187	-1.71069	-1.27065
H	1.620222	-1.79702	-2.12009
C	-0.38552	-1.77578	-1.44339
H	-0.80187	-1.91598	-2.43295
C	0.624322	-1.41395	1.157808
H	1.037929	-1.25891	2.145351
C	-0.71358	-1.50949	0.989022
H	-1.3785	-1.43276	1.839392
C	-1.28738	-1.6788	-0.32083
C	-2.66145	-1.75248	-0.49261
C	2.896181	-1.39516	0.219122
C	-3.55708	-1.80522	0.608665
C	-3.24347	-1.80103	-1.78682
C	3.442835	-1.14264	1.506561
C	3.814748	-1.50658	-0.85816
N	-4.29071	-1.88977	1.498372
N	-3.70808	-1.81923	-2.84562
N	4.56624	-1.59866	-1.73211
N	3.855854	-0.92781	2.564626

AAA-TCNQ-2

Electronic Energy (EE): -5666.726425 Hartree

EE + Thermal Free Energy Correction: -5665.465090 Hartree

E (Thermal): 916.377 kcal/mol

Entropy (S): 420.829 cal/mol-kelvin

Imaginary frequencies: 0

S	-10.076	1.390623	0.897375
S	-6.06738	-3.50255	-0.71915
C	-10.6885	0.889844	-0.77767
H	-11.6698	0.423754	-0.68189
H	-10.8155	1.845997	-1.28894
C	-9.71892	-0.00452	-1.49089

C	-9.97808	-1.36972	-1.64407
C	-8.96967	-2.24548	-2.0282
H	-9.16632	-3.31127	-2.05864
C	-7.67273	-1.79311	-2.25852
C	-7.44247	-0.39771	-2.23422
C	-8.46927	0.480773	-1.86114
H	-8.23392	1.530114	-1.73614
C	-6.53493	-2.763	-2.35635
H	-5.65313	-2.31369	-2.80916
H	-6.81313	-3.63961	-2.94385
C	-9.98042	-0.2124	1.813851
H	-10.609	-0.0816	2.69754
H	-10.4471	-0.97804	1.189984
C	-8.5795	-0.6764	2.25337
C	-8.75635	-1.88642	3.198037
H	-9.29927	-2.68356	2.676486
H	-9.3625	-1.59491	4.063571
C	-7.38678	-2.40593	3.667896
H	-7.53526	-3.26066	4.335625
C	-6.56024	-2.84725	2.450209
H	-5.58512	-3.22898	2.774748
H	-7.06894	-3.6607	1.923807
C	-6.35684	-1.66208	1.4881
C	-7.72871	-1.12292	1.053192
H	-7.58635	-0.27216	0.387866
H	-8.2599	-1.8956	0.495783
C	-7.81925	0.430395	3.010658
H	-8.40509	0.759493	3.877356
H	-7.69346	1.297438	2.355586
C	-6.44693	-0.08907	3.470312
H	-5.90919	0.708638	3.989514
C	-6.64168	-1.28692	4.413836
H	-7.20951	-0.97799	5.298331
H	-5.67059	-1.65233	4.764813
C	-5.62594	-0.53305	2.248099
H	-4.63644	-0.87687	2.566349
H	-5.47623	0.315957	1.571699
C	-5.4841	-2.06391	0.288026
H	-4.49376	-2.34684	0.650368
H	-5.35651	-1.22046	-0.38746
H	-10.9609	-1.75774	-1.39961

C	-6.12845	0.109982	-2.37619
C	-4.97756	0.493229	-2.31855
C	-3.70613	0.827483	-2.00513
C	4.831964	1.705034	1.784795
C	5.971052	1.573386	2.187016
S	10.73327	1.868362	-0.26184
S	6.826463	-2.32914	2.719503
C	10.7436	2.505024	1.479488
H	11.73053	2.352826	1.917166
H	10.58679	3.577957	1.355487
C	9.668011	1.866135	2.303492
C	9.977055	0.860694	3.225305
C	8.975713	0.066849	3.768438
H	9.238743	-0.7615	4.416466
C	7.641272	0.243301	3.40979
C	7.310855	1.343402	2.584711
C	8.330317	2.142214	2.046254
H	8.06494	2.924336	1.345502
C	6.627483	-0.80362	3.753188
H	5.606857	-0.43773	3.66007
H	6.768055	-1.17483	4.769622
C	10.97027	0.047778	-0.04983
H	11.91075	-0.19661	-0.547
H	11.09373	-0.16081	1.014578
C	9.831266	-0.80106	-0.63824
C	10.32497	-2.24274	-0.85726
H	10.81154	-2.5905	0.063653
H	11.09406	-2.25043	-1.6375
C	9.209369	-3.24562	-1.20201
H	9.66594	-4.24143	-1.21712
C	8.142723	-3.20994	-0.10186
H	7.37813	-3.97362	-0.28913
H	8.594712	-3.43596	0.868359
C	7.488531	-1.82067	-0.05781
C	8.570088	-0.76197	0.243285
H	8.128869	0.23405	0.188553
H	8.880618	-0.91369	1.279863
C	7.652377	-1.7761	-2.65717
H	7.004233	-1.85197	-3.53606
C	8.541417	-3.02919	-2.57395
H	9.300096	-2.9984	-3.36294

H	7.92241	-3.91165	-2.77592
C	6.774226	-1.58141	-1.41086
H	5.939001	-2.2896	-1.46462
H	6.332108	-0.57937	-1.42114
C	6.377129	-1.76782	1.016298
H	5.558687	-2.42161	0.710036
H	5.972262	-0.75918	1.09411
H	11.01413	0.664256	3.474357
H	9.598261	-0.37008	-1.6126
H	8.264007	-0.88954	-2.82383
S	-1.66441	-1.71935	1.247147
S	2.566093	0.647502	-3.11544
C	-2.14231	0.061915	1.1078
H	-3.1277	0.134553	0.654694
H	-2.223	0.378843	2.147834
C	-1.12088	0.866249	0.3642
C	-1.32655	1.2545	-0.98434
C	-0.25462	1.768373	-1.7242
H	-0.40348	1.978351	-2.77637
C	1.015521	1.897122	-1.18068
C	1.188481	1.631857	0.199369
C	0.111313	1.125509	0.943692
H	0.281059	0.84167	1.975191
C	2.187272	2.137165	-2.08119
H	3.073202	2.445354	-1.53099
H	1.96693	2.900108	-2.83019
C	-1.60688	-2.30274	-0.50819
H	-2.27614	-3.16531	-0.54809
H	-2.04842	-1.52469	-1.132
C	-0.22141	-2.70496	-1.04502
C	-0.414	-3.33475	-2.44266
H	-0.90288	-2.61243	-3.10651
H	-1.07537	-4.20532	-2.36971
C	0.944834	-3.75429	-3.03125
H	0.787055	-4.19746	-4.01949
C	1.853136	-2.52134	-3.16614
H	2.819041	-2.81137	-3.59681
H	1.400761	-1.79378	-3.84648
C	2.07252	-1.86894	-1.78772
C	0.706356	-1.48835	-1.19608
H	0.853017	-1.02908	-0.21842

H	0.231956	-0.75106	-1.84378
C	0.462834	-3.73922	-0.13013
H	-0.18009	-4.62058	-0.02098
H	0.598366	-3.31139	0.867493
C	1.823121	-4.14952	-0.717
H	2.302079	-4.87232	-0.0492
C	1.612346	-4.78145	-2.1024
H	0.986173	-5.67585	-2.01498
H	2.572423	-5.09937	-2.52352
C	2.725281	-2.91001	-0.85039
H	3.703871	-3.20336	-1.2468
H	2.897388	-2.46176	0.134946
C	3.014654	-0.65573	-1.88017
H	4.007588	-0.99586	-2.17674
H	3.116638	-0.17437	-0.90705
C	2.460299	1.746321	0.805916
C	3.574721	1.762462	1.290178
C	-2.58633	1.056207	-1.59227
C	-0.72228	4.893731	-1.10069
C	-0.64319	4.52729	0.292086
H	0.299902	4.644322	0.808994
C	-1.71399	4.013523	0.934799
H	-1.6362	3.72087	1.973381
C	-1.99503	4.752339	-1.76909
H	-2.07116	5.043791	-2.80887
C	-3.06496	4.236493	-1.12671
H	-4.00524	4.105797	-1.64577
C	-2.97063	3.818059	0.25235
C	-4.04365	3.221969	0.890735
C	0.396293	5.338609	-1.78257
C	-5.28556	2.999964	0.236117
C	-3.94835	2.778814	2.237898
C	0.346854	5.671894	-3.16292
C	1.666453	5.416141	-1.15033
N	-6.29514	2.822989	-0.29807
N	-3.84561	2.410541	3.328832
N	2.697943	5.448531	-0.62926
N	0.296107	5.924668	-4.29001

AAA-TCNQ-3

Electronic Energy (EE): -5666.724150 Hartree

EE + Thermal Free Energy Correction: -5665.464188 Hartree

E (Thermal): 916.230 kcal/mol

Entropy (S): 423.223 cal/mol-kelvin

Imaginary frequencies: 0

S	9.212996	-0.815	-2.8971
S	6.522516	-2.68486	2.748205
C	10.20411	0.100828	-1.62733
H	11.21483	-0.3072	-1.59514
H	10.26005	1.110579	-2.03865
C	9.554873	0.078178	-0.27588
C	10.06818	-0.71074	0.757848
C	9.316193	-0.95845	1.89913
H	9.694058	-1.63879	2.653906
C	8.029325	-0.4437	2.04344
C	7.561929	0.46216	1.063708
C	8.337106	0.718161	-0.07734
H	7.923135	1.344021	-0.85846
C	7.120781	-0.96566	3.112084
H	6.266237	-0.31862	3.2953
H	7.651193	-1.09523	4.056969
C	9.248713	-2.55994	-2.28478
H	9.662181	-3.14826	-3.10685
H	9.962514	-2.60327	-1.45915
C	7.905133	-3.17236	-1.84933
C	8.134535	-4.67708	-1.58213
H	8.902622	-4.80051	-0.80961
H	8.508733	-5.16215	-2.49085
C	6.825063	-5.3474	-1.13144
H	7.009561	-6.41051	-0.94739
C	6.330191	-4.68526	0.164363
H	5.403625	-5.16571	0.501305
H	7.069463	-4.81349	0.960632
C	6.082091	-3.18313	-0.06907
C	7.38489	-2.52898	-0.55409
H	7.211919	-1.46779	-0.73135
H	8.141468	-2.61374	0.226706
C	6.827015	-3.02719	-2.94134
H	7.175527	-3.48887	-3.87276

H	6.659048	-1.96639	-3.1493
C	5.516584	-3.69059	-2.48535
H	4.751244	-3.56536	-3.25567
C	5.761722	-5.1864	-2.23016
H	6.094401	-5.67403	-3.15277
H	4.829187	-5.67421	-1.92664
C	5.026567	-3.02979	-1.18607
H	4.084567	-3.49697	-0.87721
H	4.824724	-1.96614	-1.36121
C	5.535959	-2.50087	1.196333
H	4.559824	-2.93367	1.42877
H	5.396334	-1.43432	1.016195
H	11.03801	-1.18171	0.640365
C	6.256583	1.009666	1.131595
C	5.110612	1.413493	1.111425
C	3.803807	1.755187	1.04608
C	-5.51308	1.502097	0.140466
C	-6.68799	1.230539	-0.01138
S	-9.96732	-1.99031	2.729012
S	-7.14655	-1.37567	-3.0494
C	-10.8025	-0.47924	2.052061
H	-11.8489	-0.70407	1.843963
H	-10.7639	0.223756	2.886073
C	-10.0965	0.041382	0.837726
C	-10.6204	-0.17575	-0.44096
C	-9.84034	0.033898	-1.57002
H	-10.2329	-0.21762	-2.54869
C	-8.5168	0.456223	-1.46511
C	-8.02722	0.80432	-0.18483
C	-8.8281	0.597786	0.948589
H	-8.40436	0.784225	1.927822
C	-7.60831	0.373774	-2.65237
H	-6.70276	0.963432	-2.52268
H	-8.1107	0.711865	-3.56008
C	-10.0051	-3.17179	1.307539
H	-10.6024	-4.02383	1.637192
H	-10.5344	-2.69552	0.480034
C	-8.61467	-3.64875	0.856315
C	-8.75432	-4.95399	0.051905
H	-9.52943	-4.81339	-0.71311
H	-9.10964	-5.75328	0.711833

C	-7.46516	-5.39164	-0.66647
H	-7.72202	-6.25634	-1.28844
C	-6.984	-4.25559	-1.57578
H	-6.1104	-4.57616	-2.15544
H	-7.76759	-3.98901	-2.29136
C	-6.61953	-3.02832	-0.72643
C	-7.86782	-2.56769	0.055013
H	-7.59513	-1.75261	0.726536
H	-8.5642	-2.1534	-0.67826
C	-5.667	-4.67369	1.052605
H	-4.70351	-5.01067	1.449108
C	-6.31629	-5.82526	0.264784
H	-6.66292	-6.59953	0.957535
H	-5.55002	-6.29421	-0.36398
C	-5.4387	-3.41475	0.199588
H	-4.5635	-3.57686	-0.43846
H	-5.19306	-2.56677	0.849017
C	-6.09094	-1.88077	-1.6172
H	-5.13676	-2.17595	-2.05279
H	-5.90438	-0.99159	-1.01618
H	-11.6271	-0.56488	-0.54706
H	-8.05462	-3.86957	1.765799
H	-6.27136	-4.42923	1.926536
S	0.643991	5.229472	2.668494
S	-0.92459	2.69364	-3.11977
C	0.943669	3.420927	2.924271
H	2.010232	3.242589	3.053382
H	0.43993	3.219611	3.869877
C	0.372582	2.604854	1.806431
C	1.207575	2.05283	0.806737
C	0.633109	1.563501	-0.37611
H	1.288661	1.23352	-1.17269
C	-0.73758	1.573244	-0.58466
C	-1.57709	1.963912	0.487507
C	-1.0018	2.484845	1.657362
H	-1.65368	2.86962	2.432073
C	-1.28518	1.303583	-1.95166
H	-2.35321	1.098465	-1.9353
H	-0.78468	0.456691	-2.42331
C	1.570376	5.61661	1.114232
H	2.259543	6.420767	1.381099

H	2.173621	4.743514	0.858735
C	0.719169	6.054068	-0.09123
C	1.673715	6.521578	-1.21254
H	2.353971	5.704925	-1.48047
H	2.291552	7.352001	-0.85237
C	0.869852	6.963474	-2.44861
H	1.562935	7.289849	-3.23027
C	0.033926	5.78379	-2.9709
H	-0.5316	6.088583	-3.85945
H	0.690954	4.96227	-3.2711
C	-0.93545	5.294092	-1.87761
C	-0.12144	4.89061	-0.63867
H	-0.79965	4.5433	0.140956
H	0.537606	4.06279	-0.90062
C	-0.22088	7.220489	0.270285
H	0.365288	8.065559	0.650445
H	-0.898	6.909438	1.071086
C	-1.02838	7.652764	-0.96445
H	-1.70118	8.47067	-0.68832
C	-0.06505	8.122304	-2.06645
H	0.518859	8.979331	-1.71367
H	-0.62807	8.455015	-2.9452
C	-1.86107	6.465433	-1.47994
H	-2.45942	6.775965	-2.34401
H	-2.56096	6.133822	-0.70436
C	-1.816	4.135076	-2.37715
H	-2.48049	4.499284	-3.16371
H	-2.4455	3.757983	-1.56942
C	-2.98218	1.860179	0.378193
C	-4.18579	1.721789	0.276493
C	2.610321	1.972023	0.963484
C	0.422806	-1.65574	0.243495
C	-0.54519	-1.17988	1.203874
H	-1.5962	-1.21752	0.948691
C	-0.15076	-0.67522	2.392726
H	-0.88303	-0.30562	3.097751
C	1.819978	-1.60849	0.603601
H	2.555528	-1.97338	-0.10101
C	2.213241	-1.10159	1.790759
H	3.262698	-1.0573	2.044473
C	1.249369	-0.58428	2.731858

C	1.66461	0.013975	3.907062
C	0.027281	-2.1415	-0.99069
C	3.042101	0.160021	4.223191
C	0.731118	0.563907	4.825982
C	0.974421	-2.60356	-1.94555
C	-1.34079	-2.18299	-1.37322
N	4.165679	0.282764	4.463581
N	-0.04585	1.026636	5.546214
N	-2.45491	-2.19954	-1.68078
N	1.759074	-2.97155	-2.71071

AAA-TCNQ-4

Electronic Energy (EE): -5666.722810 Hartree

EE + Thermal Free Energy Correction: -5665.463650 Hartree

E (Thermal): 916.103 kcal/mol

Entropy (S): 424.488 cal/mol-kelvin

Imaginary Frequencies: 0

S	-9.1418	-2.61042	1.396967
S	-6.10644	0.544842	-3.42159
C	-10.3033	-1.21837	1.012246
H	-11.2002	-1.61579	0.536183
H	-10.5808	-0.84833	2.001255
C	-9.65398	-0.15847	0.173531
C	-9.98408	-0.00175	-1.1761
C	-9.18282	0.75915	-2.01794
H	-9.40976	0.805319	-3.07701
C	-8.01562	1.364019	-1.55552
C	-7.74986	1.312319	-0.16658
C	-8.58601	0.570433	0.681582
H	-8.31126	0.469048	1.724312
C	-7.00702	1.896704	-2.52702
H	-6.28045	2.56267	-2.06768
H	-7.49257	2.441273	-3.33858
C	-8.78056	-3.34009	-0.26455
H	-9.07046	-4.39028	-0.18781
H	-9.45162	-2.86613	-0.98433

C	-7.32695	-3.25148	-0.76702
C	-7.20637	-4.14444	-2.02271
H	-7.91792	-3.80569	-2.78475
H	-7.47123	-5.1774	-1.76929
C	-5.77486	-4.09215	-2.58381
H	-5.71202	-4.73113	-3.47031
C	-5.42874	-2.6461	-2.97054
H	-4.41456	-2.60008	-3.38461
H	-6.11224	-2.28887	-3.74701
C	-5.52735	-1.72736	-1.73838
C	-6.95001	-1.81382	-1.16069
H	-7.01975	-1.17808	-0.27902
H	-7.66142	-1.4365	-1.89658
C	-6.32148	-3.75408	0.287235
H	-6.56571	-4.7837	0.575049
H	-6.39956	-3.13851	1.188012
C	-4.89081	-3.68999	-0.27287
H	-4.189	-4.03379	0.492658
C	-4.78557	-4.5879	-1.51592
H	-5.0069	-5.62693	-1.24832
H	-3.76438	-4.56656	-1.91198
C	-4.54881	-2.24087	-0.65959
H	-3.51918	-2.18515	-1.03132
H	-4.60965	-1.59208	0.221402
C	-5.12984	-0.28421	-2.08933
H	-4.09447	-0.277	-2.43874
H	-5.177	0.348955	-1.20551
H	-10.8414	-0.52815	-1.58141
C	-6.5362	1.815711	0.357567
C	-5.42085	2.081274	0.761807
C	-4.11494	2.228333	1.066693
C	5.213195	1.982751	0.507044
C	6.381311	1.795065	0.228668
S	8.22258	-1.02469	-3.84956
S	8.035423	-0.99413	2.604472
C	9.304669	0.424422	-3.44137
H	10.33936	0.191446	-3.69408
H	8.952304	1.199911	-4.12377
C	9.161735	0.825711	-2.00485
C	10.15739	0.518686	-1.0714
C	9.905878	0.615962	0.290793

H	10.66166	0.29881	1.000307
C	8.655968	1.005148	0.767706
C	7.690537	1.436052	-0.17
C	7.961638	1.352454	-1.5446
H	7.176597	1.606711	-2.24629
C	8.30476	0.796521	2.208184
H	7.425872	1.365195	2.507065
H	9.131933	1.075476	2.862762
C	8.794446	-2.33104	-2.67376
H	9.185417	-3.14341	-3.28902
H	9.624128	-1.92856	-2.08932
C	7.687545	-2.86236	-1.74681
C	8.087802	-4.24818	-1.20905
H	9.113068	-4.19152	-0.82011
H	8.107937	-4.96752	-2.03516
C	7.187898	-4.76781	-0.07375
H	7.639061	-5.69556	0.295059
C	7.171648	-3.74115	1.063814
H	6.60137	-4.12811	1.9169
H	8.190453	-3.55104	1.415016
C	6.540744	-2.43071	0.5707
C	7.373634	-1.87784	-0.60523
H	6.875662	-1.00168	-1.02125
H	8.323296	-1.53435	-0.18741
C	4.874186	-3.87026	-0.81646
H	3.819136	-4.16446	-0.81321
C	5.740406	-5.09925	-0.488
H	5.7396	-5.79036	-1.33735
H	5.279751	-5.63825	0.348635
C	5.070493	-2.7154	0.178271
H	4.525674	-2.95454	1.098998
H	4.61679	-1.80161	-0.21904
C	6.466399	-1.39506	1.715415
H	5.792642	-1.77413	2.486405
H	6.046116	-0.45877	1.351521
H	11.11695	0.148841	-1.4159
H	6.798377	-2.98349	-2.36678
H	5.075933	-3.52613	-1.8308
S	-1.0698	0.733637	4.61615
S	0.693531	-0.49055	-1.53823
C	-1.35476	2.359345	3.775728

H	-2.42275	2.567533	3.729481
H	-0.89451	3.075925	4.457889
C	-0.72062	2.398524	2.420149
C	-1.50399	2.255976	1.251188
C	-0.87425	1.989828	0.02398
H	-1.49509	1.784323	-0.83914
C	0.497682	1.870978	-0.08394
C	1.287641	2.168795	1.05507
C	0.659	2.420525	2.286779
H	1.277505	2.550052	3.16647
C	1.093856	1.305016	-1.33655
H	2.170684	1.447683	-1.38581
H	0.649842	1.756775	-2.22501
C	-1.95618	-0.48026	3.535908
H	-2.66005	-0.9914	4.196275
H	-2.54462	0.085474	2.811796
C	-1.08439	-1.52669	2.818728
C	-2.02774	-2.54608	2.142154
H	-2.68656	-2.02432	1.441911
H	-2.66713	-3.01886	2.896325
C	-1.21287	-3.61853	1.39865
H	-1.89826	-4.32845	0.925566
C	-0.34784	-2.95199	0.316873
H	0.225629	-3.71186	-0.22726
H	-0.98518	-2.44305	-0.4125
C	0.613467	-1.93315	0.95903
C	-0.21444	-0.88822	1.724073
H	0.455029	-0.15755	2.17913
H	-0.85548	-0.35898	1.018956
C	-0.17167	-2.28091	3.80424
H	-0.77895	-2.75208	4.586137
H	0.499055	-1.57178	4.29834
C	0.646056	-3.34791	3.058043
H	1.298369	-3.86597	3.768012
C	-0.30701	-4.35694	2.397132
H	-0.91393	-4.8556	3.160496
H	0.265456	-5.13506	1.88055
C	1.508843	-2.67532	1.976416
H	2.112108	-3.42881	1.457877
H	2.205849	-1.96643	2.437869
C	1.529754	-1.28136	-0.09052

H	2.183792	-2.04319	-0.51795
H	2.169547	-0.52958	0.374343
C	2.693056	2.141595	0.954139
C	3.89392	2.092005	0.768665
C	-2.91375	2.278216	1.254795
C	-1.49318	5.129815	-0.39207
C	-0.8875	4.755345	-1.64839
H	-1.53288	4.510189	-2.48274
C	0.456538	4.698638	-1.78536
H	0.897929	4.410272	-2.73102
C	-0.61664	5.497785	0.691442
H	-1.05565	5.793816	1.635073
C	0.726342	5.446845	0.55233
H	1.370673	5.701132	1.383725
C	1.332262	5.014378	-0.68209
C	2.708241	4.892495	-0.79055
C	-2.86765	5.120255	-0.21779
C	3.574573	5.29338	0.262718
C	3.321179	4.340401	-1.94673
C	-3.45779	5.525274	1.010488
C	-3.75103	4.675921	-1.23672
N	4.264826	5.646036	1.120173
N	3.807721	3.86126	-2.87992
N	-4.46526	4.295927	-2.06252
N	-3.91069	5.868657	2.017023

AAA-TCNQ-5

Electronic Energy (EE): -5666.721127 Hartree

EE + Thermal Free Energy Correction: -5665.462185 Hartree

E (Thermal): 916.265 kcal/mol

Entropy (S): 425.490 cal/mol-kelvin

Imaginary Frequencies: 0

S	5.955759	2.936616	3.025276
S	6.57367	1.147048	-3.20559
C	7.304895	1.670472	3.081374
H	8.251217	2.156158	3.321384

H	7.019754	1.037164	3.924032
C	7.389422	0.892206	1.802413
C	8.44731	1.067036	0.905001
C	8.368726	0.560658	-0.38718
H	9.1619	0.773902	-1.09445
C	7.239648	-0.13136	-0.82725
C	6.232687	-0.43062	0.11617
C	6.325047	0.085108	1.417572
H	5.498171	-0.06866	2.099043
C	7.032469	-0.39492	-2.28476
H	6.277757	-1.16005	-2.46074
H	7.95736	-0.70417	-2.77439
C	6.505967	4.084571	1.682357
H	6.551502	5.07269	2.145349
H	7.5252	3.804461	1.40705
C	5.622225	4.154084	0.423552
C	6.134251	5.317165	-0.45565
H	7.186497	5.148327	-0.71269
H	6.085371	6.256725	0.106473
C	5.29301	5.4291	-1.73906
H	5.671257	6.257754	-2.34581
C	5.399472	4.121428	-2.54029
H	4.813704	4.197904	-3.46393
H	6.439282	3.941785	-2.83001
C	4.887943	2.939298	-1.69583
C	5.707603	2.858657	-0.39865
H	5.335793	2.033717	0.20717
H	6.750024	2.648318	-0.64207
C	4.144818	4.418442	0.774469
H	4.059211	5.3456	1.353524
H	3.768508	3.606833	1.40421
C	3.306461	4.520305	-0.51033
H	2.258657	4.691386	-0.24436
C	3.822998	5.686069	-1.36863
H	3.731009	6.628179	-0.81736
H	3.218956	5.783341	-2.27718
C	3.417362	3.208889	-1.30715
H	2.795539	3.264615	-2.20794
H	3.041233	2.374956	-0.70705
C	4.935923	1.618579	-2.48337
H	4.254379	1.682867	-3.33452

H	4.596729	0.791215	-1.85952
H	9.310261	1.654171	1.198971
C	5.078168	-1.17452	-0.22486
C	4.042548	-1.77505	-0.43523
C	2.841029	-2.36021	-0.63486
C	-6.43155	-2.95888	-0.9905
C	-7.62468	-2.73405	-0.93134
S	-10.4677	1.456218	-2.65222
S	-8.78115	-1.61512	2.777788
C	-11.3486	-0.16534	-2.83403
H	-12.4243	-0.00588	-2.75525
H	-11.1217	-0.46044	-3.86004
C	-10.863	-1.16972	-1.83409
C	-11.6295	-1.4849	-0.70739
C	-11.0678	-2.1757	0.357854
H	-11.6494	-2.34027	1.257818
C	-9.73042	-2.5661	0.340945
C	-8.99042	-2.36725	-0.8472
C	-9.57207	-1.67645	-1.92126
H	-8.96275	-1.45368	-2.78862
C	-9.06759	-3.02026	1.604215
H	-8.12372	-3.52875	1.41663
H	-9.71261	-3.69189	2.173054
C	-10.8318	1.944341	-0.90722
H	-11.3911	2.879924	-0.96401
H	-11.4894	1.190463	-0.47029
C	-9.57786	2.1359	-0.03801
C	-9.93122	2.995166	1.189817
H	-10.8345	2.582234	1.658017
H	-10.1859	4.009739	0.863879
C	-8.83062	3.043411	2.264848
H	-9.24561	3.579869	3.12528
C	-8.48446	1.613977	2.695866
H	-7.75775	1.630506	3.516946
H	-9.37923	1.104607	3.065807
C	-7.90311	0.839496	1.503648
C	-8.95151	0.791243	0.372377
H	-8.51352	0.319836	-0.50822
H	-9.7565	0.135906	0.713789
C	-6.69975	3.03843	0.794701
H	-5.69303	3.467759	0.77404

C	-7.54331	3.780185	1.846387
H	-7.78365	4.786123	1.486447
H	-6.93728	3.912759	2.750624
C	-6.58693	1.530506	1.070754
H	-5.85849	1.382377	1.876839
H	-6.17914	1.022108	0.191231
C	-7.50088	-0.59413	1.920352
H	-6.66808	-0.5356	2.623664
H	-7.15364	-1.15711	1.054345
H	-12.6549	-1.13762	-0.64358
H	-8.86299	2.690928	-0.64675
H	-7.1086	3.20542	-0.20205
S	-0.25139	-1.88753	2.655201
S	-2.133	-0.3726	-3.39672
C	0.191575	-3.40063	1.681034
H	1.272931	-3.51233	1.651744
H	-0.21145	-4.2093	2.293057
C	-0.42832	-3.39643	0.316427
C	0.325248	-3.06922	-0.83579
C	-0.33359	-2.82124	-2.05014
H	0.252343	-2.49005	-2.89867
C	-1.71601	-2.82374	-2.14257
C	-2.46507	-3.24458	-1.0178
C	-1.79996	-3.54951	0.180668
H	-2.39262	-3.79635	1.052925
C	-2.37957	-2.20791	-3.33692
H	-3.44341	-2.43315	-3.37454
H	-1.92281	-2.54861	-4.26768
C	0.545416	-0.50839	1.716127
H	1.166524	0.016354	2.445935
H	1.215434	-0.95269	0.981767
C	-0.39487	0.504376	1.040895
C	0.478447	1.614137	0.415533
H	1.162243	1.166687	-0.31377
H	1.094819	2.086757	1.189075
C	-0.40442	2.669621	-0.27154
H	0.234159	3.445075	-0.70597
C	-1.22812	2.005645	-1.38676
H	-1.85257	2.754114	-1.88917
H	-0.56189	1.579304	-2.14252
C	-2.11773	0.893275	-0.79904

C	-1.22191	-0.13443	-0.08784
H	-1.84078	-0.93028	0.328227
H	-0.5493	-0.58351	-0.81817
C	-1.35314	1.147043	2.061296
H	-0.77512	1.615642	2.866601
H	-1.97395	0.370059	2.517719
C	-2.2394	2.194711	1.369302
H	-2.92468	2.633039	2.102203
C	-1.35364	3.295926	0.763009
H	-0.77853	3.791985	1.55251
H	-1.97535	4.061565	0.286068
C	-3.05606	1.519499	0.254981
H	-3.71522	2.25229	-0.22333
H	-3.69709	0.741233	0.681412
C	-2.99781	0.236974	-1.87823
H	-3.7271	0.966711	-2.23706
H	-3.55658	-0.59884	-1.4553
C	-3.87924	-3.23534	-1.03904
C	-5.09102	-3.13728	-1.02227
C	1.70635	-2.78162	-0.74484
C	8.740017	-3.02595	-0.40845
C	7.53223	-3.80674	-0.5326
H	7.329379	-4.30616	-1.47125
C	6.643192	-3.87484	0.481539
H	5.718116	-4.42329	0.362884
C	9.02479	-2.41302	0.867161
H	9.95009	-1.86645	0.987061
C	8.140081	-2.48766	1.883711
H	8.35026	-2.00159	2.82641
C	6.878119	-3.16974	1.719782
C	5.89686	-3.09006	2.690819
C	9.584503	-2.84503	-1.48923
C	6.087357	-2.29887	3.856265
C	4.623695	-3.69847	2.526292
C	10.72349	-2.00012	-1.4014
C	9.318921	-3.43582	-2.7542
N	6.263159	-1.6148	4.771582
N	3.587002	-4.18764	2.379571
N	9.085925	-3.91529	-3.78003
N	11.62691	-1.2837	-1.31586

AAA-TCNQ-6

Electronic Energy (EE): -5666.721127 Hartree

EE + Thermal Free Energy Correction: -5665.462192 Hartree

E (Thermal): 916.265 kcal/mol

Entropy (S): 425.503 cal/mol-kelvin

Imaginary Frequencies: 0

S	-5.95693	2.936937	-3.02492
S	-6.57318	1.146689	3.205912
C	-7.30603	1.670741	-3.08086
H	-8.25243	2.156377	-3.32066
H	-7.02097	1.037525	-3.92362
C	-7.39025	0.892373	-1.80195
C	-8.4479	1.067168	-0.90425
C	-8.369	0.560671	0.387864
H	-9.16201	0.773839	1.095341
C	-7.23983	-0.13141	0.827583
C	-6.23311	-0.43061	-0.11611
C	-6.3258	0.085212	-1.41745
H	-5.49911	-0.06853	-2.09916
C	-7.0323	-0.39514	2.285003
H	-6.2776	-1.16035	2.460719
H	-7.9571	-0.70437	2.774832
C	-6.50678	4.084754	-1.68174
H	-6.55246	5.072914	-2.14463
H	-7.52593	3.804608	-1.40617
C	-5.62269	4.154139	-0.42317
C	-6.13448	5.317119	0.456294
H	-7.18666	5.148258	0.713593
H	-6.08574	6.256743	-0.10573
C	-5.29291	5.428897	1.739507
H	-5.671	6.257477	2.346459
C	-5.39916	4.12113	2.540602
H	-4.81315	4.197494	3.464102
H	-6.4389	3.941447	2.830575
C	-4.88786	2.939092	1.695872
C	-5.70785	2.858615	0.398898
H	-5.33621	2.033747	-0.20712

H	-6.75021	2.64826	0.642564
C	-4.14537	4.418541	-0.77445
H	-4.05992	5.345768	-1.35341
H	-3.76922	3.607009	-1.40438
C	-3.30669	4.520249	0.510146
H	-2.25895	4.691361	0.243928
C	-3.82299	5.685909	1.368733
H	-3.73114	6.628086	0.817555
H	-3.21871	5.783064	2.277135
C	-3.41738	3.208733	1.30684
H	-2.79533	3.26436	2.207474
H	-3.04139	2.374873	0.706547
C	-4.93563	1.618292	2.483282
H	-4.25386	1.682499	3.334258
H	-4.59661	0.790983	1.859265
H	-9.31094	1.654307	-1.19796
C	-5.0785	-1.17454	0.224578
C	-4.04283	-1.77503	0.434766
C	-2.84128	-2.36015	0.634374
C	6.431386	-2.95855	0.990529
C	7.62456	-2.73394	0.931455
S	10.46912	1.455251	2.652692
S	8.781188	-1.61482	-2.77755
C	11.3494	-0.16666	2.834319
H	12.42516	-0.0076	2.755561
H	11.12237	-0.46178	3.860296
C	10.86341	-1.17076	1.834274
C	11.62974	-1.48604	0.707506
C	11.06779	-2.1765	-0.35781
H	11.64931	-2.34114	-1.25782
C	9.730308	-2.56646	-0.3409
C	8.990414	-2.36755	0.84731
C	9.572346	-1.67709	1.921428
H	8.963136	-1.45423	2.78885
C	9.06727	-3.02023	-1.6042
H	8.123273	-3.52849	-1.41665
H	9.712084	-3.69195	-2.17319
C	10.8333	1.943429	0.907721
H	11.39302	2.878794	0.964598
H	11.49065	1.18935	0.470688
C	9.579403	2.135551	0.038615

C	9.932968	2.994816	-1.18915
H	10.83607	2.581591	-1.6575
H	10.18803	4.009256	-0.86313
C	8.832251	3.0436	-2.26403
H	9.247317	3.580037	-3.12444
C	8.485527	1.614346	-2.69521
H	7.758728	1.631247	-3.5162
H	9.380076	1.104711	-3.06532
C	7.90405	0.839902	-1.50303
C	8.95255	0.791158	-0.37188
H	8.514507	0.31978	0.508707
H	9.757286	0.135604	-0.71347
C	6.701558	3.039143	-0.79362
H	5.695005	3.468847	-0.77276
C	7.545256	3.780766	-1.84529
H	7.786004	4.786556	-1.48521
H	6.939163	3.913713	-2.74943
C	6.588148	1.531305	-1.06991
H	5.859579	1.383576	-1.87595
H	6.18024	1.022905	-0.19043
C	7.501303	-0.59353	-1.91988
H	6.668474	-0.53464	-2.62313
H	7.153947	-1.15651	-1.05393
H	12.65525	-1.13909	0.643698
H	8.864771	2.690767	0.64746
H	7.110594	3.205816	0.203115
S	0.251212	-1.88799	-2.65584
S	2.132751	-0.37155	3.395681
C	-0.1917	-3.4009	-1.68136
H	-1.27305	-3.51264	-1.65204
H	0.211354	-4.2097	-2.29319
C	0.428168	-3.39636	-0.31675
C	-0.32544	-3.06894	0.835377
C	0.33335	-2.82063	2.049683
H	-0.25262	-2.48928	2.898125
C	1.715774	-2.82302	2.142144
C	2.46489	-3.2441	1.017489
C	1.799823	-3.54933	-0.18092
H	2.392514	-3.79635	-1.05311
C	2.379275	-2.20689	3.336355
H	3.443109	-2.43216	3.374107

H	1.922434	-2.5473	4.267179
C	-0.54571	-0.50872	-1.71703
H	-1.16694	0.015785	-2.44691
H	-1.2156	-0.95294	-0.9825
C	0.394518	0.504296	-1.04209
C	-0.47886	1.614135	-0.41696
H	-1.16263	1.166815	0.312443
H	-1.09526	2.08655	-1.1906
C	0.403953	2.669824	0.269864
H	-0.23467	3.445331	0.704134
C	1.227728	2.006158	1.385207
H	1.852136	2.75479	1.887433
H	0.561555	1.579941	2.14108
C	2.117409	0.893717	0.797719
C	1.221645	-0.1342	0.086769
H	1.840555	-0.93009	-0.32916
H	0.549083	-0.58317	0.817204
C	1.352713	1.146799	-2.06266
H	0.774642	1.615185	-2.86805
H	1.973568	0.369761	-2.51893
C	2.238921	2.194678	-1.37092
H	2.924142	2.632889	-2.10394
C	1.353102	3.295968	-0.76484
H	0.777944	3.791818	-1.55444
H	1.974775	4.06175	-0.28808
C	3.055671	1.519775	-0.25647
H	3.714789	2.252725	0.221663
H	3.696762	0.741459	-0.68273
C	2.997539	0.237714	1.877057
H	3.726768	0.967587	2.235734
H	3.556384	-0.59815	1.454325
C	3.879056	-3.23485	1.038812
C	5.09084	-3.13683	1.022148
C	-1.70657	-2.78148	0.744364
C	-8.74024	-3.02586	0.409082
C	-7.53255	-3.80683	0.53313
H	-7.3297	-4.30628	1.471761
C	-6.64363	-3.8751	-0.48109
H	-5.71865	-4.42374	-0.36254
C	-9.02498	-2.41281	-0.86648
H	-9.95015	-1.86599	-0.98627

C	-8.14037	-2.4876	-1.8831
H	-8.35049	-2.00138	-2.82574
C	-6.87854	-3.16994	-1.71931
C	-5.89737	-3.09046	-2.69046
C	-9.58464	-2.84489	1.489919
C	-6.08784	-2.29921	-3.85587
C	-4.62431	-3.69912	-2.52606
C	-10.7235	-1.99984	1.402174
C	-9.31907	-3.43579	2.754836
N	-6.26362	-1.61509	-4.77115
N	-3.5877	-4.18848	-2.37942
N	-9.08609	-3.91534	3.780628
N	-11.6269	-1.28331	1.316694

AAA-TCNQ-7

Electronic Energy (EE):	-5666.720806	Hartree
EE + Thermal Free Energy Correction:	-5665.461452	Hartree
E (Thermal):	916.236	kcal/mol
Entropy (S):	424.525	cal/mol-kelvin

Imaginary Frequencies: 0

S	9.233639	2.406488	2.645151
S	6.988791	1.71603	-3.42931
C	9.289829	0.576343	2.372851
H	10.32769	0.247801	2.317958
H	8.856254	0.178493	3.292479
C	8.514686	0.166275	1.157527
C	9.175054	-0.22392	-0.01466
C	8.483455	-0.32414	-1.21471
H	9.024725	-0.53836	-2.12927
C	7.120127	-0.04803	-1.29009
C	6.422539	0.204217	-0.08182
C	7.133786	0.310385	1.123788
H	6.597825	0.592561	2.021453
C	6.457057	0.136064	-2.61934
H	5.372376	0.106413	-2.54867
H	6.768774	-0.62768	-3.33307
C	10.09431	3.085963	1.154936

H	10.91008	3.697273	1.546734
H	10.54355	2.245354	0.62171
C	9.243896	3.931547	0.189758
C	10.19365	4.569487	-0.84883
H	10.73906	3.781686	-1.38146
H	10.9388	5.190324	-0.33857
C	9.395768	5.42368	-1.84955
H	10.08548	5.865641	-2.57538
C	8.378533	4.538312	-2.58777
H	7.814987	5.137862	-3.31237
H	8.898791	3.756423	-3.1491
C	7.408602	3.890116	-1.58109
C	8.220086	3.070299	-0.56615
H	7.540361	2.614782	0.153493
H	8.73781	2.265845	-1.08909
C	8.495505	5.058717	0.927248
H	9.211845	5.692307	1.463621
H	7.825064	4.623553	1.674072
C	7.692158	5.904318	-0.07471
H	7.155117	6.690546	0.464963
C	8.652127	6.537492	-1.09474
H	9.367301	7.189861	-0.58208
H	8.095071	7.161774	-1.80167
C	6.67687	5.011606	-0.81041
H	6.084157	5.615841	-1.50655
H	5.978177	4.567557	-0.09211
C	6.350081	3.027023	-2.29008
H	5.719235	3.667694	-2.91022
H	5.700619	2.543673	-1.55885
H	10.24506	-0.39597	0.008088
C	5.023938	0.405761	-0.08021
C	3.813684	0.528268	-0.07158
C	2.464537	0.574771	-0.07477
C	-6.8522	-0.69413	-0.13662
C	-8.05229	-0.88743	-0.15484
S	-12.2023	1.55962	-2.58866
S	-9.95641	0.108135	3.298241
C	-12.2903	-0.29234	-2.58062
H	-13.3345	-0.60616	-2.58456
H	-11.8472	-0.56903	-3.53892
C	-11.5421	-0.87497	-1.42105

C	-12.2265	-1.3546	-0.29943
C	-11.5473	-1.62075	0.881493
H	-12.1018	-1.91041	1.766871
C	-10.1728	-1.41829	0.985383
C	-9.45734	-1.0674	-0.18275
C	-10.1553	-0.80114	-1.37087
H	-9.60198	-0.44763	-2.23236
C	-9.51754	-1.41046	2.331126
H	-8.4345	-1.49418	2.264631
H	-9.88734	-2.22165	2.960356
C	-12.9278	2.025765	-0.95414
H	-13.816	2.620851	-1.17455
H	-13.2561	1.113779	-0.45199
C	-11.9725	2.825442	-0.05342
C	-12.782	3.560912	1.029965
H	-13.4649	2.843909	1.504642
H	-13.4107	4.325451	0.560263
C	-11.9214	4.190266	2.140085
H	-12.6097	4.576213	2.900185
C	-11.045	3.10509	2.775628
H	-10.4824	3.51874	3.621218
H	-11.6707	2.297697	3.167187
C	-10.0715	2.544103	1.727502
C	-10.8816	1.933263	0.564579
H	-10.1998	1.607417	-0.22201
H	-11.3654	1.034873	0.955308
C	-9.85373	4.963812	0.788016
H	-9.13077	5.78508	0.756102
C	-11.0358	5.367106	1.686884
H	-11.644	6.124847	1.181715
H	-10.6404	5.842687	2.592397
C	-9.13659	3.69281	1.272509
H	-8.50597	3.958657	2.129561
H	-8.45988	3.328444	0.492006
C	-9.14585	1.47171	2.348405
H	-8.4753	1.954113	3.061863
H	-8.52402	1.015562	1.577676
H	-13.3045	-1.46617	-0.33775
H	-11.5021	3.573405	-0.69267
H	-10.1902	4.832868	-0.24048
S	-0.30995	-0.6514	-3.66264

S	-2.17433	-3.02412	2.111619
C	-0.24948	0.84448	-2.57144
H	0.783288	1.167823	-2.45598
H	-0.78247	1.597093	-3.15464
C	-0.91353	0.603146	-1.2507
C	-0.15312	0.360279	-0.08229
C	-0.78185	-0.15456	1.063114
H	-0.16552	-0.44114	1.90592
C	-2.1394	-0.43392	1.082057
C	-2.9217	-0.0571	-0.03686
C	-2.29006	0.457571	-1.18049
H	-2.89014	0.65348	-2.06056
C	-2.71887	-1.25612	2.192558
H	-3.80604	-1.20959	2.20763
H	-2.34686	-0.929	3.16479
C	0.665407	-1.92058	-2.7321
H	1.45751	-2.2379	-3.41238
H	1.14754	-1.41857	-1.89306
C	-0.11821	-3.15085	-2.24164
C	0.894113	-4.17243	-1.67743
H	1.451535	-3.71119	-0.85706
H	1.619209	-4.44644	-2.45094
C	0.162252	-5.42398	-1.16332
H	0.896967	-6.13298	-0.76769
C	-0.81321	-5.02571	-0.0436
H	-1.32827	-5.91475	0.339925
H	-0.26442	-4.58026	0.79121
C	-1.84607	-4.01297	-0.57555
C	-1.09798	-2.78403	-1.11557
H	-1.81903	-2.05907	-1.49402
H	-0.54907	-2.31631	-0.29821
C	-0.90048	-3.82142	-3.38719
H	-0.21099	-4.10107	-4.19264
H	-1.61693	-3.10891	-3.80628
C	-1.63818	-5.06642	-2.86839
H	-2.20117	-5.52197	-3.68924
C	-0.61669	-6.07439	-2.3176
H	0.071613	-6.38271	-3.11217
H	-1.12788	-6.97667	-1.96425
C	-2.61358	-4.66077	-1.74949
H	-3.16112	-5.54138	-1.39427

H	-3.35565	-3.95347	-2.13736
C	-2.86936	-3.62553	0.506534
H	-3.4715	-4.50075	0.76105
H	-3.5513	-2.86189	0.12962
C	-4.31936	-0.27221	-0.05904
C	-5.51747	-0.47532	-0.10234
C	1.250449	0.515862	-0.08112
C	8.014507	-2.90572	2.270856
C	6.785619	-2.78325	3.018221
H	6.838637	-2.69694	4.096132
C	5.591184	-2.73835	2.388012
H	4.677227	-2.61617	2.955398
C	7.923278	-3.07041	0.842509
H	8.833357	-3.20891	0.275735
C	6.72861	-3.02268	0.211866
H	6.67566	-3.12323	-0.86361
C	5.507678	-2.81987	0.949183
C	4.294277	-2.67548	0.299126
C	9.243983	-2.82511	2.905534
C	4.215147	-2.67297	-1.11994
C	3.07797	-2.49084	1.009616
C	10.45268	-2.82318	2.160862
C	9.351361	-2.65197	4.310997
N	4.177082	-2.6567	-2.275
N	2.090355	-2.33956	1.590888
N	9.419656	-2.49847	5.455105
N	11.41701	-2.78145	1.523784

AAA-TCNQ-8

Electronic Energy (EE):	-5666.720585	Hartree
EE + Thermal Free Energy Correction:	-5665.462155	Hartree
E (Thermal):	916.282	kcal/mol
Entropy (S):	426.625	cal/mol-kelvin

Imaginary Frequencies: 0

S	-8.75596	0.462349	-3.10584
S	-7.69988	3.311994	2.642311
C	-8.83312	-0.9546	-1.91749
H	-9.85154	-1.33942	-1.8667
H	-8.21692	-1.71348	-2.40148
C	-8.30971	-0.58063	-0.56378
C	-9.17542	-0.37899	0.516205
C	-8.72753	0.241788	1.675831
H	-9.42932	0.471593	2.469497
C	-7.40925	0.679083	1.799215
C	-6.49658	0.340977	0.774015
C	-6.96146	-0.28102	-0.39395
H	-6.2648	-0.4512	-1.2057
C	-7.01723	1.609996	2.903477
H	-5.93643	1.670243	3.023268
H	-7.45652	1.313746	3.856187
C	-9.90608	1.707216	-2.36538
H	-10.6438	1.920421	-3.14199
H	-10.4308	1.221373	-1.53987
C	-9.27501	3.026267	-1.88567
C	-10.4173	3.99041	-1.49431
H	-11.0318	3.5335	-0.70979
H	-11.0703	4.162969	-2.35745
C	-9.84119	5.327647	-0.9964
H	-10.6649	5.995141	-0.72447
C	-8.95779	5.081113	0.237045
H	-8.55358	6.0314	0.605477
H	-9.5542	4.647797	1.045353
C	-7.80175	4.125891	-0.1186
C	-8.38912	2.809394	-0.64948
H	-7.57625	2.132143	-0.9106
H	-8.97514	2.335329	0.138335
C	-8.4309	3.689844	-2.99102
H	-9.05073	3.864044	-3.87856
H	-7.62255	3.015097	-3.2876
C	-7.84906	5.020468	-2.48572
H	-7.24026	5.471625	-3.27542
C	-8.99801	5.970066	-2.10935
H	-9.62061	6.171836	-2.98775
H	-8.59858	6.931584	-1.76913
C	-6.9668	4.765149	-1.25039

H	-6.52908	5.707873	-0.903
H	-6.13599	4.100647	-1.51407
C	-6.87703	3.885263	1.087435
H	-6.37677	4.819588	1.351475
H	-6.10028	3.161801	0.834541
H	-10.2202	-0.65352	0.421575
C	-5.11361	0.626956	0.884649
C	-3.9059	0.755353	0.92945
C	-2.55422	0.794032	0.956341
C	6.664859	-0.71359	-0.02354
C	7.839065	-0.96837	-0.20808
S	12.34687	-0.32251	2.694888
S	9.650968	1.481718	-2.89846
C	12.2373	-1.93015	1.777586
H	13.24103	-2.27407	1.526529
H	11.81753	-2.61362	2.517737
C	11.3725	-1.8121	0.56029
C	11.94351	-1.71647	-0.71301
C	11.17978	-1.31709	-1.80113
H	11.65405	-1.16587	-2.7642
C	9.83064	-1.0002	-1.65894
C	9.217659	-1.22899	-0.40524
C	10.00017	-1.63302	0.687351
H	9.532529	-1.71802	1.660682
C	9.109951	-0.28504	-2.75877
H	8.029033	-0.32201	-2.63788
H	9.36101	-0.69971	-3.73639
C	13.03141	0.840526	1.432287
H	13.98859	1.187478	1.825836
H	13.23284	0.276372	0.519777
C	12.12097	2.042878	1.134501
C	12.94575	3.161504	0.472134
H	13.52177	2.728442	-0.35637
H	13.67675	3.550223	1.189735
C	12.09967	4.313139	-0.1
H	12.78385	4.976571	-0.64048
C	11.079	3.744739	-1.09259
H	10.51948	4.558522	-1.56936
H	11.59251	3.19603	-1.88749
C	10.1086	2.806203	-0.35908
C	10.90936	1.64789	0.271995

H	10.23922	1.022974	0.863823
H	11.27196	1.029336	-0.55239
C	10.20279	4.455166	1.655481
H	9.57447	5.199825	2.154326
C	11.37075	5.169558	0.953508
H	12.08283	5.537137	1.699898
H	10.98031	6.054976	0.4377
C	9.32846	3.639612	0.68838
H	8.684189	4.335239	0.137051
H	8.660161	2.983339	1.256317
C	9.041816	2.244617	-1.3282
H	8.387674	3.059152	-1.6444
H	8.419058	1.507762	-0.82034
H	13.0044	-1.90292	-0.83887
H	11.76751	2.40704	2.099795
H	10.57838	3.812212	2.451529
S	0.025515	-2.66295	2.683457
S	1.918786	-0.34107	-3.12167
C	0.101828	-0.83087	2.960498
H	-0.89875	-0.44882	3.147619
H	0.667229	-0.75444	3.890639
C	0.794839	-0.11814	1.840906
C	0.063018	0.5814	0.852646
C	0.710986	1.003814	-0.31848
H	0.119131	1.460081	-1.10242
C	2.048783	0.72817	-0.55354
C	2.807017	0.136817	0.48524
C	2.1633	-0.26141	1.667911
H	2.739706	-0.78597	2.419994
C	2.611583	0.898329	-1.93188
H	3.698161	0.840709	-1.93876
H	2.315472	1.853379	-2.36902
C	-0.98326	-2.84846	1.145744
H	-1.78644	-3.53741	1.418338
H	-1.43305	-1.87981	0.923289
C	-0.27278	-3.39985	-0.1021
C	-1.34349	-3.63539	-1.19
H	-1.86177	-2.69436	-1.41032
H	-2.09476	-4.34644	-0.82784
C	-0.69755	-4.18654	-2.47185
H	-1.47811	-4.34175	-3.22113

C	0.337954	-3.18069	-3.00072
H	0.795325	-3.56347	-3.92073
H	-0.1514	-2.23512	-3.25234
C	1.428636	-2.92988	-1.94073
C	0.76074	-2.40821	-0.65817
H	1.522692	-2.22406	0.099765
H	0.270738	-1.45881	-0.87433
C	0.427051	-4.73993	0.192763
H	-0.30222	-5.46147	0.580753
H	1.183047	-4.59372	0.969865
C	1.079874	-5.28772	-1.08651
H	1.584536	-6.232	-0.85904
C	-0.00067	-5.51969	-2.1555
H	-0.73256	-6.25236	-1.79848
H	0.449587	-5.93155	-3.06506
C	2.113189	-4.27545	-1.61092
H	2.602127	-4.67219	-2.50789
H	2.895629	-4.11505	-0.86032
C	2.50996	-1.9621	-2.45305
H	3.051167	-2.43338	-3.27642
H	3.23556	-1.75176	-1.66591
C	4.182984	-0.14423	0.316024
C	5.357144	-0.42017	0.162562
C	-1.34015	0.735828	0.947844
C	-6.10796	-3.37686	0.305283
C	-4.76912	-2.96856	0.656461
H	-3.96691	-3.13888	-0.0494
C	-4.51778	-2.35908	1.834091
H	-3.51521	-2.039	2.079755
C	-7.16048	-3.16445	1.271223
H	-8.16486	-3.48034	1.023932
C	-6.90678	-2.56242	2.452481
H	-7.70586	-2.39051	3.160652
C	-5.57907	-2.09878	2.776596
C	-5.33282	-1.3902	3.937881
C	-6.37544	-3.94847	-0.92633
C	-6.37174	-1.10591	4.864353
C	-4.04608	-0.86373	4.230476
C	-7.69622	-4.3186	-1.29835
C	-5.35019	-4.16096	-1.88817
N	-7.23839	-0.86897	5.591853

N	-2.9984	-0.42754	4.448154
N	-4.50561	-4.32481	-2.66042
N	-8.78215	-4.59197	-1.58609

8.0 P6P6P6-TCNQ coordinates

P6P6P6

Electronic Energy (EE): -2415.205311 Hartree

EE + Thermal Free Energy Correction: -2414.013174 Hartree

E (Thermal): 874.031 kcal/mol

Entropy (S): 424.436 cal/mol-kelvin

Imaginary Frequencies: 0

C	-2.16819	1.97279	-1.9361
H	-2.90942	1.670265	-2.68263
H	-1.78461	2.948267	-2.2477
C	-2.87497	2.102514	-0.57197
H	-2.23456	2.665382	0.116269
H	-3.00119	1.108583	-0.13559
C	-4.24822	2.766586	-0.6858
H	-4.14101	3.775209	-1.10496
H	-4.85145	2.196876	-1.40333
C	-4.99722	2.841895	0.645742
H	-5.09082	1.830837	1.061302
H	-4.40432	3.416085	1.368879
C	-6.38986	3.466294	0.527254
H	-6.98382	2.885105	-0.18845
H	-6.30092	4.471913	0.09967
C	-7.12905	3.541286	1.864048
H	-8.12173	3.983769	1.751025
H	-6.57398	4.14735	2.586397
H	-7.256	2.545432	2.298808
C	2.168075	-1.97285	-1.93607
H	2.90929	-1.67034	-2.68262
H	1.784499	-2.94834	-2.24765
C	2.874854	-2.10253	-0.57194
H	2.234488	-2.66546	0.116283
H	3.000988	-1.10859	-0.13555

C	4.24816	-2.76648	-0.68576
H	4.141044	-3.77512	-1.10491
H	4.851347	-2.19673	-1.4033
C	4.997163	-2.84169	0.645783
H	5.090643	-1.83062	1.061339
H	4.404338	-3.41595	1.368921
C	6.389882	-3.46593	0.527304
H	6.983782	-2.88467	-0.18839
H	6.301056	-4.47156	0.099715
C	7.129063	-3.54085	1.864105
H	8.121797	-3.9832	1.751088
H	6.574065	-4.14699	2.586436
H	7.255875	-2.54498	2.298885
C	-1.0464	0.966196	-1.93788
C	-1.33333	-0.42288	-1.92905
C	0.283401	1.357341	-1.93584
H	0.528484	2.412957	-1.94086
C	1.333211	0.422816	-1.92903
C	-0.28352	-1.35741	-1.93587
H	-0.5286	-2.41302	-1.9409
C	1.046282	-0.96626	-1.93787
C	2.683018	0.84252	-1.89568
C	10.49628	-1.17615	-0.21106
H	11.55392	-1.16144	-0.49334
H	10.04178	-2.02132	-0.73879
C	10.37385	-1.40855	1.303758
H	9.315679	-1.36638	1.582466
H	10.86184	-0.58236	1.833614
C	10.96786	-2.74173	1.758043
H	10.47887	-3.55757	1.210846
H	12.02881	-2.7844	1.481318
C	10.82388	-2.98501	3.261395
H	11.33575	-2.18403	3.809533
H	9.765083	-2.91463	3.538555
C	11.37273	-4.33972	3.714832
H	12.43093	-4.41226	3.436926
H	10.8584	-5.13699	3.165095
C	11.21962	-4.57446	5.21858
H	11.61678	-5.54864	5.515521
H	10.16721	-4.53845	5.516189
H	11.7509	-3.80885	5.792252

C	7.84586	3.829699	-1.69117
H	7.111406	3.657744	-2.48437
H	8.577691	4.543263	-2.08195
C	7.129492	4.446099	-0.47703
H	7.868749	4.65995	0.303493
H	6.440125	3.706402	-0.05782
C	6.358029	5.718891	-0.82668
H	7.046358	6.459294	-1.25397
H	5.627772	5.490613	-1.61293
C	5.633251	6.33271	0.372635
H	4.948884	5.588924	0.799636
H	6.362643	6.561992	1.159937
C	4.848262	7.600571	0.027643
H	4.121123	7.369629	-0.75982
H	5.53291	8.343396	-0.39878
C	4.124073	8.203562	1.232673
H	3.571617	9.106318	0.958928
H	4.831953	8.472638	2.022696
H	3.410123	7.491851	1.658425
C	9.83217	0.10607	-0.647
C	10.54559	1.303577	-0.74731
C	8.466037	0.136226	-0.90691
H	7.883278	-0.77554	-0.84132
C	7.809703	1.328632	-1.25586
C	9.906712	2.490225	-1.09697
H	10.48399	3.405507	-1.17475
C	8.538976	2.535395	-1.35691
H	11.61325	1.305626	-0.55396
C	6.413444	1.29376	-1.49348
C	5.212053	1.217747	-1.66434
C	3.876537	1.060878	-1.81261
C	-10.4962	1.17615	-0.211
H	-11.5539	1.161481	-0.49324
H	-10.0417	2.021297	-0.73875
C	-10.3737	1.408551	1.303812
H	-9.31553	1.366368	1.582466
H	-10.8617	0.582368	1.833696
C	-10.9677	2.741739	1.75812
H	-10.4788	3.557571	1.210827
H	-12.0287	2.784386	1.481532
C	-10.8235	2.985087	3.261445

H	-11.3353	2.184136	3.80968
H	-9.76468	2.914698	3.538479
C	-11.3723	4.339821	3.714888
H	-12.4305	4.412382	3.437104
H	-10.858	5.137057	3.165055
C	-11.219	4.574624	5.218607
H	-11.6161	5.548823	5.515554
H	-10.1665	4.538618	5.516089
H	-11.7502	3.80905	5.792377
C	-7.84604	-3.82985	-1.69103
H	-7.11163	-3.65795	-2.48429
H	-8.57791	-4.54342	-2.08172
C	-7.1296	-4.44619	-0.47691
H	-7.86882	-4.66	0.303669
H	-6.4402	-3.70649	-0.05777
C	-6.35817	-5.71901	-0.82655
H	-7.04654	-6.45943	-1.25376
H	-5.62797	-5.49077	-1.61285
C	-5.63332	-6.33278	0.372746
H	-4.94892	-5.58898	0.799664
H	-6.36266	-6.56202	1.160108
C	-4.84837	-7.60066	0.027758
H	-4.12128	-7.36977	-0.75977
H	-5.53305	-8.3435	-0.39858
C	-4.1241	-8.2036	1.232765
H	-3.57167	-9.10638	0.959024
H	-4.83192	-8.47263	2.022854
H	-3.41011	-7.49188	1.65843
C	-9.83219	-0.10611	-0.64695
C	-10.5456	-1.3036	-0.74713
C	-8.46608	-0.13631	-0.90698
H	-7.88329	0.775444	-0.84148
C	-7.8098	-1.32876	-1.25589
C	-9.90682	-2.49029	-1.09678
H	-10.4841	-3.40556	-1.17446
C	-8.53911	-2.53551	-1.35681
C	-6.41355	-1.29393	-1.49356
H	-11.6133	-1.30562	-0.55369
C	-5.21216	-1.21791	-1.66443
C	-3.87665	-1.061	-1.8127
C	-2.68314	-0.84259	-1.89573

P6P6P6-TCNQ-1

Electronic Energy (EE):	-3094.037792	Hartree
EE + Thermal Free Energy Correction:	-3092.735899	Hartree
E (Thermal):	964.897	kcal/mol
Entropy (S):	498.203	cal/mol-kelvin

Imaginary Frequencies: 0

C	1.686586	3.07521	-1.02545
H	2.494209	2.79513	-0.34146
H	1.055152	3.794455	-0.49567
C	2.308174	3.749853	-2.26017
H	1.508231	4.122443	-2.90972
H	2.856505	3.000374	-2.83873
C	3.256924	4.88787	-1.88402
H	2.711285	5.648826	-1.31175
H	4.028505	4.496164	-1.21127
C	3.92382	5.541581	-3.09559
H	4.466687	4.775561	-3.66364
H	3.151019	5.930072	-3.77104
C	4.888078	6.672055	-2.72745
H	5.664623	6.281125	-2.06166
H	4.349111	7.432568	-2.1501
C	5.540546	7.321578	-3.94877
H	6.224216	8.12362	-3.65848
H	4.787542	7.750634	-4.61702
H	6.113026	6.58798	-4.5247
C	-1.43816	-1.7423	-2.21129
H	-2.15789	-1.87892	-1.39951
H	-0.77969	-2.61537	-2.20025
C	-2.22008	-1.69857	-3.53718
H	-1.52269	-1.8007	-4.37584
H	-2.69395	-0.71897	-3.64336
C	-3.29895	-2.77981	-3.59964
H	-2.83924	-3.77131	-3.50067
H	-3.95683	-2.66241	-2.73098
C	-4.13896	-2.72974	-4.87635
H	-4.58037	-1.72999	-4.97608

H	-3.48881	-2.8645	-5.75007
C	-5.25533	-3.77667	-4.90692
H	-5.90163	-3.63605	-4.03438
H	-4.81643	-4.77574	-4.80008
C	-6.09704	-3.71773	-6.18246
H	-6.88626	-4.47421	-6.17709
H	-5.48036	-3.88592	-7.07074
H	-6.57426	-2.73909	-6.2927
C	0.875469	1.851717	-1.36008
C	1.517864	0.637325	-1.71535
C	-0.50729	1.860796	-1.30442
H	-1.0243	2.767359	-1.0129
C	-1.2726	0.707079	-1.56383
C	0.750057	-0.50406	-2.01875
H	1.269064	-1.41835	-2.2805
C	-0.62991	-0.50174	-1.942
C	-2.6704	0.718676	-1.39681
C	-10.7089	2.14492	0.715232
H	-11.7237	2.164631	0.304088
H	-10.2576	3.115177	0.484268
C	-10.7842	1.973465	2.241564
H	-9.76631	1.94586	2.646589
H	-11.2289	0.997798	2.469633
C	-11.5847	3.079435	2.930477
H	-11.1362	4.052486	2.693827
H	-12.6009	3.104042	2.516873
C	-11.6604	2.913701	4.449585
H	-12.1087	1.940465	4.686276
H	-10.644	2.887952	4.862076
C	-12.4589	4.019175	5.144599
H	-13.4742	4.04413	4.731181
H	-12.0097	4.990798	4.907228
C	-12.5281	3.844085	6.662691
H	-13.1031	4.645844	7.133352
H	-11.5271	3.84782	7.104761
H	-13.002	2.894012	6.927781
C	-7.61573	-2.23655	-1.63437
H	-6.74385	-1.8665	-2.18058
H	-8.22813	-2.79505	-2.34942
C	-7.14602	-3.18618	-0.51694
H	-8.02607	-3.59953	-0.0103

H	-6.59442	-2.61215	0.233564
C	-6.2578	-4.317	-1.033
H	-6.75783	-4.83317	-1.86302
H	-5.34103	-3.88366	-1.44546
C	-5.88199	-5.3312	0.047292
H	-5.45867	-4.79618	0.90281
H	-6.7874	-5.83781	0.406056
C	-4.87229	-6.37342	-0.43778
H	-3.97138	-5.85446	-0.78555
H	-5.27947	-6.89857	-1.31054
C	-4.49324	-7.38832	0.641973
H	-3.76616	-8.11714	0.273272
H	-5.37187	-7.94093	0.989064
H	-4.05451	-6.88802	1.510698
C	-9.91572	1.045974	0.05335
C	-10.5279	-0.14767	-0.34457
C	-8.54406	1.168148	-0.13273
H	-8.03578	2.079998	0.158767
C	-7.78527	0.12645	-0.698
C	-9.78771	-1.1835	-0.90398
H	-10.2885	-2.09714	-1.20618
C	-8.41071	-1.0794	-1.0918
H	-11.5993	-0.26316	-0.21702
C	-6.39439	0.307115	-0.87894
C	-5.20053	0.468947	-1.04669
C	-3.87247	0.614896	-1.23146
C	10.24489	-2.97576	0.201096
H	11.31918	-3.05706	0.005791
H	9.749684	-3.69613	-0.45812
C	9.963564	-3.35609	1.66432
H	8.888758	-3.2603	1.85538
H	10.45607	-2.6305	2.322108
C	10.42575	-4.77049	2.016899
H	9.931921	-5.48925	1.350948
H	11.50151	-4.86181	1.819878
C	10.14318	-5.15224	3.471258
H	10.63711	-4.43344	4.137338
H	9.067723	-5.05842	3.667315
C	10.59969	-6.56841	3.830344
H	11.6744	-6.66121	3.633412
H	10.10522	-7.28531	3.16422

C	10.3118	-6.93936	5.286209
H	10.64683	-7.95429	5.515694
H	9.240145	-6.88544	5.50088
H	10.82051	-6.25683	5.973895
C	8.34472	2.468301	-0.81892
H	7.657369	2.493008	-1.67031
H	9.182845	3.127101	-1.06573
C	7.615977	3.007753	0.425077
H	8.323726	3.053251	1.260816
H	6.833137	2.303864	0.720377
C	6.990613	4.383934	0.196772
H	7.763321	5.094738	-0.12459
H	6.27687	4.313927	-0.63317
C	6.275177	4.926177	1.436062
H	5.521892	4.199878	1.758217
H	6.995355	5.006539	2.260597
C	5.612072	6.287634	1.214113
H	4.890986	6.208022	0.39397
H	6.366571	7.012567	0.884934
C	4.90407	6.814684	2.463502
H	4.433072	7.784472	2.280841
H	5.607344	6.93636	3.293106
H	4.124944	6.120359	2.792738
C	9.773665	-1.58092	-0.12663
C	10.62647	-0.47915	-0.0078
C	8.454725	-1.3478	-0.50093
H	7.760964	-2.17498	-0.59841
C	7.985857	-0.04543	-0.74623
C	10.17153	0.81404	-0.2502
H	10.85597	1.649865	-0.15186
C	8.852438	1.065274	-0.61865
C	6.630436	0.139001	-1.10237
H	11.66144	-0.63745	0.277202
C	5.454266	0.285505	-1.37298
C	4.128462	0.424813	-1.57667
C	2.921464	0.535055	-1.69285
C	-1.13933	-0.54982	1.511012
C	-0.37341	-1.72146	1.166464
H	-0.90156	-2.63711	0.933588
C	0.978607	-1.6874	1.127192
H	1.53931	-2.5753	0.863768

C	-0.41383	0.652694	1.829126
H	-0.97373	1.540671	2.09242
C	0.937133	0.689801	1.778632
H	1.464038	1.608262	2.002237
C	1.702745	-0.47541	1.41843
C	3.088018	-0.42638	1.350869
C	-2.52723	-0.57144	1.533256
C	3.79436	0.77567	1.624052
C	3.868896	-1.56337	1.012481
C	-3.28491	0.559372	1.939654
C	-3.25578	-1.72819	1.151503
N	4.342927	1.768564	1.846798
N	4.502352	-2.49215	0.742281
N	-3.8274	-2.67625	0.817711
N	-3.88583	1.482557	2.29072

P6P6P6-TCNQ-2

Electronic Energy (EE):	-3094.037398	Hartree
EE + Thermal Free Energy Correction:	-3092.731556	Hartree
E (Thermal):	964.972	kcal/mol
Entropy (S):	490.145	cal/mol-kelvin

Imaginary Frequencies: 0

C	-1.69648	0.475534	-2.39285
H	-2.33226	-0.40866	-2.28613
H	-1.07319	0.303309	-3.27387
C	-2.62183	1.69389	-2.6206
H	-2.1043	2.441445	-3.23045
H	-2.83137	2.173783	-1.66264
C	-3.95284	1.293431	-3.2655
H	-3.78435	0.955434	-4.29548
H	-4.35396	0.429693	-2.72503
C	-5.00221	2.405514	-3.24751
H	-5.11477	2.765554	-2.21713
H	-4.65355	3.262871	-3.83736
C	-6.36965	1.946884	-3.76141
H	-6.70524	1.094025	-3.16246
H	-6.26764	1.579101	-4.78927
C	-7.42922	3.048346	-3.71293

H	-8.40327	2.680905	-4.04565
H	-7.1512	3.894628	-4.34891
H	-7.55226	3.425269	-2.69355
C	1.696742	0.475183	2.392787
H	2.332603	-0.40893	2.285892
H	1.073477	0.302758	3.273791
C	2.622003	1.693572	2.620715
H	2.104385	2.441038	3.230609
H	2.831569	2.173566	1.662809
C	3.952996	1.293124	3.265642
H	3.784484	0.955054	4.295599
H	4.354173	0.429438	2.725128
C	5.002325	2.405249	3.247767
H	5.1149	2.765369	2.217411
H	4.653616	3.26255	3.837666
C	6.369773	1.946634	3.76166
H	6.705405	1.093825	3.16266
H	6.267754	1.578783	4.789498
C	7.429301	3.048136	3.713272
H	8.403363	2.680708	4.045995
H	7.15124	3.894371	4.349301
H	7.55235	3.425125	2.69392
C	-0.81715	0.561088	-1.17125
C	-1.39004	0.553293	0.128648
C	0.563937	0.574684	-1.26674
H	1.032257	0.561123	-2.24376
C	1.390281	0.553248	-0.12869
C	-0.56369	0.574557	1.266704
H	-1.03202	0.560896	2.243722
C	0.817386	0.560905	1.171208
C	2.7934	0.4775	-0.25712
C	10.67782	2.951085	0.960079
H	11.43569	2.967393	1.750181
H	9.989238	3.775842	1.170817
C	11.35702	3.192899	-0.39783
H	10.59614	3.166515	-1.18633
H	12.04083	2.362303	-0.6077
C	12.11875	4.517512	-0.45939
H	11.42845	5.342914	-0.24419
H	12.87494	4.538562	0.335563
C	12.79642	4.763573	-1.80884

H	13.48644	3.937859	-2.02391
H	12.0401	4.741642	-2.60356
C	13.55956	6.088754	-1.87539
H	14.3146	6.109451	-1.08057
H	12.86891	6.912766	-1.66041
C	14.23331	6.324807	-3.22829
H	14.77041	7.276655	-3.24846
H	13.4964	6.34065	-4.03709
H	14.95257	5.531333	-3.45314
C	7.807367	-2.15525	1.027473
H	6.807033	-2.02851	1.451246
H	8.336815	-2.86065	1.674843
C	7.68271	-2.75521	-0.38447
H	8.685398	-2.90563	-0.80108
H	7.180756	-2.03459	-1.03781
C	6.907417	-4.07251	-0.40226
H	7.403259	-4.80229	0.250384
H	5.916768	-3.9065	0.034729
C	6.747756	-4.65873	-1.80546
H	6.324423	-3.89526	-2.46633
H	7.735197	-4.91086	-2.21256
C	5.849957	-5.89709	-1.8436
H	4.875695	-5.64147	-1.40976
H	6.274624	-6.67801	-1.20123
C	5.64627	-6.44201	-3.25799
H	5.003095	-7.32636	-3.26048
H	6.601041	-6.72345	-3.71293
H	5.182917	-5.68708	-3.89981
C	9.92869	1.643173	1.0071
C	10.55259	0.464279	1.424631
C	8.601959	1.567889	0.597232
H	8.08346	2.463683	0.275782
C	7.899252	0.351411	0.609693
C	9.867545	-0.74696	1.433006
H	10.3737	-1.64584	1.768156
C	8.535826	-0.83767	1.033813
H	11.58578	0.497692	1.754475
C	6.531356	0.34546	0.242497
C	5.345188	0.364794	-0.02203
C	4.006617	0.407555	-0.19552
C	-10.6776	2.95141	-0.95948

H	-11.4352	2.967999	-1.74981
H	-9.98889	3.776212	-1.16973
C	-11.3572	3.192803	0.3983
H	-10.5966	3.16614	1.187021
H	-12.0411	2.36216	0.607689
C	-12.1189	4.517417	0.460049
H	-11.4285	5.342871	0.245358
H	-12.8748	4.538756	-0.33515
C	-12.797	4.763044	1.809361
H	-13.4871	3.937285	2.023913
H	-12.041	4.740807	2.604326
C	-13.5601	6.088234	1.876105
H	-14.3149	6.109241	1.081035
H	-12.8694	6.912291	1.661652
C	-14.2343	6.323844	3.228859
H	-14.7714	7.275709	3.249175
H	-13.4977	6.339373	4.037918
H	-14.9537	5.530324	3.453189
C	-7.80728	-2.155	-1.02758
H	-6.80695	-2.02827	-1.45138
H	-8.33678	-2.86031	-1.67501
C	-7.6826	-2.75514	0.384286
H	-8.68528	-2.90555	0.800913
H	-7.18058	-2.03463	1.037693
C	-6.90739	-4.07249	0.401882
H	-7.40328	-4.80215	-0.25087
H	-5.91673	-3.90648	-0.03507
C	-6.74779	-4.65893	1.805001
H	-6.32442	-3.89559	2.465993
H	-7.73525	-4.91106	2.212045
C	-5.85007	-5.89736	1.84296
H	-4.87577	-5.64172	1.409221
H	-6.27476	-6.67813	1.200428
C	-5.64649	-6.44255	3.257259
H	-5.00336	-7.32693	3.259631
H	-6.60131	-6.72403	3.712093
H	-5.18314	-5.68776	3.899252
C	-9.92846	1.64349	-1.00668
C	-10.5524	0.464664	-1.42433
C	-8.60172	1.568114	-0.59688
H	-8.08318	2.463853	-0.27534

C	-7.89905	0.351613	-0.60951
C	-9.86742	-0.74659	-1.43287
H	-10.3736	-1.64542	-1.76811
C	-8.53568	-0.83739	-1.03374
C	-6.53114	0.345584	-0.24238
H	-11.5856	0.49815	-1.75413
C	-5.34496	0.36489	0.022092
C	-4.00638	0.407637	0.195527
C	-2.79316	0.477568	0.257078
C	1.419753	-2.76566	-0.06782
C	0.73435	-2.79161	1.20003
H	1.322456	-2.79477	2.108537
C	-0.61582	-2.79099	1.264355
H	-1.1159	-2.79224	2.224285
C	0.61574	-2.79091	-1.26487
H	1.115819	-2.79213	-2.2248
C	-0.73444	-2.79153	-1.20055
H	-1.32255	-2.79466	-2.10905
C	-1.41984	-2.76564	0.067305
C	-2.80306	-2.70532	0.13523
C	2.802976	-2.70537	-0.13574
C	-3.60354	-2.64189	-1.03611
C	-3.48551	-2.68761	1.380987
C	3.485427	-2.68756	-1.3815
C	3.603459	-2.6421	1.035611
N	-4.24474	-2.58339	-1.99651
N	-4.01791	-2.68325	2.407085
N	4.244661	-2.58376	1.996021
N	4.017809	-2.6831	-2.40761

P6P6P6-TCNQ-3

Electronic Energy (EE):	-3094.036258	Hartree
EE + Thermal Free Energy Correction:	-3092.732858	Hartree
E (Thermal):	965.025	kcal/mol
Entropy (S):	495.460	cal/mol-kelvin

Imaginary Frequencies: 0

C	1.702273	-2.48481	1.054641

H	2.556184	-2.37441	0.381132
H	1.06635	-3.2759	0.64682
C	2.236726	-2.92347	2.42918
H	1.399516	-3.03522	3.126848
H	2.876332	-2.13101	2.829858
C	3.028905	-4.22789	2.338048
H	2.365814	-5.03711	2.007796
H	3.791528	-4.12698	1.559109
C	3.709983	-4.62448	3.647993
H	4.362349	-3.80537	3.976057
H	2.955277	-4.74748	4.43507
C	4.536001	-5.90777	3.528975
H	5.280818	-5.77944	2.734964
H	3.883967	-6.72729	3.204513
C	5.236365	-6.29356	4.832765
H	5.81855	-7.21178	4.718953
H	4.511307	-6.45616	5.636143
H	5.919612	-5.50385	5.160073
C	-1.34565	2.502168	1.352411
H	-2.14993	2.464759	0.613093
H	-0.72065	3.360265	1.093248
C	-1.99138	2.717971	2.733964
H	-1.21739	2.984759	3.461762
H	-2.43216	1.777353	3.076115
C	-3.07981	3.789815	2.685867
H	-2.65024	4.741921	2.350405
H	-3.81163	3.501828	1.922493
C	-3.80685	3.992697	4.01499
H	-4.22307	3.03305	4.346605
H	-3.08873	4.297396	4.786674
C	-4.93264	5.026916	3.930215
H	-5.63846	4.723832	3.148843
H	-4.51563	5.987636	3.605946
C	-5.67993	5.209776	5.251956
H	-6.47618	5.953501	5.162744
H	-5.00268	5.540315	6.045475
H	-6.13647	4.270133	5.577776
C	0.928423	-1.19653	1.100353
C	1.593646	0.055531	1.176051
C	-0.45775	-1.20173	1.095045
H	-0.98391	-2.14622	1.024756

C	-1.20933	-0.01781	1.157445
C	0.841228	1.239767	1.267657
H	1.367875	2.184773	1.329201
C	-0.54497	1.233649	1.255956
C	-2.61927	-0.05872	1.10141
C	-10.9394	-1.73721	0.736988
H	-11.8736	-1.56447	1.281585
H	-10.4723	-2.61902	1.187502
C	-11.2609	-2.03353	-0.73728
H	-10.3226	-2.19584	-1.27995
H	-11.7219	-1.14663	-1.18707
C	-12.1796	-3.2423	-0.92021
H	-11.7135	-4.12449	-0.46343
H	-13.1148	-3.07411	-0.37136
C	-12.4999	-3.54163	-2.38606
H	-12.9664	-2.65955	-2.84261
H	-11.5642	-3.70789	-2.93467
C	-13.4173	-4.75217	-2.57524
H	-14.3518	-4.58479	-2.02652
H	-12.9499	-5.63263	-2.11856
C	-13.7307	-5.04255	-4.04407
H	-14.3866	-5.91054	-4.15048
H	-12.8155	-5.24502	-4.60879
H	-14.2275	-4.18944	-4.51624
C	-7.42552	2.928192	1.137289
H	-6.53993	2.704789	1.739639
H	-7.94728	3.753918	1.63095
C	-6.97851	3.36984	-0.26819
H	-7.86313	3.663168	-0.84526
H	-6.54041	2.515338	-0.79082
C	-5.97523	4.522508	-0.23542
H	-6.40387	5.369336	0.316192
H	-5.08933	4.212533	0.330575
C	-5.54029	4.99145	-1.62472
H	-5.09734	4.148686	-2.16879
H	-6.42502	5.290104	-2.20136
C	-4.5436	6.151969	-1.58631
H	-3.66432	5.852037	-1.00688
H	-4.99651	6.995462	-1.05103
C	-4.09772	6.603532	-2.97723
H	-3.39697	7.440522	-2.91965

H	-4.95242	6.921978	-3.58264
H	-3.5947	5.789701	-3.50632
C	-10.0313	-0.54359	0.898011
C	-10.5511	0.751121	0.993395
C	-8.64896	-0.69397	0.903781
H	-8.21212	-1.68385	0.837562
C	-7.7914	0.415867	0.997989
C	-9.71199	1.856582	1.088716
H	-10.1411	2.849928	1.165649
C	-8.32549	1.722515	1.092259
H	-11.6269	0.892986	0.997417
C	-6.38982	0.220985	1.008944
C	-5.1814	0.089465	1.028652
C	-3.83413	-0.00881	1.055309
C	11.2507	2.206474	0.686782
H	12.19651	2.07138	1.221892
H	10.7532	3.068828	1.142342
C	11.54553	2.515675	-0.79039
H	10.59625	2.64192	-1.32349
H	12.03614	1.647513	-1.24548
C	12.41486	3.759167	-0.98168
H	11.91945	4.622551	-0.51986
H	13.36123	3.627135	-0.44207
C	12.70913	4.070938	-2.45039
H	13.2045	3.207428	-2.91206
H	11.7625	4.201605	-2.98973
C	13.57786	5.31561	-2.64775
H	14.52334	5.183824	-2.10833
H	13.08174	6.177484	-2.18597
C	13.86556	5.618103	-4.11941
H	14.48683	6.510468	-4.23166
H	12.93784	5.78573	-4.67499
H	14.3898	4.784667	-4.59696
C	7.921864	-2.5868	1.142042
H	7.060347	-2.39018	1.787332
H	8.484399	-3.40454	1.60203
C	7.411906	-3.02736	-0.24343
H	8.247544	-3.44271	-0.8183
H	7.059119	-2.15128	-0.79544
C	6.270137	-4.03841	-0.15246
H	6.551585	-4.86762	0.509704

H	5.416802	-3.54194	0.323509
C	5.8366	-4.58837	-1.51148
H	5.689052	-3.75199	-2.20218
H	6.644046	-5.2011	-1.93206
C	4.549805	-5.41383	-1.44965
H	3.747664	-4.79257	-1.03466
H	4.678406	-6.24482	-0.74562
C	4.121213	-5.95496	-2.81384
H	3.179051	-6.50322	-2.7509
H	4.881163	-6.62604	-3.22585
H	3.981471	-5.13853	-3.52929
C	10.39294	0.977301	0.856862
C	10.96579	-0.29503	0.946635
C	9.005238	1.07254	0.877803
H	8.528564	2.044245	0.816471
C	8.194957	-0.07068	0.981713
C	10.17337	-1.4341	1.053394
H	10.64321	-2.409	1.128213
C	8.783418	-1.35492	1.072769
C	6.784905	0.056809	1.004595
H	12.04643	-0.39299	0.938391
C	5.570211	0.10534	1.033599
C	4.219081	0.124544	1.077342
C	3.004024	0.113693	1.13357
C	0.489241	-1.43697	-2.16813
C	-0.9173	-1.76696	-2.19686
H	-1.20443	-2.80842	-2.2663
C	-1.86058	-0.80247	-2.14303
H	-2.91142	-1.06036	-2.16188
C	0.855457	-0.04842	-2.04244
H	1.90463	0.20968	-1.98355
C	-0.08975	0.916311	-1.98505
H	0.197824	1.954421	-1.88523
C	-1.49201	0.591153	-2.05603
C	-2.45803	1.582593	-2.05415
C	1.454774	-2.42507	-2.25891
C	-2.10719	2.959114	-1.99787
C	-3.8447	1.287626	-2.13853
C	2.8409	-2.11151	-2.26331
C	1.107776	-3.80118	-2.32116
N	-1.80478	4.073901	-1.94977

N	-4.97386	1.054685	-2.22375
N	0.807655	-4.91735	-2.34978
N	3.964241	-1.8386	-2.26547

P6P6P6-TCNQ-4

Electronic Energy (EE):	-3094.033700	Hartree
EE + Thermal Free Energy Correction:	-3092.732044	Hartree
E (Thermal):	964.907	kcal/mol
Entropy (S):	498.736	cal/mol-kelvin

Imaginary Frequencies: 0

C	-1.95665	2.184661	0.032048
H	-2.58523	2.002712	-0.84501
H	-1.44129	3.13209	-0.14607
C	-2.88671	2.309006	1.257819
H	-2.38562	2.88273	2.044517
H	-3.07366	1.316457	1.672733
C	-4.23064	2.946659	0.89433
H	-4.08178	3.994432	0.604941
H	-4.62822	2.439367	0.007793
C	-5.26894	2.859131	2.013626
H	-5.41246	1.803808	2.278107
H	-4.89108	3.353348	2.917569
C	-6.62024	3.466514	1.627357
H	-6.95832	3.012406	0.688519
H	-6.49091	4.533789	1.41392
C	-7.69142	3.284186	2.70417
H	-8.64723	3.718179	2.399039
H	-7.39356	3.761671	3.642411
H	-7.86061	2.224029	2.916246
C	1.956691	-2.18399	0.029833
H	2.585367	-2.00113	-0.84697
H	1.441338	-3.13123	-0.14932
C	2.886633	-2.30963	1.255558
H	2.385376	-2.88398	2.04169
H	3.073733	-1.31749	1.671389
C	4.230468	-2.9472	0.891583

H	4.081432	-3.99467	0.60121
H	4.628226	-2.43915	0.005554
C	5.268687	-2.86092	2.011059
H	5.412382	-1.80587	2.276543
H	4.890636	-3.3559	2.914501
C	6.619897	-3.46821	1.624365
H	6.958209	-3.01325	0.686022
H	6.490362	-4.53525	1.409862
C	7.690972	-3.28719	2.701501
H	8.646721	-3.72111	2.396076
H	7.392874	-3.76552	3.639235
H	7.860394	-2.22728	2.914639
C	-0.94322	1.072514	0.114689
C	-1.3664	-0.28377	0.098902
C	0.417131	1.323235	0.126519
H	0.772598	2.346639	0.110086
C	1.366444	0.284378	0.099083
C	-0.41708	-1.32265	0.125263
H	-0.77255	-2.34604	0.107855
C	0.943268	-1.07192	0.113582
C	2.740662	0.572304	-0.00779
C	10.78102	-1.41399	0.73944
H	11.76508	-1.54884	0.279551
H	10.25359	-2.36826	0.637038
C	10.95399	-1.09812	2.233731
H	9.967204	-0.92329	2.675984
H	11.50655	-0.15751	2.340119
C	11.66682	-2.21101	3.002155
H	11.11849	-3.15129	2.865107
H	12.66266	-2.37307	2.57077
C	11.8013	-1.92123	4.498103
H	12.34844	-0.98047	4.638773
H	10.80345	-1.75931	4.924916
C	12.50616	-3.03643	5.274034
H	13.50307	-3.19772	4.846959
H	11.95817	-3.97527	5.130862
C	12.63117	-2.73925	6.769356
H	13.13711	-3.5507	7.29896
H	11.64627	-2.60545	7.227125
H	13.20217	-1.82174	6.941577
C	7.790907	2.806508	-2.01659

H	6.910337	2.377661	-2.50483
H	8.411741	3.240335	-2.80615
C	7.326559	3.921579	-1.0633
H	8.203351	4.38837	-0.5994
H	6.744807	3.475702	-0.25044
C	6.477666	4.977057	-1.77304
H	7.071155	5.448925	-2.56686
H	5.635489	4.483131	-2.26888
C	5.945693	6.056309	-0.82859
H	5.359907	5.579627	-0.03221
H	6.785141	6.558295	-0.3301
C	5.077	7.101085	-1.53327
H	4.244963	6.593282	-2.03454
H	5.662805	7.580447	-2.32663
C	4.531924	8.168747	-0.58295
H	3.915478	8.900968	-1.11143
H	5.345649	8.711139	-0.09148
H	3.915796	7.718575	0.2017
C	10.01926	-0.33926	0.00428
C	10.67011	0.646295	-0.74261
C	8.632039	-0.28198	0.087596
H	8.092666	-1.03021	0.655336
C	7.900536	0.72986	-0.55571
C	9.952991	1.64846	-1.3922
H	10.48373	2.392147	-1.97686
C	8.563928	1.716946	-1.32181
H	11.75168	0.622701	-0.82574
C	6.492501	0.739878	-0.42637
C	5.284319	0.738086	-0.29093
C	3.94513	0.681851	-0.1447
C	-10.7815	1.412605	0.739754
H	-11.7656	1.547547	0.279843
H	-10.2543	2.367054	0.638031
C	-10.9546	1.095726	2.233822
H	-9.96782	0.920778	2.676049
H	-11.507	0.154956	2.339538
C	-11.6677	2.207991	3.002917
H	-11.1195	3.148463	2.866515
H	-12.6635	2.370148	2.571572
C	-11.8022	1.917205	4.498668
H	-12.3492	0.97628	4.638691

H	-10.8044	1.755146	4.925425
C	-12.5073	3.0318	5.275295
H	-13.5042	3.193267	4.848254
H	-11.9594	3.970801	5.132795
C	-12.6323	2.733601	6.770408
H	-13.1384	3.544647	7.300523
H	-11.6475	2.599593	7.228156
H	-13.2032	1.815917	6.941969
C	-7.79018	-2.80556	-2.0185
H	-6.90964	-2.37626	-2.50638
H	-8.41083	-3.23907	-2.80838
C	-7.32574	-3.92107	-1.06576
H	-8.2025	-4.38827	-0.60223
H	-6.74417	-3.47552	-0.25258
C	-6.47657	-4.97601	-1.77596
H	-7.06986	-5.44752	-2.57015
H	-5.6344	-4.48168	-2.27141
C	-5.94461	-6.05572	-0.83202
H	-5.359	-5.57941	-0.03529
H	-6.78408	-6.55806	-0.33393
C	-5.07571	-7.10003	-1.53713
H	-4.24364	-6.59189	-2.038
H	-5.66135	-7.57902	-2.33083
C	-4.53068	-8.16815	-0.58729
H	-3.91408	-8.90003	-1.11607
H	-5.34444	-8.71088	-0.09625
H	-3.91472	-7.71834	0.197696
C	-10.0195	0.338518	0.003992
C	-10.67	-0.64676	-0.74353
C	-8.63223	0.281525	0.087377
H	-8.09309	1.029561	0.655594
C	-7.90043	-0.72976	-0.55646
C	-9.9526	-1.64837	-1.39364
H	-10.4831	-2.39184	-1.97879
C	-8.56352	-1.71656	-1.32318
C	-6.49241	-0.73953	-0.42701
H	-11.7516	-0.62338	-0.82674
C	-5.28423	-0.73758	-0.29148
C	-3.94506	-0.68125	-0.14518
C	-2.74061	-0.57164	-0.00819
C	1.409531	0.194433	-3.22589

C	0.837416	-1.12802	-3.24338
H	1.502938	-1.9815	-3.24443
C	-0.50268	-1.31117	-3.24378
H	-0.91545	-2.31188	-3.24386
C	0.502583	1.314636	-3.24262
H	0.915352	2.315341	-3.24183
C	-0.83751	1.131482	-3.24237
H	-1.50304	1.98497	-3.2427
C	-1.40963	-0.19099	-3.22604
C	-2.78367	-0.37992	-3.18676
C	2.783581	0.383333	-3.18651
C	-3.68538	0.716382	-3.13577
C	-3.35972	-1.6787	-3.19061
C	3.359609	1.682123	-3.18919
C	3.685292	-0.71301	-3.13656
N	-4.40377	1.621026	-3.08361
N	-3.8187	-2.73966	-3.20449
N	4.403686	-1.6177	-3.08529
N	3.81855	2.743119	-3.20212

P6P6P6-TCNQ-5

Electronic Energy (EE):	-3094.032767	Hartree
EE + Thermal Free Energy Correction:	-3092.730355	Hartree
E (Thermal):	965.091	kcal/mol
Entropy (S):	497.762	cal/mol-kelvin

Imaginary Frequencies: 0

C	3.207885	2.738275	0.162288
H	4.11586	2.604813	0.759003
H	2.6335	3.543891	0.629208
C	3.608597	3.156235	-1.26265
H	2.703784	3.293745	-1.86514
H	4.171771	2.341426	-1.72769
C	4.447992	4.433656	-1.28424
H	3.860115	5.265346	-0.87557
H	5.304983	4.308401	-0.61261
C	4.949249	4.800887	-2.6823
H	5.575694	3.984891	-3.06437

H	4.094842	4.872859	-3.36706
C	5.741938	6.10967	-2.72789
H	6.592653	6.042221	-2.04182
H	5.112303	6.924916	-2.35199
C	6.24481	6.454067	-4.13075
H	6.804135	7.393315	-4.13703
H	5.412518	6.556618	-4.83378
H	6.905264	5.670115	-4.51392
C	0.013843	-2.17438	0.144247
H	-0.75769	-2.10724	0.91745
H	0.63158	-3.04313	0.387469
C	-0.67075	-2.38245	-1.22271
H	0.059148	-2.78675	-1.93286
H	-0.99022	-1.41824	-1.62462
C	-1.88699	-3.30249	-1.12557
H	-1.61006	-4.24397	-0.63387
H	-2.62521	-2.82252	-0.47179
C	-2.53455	-3.6089	-2.47644
H	-2.74022	-2.66506	-2.99686
H	-1.82003	-4.14848	-3.11002
C	-3.82346	-4.42649	-2.35575
H	-4.54274	-3.87426	-1.74049
H	-3.61529	-5.35132	-1.80605
C	-4.45155	-4.77041	-3.70765
H	-5.39078	-5.31769	-3.58944
H	-3.7789	-5.39204	-4.30523
H	-4.65658	-3.868	-4.29358
C	2.396336	1.469786	0.192048
C	3.013605	0.204236	0.036022
C	1.018287	1.509315	0.339251
H	0.523168	2.465329	0.461921
C	0.235739	0.342016	0.336537
C	2.229545	-0.96486	0.039428
H	2.726091	-1.92097	-0.07617
C	0.852897	-0.92534	0.184852
C	-1.1661	0.416757	0.483089
C	-9.41574	2.305707	1.08643
H	-10.3715	2.138012	0.57891
H	-8.93582	3.153945	0.587414
C	-9.68259	2.671	2.556187
H	-8.72348	2.828706	3.062463

H	-10.1548	1.817262	3.055606
C	-10.5605	3.913021	2.713593
H	-10.0835	4.760548	2.205679
H	-11.5167	3.748979	2.200989
C	-10.8261	4.2826	4.174318
H	-11.3026	3.43455	4.682104
H	-9.86943	4.444977	4.686604
C	-11.7033	5.526206	4.338219
H	-12.6587	5.36271	3.825859
H	-11.2261	6.37241	3.829964
C	-11.962	5.887436	5.801937
H	-12.5898	6.777881	5.890187
H	-11.0243	6.087084	6.329379
H	-12.4674	5.070072	6.325348
C	-6.05412	-2.46636	0.614476
H	-5.23347	-2.27204	-0.08143
H	-6.64691	-3.28011	0.184837
C	-5.45949	-2.92145	1.957392
H	-6.27324	-3.13272	2.660014
H	-4.88296	-2.09586	2.386847
C	-4.56175	-4.14981	1.806778
H	-5.14942	-4.98605	1.407592
H	-3.78964	-3.93835	1.059603
C	-3.88755	-4.57574	3.111422
H	-3.31805	-3.72935	3.51564
H	-4.65295	-4.81373	3.860728
C	-2.9524	-5.7756	2.941399
H	-2.19204	-5.53196	2.189744
H	-3.52094	-6.62103	2.536234
C	-2.26937	-6.1937	4.244509
H	-1.60746	-7.05046	4.093739
H	-3.00701	-6.47196	5.003376
H	-1.66829	-5.37564	4.653078
C	-8.54807	1.080279	0.947105
C	-9.10733	-0.20009	0.894779
C	-7.16205	1.191714	0.903888
H	-6.69542	2.169327	0.936512
C	-6.3368	0.055557	0.799271
C	-8.29956	-1.33004	0.789964
H	-8.76085	-2.31019	0.737511
C	-6.91239	-1.23623	0.731894

H	-10.1855	-0.31672	0.918997
C	-4.9337	0.214342	0.741425
C	-3.72273	0.319299	0.676243
C	-2.37741	0.392094	0.592725
C	12.4133	-2.60906	-0.49349
H	13.2952	-2.64376	-1.14176
H	11.7829	-3.4584	-0.77605
C	12.85574	-2.77327	0.969978
H	11.97097	-2.73342	1.615645
H	13.47737	-1.91594	1.253046
C	13.62212	-4.07229	1.222781
H	12.99576	-4.92526	0.932559
H	14.50358	-4.10634	0.570015
C	14.06353	-4.24099	2.677966
H	14.68838	-3.38698	2.968609
H	13.18184	-4.20773	3.330346
C	14.83266	-5.53911	2.935407
H	15.71329	-5.57095	2.282932
H	14.20739	-6.39146	2.644308
C	15.26895	-5.69803	4.392941
H	15.81524	-6.63194	4.548766
H	14.40456	-5.70153	5.063956
H	15.92142	-4.87495	4.700096
C	9.539503	2.438851	-1.23292
H	8.627519	2.281576	-1.81783
H	10.16614	3.134152	-1.79982
C	9.15794	3.078471	0.11294
H	10.06584	3.240349	0.704946
H	8.539137	2.374864	0.678144
C	8.405055	4.398168	-0.05604
H	9.049912	5.125445	-0.56561
H	7.547082	4.23675	-0.71865
C	7.917636	4.987882	1.268887
H	7.233779	4.275856	1.748138
H	8.768827	5.097142	1.952866
C	7.215509	6.339993	1.12003
H	6.369118	6.235145	0.433406
H	7.9029	7.052255	0.648247
C	6.723225	6.906825	2.452638
H	6.229365	7.872984	2.319718
H	7.553374	7.049692	3.151215

H	6.006784	6.228703	2.926411
C	11.66417	-1.32083	-0.7277
C	12.34211	-0.1393	-1.04178
C	10.28143	-1.26324	-0.58753
H	9.724421	-2.16191	-0.3481
C	9.575629	-0.05877	-0.75247
C	11.65397	1.057962	-1.21151
H	12.20246	1.959286	-1.46385
C	10.26966	1.131107	-1.07462
C	8.167745	-0.04609	-0.59376
H	13.42037	-0.15982	-1.16147
C	6.961371	-0.01352	-0.44501
C	5.620373	0.041461	-0.27258
C	4.415006	0.105693	-0.12302
C	-8.16271	-1.71954	-2.52014
C	-6.81621	-2.12229	-2.84469
H	-6.62224	-3.16228	-3.06901
C	-5.80194	-1.23073	-2.82591
H	-4.7898	-1.5528	-3.03316
C	-8.41387	-0.3199	-2.28945
H	-9.42833	0.004105	-2.10338
C	-7.39717	0.570751	-2.26384
H	-7.59186	1.614338	-2.05907
C	-6.03681	0.150047	-2.48278
C	-4.97969	1.031146	-2.33038
C	-9.16969	-2.66309	-2.38929
C	-5.195	2.366113	-1.8946
C	-3.63765	0.638657	-2.57743
C	-10.4723	-2.28933	-1.96595
C	-8.91798	-4.04694	-2.58749
N	-5.4066	3.439125	-1.52019
N	-2.55176	0.307811	-2.79536
N	-8.68186	-5.16838	-2.74295
N	-11.5129	-1.95365	-1.58894

P6P6P6-TCNQ-6

Electronic Energy (EE):	-3094.031708	Hartree
EE + Thermal Free Energy Correction:	-3092.727717	Hartree
E (Thermal):	965.086	kcal/mol

Entropy (S): 494.422 cal/mol-kelvin

Imaginary Frequencies: 0

C	2.135136	-1.03537	-2.25975
H	3.002268	-0.85298	-2.90184
H	1.612549	-1.9052	-2.66385
C	2.636178	-1.36187	-0.8406
H	1.804229	-1.75629	-0.24757
H	2.971307	-0.44385	-0.35084
C	3.78143	-2.37387	-0.84803
H	3.444279	-3.30026	-1.32685
H	4.596032	-1.97668	-1.46653
C	4.317332	-2.68563	0.549872
H	4.617536	-1.75039	1.039409
H	3.510407	-3.10795	1.161849
C	5.503614	-3.65309	0.546161
H	6.319022	-3.22012	-0.04627
H	5.213977	-4.5774	0.03309
C	6.011176	-3.98352	1.950433
H	6.859437	-4.67155	1.920629
H	5.224964	-4.44956	2.551994
H	6.334437	-3.07919	2.474578
C	-1.38235	3.659076	-2.37074
H	-2.10366	3.538386	-3.18582
H	-0.8	4.557624	-2.59273
C	-2.15771	3.853039	-1.05622
H	-1.46366	4.124755	-0.25501
H	-2.60217	2.896887	-0.76845
C	-3.27359	4.893321	-1.1707
H	-2.84188	5.883149	-1.36349
H	-3.89415	4.653027	-2.04303
C	-4.16358	4.950552	0.071678
H	-4.58796	3.953743	0.245154
H	-3.55339	5.180315	0.952097
C	-5.31222	5.9553	-0.03034
H	-5.91486	5.724504	-0.91681
H	-4.90282	6.959573	-0.19154
C	-6.20535	5.95373	1.212234
H	-7.02579	6.669992	1.118184
H	-5.63256	6.218269	2.106401

H	-6.64072	4.964458	1.381558
C	1.231588	0.16905	-2.30742
C	1.763155	1.479725	-2.18345
C	-0.14117	0.038272	-2.44029
H	-0.57419	-0.94921	-2.54262
C	-1.00087	1.152113	-2.44147
C	0.906685	2.593499	-2.22241
H	1.339912	3.583574	-2.14326
C	-0.46864	2.460284	-2.35071
C	-2.39789	0.943277	-2.46414
C	-9.70187	-0.41307	1.430824
H	-10.6687	-0.91884	1.515378
H	-9.91174	0.658563	1.347529
C	-8.87706	-0.65832	2.705208
H	-7.90464	-0.16822	2.594469
H	-8.67974	-1.73259	2.802957
C	-9.55484	-0.13604	3.971845
H	-9.77033	0.932203	3.847631
H	-10.5246	-0.63283	4.102464
C	-8.706	-0.33443	5.229058
H	-8.48008	-1.40159	5.351449
H	-7.74092	0.169238	5.092141
C	-9.37111	0.189438	6.504022
H	-10.3358	-0.31367	6.64014
H	-9.59788	1.254661	6.378599
C	-8.50981	-0.00719	7.752803
H	-9.00699	0.375566	8.64791
H	-7.55222	0.513413	7.655262
H	-8.2939	-1.06715	7.918993
C	-6.78982	-2.25804	-3.30928
H	-6.6633	-1.40039	-3.9782
H	-7.38071	-2.99756	-3.85612
C	-5.39338	-2.83513	-2.99369
H	-5.48848	-3.86793	-2.64503
H	-4.95349	-2.26796	-2.17115
C	-4.44332	-2.75402	-4.19065
H	-4.82468	-3.37067	-5.01372
H	-4.43482	-1.72039	-4.55706
C	-3.00919	-3.16898	-3.85999
H	-2.65161	-2.55382	-3.02473
H	-2.99683	-4.20787	-3.5105

C	-2.04107	-3.01616	-5.03487
H	-2.0335	-1.96859	-5.35765
H	-2.41457	-3.59631	-5.88664
C	-0.61717	-3.46122	-4.69776
H	0.053882	-3.33987	-5.5518
H	-0.5914	-4.51284	-4.3977
H	-0.20499	-2.8792	-3.86944
C	-8.98753	-0.88401	0.188343
C	-9.34366	-2.06446	-0.46728
C	-7.90242	-0.16245	-0.30632
H	-7.60011	0.757107	0.18101
C	-7.1774	-0.61168	-1.41958
C	-8.64443	-2.5053	-1.59061
H	-8.95368	-3.41773	-2.08904
C	-7.5559	-1.80013	-2.0937
H	-10.1837	-2.64396	-0.09943
C	-6.0014	0.065674	-1.82397
C	-4.89381	0.46468	-2.12603
C	-3.59414	0.755101	-2.35526
C	10.0044	-2.27701	0.252395
H	11.05492	-2.51554	0.057239
H	9.408034	-2.94699	-0.37582
C	9.683147	-2.55795	1.729473
H	8.644061	-2.27263	1.925727
H	10.30208	-1.90939	2.36022
C	9.893793	-4.02028	2.121507
H	9.267896	-4.65629	1.483114
H	10.9316	-4.3098	1.913727
C	9.571439	-4.30457	3.589609
H	10.22247	-3.69502	4.228853
H	8.545737	-3.98124	3.803716
C	9.722906	-5.77874	3.971483
H	10.74739	-6.1046	3.755826
H	9.070062	-6.38367	3.330985
C	9.395032	-6.05326	5.440122
H	9.508683	-7.11233	5.685825
H	8.365152	-5.76556	5.672557
H	10.05405	-5.48452	6.103265
C	8.843959	3.314794	-1.03895
H	8.184516	3.380897	-1.91011
H	9.767734	3.841705	-1.2966

C	8.168472	4.020435	0.149814
H	8.842946	3.990546	1.013281
H	7.273447	3.45855	0.435383
C	7.783497	5.467137	-0.16046
H	8.678705	6.02919	-0.4558
H	7.113241	5.481401	-1.02889
C	7.104388	6.175213	1.013357
H	6.212082	5.609258	1.308973
H	7.77431	6.162196	1.882507
C	6.705563	7.620542	0.705591
H	6.036706	7.631316	-0.16314
H	7.597776	8.185148	0.40967
C	6.02512	8.318419	1.884584
H	5.750601	9.347455	1.638091
H	6.684775	8.349308	2.757226
H	5.112621	7.791486	2.179739
C	9.72584	-0.84528	-0.1315
C	10.71608	0.138478	-0.05634
C	8.448583	-0.45667	-0.52091
H	7.657378	-1.19436	-0.59328
C	8.149266	0.881908	-0.82791
C	10.43171	1.466497	-0.36224
H	11.21988	2.209591	-0.30273
C	9.156467	1.870246	-0.75132
C	6.824028	1.210144	-1.20525
H	11.72197	-0.14003	0.240138
C	5.665713	1.432043	-1.50063
C	4.353562	1.587083	-1.78945
C	3.156948	1.630715	-2.00127
C	-3.37794	0.225584	1.157821
C	-1.98737	-0.02509	0.862971
H	-1.26576	0.76251	1.036789
C	-1.58912	-1.21185	0.358062
H	-0.54588	-1.37941	0.126845
C	-4.33351	-0.82002	0.878813
H	-5.38291	-0.63831	1.069068
C	-3.93151	-2.0159	0.398212
H	-4.65713	-2.79449	0.20331
C	-2.54066	-2.26881	0.10825
C	-2.13161	-3.48569	-0.40779
C	-3.77968	1.427469	1.713159

C	-3.04745	-4.54867	-0.63293
C	-0.77575	-3.73063	-0.75677
C	-5.15072	1.716388	1.952637
C	-2.84119	2.421818	2.100472
N	-3.80184	-5.4057	-0.81242
N	0.32613	-3.91311	-1.05479
N	-2.06698	3.214286	2.429331
N	-6.26991	1.945257	2.130301

P6P6P6-TCNQ-7

Electronic Energy (EE):	-3094.031089	Hartree
EE + Thermal Free Energy Correction:	-3092.730260	Hartree
E (Thermal):	965.038	kcal/mol
Entropy (S):	500.915	cal/mol-kelvin

Imaginary Frequencies: 0

C	-2.89987	-2.51708	0.117683
H	-3.7958	-2.47161	0.745039
H	-2.23725	-3.26698	0.560083
C	-3.3075	-2.96554	-1.29592
H	-2.41433	-3.01604	-1.92874
H	-3.95862	-2.20461	-1.73669
C	-4.02548	-4.31509	-1.29963
H	-3.35097	-5.0904	-0.9144
H	-4.86912	-4.27204	-0.60132
C	-4.53377	-4.72207	-2.68412
H	-5.24422	-3.96599	-3.04158
H	-3.69793	-4.71208	-3.39508
C	-5.20334	-6.09829	-2.71453
H	-6.03546	-6.11227	-2.00297
H	-4.49016	-6.85338	-2.36289
C	-5.71465	-6.48242	-4.10397
H	-6.18489	-7.4693	-4.09972
H	-4.89819	-6.50493	-4.83227
H	-6.45592	-5.76142	-4.46208
C	-0.19184	2.677944	0.012017
H	0.648346	2.678969	0.712982
H	-0.87182	3.474484	0.327622

C	0.338953	2.978065	-1.40239
H	-0.50875	3.180893	-2.06678
H	0.835597	2.089316	-1.79941
C	1.316895	4.151914	-1.42057
H	0.85431	5.029762	-0.95088
H	2.179617	3.893601	-0.795
C	1.799355	4.525945	-2.82306
H	2.232647	3.64216	-3.30625
H	0.935177	4.810545	-3.43656
C	2.822971	5.664268	-2.83273
H	3.711006	5.357848	-2.26981
H	2.405351	6.527511	-2.30024
C	3.240006	6.081564	-4.24322
H	3.968607	6.896006	-4.2189
H	2.376531	6.419873	-4.82472
H	3.700868	5.246344	-4.7765
C	-2.2104	-1.17791	0.128616
C	-2.94841	0.024089	-0.00118
C	-0.83092	-1.0873	0.231952
H	-0.24527	-1.99318	0.335547
C	-0.1602	0.148035	0.210089
C	-2.27699	1.26014	-0.01634
H	-2.8637	2.166185	-0.11147
C	-0.89774	1.350705	0.084992
C	1.248017	0.192531	0.319611
C	9.44269	-1.43511	1.545366
H	10.42957	-1.38229	1.077538
H	8.982178	-2.37111	1.212704
C	9.61027	-1.48663	3.073673
H	8.619796	-1.52738	3.540853
H	10.07093	-0.55233	3.414049
C	10.45059	-2.67841	3.53405
H	9.985625	-3.60703	3.180321
H	11.43748	-2.63167	3.05726
C	10.6232	-2.74443	5.052685
H	11.08829	-1.81539	5.405984
H	9.635409	-2.78986	5.528469
C	11.46227	-3.93719	5.517746
H	12.44863	-3.89058	5.041412
H	10.99672	-4.86427	5.163226
C	11.6289	-3.9956	7.037165

H	12.23122	-4.85545	7.341457
H	10.65845	-4.07502	7.536562
H	12.12109	-3.09376	7.413969
C	6.155825	3.159103	0.03289
H	5.346514	2.850352	-0.6337
H	6.756038	3.889477	-0.51667
C	5.520421	3.832293	1.261487
H	6.308708	4.156108	1.949994
H	4.919316	3.092224	1.799267
C	4.641306	5.018307	0.864424
H	5.262274	5.798232	0.406624
H	3.943086	4.695875	0.085097
C	3.844183	5.611906	2.025808
H	3.227108	4.824625	2.47707
H	4.531038	5.949469	2.812278
C	2.943841	6.775132	1.601545
H	2.271903	6.43382	0.805963
H	3.561235	7.565741	1.159032
C	2.122367	7.349904	2.756493
H	1.490026	8.177999	2.425741
H	2.771565	7.724263	3.55399
H	1.470785	6.586567	3.192787
C	8.601971	-0.26828	1.097951
C	9.181301	0.969552	0.793993
C	7.216187	-0.377	1.030963
H	6.738836	-1.32418	1.254231
C	6.4089	0.715188	0.674402
C	8.39229	2.060991	0.448656
H	8.862278	3.011373	0.219202
C	7.002938	1.966464	0.378604
H	10.26034	1.074176	0.832309
C	5.005386	0.548913	0.5905
C	3.800161	0.411959	0.502475
C	2.456991	0.283954	0.411652
C	-12.5754	1.963334	-0.23797
H	-13.4759	1.919829	-0.85947
H	-12.0325	2.866424	-0.53533
C	-12.9863	2.08447	1.2388
H	-12.0825	2.120368	1.857665
H	-13.522	1.175339	1.535719
C	-13.855	3.311011	1.521189

H	-13.3137	4.216126	1.218047
H	-14.7548	3.270616	0.894352
C	-14.2666	3.435857	2.989481
H	-14.8079	2.530649	3.292485
H	-13.3666	3.475133	3.616041
C	-15.1352	4.662859	3.27724
H	-16.0338	4.62263	2.650216
H	-14.5931	5.566419	2.974207
C	-15.5413	4.778034	4.747577
H	-16.1599	5.661603	4.925054
H	-14.6613	4.851935	5.39384
H	-16.1126	3.901293	5.067583
C	-9.28099	-2.80274	-1.0847
H	-8.4066	-2.5628	-1.69831
H	-9.85984	-3.552	-1.6334
C	-8.79871	-3.40547	0.245744
H	-9.66809	-3.65057	0.866271
H	-8.22907	-2.64827	0.793053
C	-7.93337	-4.65006	0.047451
H	-8.52565	-5.43451	-0.4407
H	-7.11803	-4.41042	-0.64469
C	-7.34679	-5.19053	1.353036
H	-6.71394	-4.41817	1.808298
H	-8.15922	-5.37569	2.067162
C	-6.53014	-6.47326	1.17706
H	-5.72297	-6.29338	0.459444
H	-7.16664	-7.24658	0.730692
C	-5.93889	-6.98928	2.489939
H	-5.3642	-7.90673	2.338006
H	-6.72593	-7.20542	3.218924
H	-5.27024	-6.2473	2.937141
C	-11.7207	0.748234	-0.5001
C	-12.2983	-0.48897	-0.7997
C	-10.3348	0.815466	-0.40056
H	-9.8544	1.760328	-0.17339
C	-9.52831	-0.31983	-0.59154
C	-11.5102	-1.61882	-0.99477
H	-11.9822	-2.56555	-1.23515
C	-10.1214	-1.56682	-0.89866
C	-8.1208	-0.20497	-0.47449
H	-13.3771	-0.5657	-0.88789

C	-6.91273	-0.12681	-0.36143
C	-5.5676	-0.05745	-0.22911
C	-4.35765	-0.0089	-0.11617
C	6.03515	0.303414	-2.58765
C	5.665406	-1.04703	-2.23269
H	4.615726	-1.30834	-2.20972
C	6.606849	-1.96502	-1.92716
H	6.319608	-2.97445	-1.66179
C	7.435212	0.648751	-2.57514
H	7.721182	1.664232	-2.81462
C	8.377409	-0.27136	-2.27417
H	9.423872	0.001133	-2.26619
C	8.011194	-1.62576	-1.94251
C	8.976358	-2.57116	-1.64304
C	5.072621	1.234288	-2.93312
C	10.35896	-2.24341	-1.64898
C	8.633985	-3.90053	-1.27663
C	5.418076	2.563938	-3.29939
C	3.689265	0.912413	-2.95989
N	11.47774	-1.95212	-1.64471
N	8.340322	-4.97399	-0.9631
N	2.564479	0.648769	-2.99553
N	5.71027	3.643903	-3.59014

P6P6P6-TCNQ-8

Electronic Energy (EE):	-3094.031009	Hartree
EE + Thermal Free Energy Correction:	-3092.727732	Hartree
E (Thermal):	965.006	kcal/mol
Entropy (S):	495.656	cal/mol-kelvin

Imaginary Frequencies: 0

C	-3.75009	2.079239	-1.50078
H	-4.44613	1.812894	-2.30249
H	-3.35581	3.070621	-1.74027
C	-4.53289	2.13609	-0.17363
H	-3.93585	2.665393	0.577353
H	-4.67666	1.120666	0.203879
C	-5.90204	2.799893	-0.33038
H	-5.77818	3.830083	-0.68782

H	-6.45936	2.267558	-1.11096
C	-6.72642	2.799698	0.958076
H	-6.8374	1.766965	1.311483
H	-6.17953	3.334971	1.744529
C	-8.11379	3.425492	0.794027
H	-8.66157	2.884	0.013293
H	-8.00663	4.453915	0.4296
C	-8.92955	3.422392	2.087697
H	-9.91699	3.867036	1.942171
H	-8.42135	3.988714	2.873939
H	-9.07471	2.403188	2.457822
C	0.605586	-1.84879	-1.49412
H	1.346075	-1.518	-2.22687
H	0.22767	-2.81218	-1.84699
C	1.3063	-2.03685	-0.13154
H	0.704579	-2.69627	0.503646
H	1.365131	-1.07713	0.387706
C	2.721552	-2.59571	-0.29118
H	2.683218	-3.5503	-0.83141
H	3.289996	-1.90682	-0.92696
C	3.466569	-2.78813	1.030539
H	3.490757	-1.8347	1.573666
H	2.91347	-3.48843	1.668373
C	4.897916	-3.2971	0.840247
H	5.446385	-2.58115	0.218412
H	4.874745	-4.23539	0.273049
C	5.6529	-3.51382	2.152026
H	6.670204	-3.87174	1.973729
H	5.146326	-4.25298	2.77915
H	5.718516	-2.58575	2.727502
C	-2.62371	1.078026	-1.49129
C	-2.90104	-0.31146	-1.56785
C	-1.29873	1.471956	-1.39758
H	-1.0589	2.526959	-1.33606
C	-0.24621	0.539697	-1.38069
C	-1.84747	-1.24169	-1.5657
H	-2.08614	-2.29592	-1.64104
C	-0.52097	-0.84892	-1.47623
C	1.092438	0.968366	-1.24259
C	8.438833	-0.81928	1.862475
H	9.525142	-0.8965	1.76119

H	8.014656	-1.74969	1.475089
C	8.084614	-0.70211	3.353605
H	6.995958	-0.62828	3.455976
H	8.491834	0.235716	3.747842
C	8.603777	-1.88265	4.174798
H	8.231246	-2.81747	3.740344
H	9.697057	-1.92537	4.095861
C	8.201491	-1.81512	5.648877
H	8.571256	-0.87878	6.085682
H	7.107317	-1.77415	5.723245
C	8.718261	-2.99653	6.473386
H	9.811348	-3.03614	6.399295
H	8.349647	-3.93022	6.032683
C	8.306037	-2.92579	7.944693
H	8.687634	-3.78033	8.50942
H	7.21661	-2.91772	8.047963
H	8.688966	-2.01611	8.417344
C	6.394573	3.860629	-0.99362
H	5.719863	3.584715	-1.80629
H	7.216158	4.426062	-1.44347
C	5.634453	4.762803	-0.00512
H	6.315092	5.077228	0.794329
H	4.842408	4.177203	0.473012
C	5.023127	5.988641	-0.68425
H	5.817009	6.573532	-1.16552
H	4.35792	5.656977	-1.49059
C	4.244949	6.885482	0.280209
H	3.452891	6.297562	0.760947
H	4.910671	7.216339	1.087574
C	3.623869	8.110468	-0.39542
H	2.961026	7.777771	-1.20278
H	4.416098	8.697514	-0.8749
C	2.843789	8.99877	0.575334
H	2.411157	9.864743	0.067538
H	3.49122	9.370637	1.375446
H	2.025337	8.444157	1.044496
C	7.935658	0.348907	1.055025
C	8.760438	1.443906	0.768462
C	6.61513	0.396815	0.624299
H	5.951695	-0.43648	0.821278
C	6.106266	1.510996	-0.06643

C	8.265118	2.556723	0.094715
H	8.9227	3.395572	-0.10661
C	6.940897	2.624725	-0.33176
H	9.799289	1.421411	1.079589
C	4.751934	1.491024	-0.47316
C	3.577586	1.39544	-0.77837
C	2.268217	1.218977	-1.0578
C	-12.1538	1.168386	-0.29058
H	-13.1931	1.171009	-0.63447
H	-11.673	2.045639	-0.73625
C	-12.1205	1.304002	1.240459
H	-11.08	1.246574	1.576855
H	-12.6333	0.444583	1.687574
C	-12.7477	2.604105	1.743389
H	-12.2361	3.454317	1.274951
H	-13.7926	2.659662	1.412973
C	-12.6861	2.753948	3.264473
H	-13.219	1.917413	3.733696
H	-11.6428	2.672415	3.592922
C	-13.2703	4.074554	3.771117
H	-14.313	4.158127	3.442363
H	-12.735	4.907554	3.300013
C	-13.1989	4.215868	5.292536
H	-13.6203	5.167161	5.627748
H	-12.1633	4.167822	5.642911
H	-13.7524	3.412734	5.788568
C	-9.40291	-3.73267	-1.93112
H	-8.6191	-3.50959	-2.66183
H	-10.1068	-4.41615	-2.41571
C	-8.76582	-4.43177	-0.71756
H	-9.55361	-4.69373	-0.0019
H	-8.10451	-3.72481	-0.20654
C	-7.97418	-5.68213	-1.10139
H	-8.63387	-6.38837	-1.62152
H	-7.19393	-5.40551	-1.8213
C	-7.33009	-6.37915	0.098508
H	-6.67435	-5.66961	0.6186
H	-8.10978	-6.65686	0.819178
C	-6.52562	-7.62527	-0.27929
H	-5.74777	-7.34588	-0.99973
H	-7.18158	-8.33359	-0.79922

C	-5.88344	-8.31247	0.927141
H	-5.31523	-9.19776	0.629717
H	-6.64232	-8.63035	1.648712
H	-5.19829	-7.6353	1.446331
C	-11.4601	-0.08316	-0.76756
C	-12.1624	-1.27054	-0.99158
C	-10.0804	-0.09805	-0.94306
H	-9.50521	0.806427	-0.78101
C	-9.40072	-1.26586	-1.3288
C	-11.5	-2.43238	-1.3788
H	-12.0691	-3.33943	-1.5532
C	-10.1187	-2.46236	-1.55534
C	-7.99279	-1.21675	-1.47683
H	-13.24	-1.28391	-0.86607
C	-6.78358	-1.12982	-1.56985
C	-5.44278	-0.96218	-1.62936
C	-4.24773	-0.73658	-1.63183
C	6.459108	-0.22553	-2.8737
C	7.70733	0.478171	-2.72757
H	7.780035	1.490586	-3.10121
C	8.770281	-0.10563	-2.12852
H	9.69789	0.438029	-2.0145
C	6.383481	-1.57997	-2.38267
H	5.451941	-2.12095	-2.48848
C	7.445484	-2.16361	-1.7859
H	7.37101	-3.17554	-1.40934
C	8.683799	-1.44554	-1.60699
C	9.741908	-2.02019	-0.92038
C	5.357585	0.376975	-3.45684
C	9.643424	-3.32037	-0.35776
C	10.93464	-1.29362	-0.66703
C	4.127922	-0.31282	-3.62332
C	5.405758	1.712043	-3.94046
N	9.529706	-4.37087	0.112405
N	11.88152	-0.66726	-0.44636
N	5.460759	2.799081	-4.32945
N	3.136382	-0.88869	-3.76809

P6P6P6-TCNQ-9

Electronic Energy (EE): -3094.026956 Hartree

EE + Thermal Free Energy Correction: -3092.726436 Hartree

E (Thermal): 964.817 kcal/mol

Entropy (S): 500.825 cal/mol-kelvin

Imaginary Frequencies: 0

C	3.720974	-2.12024	-1.1452
H	4.369941	-1.95223	-2.01048
H	3.270153	-3.10798	-1.27438
C	4.590652	-2.10612	0.127891
H	4.024862	-2.54294	0.958341
H	4.803149	-1.07134	0.407949
C	5.916477	-2.84546	-0.06038
H	5.725008	-3.89419	-0.32084
H	6.441535	-2.40569	-0.91726
C	6.825202	-2.77973	1.168279
H	7.00321	-1.72815	1.42566
H	6.310235	-3.22247	2.030188
C	8.170742	-3.48231	0.97012
H	8.687464	-3.03299	0.113425
H	7.995981	-4.53072	0.701346
C	9.070667	-3.41271	2.204747
H	10.02619	-3.91487	2.035309
H	8.59247	-3.88771	3.066645
H	9.283747	-2.37484	2.477302
C	-0.45012	2.006377	-1.15423
H	-1.28257	1.661779	-1.77442
H	-0.06364	2.91462	-1.62481
C	-0.9947	2.341276	0.248637
H	-0.23732	2.89835	0.811602
H	-1.16644	1.412433	0.798289
C	-2.30642	3.127513	0.190822
H	-2.13119	4.110974	-0.26312
H	-2.99898	2.605161	-0.47755
C	-2.96434	3.302058	1.560539
H	-3.13126	2.31213	2.004312
H	-2.27993	3.827775	2.238389
C	-4.29528	4.05632	1.504685
H	-4.9744	3.535056	0.818443
H	-4.13187	5.047891	1.066994

C	-4.95946	4.202552	2.874657
H	-5.9101	4.737351	2.808113
H	-4.31497	4.753699	3.565894
H	-5.15794	3.22369	3.321483
C	2.64055	-1.06966	-1.14428
C	2.968974	0.294411	-1.35802
C	1.310899	-1.39096	-0.92611
H	1.031228	-2.4247	-0.76069
C	0.302769	-0.41068	-0.91556
C	1.958386	1.271337	-1.36222
H	2.23548	2.303374	-1.54166
C	0.626572	0.951599	-1.1478
C	-1.04108	-0.75743	-0.6597
C	-8.49076	2.157879	1.183594
H	-9.52296	2.251271	0.831305
H	-7.90049	2.902838	0.639228
C	-8.42938	2.477591	2.685926
H	-7.40227	2.331104	3.035826
H	-9.04553	1.754463	3.232246
C	-8.88254	3.900289	3.012819
H	-8.28996	4.610713	2.422844
H	-9.92367	4.035445	2.694955
C	-8.75132	4.246192	4.496923
H	-9.35142	3.543553	5.088629
H	-7.71063	4.097224	4.810626
C	-9.17643	5.678568	4.828212
H	-10.2162	5.828093	4.514481
H	-8.57559	6.377508	4.234228
C	-9.03473	6.01603	6.313391
H	-9.34296	7.043679	6.522462
H	-7.99758	5.905019	6.644225
H	-9.65003	5.35189	6.928016
C	-6.45413	-3.21299	0.038709
H	-5.6932	-3.16477	-0.74651
H	-7.26406	-3.84363	-0.3397
C	-5.82905	-3.87075	1.28029
H	-6.59481	-3.96997	2.057843
H	-5.05847	-3.20677	1.684668
C	-5.21461	-5.23615	0.9712
H	-5.9859	-5.89955	0.559571
H	-4.45956	-5.11879	0.184159

C	-4.57461	-5.89921	2.192106
H	-3.80588	-5.23289	2.603146
H	-5.32922	-6.01622	2.980242
C	-3.94907	-7.26214	1.885631
H	-3.19713	-7.14285	1.096816
H	-4.71815	-7.92713	1.475109
C	-3.30766	-7.91554	3.110986
H	-2.86906	-8.88598	2.864553
H	-4.04538	-8.07472	3.90345
H	-2.51307	-7.2852	3.521797
C	-7.9705	0.778184	0.869718
C	-8.82863	-0.32367	0.800888
C	-6.61289	0.561214	0.664864
H	-5.91772	1.392008	0.70396
C	-6.10853	-0.72287	0.385103
C	-8.33888	-1.60009	0.530454
H	-9.02918	-2.43477	0.471757
C	-6.98438	-1.83212	0.313943
H	-9.89321	-0.17685	0.947153
C	-4.72636	-0.87404	0.138964
C	-3.53676	-0.94058	-0.10945
C	-2.22093	-0.90574	-0.40379
C	12.2111	-1.53106	-0.58077
H	13.22142	-1.61428	-0.99401
H	11.65989	-2.4154	-0.91763
C	12.28351	-1.54216	0.954901
H	11.27402	-1.40508	1.356653
H	12.86608	-0.67724	1.292533
C	12.88645	-2.82846	1.519241
H	12.30226	-3.68575	1.161651
H	13.89961	-2.96474	1.120599
C	12.93358	-2.85221	3.048015
H	13.5394	-2.01017	3.40577
H	11.92338	-2.6894	3.442809
C	13.49223	-4.15704	3.620221
H	14.50157	-4.32201	3.224927
H	12.88386	-4.99553	3.260789
C	13.53097	-4.17176	5.149288
H	13.93158	-5.11415	5.531799
H	12.52873	-4.04071	5.568552
H	14.15812	-3.36202	5.534648

C	9.573267	3.355401	-2.43689
H	8.732112	3.113646	-3.09424
H	10.27186	3.961689	-3.02148
C	9.052533	4.181131	-1.24765
H	9.898291	4.460052	-0.60866
H	8.396551	3.552008	-0.63761
C	8.293613	5.434673	-1.68371
H	8.947371	6.061937	-2.30311
H	7.45428	5.140309	-2.32592
C	7.765163	6.258879	-0.50821
H	7.11495	5.628388	0.111184
H	8.603975	6.554494	0.134753
C	6.994116	7.509304	-0.93796
H	6.156874	7.211926	-1.5804
H	7.644455	8.138309	-1.55745
C	6.467934	8.324783	0.244498
H	5.92152	9.21058	-0.08994
H	7.288111	8.661504	0.886013
H	5.789845	7.728226	0.862429
C	11.54199	-0.28795	-1.11183
C	12.27902	0.841622	-1.47816
C	10.15551	-0.21826	-1.19721
H	9.553379	-1.07728	-0.92385
C	9.503244	0.947754	-1.63272
C	11.64368	2.000658	-1.9157
H	12.23897	2.861514	-2.2012
C	10.25609	2.085238	-2.00374
C	8.087728	0.956929	-1.6839
H	13.36229	0.811143	-1.42485
C	6.872323	0.922012	-1.68966
C	5.524804	0.815348	-1.64592
C	4.324019	0.648084	-1.54948
C	-9.23673	-1.11472	-2.56132
C	-8.57127	0.152139	-2.39311
H	-9.15918	1.014801	-2.11075
C	-7.235	0.264799	-2.56101
H	-6.74563	1.218326	-2.41442
C	-8.4417	-2.24576	-2.97497
H	-8.93436	-3.19592	-3.13758
C	-7.1031	-2.13746	-3.12196
H	-6.51287	-2.99983	-3.40408

C	-6.42964	-0.87994	-2.90081
C	-5.052	-0.78046	-2.99965
C	-10.5916	-1.24994	-2.3008
C	-4.23801	-1.91512	-3.26241
C	-4.38468	0.465326	-2.84877
C	-11.2613	-2.49295	-2.45028
C	-11.3472	-0.16289	-1.78754
N	-3.59204	-2.852	-3.46525
N	-3.85752	1.488692	-2.74558
N	-11.9242	0.733155	-1.3379
N	-11.7876	-3.51592	-2.56898
